# Multiomics Analysis Reveals the Prognostic Non-tumor Cell Landscape in Glioblastoma Niches

**DOI:** 10.3389/fgene.2021.741325

**Published:** 2021-09-16

**Authors:** Zixuan Xiao, Wei Zhang, Guanzhang Li, Wendong Li, Lin Li, Ting Sun, Yufei He, Guang Liu, Lu Wang, Xiaohan Han, Hao Wen, Yong Liu, Yifan Chen, Haoyu Wang, Jing Li, Yubo Fan, Jing Zhang

**Affiliations:** ^1^Key Laboratory for Biomechanics and Mechanobiology of Ministry of Education, Beijing Advanced Innovation Centre for Biomedical Engineering, School of Engineering Medicine, School of Biological Science and Medical Engineering, Beihang University, Beijing, China; ^2^Department of Molecular Neuropathology, Beijing Neurosurgical Institute, Capital Medical University, Beijing, China; ^3^Department of Neurosurgery, Beijing Tiantan Hospital, Capital Medical University, Beijing, China

**Keywords:** glioblastoma, tumor microenvironment, immunology, prognosis, tumor-infiltrating cells

## Abstract

A comprehensive characterization of non-tumor cells in the niches of primary glioblastoma is not fully established yet. This study aims to present an overview of non-malignant cells in the complex microenvironment of glioblastoma with detailed characterizations of their prognostic effects. We curate 540 gene signatures covering a total of 64 non-tumor cell types. Cell type-specific expression patterns are interrogated by normalized enrichment score across four large gene expression profiling cohorts of glioblastoma with a total number of 967 cases. The glioblastoma multiforms (GBMs) in each cohort are hierarchically clustered into negative or positive immune response classes with significantly different overall survival. Our results show that astrocytes, macrophages, monocytes, NKTs, and MSC are risk factors, while CD8 T cells, CD8 naive T cells, and plasma cells are protective factors. Moreover, we find that the immune system and organogenesis are uniformly enriched in negative immune response clusters, in contrast to the enrichment of nervous system in positive immune response clusters. Mesenchymal differentiation is also observed in the negative immune response clusters. High enrichment status of macrophages in negative immune response clusters is independently validated by analyzing scRNA-seq data from eight high-grade gliomas, revealing that negative immune response samples comprised 46.63 to 55.12% of macrophages, whereas positive immune response samples comprised only 1.70 to 8.12%, with IHC staining of samples from six short-term and six long-term survivors of GBMs confirming the results.

## Highlights

1. A comprehensive characterization of non-tumor cells in the niches of primary glioblastoma.
2. Astrocytes, macrophages, monocytes, NKTs, and MSC are risk factors, while CD8 T cells, CD8 naive T cells, and plasma cells are protective factors.
3. Mesenchymal differentiation is observed in the negative immune response clusters.
4. High enrichment status of macrophages is in negative immune response clusters of glioblastomas.

## Introduction

Gliomas account for 70% of all brain tumors (Ohgaki and Kleihues, 2005) and are categorized into four types: Grade I pilocytic astrocytoma and grade II astrocytoma are low-grade gliomas, whereas grade III anaplastic astrocytoma and grade IV glioblastoma multiform (GBM) are malignant tumors (Kleihues et al., 1993). The GBMs have poor prognosis with a median survival rate of 1year after diagnosis and a 2-year survival rate of only 12.7 to 19.8% according to the SEER database.

Categorization of gliomas previously focused on histological features (Bailey and Cushing, 1927); however, characterization methods have shifted toward high-resolution molecular profiling, including identification of isocitrate dehydrogenase (IDH) mutation, co-deletion of chromosomal arms, O6-methylguanine-DNA methyltransferase (*MGMT*) promoter methylation, and miR-181d expression (Jiang et al., 2016). Additionally, new stratifications have been proposed using gene expression profiles or specific gene mutations (Phillips et al., 2006; Ceccarelli et al., 2016), methylation status (Hegi et al., 2005; Shah et al., 2011), and the presence of neoantigens (Zhang et al., 2019; Sun et al., 2021). Numerous studies have focused on interpreting the RNA-seq profiles of gliomas in an attempt to elucidate their dynamics and mechanisms, with studies on recurrent glioblastoma able to distinguish comprehensive transcriptome profiling in the malignant progression of human gliomas (Zhao et al., 2017) and find critical clues of *MET*-related mutations (Hu et al., 2018) and oncogenic fusions (Bao et al., 2014). The findings of these studies have markedly advanced the investigation of GBM and facilitated prognostic and therapeutic developments, but the highly heterogeneous nature of GBM still often leads to the failure of extensive treatment regimens.

The complexity of GBM components and the immune microenvironment has attracted significant attention in recent years, with categorizations based on molecular profiling revealing tissue similarities between proneural, proliferative, and mesenchymal-type gliomas, respectively (Phillips et al., 2006). Certain immune components, such as tumor-associated macrophages (TAMs), have been identified as regulators of the proneural-to-mesenchymal transition (Bhat et al., 2013) and contributors to immunosuppression (Gabrilovich, 2017), thus leading to poor prognosis. However, a comprehensive characterization of non-tumor cells in the niches of primary glioblastoma has not been fully established. Investigations into the tumor components and immune microenvironment would help unravel the cross-talk between the immune system and cancer cells and allow determination of therapeutic targets for the development of novel cancer treatments.

In this study, we generated a comprehensive non-tumor cell landscape in the microenvironment of GBM by integrating four large-scale gene expression profiling data cohorts of primary glioblastoma with gene signatures covering a total of 64 non-tumor cell types. The GBMs in each cohort are hierarchically clustered into negative or positive immune response classes with significantly different overall survival. Additionally, we investigated the risk levels associated with immune cell types and the enrichment of Gene Ontology (GO) terms. In particular, we confirmed enrichment of a negative prognostic factor (macrophages) in scRNA-seq data of high-grade gliomas and in samples from GBM patients exhibiting short-term survival by immunohistochemical (IHC) staining.

## Materials and Methods

### Gene Expression and Clinical Data

Four cohorts of gene expression profiles of GBM tumor tissues were collected from public domains including Cohort 1 (Wang et al., 2016; Zhang et al., 2019), Cohort 2 (TCGA; RNA sequences; Cancer Genome Atlas Research, 2008), Cohort 3 (REMBRANDT, mRNA microarray; Gusev et al., 2018), and Cohort 4 (TCGA, mRNA microarray; Brennan et al., 2013), respectively. Samples that were not diagnosed as GBM or did not include complete gene expression or clinical data were removed, resulting in 75, 152, 181, and 559 samples in Cohorts 1, 2, 3, and 4, respectively. The single-cell RNAseq data of eight HGGs can be accessed through Gene Expression Omnibus (accession: GSE103224; Yuan et al., 2018). Tumor samples were obtained from 12 glioblastomas, including from six short-term-survival and six long-term-survival patients. All research protocols and ethics comply with the Declaration of Helsinki. Sample collection and data analyses were approved by the Beijing Tiantan Hospital institutional review board (KY 2020–093-02), and written informed consent was obtained from each participant.

### Gene Signatures of Immune Cells

Gene signatures (*n*=540) covering 64 cell types were collected from multiple sources (Bindea et al., 2013; Rooney et al., 2015; Tirosh et al., 2016; Aran et al., 2017; Charoentong et al., 2017). The 64 cell types were further categorized into five groups: hematopoietic stem cells (HSCs) and hematopoietic cells (lymphoid and myeloid lineage), stromal cells, and others, as shown in Supplementary Material 1A,B.

### Generating a Normalized Enrichment Score for Estimating Cell-Enrichment Status

An normalized enrichment score (NES) for the Mann–Whitney–Wilcoxon gene set test was adapted to evaluate the enrichment status of cells (Frattini et al., 2018). The NES was determined as follows:


$$NES=1-\frac{U}{mn}$$

$$U=nm+\frac{m\left(m+1\right)}{2}-T$$

where m is the number of genes in a gene set, n is the number of genes outside the gene set, and T is the sum of the ranks of the genes in the gene set (Zhang et al., 2019).

Given a gene signature, the gene expression data of a glioblastoma tumor sample were separated into two sections comprising genes expressed in the gene signature and the rest of the genes, respectively. The Wilcoxon rank-sum test was then applied to calculate the NES. For each cell signature, the NES value was calculated to quantify the probability that the expression of a gene in the gene signature was greater than the expression of a gene outside of the gene signature. The higher the NES value, the more likely that the cell is enriched in the tumor sample.

### Risk Level for Gene Signatures

Cox regression (proportional hazards regression) in the R was applied for every gene signature in each cohort. The protective factor was defined when the hazard ratio of a gene signature was <1, and the risk factor was defined when this was >1. Signatures with a *p*≤0.05 were defined as significantly associated with survival (addressed as prognostic signatures below), with only prognostic signatures used for further analysis. If all prognostic signatures of one cell type were either protective or risk factors, they were defined as consistent factors, otherwise, inconsistent factors.

### Stratification of Glioblastoma Patients

Hierarchical clustering of GBMs was applied to z-score transformed NESs of these signatures using R. Euclidean distance and complete method were used for clustering, and heat maps were drawn using the R: “pheatmap.” Kaplan–Meier survival analysis was performed using R: “survival” and “survminer.”

### Go Enrichment Analysis

Gene Set Enrichment Analysis (GSEA; Subramanian et al., 2005) was performed upon negative and positive immune response clusters using a total of 6,166 GO terms from the Molecular Signatures Database (MSigDB; Liberzon et al., 2011), including cellular component (cc), molecular function (mf), and biological process (bp), followed by visualization through cytoscape (Shannon et al., 2003). The results are shown in Supplementary Material 2A–D.

### Identification of Non-Transformed Cells From scRNA-Seq Data

For scRNA-seq data, genes expressed in less than or equal to 10 cells were eliminated, followed by a moving average method (Chung et al., 2017) to determine chromosome expression patterns. The number of original molecules per cell was converted to log_2_(cpm+1). The moving average used 100 gene lengths as the window, and the value for the gene in the center of the window was considered the average expression of the window. We used the Seurat package (v.3.0; Butler et al., 2018; Stuart et al., 2019) to analyze the screened data according to standard procedures. Amplification of chromosome 7 and loss of chromosome 10 were used to differentiate malignant (transformed) cells from non-malignant (non-transformed) cells (Weller et al., 2015).

### Determination of Non-Transformed Cell Types

Scibet (Li et al., 2020) was used to predict the identities of the non-transformed cells in the scRNA-seq data. The trained model “30 major human cell types,”^1^ including 30 major human cell types from 42 scRNA-seq datasets, served as the reference for cell type identification.

### Stratification of Single-Cell Gene Expression Samples

To determine whether a sample in the scRNA-seq data was positive or negative immune response, Spearman correlation analysis was applied between the sample in the scRNA-seq cohort and the samples in the four gene expression profiling cohorts, respectively. Only positive correlations were retained, and the mean value of the correlation coefficients in each cohort was calculated. The fold change for a sample in the scRNA-seq data was calculated as the mean correlation coefficient of the sample in the scRNA-seq data involving samples in the positive immune response clusters divided by the mean correlation coefficients of the sample in the scRNA-seq data involving samples in the negative immune response clusters. The fold changes in the correlation coefficients calculated for the four cohorts were multiplied to determine the total fold change. A total fold change >1 indicated that the Spearman correlation coefficient was higher in the positive immune response clusters, and thus, the sample in the scRNA-seq data was determined as positive immune response; otherwise, it was designated as negative immune response (Supplementary Material 3).

### IHC Staining for Macrophage Markers

Tumor samples used for IHC staining were obtained from 12 GBMs, including six short-term-survival and six long-term-survival patients. The surgically removed tumor tissues were stored in formalin immediately after excision and embedded in paraffin within 3days. IHC staining and image capture were performed as previously described (Hu et al., 2018). The primary antibody for the detection of macrophage marker MS4A4A was obtained from Sigma-Aldrich (HPA029323; St. Louis, MO, United States), with staining was performed according to manufacturer instructions. The proportion of positive cells was counted using ImageJ software (v.1.52; National Institutes of Health, Bethesda, MD, United States). Clinical information and IHC staining results are summarized in Supplementary Material 4.

### Statistical Analysis

Values of *p* for NES distributions in negative immune response and positive immune response clusters were calculated using Student’s *t*-test, and those for IHC staining percentages were generated from the Wilcoxon test. All analyses were conducted in R. Values of *p*≤0.05 were determined as statistical significance.

## Results

### Stratification of Glioblastomas Based on Cell Type-Specific Enrichment Status

**Figure 1:**
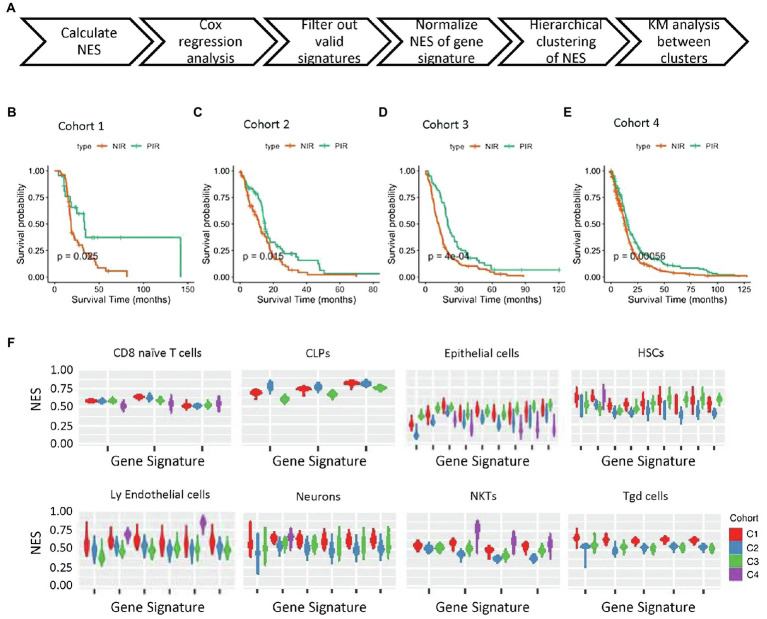
NES-based stratification of patient survival. **(A)** Workflow of NES-based stratification and validation of survival time. **(B–E)** Kaplan–Meier survival curves of the NIR and PIR clusters in the four cohorts (PIR, orange; NIR, green). **(F)** NES distribution of four gene expression profiling cohorts of tumor tissues from GBM patients. Cell types and cohorts are noted. NES, normalized enrichment score; NIR, negative immune response; and PIR, positive immune response.

Based on a total of 540 gene signatures covering 64 cell types (Supplementary Material 1A), we applied the NES algorithm we previously developed (Frattini et al., 2018) to determine the enrichment status of each cell type, followed by filtering the gene signatures with enrichment status correlated with overall survival (prognostic signatures). The workflow for stratifying samples is shown in Figure 1A. Unsupervised hierarchical clustering stratified samples into two significantly different prognostic clusters among the four cohorts (*p*=0.025, *p*=0.015, *p*=0.0004, and *p*=0.00056 for cohort 1–4, respectively; Figures 1B–E; Supplementary Figures 1A–D; Table 1). Clusters with patients exhibiting long-term overall survival were found universally enriched with CD8 T cells, whereas short-term overall survival clusters were characterized by enrichment of “stromal cells,” such as mesenchymal stem cells (MSCs). Therefore, we designated the long- and short-term overall survival clusters as positive and negative immune response, respectively. Additionally, we discovered that the enrichment status calculated from different gene signatures exhibited similar and stable trends for CD8 naïve T cells, common lymphoid progenitors (CLPs), epithelial cells, HSCs, lymphoid endothelial cells, neurons, natural killer T cells (NKTs), and γΔT cells (Figure 1F).

**Table 1:** Hierarchical clustering results for the four cohorts.

Cohort	Sample	Signature	Cell type	PIR	NIR	*p*	Data source (References)
1	75	31	18	22	53	0.02499	Wang et al., 2016; Zhang et al., 2019
2	152	51	24	67	85	0.01462	Cancer Genome Atlas Research, 2008
3	181	57	24	60	121	0.0004	Gusev et al., 2018
4	559	138	46	198	361	0.00056	Brennan et al., 2013

NIR, negative immune response; PIR, positive immune response.

### The Predicted Risk and Protective Landscape of Non-Tumor Cells in the Glioblastoma Microenvironment

**Figure 2:**
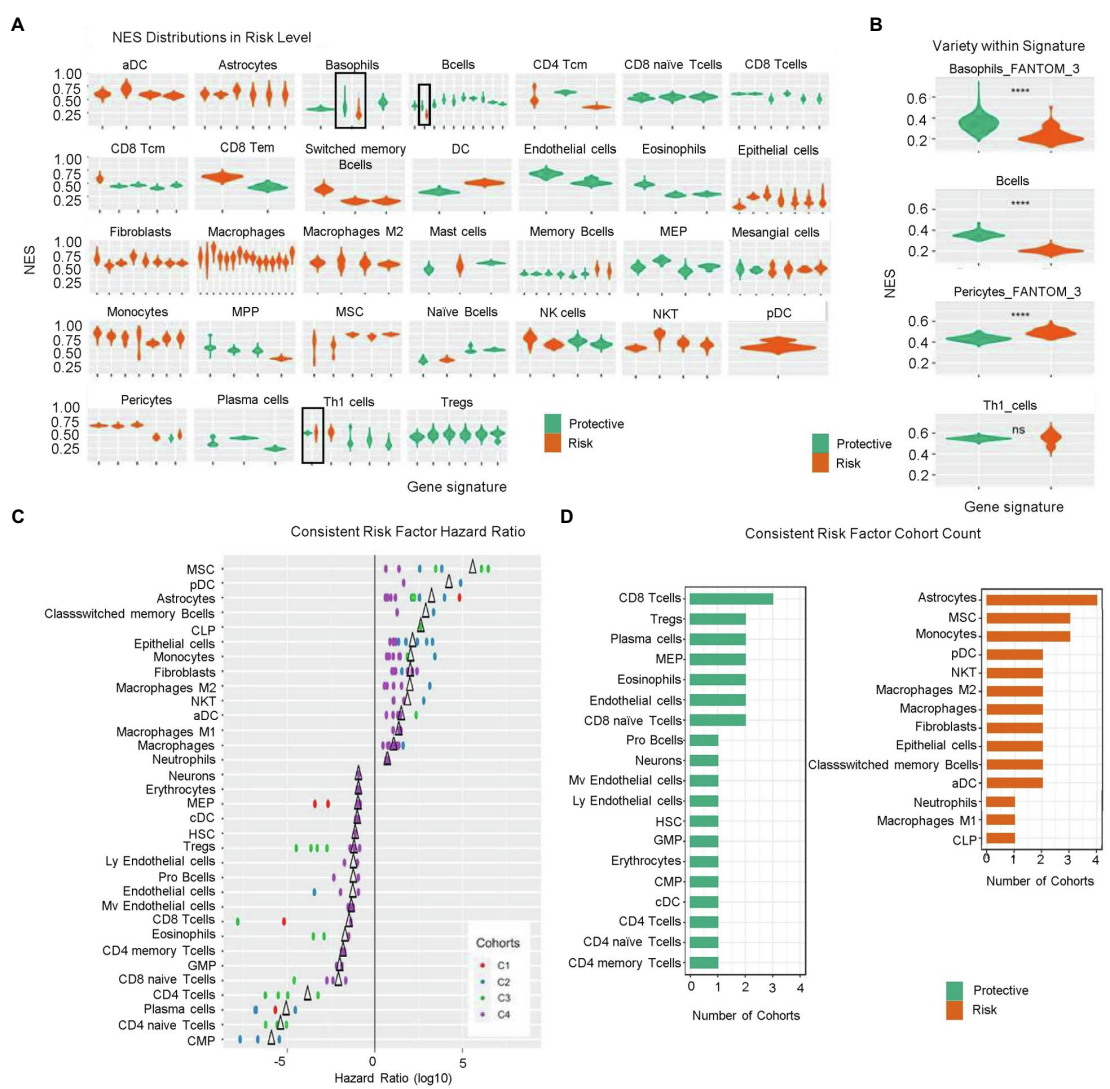
Risk levels according to calculated NESs. **(A)** NES distribution of prognostic signatures as denoted by risk levels (risk factors, orange; protective factors, green). **(B)** Variety of NES distribution within signatures. ns, *p*>0.05; and ^****^p≤0.0001. **(C)** Hazard ratio of consistent risk factors (>1, risk factor; <1, protective factor; and ∆, mean value). **(D)** Group count of consistent risk factors. NES, normalized enrichment score; NIR, negative immune response; ns, not significant; and PIR, positive immune response.

To understand the prognostic effect of different cell types, we estimated associations between the enrichment status of gene signatures and overall survival through Cox regression analysis across four gene expression profiling cohorts. In each cohort, statistically significant gene signatures with a hazard ratio>1 or<1 were defined as risk or protective factors, respectively. We found that risk effects consistently agreed with statistically significant gene signatures for given cell types, including activated dendritic cells (aDCs), astrocytes, class-switched memory (CSM) B cells, epithelial cells, fibroblasts, macrophages, M2 macrophages, monocytes, MSCs, NKTs, and plasmacytoid (p)DCs. By contrast, CD8 naïve T cells, CD8 T cells, endothelial cells, eosinophils, megakaryocyte–erythroid progenitor cells, plasma cells, and regulatory T cells (Tregs) were consistently estimated as being protective. Additionally, basophils, B cells, CD8 central memory T cells, mast cells, multi-potent progenitor cells, memory B cells, naïve B cell, and T helper 1 (Th1) cells were predicted as being protective according to majority of gene signatures across the four cohorts, whereas CD4 central memory T cells, mesangial cells, and pericytes were predicted as a risk by most of the gene signatures. Interestingly, the risk and protective effects of CD8 effector memory T cells, DCs, myocytes, and NK cells were inconsistent according to the different gene signatures (Figure 2A).

Notably, we identified inconsistencies in some estimated risk or protective effects predicted by the gene signatures across the four cohorts. The prognostic effects of enrichment status estimated from one gene signature for basophils, B cells, pericytes, and Th1 cells were inconsistent among the four cohorts (Figures 2A,B); however, basophils, B cells, and pericytes were more likely to manifest an enrichment-dependent effect on survival time, with basophils and B cells being protective when highly enriched and pericytes presenting a risk when highly enriched.

Statistically significant signatures showed consistency across risk levels valued from different perspective, i.e., risk level NES distribution, risk factor hazard ratio, and occurrence cohort count. Figure 2C shows the hazard ratios for cell types demonstrating consistent agreement in their prognostic effects across all corresponding signatures in at least two cohorts. MSCs, pDCs, CSM B cells, and CLPs were consistent risk factors with relatively high hazard ratios in at least two cohorts. Conversely, common myeloid progenitors, CD4 naïve T cells, plasma cells, and CD4 T cells showed hazard ratios <1, suggesting potentially strong protective effects (Figure 2C). Figure 2D shows the group count of consistent risk levels. Astrocytes, MSCs, monocytes, pDCs, NKTs, macrophages, M2 macrophages, fibroblasts, epithelial cells, CSM B cells, and aDCs were consistent risk factors appearing in at least two cohorts, with astrocytes being significantly negatively correlated with overall survival in all four cohorts. CD8 T cells, Tregs, plasma cells, MEPs, eosinophils, endothelial cells, and CD8 naïve T cells were also consistent risk factors, with CD8 T cells most frequently identified in three cohorts; however, for risk factors identified in only two cohorts (i.e., Tregs), more evidence is needed to support these findings.

### Identification of Immune Dysregulation in the Negative Immune Response Cluster

**Figure 3:**
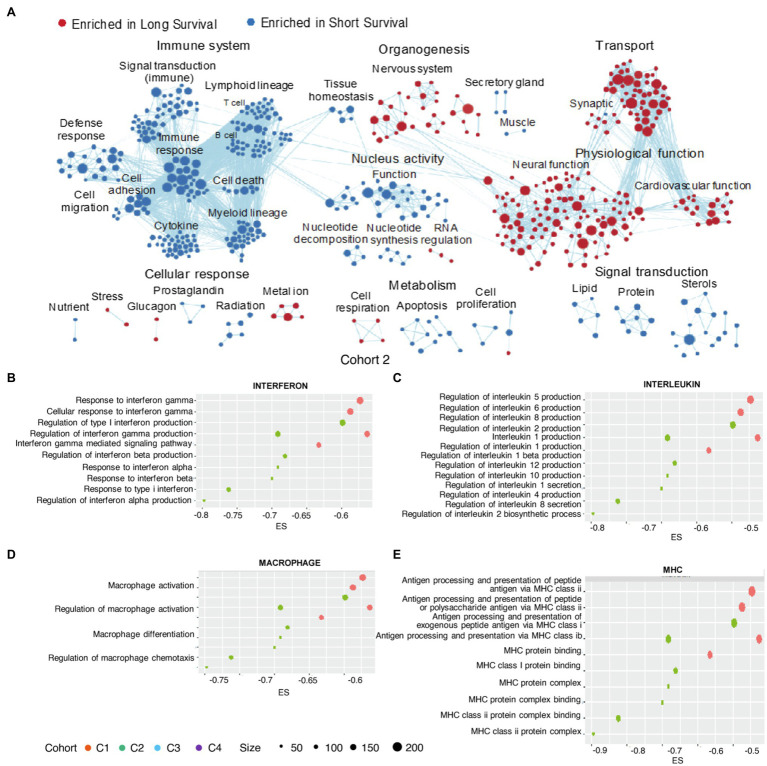
GO enrichment in clusters. Enrichment map of GO terms (selected according to *p*<0.05) aggregated by functions for Cohort 2 **(A)**. Enrichments scores for GO terms (selected according to *p*<0.05) associated with the immune-related **(B)** interferons, **(C)** interleukins, **(D)** macrophages, and **(E)** major histocompatibility complex (MHCs). GO, Gene Ontology; MHC, major histocompatibility complex.

We then performed GSEA for the four cohorts. Enrichment map analysis of dysregulated GO terms revealed that those related to the immune system, metabolism, and organogenesis were highly enriched in all four cohorts (Figure 3A; Supplementary Figures 2A–C; Supplementary Material 2). Specifically, GO terms related to the immune system (defense response, cytokines, myeloid lineage, and lymphoid lineage cell regulation) were enriched in negative immune response clusters, suggesting uniform dysregulation of the immune response in negative immune response clusters. Interferon (IFN)-related GO terms were significantly enriched in the negative immune response group (Figure 3B), consistent with constitutive type I IFNs (IFN-α and IFN-β) facilitating glioma-related immune escape (Silginer et al., 2017), unfavorable prognosis, chemotherapy resistance, and more aggressive immune response (Zhu et al., 2019).

Activities associated with several interleukins (ILs), including IL-6, IL-8, and IL-10, were enriched in negative immune response clusters (Figure 3C), with IL-8 expression negatively correlated with GBMs survival and positively correlated with the expression of genes associated with the glioblastoma-initiating cell phenotype, as well as the possibility of GBM recurrence (Hasan et al., 2019). Additionally, IL-1β contributes to cancer cell stemness, invasiveness, and drug resistance in glioblastoma (Wang et al., 2012; Yeung et al., 2013).

Moreover, we identified macrophage activation, differentiation, and chemotaxis as enriched activities in negative immune response clusters (Figure 3D), consistent with identification of macrophages as risk factors. Downregulation of major histocompatibility complex (MHC)-I and -II molecules is associated with glioma migration and invasion (Zagzag et al., 2005), with their altered expression associated with the negative immune response cluster (Figure 3E).

Majority of nervous system-associated GO terms (nervous system organogenesis in G1, nervous system organogenesis, neural function and synaptic in G2, and nervous system organogenesis in G4) was enriched in the positive immune response cluster (Figure 3A; Supplementary Figures 2B,C), demonstrating that regulation of the nervous system was a shared feature in the positive immune response cluster. This agrees with the proneural subtype of gliomas categorized by molecular profiling, in that this subtype usually demonstrated tissue similarity with adult and fetal brain and biological processes related to neurogenesis (Phillips et al., 2006). Additionally, this glioma subtype is regarded as less malignant relative to other subtypes (e.g., proliferative and mesenchymal; Phillips et al., 2006).

### Mesenchymal Differentiation Characterized in the Negative Immune Response Cluster

**Figure 4:**
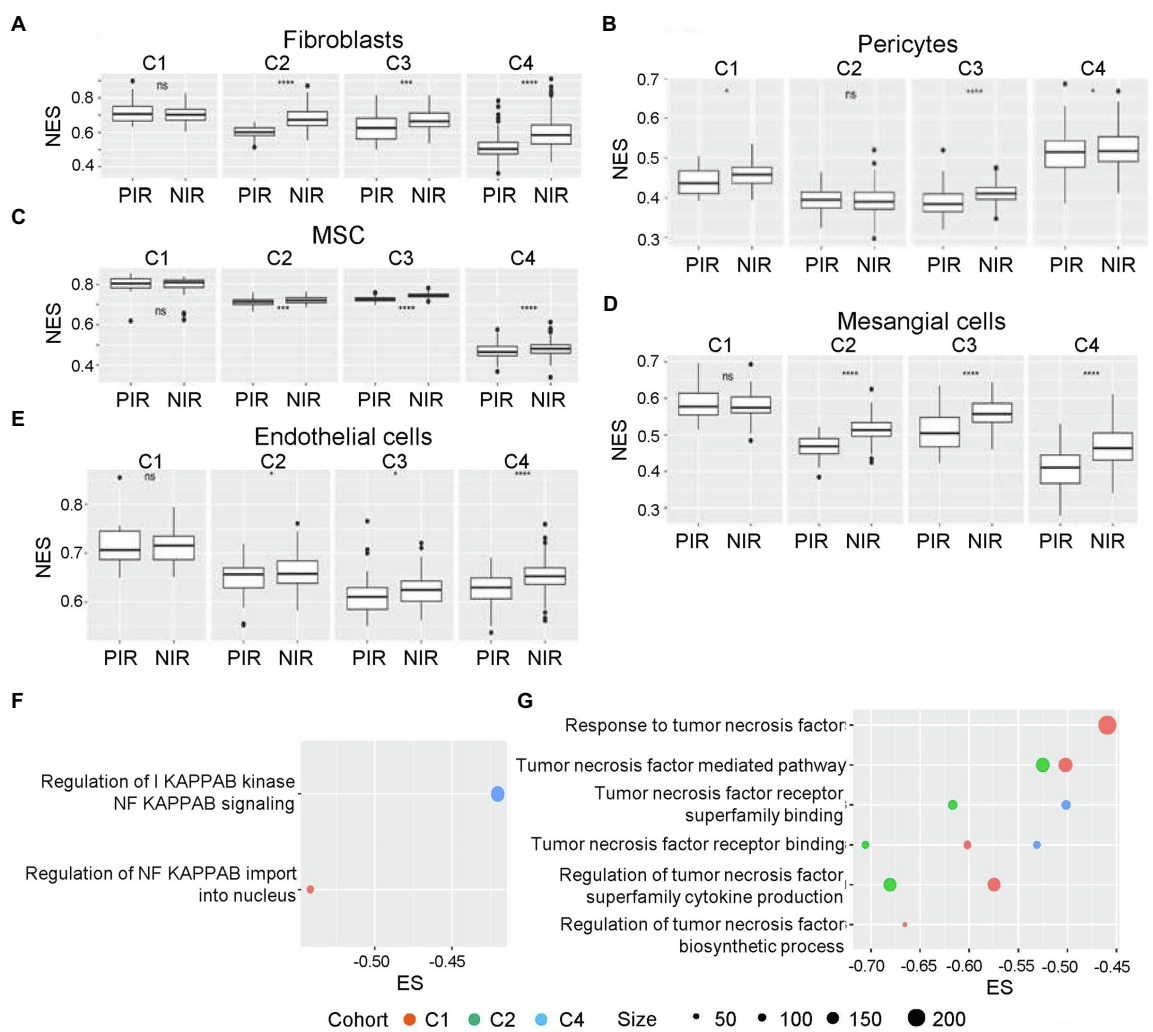
NES distributions in NIR and PIR clusters. **(A)** Fibroblasts, **(B)** pericytes, **(C)** mesenchymal stem cells (MSCs), **(D)** mesangial cells, and **(E)** endothelial cells. ns, p>0.05; ^*^p≤0.05; ^**^p≤0.01; ^***^p≤0.001; and ^****^p≤0.0001. Enrichment scores for GO terms associated with the mesenchymal differentiation-related cytokines (selected according to a *p*<0.05; F) NF-κB and **(G)** tumor necrosis factor (TNF)-α. GO, Gene Ontology; MSC, mesenchymal stem cell; NES, normalized enrichment score; NF-κB, nuclear factor-kappaB; NIR, negative immune response; PIR, positive immune response; and TNF-α, tumor necrosis factor-α.

Gliomas of the mesenchymal subtype are defined by high expression of chitinase 3-like 1 and MET5, as well as a high frequency of neurofibromatosis type 1 (*NF1*) mutation/deletion and low levels of *NF1* mRNA (Verhaak et al., 2010). The negative immune response clusters defined by cell-enrichment analysis shared an obvious similarity with this glioma subtype. We discovered that five stromal cell types (fibroblasts, pericytes, MSC, mesangial cells, and endothelial cells) exhibited a significantly higher NES value in the negative immune response cluster than in the positive immune response cluster in at least three cohorts (Figures 4A–E). Of note, negative immune response clusters with endothelial cells showed higher NESs in three cohorts but distributed between two different signatures (Supplementary Figure 2D). Lymphoid endothelial cells showed higher negative immune response enrichment in one cohort, with no significant differences observed in other cohorts. These results supported tissue similarities between negative immune response clusters and the mesenchymal subtype.

Furthermore, we identified aspects related to mesenchymal differentiation in negative immune response clusters, with enrichment of activities related to tumor necrosis factor (TNF)-α and nuclear factor-kappaB (NF-κB) identified from three cohorts and all four cohorts (Figures 4F,G), respectively. Previous studies of glioma sphere cultures indicated that TNF-α promotes mouse embryonic stem cell differentiation accompanied by increased resistance to radiotherapy in an NF-κB-dependent manner (Bhat et al., 2013). Macrophages are also an important source of TNF-α secretion.

### scRNA-seq and IHC Confirmation of the Negative Prognostic Effects of TAMs

To validate our findings, we collected scRNA-seq data for cell-component analysis. We classified all eight samples with available scRNA-seq data into negative or positive immune response clusters by calculating NES-based Spearman similarity between single-cell samples and bulk tumor samples (Supplementary Material 3). The results identified samples PJ016, PJ017, PJ032, and PJ048 as negative immune response and PJ018, PJ025, PJ032, and PJ035 as positive immune response.

We applied Seurat and copy number variation analyses to distinguish non-transformed cells from malignant transformed glioma cells in the scRNA-seq data. All HGGs, except PJ016, harbored clear amplification of chromosome 7 and loss of chromosome 10 (Supplementary Figures 3A–H), consistent with transformed tissues demonstrating large-scale copy number alterations and aneuploidies (Venteicher et al., 2017; Taylor et al., 2018), as well as glioblastoma often being accompanied with amplification of chromosome 7 and loss of chromosome 10 (Zagzag et al., 2005). PJ016 was found apparent loss of chromosomes 13 and 19, revealing that the cell population had indeed undergone transformation (Lee et al., 1995; Ritland et al., 1995; Nakamura et al., 2000).

**Figure 5:**
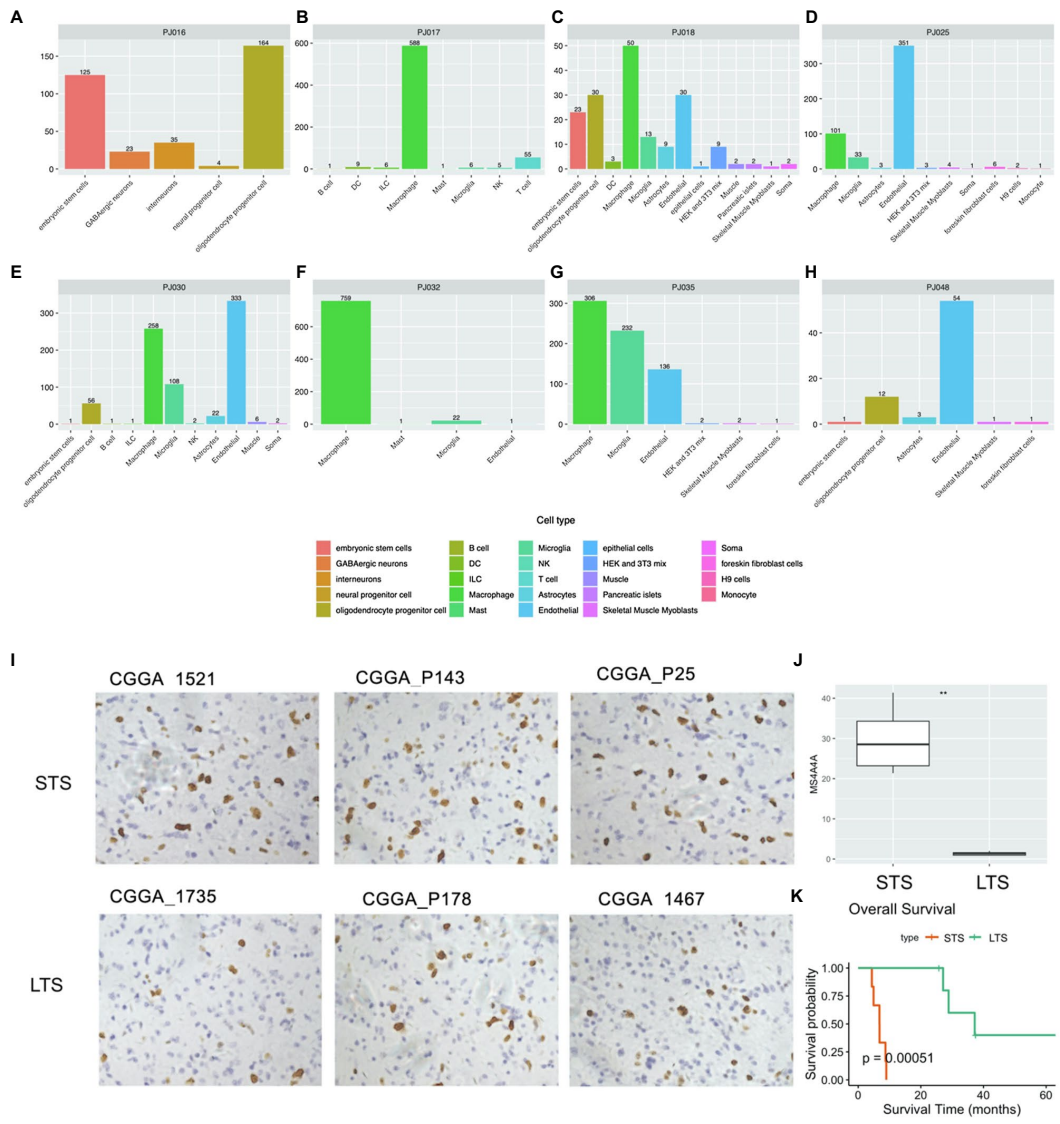
Cell type analysis using scRNA-seq data. **(A–H)** Cell type counts in scRNA-seq samples. **(I)** IHC staining of macrophages (NIR samples, upper; PIR samples, bottom). **(J)** Percentage of macrophages in NIR and PIR samples (according to staining for MS4A4A; scale bar: 100μm). **(K)** Kaplan–Meier survival curves of NIR and PIR samples. IHC, immunohistochemical; LTS, long-term survival; NIR, negative immune response; PIR, positive immune response; scRNA, single-cell RNA; and STS, short-term survival.

The identities of non-transformed cells in the glioma microenvironment were then determined using Scibet (Li et al., 2020; Figures 5A–H). We found no immune cells in PJ016 or PJ048 (Table 2), possibly due to the heterogeneity of different sampling areas. Those with a high percentage of macrophages (PJ017 and PJ032; 46.63 and 55.12%, respectively) belonged to the negative immune response cluster (Table 2), whereas samples with fewer macrophages (PJ018, PJ025, and PJ035; 2.28, 1.70, and 8.12%, respectively) overlapped with the positive immune response cluster (Table 2), confirming macrophage enrichment as a risk factor.

**Table 2:** Summary of scRNA-seq analysis.

Patient	Age	Sex	Diagnosis	Macrophage	Microglial	All	Macrophage: All (%)	Macrophage: Microglia	Cluster
PJ016	49	F	Glioblastoma, WHO grade IV	0	0	3,085	0	—	NIR
PJ017	62	M	Glioblastoma, WHO grade IV	588	6	1,261	46.63	98	NIR
PJ018	65	M	Glioblastoma, WHO grade IV	50	13	2,197	2.28	3.85	PIR
PJ025	74	M	Glioblastoma, WHO grade IV	101	33	5,924	1.70	3.06	PIR
PJ030	56	F	Anaplastic astrocytoma, WHO grade III	258	108	3,097	8.33	2.39	PIR
PJ032	63	F	Glioblastoma, recurrent	759	22	1,377	55.12	34.5	NIR
PJ035	50	M	Glioblastoma, recurrent	306	232	3,768	8.12	1.32	PIR
PJ048	59	M	Glioblastoma, WHO grade IV	0	0	3,084	0	—	NIR

NIR, negative immune response; PIR, positive immune response; scRNA, single-cell RNA; and WHO, World Health Organization.

Moreover, we confirmed the negative prognosis associated with macrophages IHC staining for the macrophage marker MS4A4A in 12 glioblastoma samples, including six from short-term-survival and six from long-term-survival patients (Figure 5K; Supplementary Material 4). The short-term-survival samples showed a significantly higher percentage of MS4A4A-positive cells relative to the six long-term-survival samples (*p*=0.00051; Figures 5I,J).

## Discussion

In this study, we generated a landscape of glioblastoma niches using four gene expression profiling cohorts of tumor tissues from GBMs based on the NES method. The patients in each cohort were divided into two categories (positive or negative immune response) according to hierarchical clustering analysis of cell type-based enrichment status and showing a significantly different survival (*p*<0.05). The analysis revealed risk factors, including astrocytes, macrophages, monocytes, NKTs, and MSC, as well as protective factors, CD8 T cells, CD8 naive T cells, and plasma cells. Additionally, GSEA demonstrated that immune system- and organogenesis-related GO terms were uniformly enriched in negative immune response clusters, whereas positive immune response clusters were enriched in the nervous system. Moreover, significant signs of mesenchymal differentiation were observed in the negative immune response clusters, and validation using scRNA-seq analysis and IHC staining showed correlations between the presence of macrophages and negative immune response.

Potential mechanisms associated with specific cell types manifested consistent risk levels. Some cell types exhibited identical risk levels across the four cohorts and all gene expression signatures. Specifically, astrocytes were frequently observed as a consistent risk factor with a high hazard ratio. As an important component of the blood–brain barrier and the tripartite synaptic neural network, the normal physiological role of astrocytes involves promoting mutual communication with neurons. However, astrocytes can also develop into tumor cells and form astrocytomas. Given the heterogeneity of gliomas, the high frequency of astrocytes as a risk factor is explainable. Moreover, evidence suggests that tumor-reactive astrocytes can interact with glioma tumor cells and promote the development, invasion, and survival of gliomas by releasing different cytokines or regulating the entry and exit of calcium and hydrogen ions in cell channels (Guan et al., 2018).

NKTs were also a consistent risk factor. miR-92a was reported to induce immune tolerance of NKTs to glioma cells (Tang et al., 2014). Co-culture of glioma cells and NKTs showed miR-92a expressing in glioma cells played a key role in inducing the elevated expression of IL-6 and IL-10 in NKTs (Tang et al., 2014). In the present study, we found IL-6- and IL-10-related GO terms in the negative immune response cluster. Compared with NKTs cultured alone, the expression of antitumor molecules, including perforin, Fas ligand, and IFN-γ, was significantly reduced in NKTs co-cultured with glioma cells (Tang et al., 2014). Moreover, IL-6+IL-10+ NKTs exhibit a weak ability to induce apoptosis in glioma cells but have an immunosuppressive effect on CD8 T cell activity (Tang et al., 2014).

CD8 T cells play defensive roles against cancer cells, consistent with the risk levels generated in the present analysis. Serologic analysis of antigens using recombinant cDNA expression cloning identified several tumor-associated antigens capable of generating a specific response in a variety of human cancers, including malignant glioma (Struss et al., 2001; Prins et al., 2003). Tumor-related antigens can be recognized by cytotoxic CD8 T cells in the context of tumors expressing MHC-I (Prins and Liau, 2003; Yang et al., 2004), suggesting that a T cell-dependent immune response might improve the outcome of glioma patients through an antigen-mediated immune response. This was supported by a clinical study of newly diagnosed glioblastoma patients that reported significantly attenuated CD8 T cell infiltration in samples from long-survival patients (>403days) relative to that in samples from short-survival patients (<95days; Yang et al., 2010). These findings agreed with those of the present study showing that CD8 T cells were categorized as a protective factor.

Some cell types exhibited inconsistent risk levels. In these cases, it is likely that other conditions caused a shift in risk levels (e.g., age, co-existence with other cells, or a combination of other clinical symptoms). Different signatures of the same cell type might display different risk levels, suggesting the impact of cell status. To further investigate this concept, a specific gene in each gene signature should be investigated. Other conditions, such as the presence of neoantigens (Zhang et al., 2019), IDH mutation(s) (Phillips et al., 2006; Parsons et al., 2008), and *MGMT* methylation (Shah et al., 2011), can also provide insight into conditions causing a shift in risk levels. Furthermore, the data used in this study were from primary gliomas; therefore, comparisons between recurrent and primary glioma samples would provide additional information concerning dynamics in the glioma microenvironment.

Myeloid lineage cells, such as monocytes and macrophages, were consistent risk factors in agreement with previously reported results (Hambardzumyan et al., 2016). These cells (i.e., TAMs) account for more than 30% of the total number of solid tumor cells (Boussiotis and Charest, 2018, 1–3). Numerous studies report that the frequency of TAM detection is usually higher in tumors with a mesenchymal subtype and/or recurrent tumors (Wang et al., 2017). Glioma stem cells are recently shown to release periostin, which accumulates in the surrounding environment of blood vessels and acts as an inducer of TAM chemotaxis through signaling *via* the integrin receptor αvβ3 (Zhou et al., 2015). Transforming growth factor (TGF)-β released by TAMs induces matrix metalloprotein-9 expression in glioblastoma stem cells, thereby increasing their invasiveness (Ye et al., 2012). Furthermore, the supernatant from glioma stem cells (GSCs) inhibits the phagocytic activity of TAMs and induces IL-10 and TGF-β secretion (Wu et al., 2010).

Ontogeny analysis revealed that macrophages in human GBM can be divided into either blood-derived or tissue-resident variants (i.e., microglia; Wang et al., 2017). These two ontogenies were also found in other types of cancer and displayed different prognostic effects. In mouse mammary carcinoma, a distinction was made between monocyte-derived TAMs and resident mammary tissue macrophages; it was found that only the former contributes to the suppression of antitumor cytotoxic T cell responses (Franklin et al., 2014; Pombo Antunes et al., 2020). Normal naïve microglial cells can reduce the ability of human stem cells to acquire a spheroid morphology, thereby adversely affecting GSCs and inhibiting the growth of gliomas. However, another study suggested that microglial cells or monocytes derived from gliomas lack such antitumor potential (Sarkar et al., 2013). scRNA-seq analysis of human gliomas showed that blood-derived TAMs upregulate immunosuppressive cytokines and demonstrate an altered metabolism relative to microglial TAMs and that the gene signature of blood-derived TAMs but not microglial TAMs correlates with significantly inferior survival in low-grade glioma (Wu et al., 2010). Signatures of microglial TAMs were not included among the curated markers used for tumor tissue analysis; however, scRNA-seq analysis showed that negative immune response samples comprised a significantly higher macrophage: microglia ratio than positive immune response samples (98 vs. 34.5, respectively; Table 2).

## Conclusion

We present a comprehensive characterization of non-tumor cells in the niches of primary glioblastoma by integrating four large cohorts of GBM gene expression data and 540 gene signatures covering 64 non-tumor cells types. We find that non-tumor cell type enrichment status is useful for stratifying glioblastomas into different prognostic groups (positive or negative immune response clusters). The negative immune response clusters are uniformly enriched with immune system- and organogenesis-related GO terms, whereas positive immune response clusters are enriched with the nervous system. The mesenchymal differentiation is also observed in the negative immune response clusters. Moreover, risk analysis using cell components to determine glioma niches helps interpret the impact of cell type on cancer prognosis. Astrocytes, macrophages, monocytes, NKTs, and MSC are found as risk factors, and CD8 T cells, CD8 naive T cells, and plasma cells are protective factors. Particularly, the high presence of macrophages in the negative immune response clusters is validated using scRNA-seq analysis and IHC staining of GBMs from independent cohorts. Future investigations should focus on cell types with variable risk levels in order to elucidate the potential mechanisms involved in shifts in prognostic effects. Other stratification methods should be established and evaluated for categorizing samples individually rather than as groups.

## Data Availability Statement

This data can be found at: The data that support the findings of this study are openly available. The availability of download URL and clinical information for the four datasets was indicated in the original researches including Cohort 1 (Wang et al., 2016; Zhang et al., 2019), Cohort 2 (TCGA; RNA sequences; Cancer Genome Atlas Research, 2008), Cohort 3 (REMBRANDT, mRNA microarray; Gusev et al., 2018), and Cohort 4 (TCGA, mRNA microarray; Brennan et al., 2013). The single-cell RNAseq data of eight HGGs can be accessed through Gene Expression Omnibus (accession: GSE103224; Yuan et al., 2018). All the raw data and original images of IHC were also deposited at github.^2^ The code for calculating clustering and survival analysis, cox regression analysis, NES score, and NES distribution, was deposited at github.^3^

## Ethics Statement

The studies involving human participants were reviewed and approved by the Beijing Tiantan Hospital institutional review board. The patients/participants provided their written informed consent to participate in this study.

## Author Contributions

WZ, YF, and JZ conceived and supervised the study. LL, TS, YH, GL, LW, XH, HW, YL, YC, HYW, and JL curated the data. ZX, GZL, and WL performed the analysis. ZX, GZL, and JZ investigated the results. WZ, GZL, and WL performed the validation experiments. ZX and WL conducted the visualization. ZX, WZ, YF, and JZ wrote the manuscript. All authors contributed to the article and approved the submitted version.

## Funding

This work was supported by grants from the Youth Thousand Scholar Program of China (JZ), Program for High-Level Overseas Talents, Beihang University (JZ), Outstanding and innovative program in medicine and engineering, Beihang University (JZ), National Natural Science Foundation of China (no. 81672479 to WZ, 11421202, and 11827803 to YBF), National Natural Science Foundation of China (NSFC)/Research Grants Council (RGC) Joint Research Scheme (81761168038; WZ), and Beijing Municipal Administration of Hospitals’ Mission Plan (SML20180501; WZ).

## Conflict of Interest

The authors declare that the research was conducted in the absence of any commercial or financial relationships that could be construed as a potential conflict of interest.

## Publisher’s Note

All claims expressed in this article are solely those of the authors and do not necessarily represent those of their affiliated organizations, or those of the publisher, the editors and the reviewers. Any product that may be evaluated in this article, or claim that may be made by its manufacturer, is not guaranteed or endorsed by the publisher.

## Footnotes

^1^ http://scibet.cancer-pku.cn/download_references.html

^2^ https://github.com/zhangjbig/xzx/tree/main/Data

^3^ https://github.com/zhangjbig/xzx
