# Supplementary material for: Multiomics Analysis Reveals the Prognostic Non-tumor Cell Landscape in Glioblastoma Niches
Source: Front Genet. 2021 Sep 16;12:741325. doi: 10.3389/fgene.2021.741325 (PMC8481948; doi:10.3389/fgene.2021.741325)
Supplement: Supplementary file 1 [file Data_Sheet_1.PDF]

Description of supplementary materials

Supplementary Figures:

Figure S1. NES distribution and NES heatmap of gene signatures generated from hierarchical clustering.

Figure S2. GO enrichment in clusters and NES distribution in NIR and PIR of gene signatures for

Endothelial cells in four cohorts.

Figure S3. Separation of malignant (transformed) cells from non-malignant (non-transformed) cells based on amplification of chromosome 7 and loss of chromosome 10 for eight high grade gliomas.

Supplementary Data

Supplementary Data 1A. Summary and categorization of 64 cell types used in this analysis.

Supplementary Data 1B. A full list of 540 gene signatures for a total of 64 cells types.

Supplementary Data 2A. The grouping of GO terms in Cohort 1.

Supplementary Data 2B. The grouping of GO terms in Cohort 2.

Supplementary Data 2C. The grouping of GO terms in Cohort 3.

Supplementary Data 2D. The grouping of GO terms in Cohort 4.

Supplementary Data 3. Classification of all eight samples with scRNA-seq data available into NIR or PIR clusters.

Supplementary Data 4. Clinical information and IHC staning results for 12 patients.

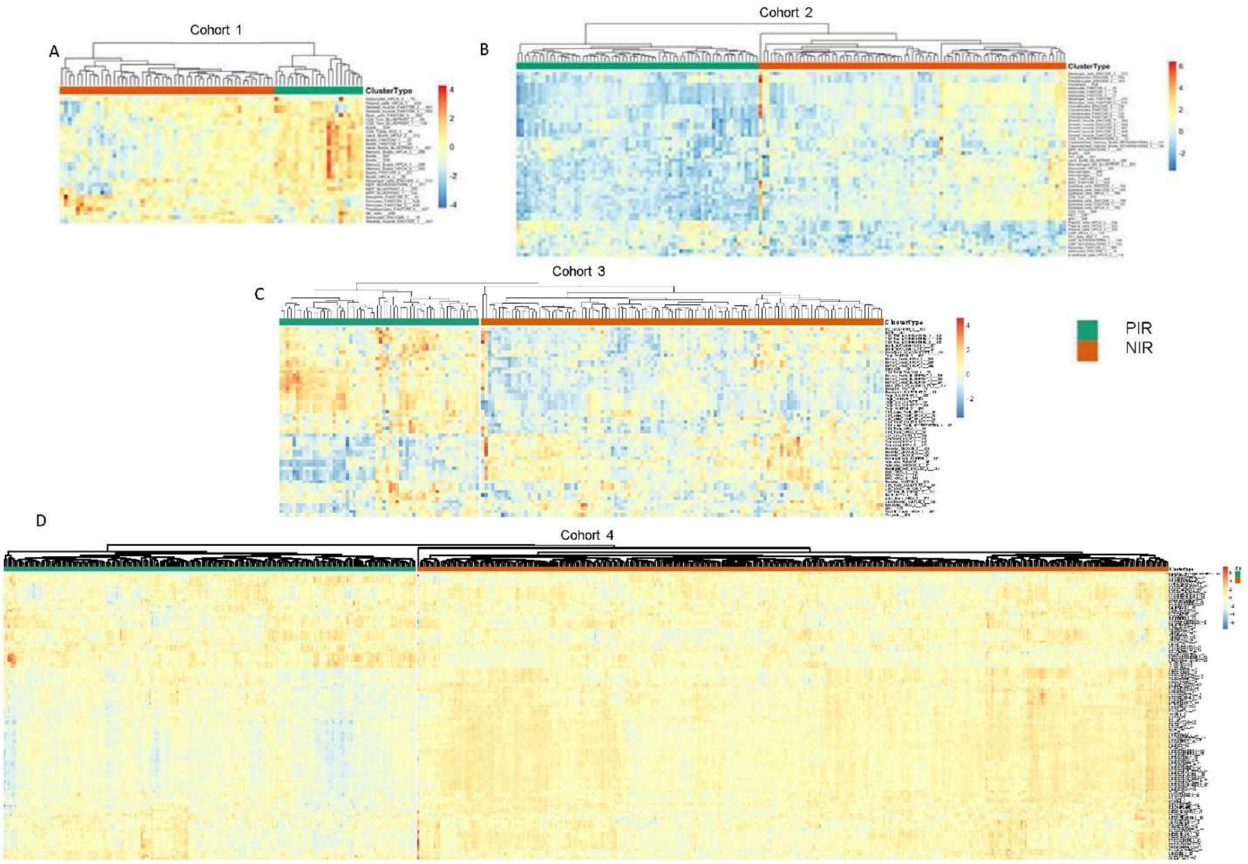

Figure S1. NES heatmap of gene signatures generated from hierarchical clustering (A) Cohort 1. (B) Cohort 2. (C) Cohort 3. (D) Cohort 4. (Green: samples of PIR, orange: samples of NIR).

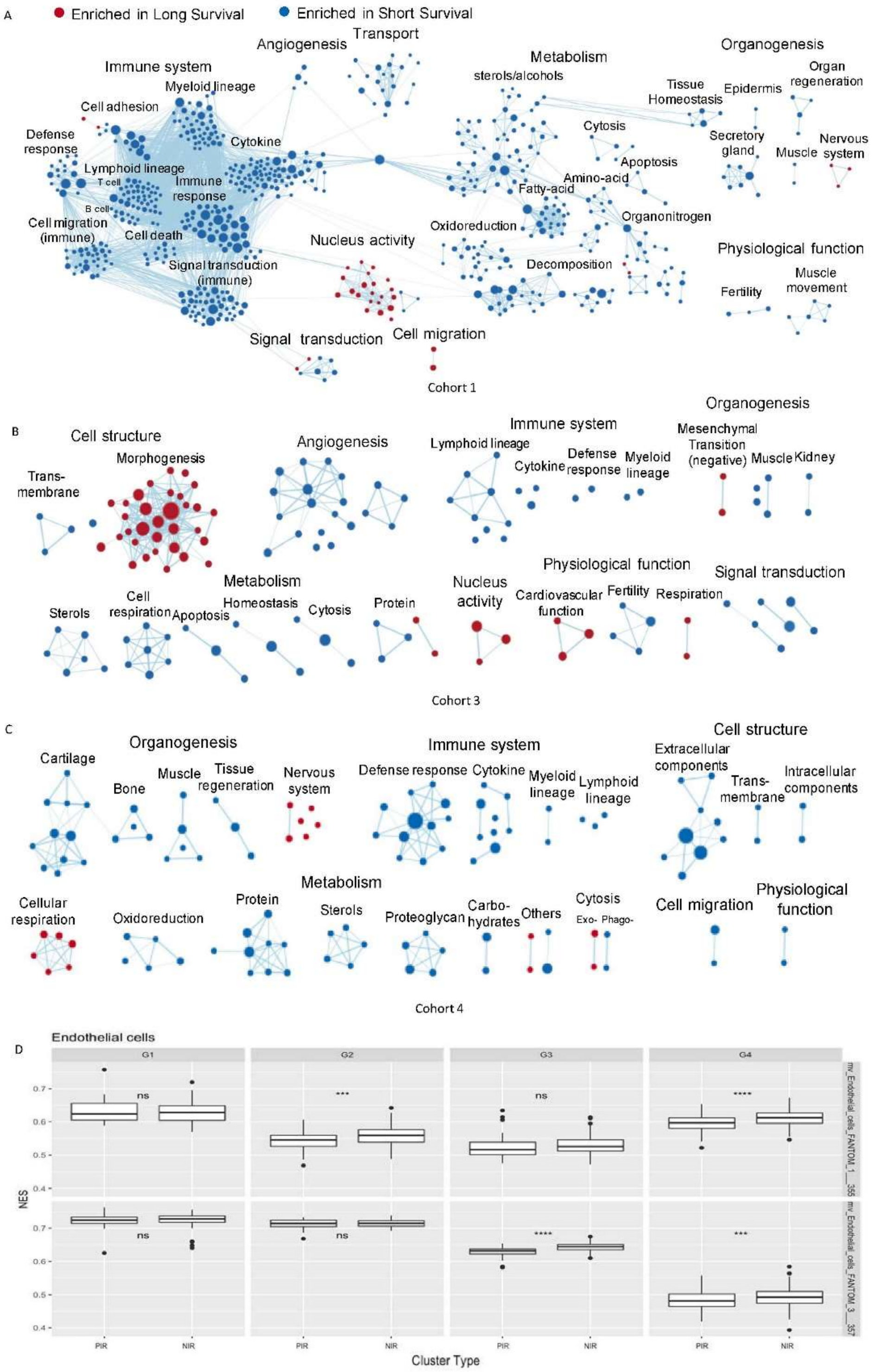

Figure S2. GO enrichment in clusters and NES distribution in NIR and PIR of gene signatures for Skeletal muscle and Endothelial cells in four cohorts. Enrichment map of GO terms (selected according to p < 0.05) aggregrated by functions for cohorts (A) 1, (B) 3, and (C) 4. GO, Genen Ontology. (D) endothelial cells. (ns: p > 0.05; *: p < 0.05; **: p < 0.01; ***: p < 0.001; ****: p < 0001)

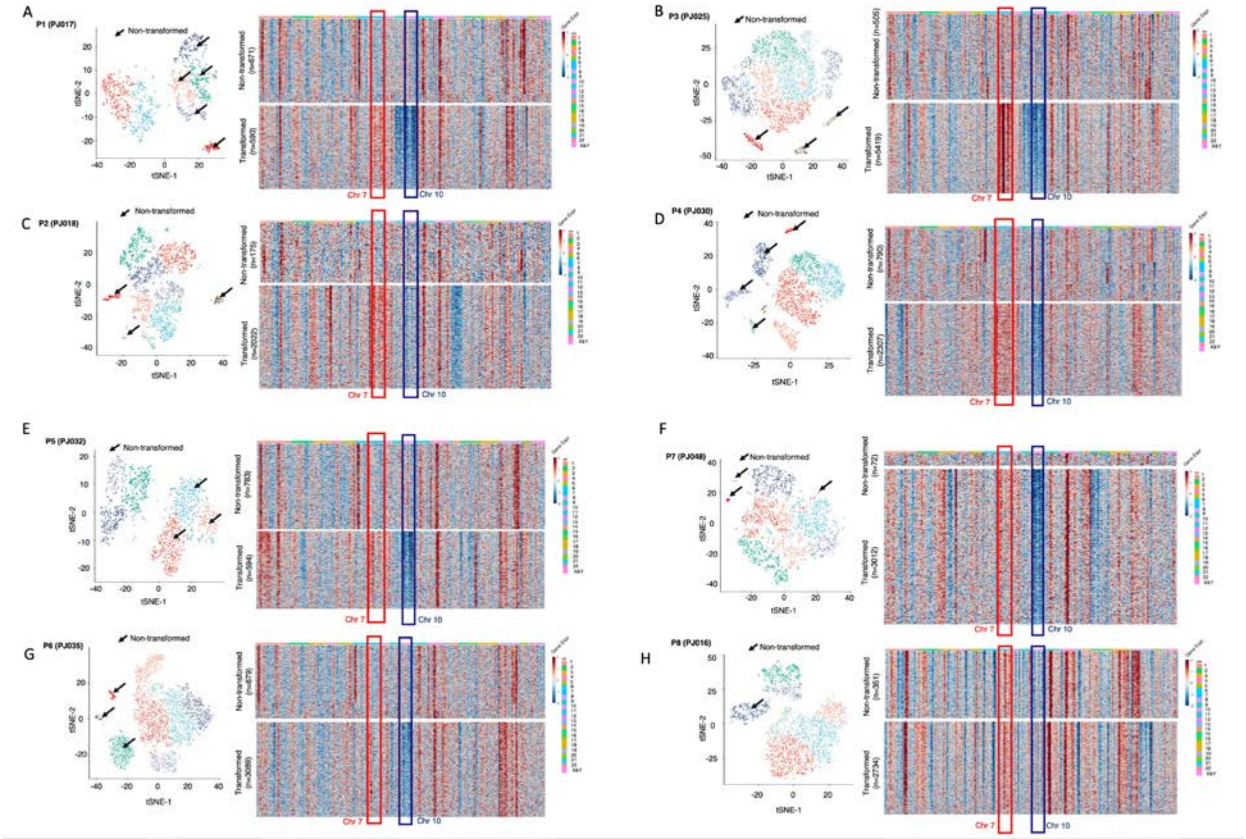

Figure S3. Separation of malignant (transformed) cells from non-malignant (non-transformed) cells based on amplification of chromosome 7 and loss of chromosome 10 for eight high grade gliomas including (A) PJ017, (B) PJ025, (C) PJ018, (D) PJ030, (E) PJ032, (F) PJ048, (G) PJ035 and (H) PJ016.

Supplementary Data 1A. Summary and categorization of 64 cell types used in this analysis

Full name	Cell types	Number of Signtures	Subgroup
Epithelial cells	Epithelial cells	9	
Keratinocytes	Keratinocytes	9	
Melanocytes	Melanocytes	7	
Astrocytes	Astrocytes	6	
Neurons	Neurons	6	
Hepatocytes	Hepatocytes	6	
Sebocytes	Sebocytes	3	Others
Erythrocytes	Erythrocytes	12	
Multipotent progenitors	MPP	12	
Common myeloid progenitors	CMP	9	
Granulocyte-macrophage progenitors	GMP	9	
Megakaryocyte–erythroid progenitors	MEP	9	
Hematopoietic stem cells	HSC	9	
Megakaryocytes	Megakaryocytes	6	
Common lymphoid progenitors	CLP	3	
Platelets	Platelets	3	Hematopoietic stem cells and hematopoietic cells
CD8+ T-cells	CD8+ T-cells	18	Lymphoid
NK cells	NK cells	18	lineage
CD4+ naive T-cells	CD4+ naive T-cells	12	
B-cells	B-cells	13	
CD4+ T-cells	CD4+ T-cells	11	
CD8+ effector memory T-cells	CD8+ Tem	10	
Regulatory T-cells	Tregs	11	
Plasma cells	Plasma cells	9	
CD4+ central memory T-cells	CD4+ Tcm	11	
CD4+ effector memory T-cells	CD4+ Tem	11	
Memory B-cells	Memory B-cells	10	
CD8+ central memory T-cells	CD8+ Tcm	10	
naive B-cells	naive B-cells	9	
CD4+ memory T-cells	CD4+ memory T-cells	6	
pro B-cells	pro B-cells	6	
Class-switched memory B-cells	Class-switched memory B-cells	6	
Type 2 T-helper cells	Th2 cells	5	
Type 1 T-helper cells	Th1 cells	5	
CD8+ naive T-cells	CD8+ naive T-cells	3	
Natural killer T-cells	NKT	4	
Gamma delta T-cells	Tgd cells	5	
Monocytes	Monocytes	16	Myeloid
Macrophages	Macrophages	16	lineage
Dendritic cells	DC	14	
Neutrophils	Neutrophils	15	
Eosinophils	Eosinophils	11	
Macrophages M1	Macrophages M1	6	
Macrophages M2	Macrophages M2	6	
Activated dendritic cells	aDC	8	
Basophils	Basophils	6	
Conventional dendritic cells	cDC	6	
Plasmacytoid dendritic cells	pDC	8	
Immature dendritic cells	iDC	5	
Mast cells	Mast cells	5	
Endothelial cells	Endothelial cells	13	
Smooth muscle cells	Smooth muscle	9	
Fibroblasts	Fibroblasts	10	
Chondrocytes	Chondrocytes	9	
Adipocytes	Adipocytes	9	
Microvascular endothelial cells	mv Endothelial cells	9	
Myocytes	Myocytes	6	
Lymphatic endothelial cells	ly Endothelial cells	6	
Mesenchymal stem cells	MSC	6	
Osteoblasts	Osteoblast	6	
Preadipocytes	Preadipocytes	6	
Skeletal muscle cells	Skeletal muscle	6	
Pericytes	Pericytes	6	
Mesangial cells	Mesangial cells	6	Stromal cells
**SUM**	**64**	**540**	**5**

Supplementary Data 1B. A full list of 540 gene signatures for a total of 64 cells types.

aDC_HPCA_1___1	Aran_et_al_2017	C1QA, C1QB, CD80, IL12B, CCL13, CCL17, CCL19, CCL22
aDC_HPCA_2___2	Aran_et_al_2017	C1QA, C1QB, CD80, FPR3, HLA-DQA1, IL12B, CCL13, CCL17, CCL19, CCL22
aDC_HPCA_3___3	Aran_et_al_2017	C1QA, C1QB, CD80, FPR3, HLA-DQA1, IL12B, CCL13, CCL17, CCL19, CCL22
aDC_IRIS_1___4	Aran_et_al_2017	CD80, IL3RA, IL12B, CXCL9, PTGIR, CCL8, CCL13, CCL17, CCL19, CCL23, SLAMF1, SIGLEC1, TRAF1, TNFRSF4, TXN, SOCS3, HS3ST3B1, CD209, LILRA5
aDC_IRIS_2___5	Aran_et_al_2017	ACHE, ADPRH, ALOX15B, ANXA5, XIAP, ARF3, RHOG, ATP1B3, BLVRA, C1QA, C1QB, C3AR1, CASP5, CD80, CD86, CCR5, CMKLR1, EIF5, ENO1, ETV3, FCER1G, FCER2, FPR3, GMFB, GNG5, GRB2, RAPGEF1, HCK, HLA-DQA1, HRH2, DNAJA1, IL2RA, IL3RA, IL9, IL10, IL10RA, IL12B, IMPDH1, IRF4, KCNMB1, LAIR1, LOR, RAB8A, CXCL9, MTF1, NFE2L2, NFKB1, NFKBIB, NRAS, OSM, P2RX7, PAK2, PGK1, PITPNA, MAP2K1, PTGIR, RAB5A, RELA, CLIP1, S100A10, CCL1, CCL4, CCL7, CCL8, CCL17, CCL18, CCL19, CCL22, CCL23, CCL24, SLAMF1, SLC1A2, SLC6A12, SIGLEC1, SRC, STAT2, TCF21, DYNLT1, TPI1, TRAF1, TNFRSF4, TXN, VRK2, XPNPEP1, ZBTB17, CUL1, RRP1, SCARF1, ALDH1A2, TAX1BP1, SOCS3, SPAG9, TMSB10, MAP3K13, TRIP4, H6PD, WTAP, ARHGEF11, TMCC2, HS3ST3B1, ABI1, BCL2L11, ARPC4, ARFRP1, RAMP3, LILRB2, BCKDK, CXCL13, EXOC5, MTHFD2, SEC24A, NEU3, LILRB1, CD300C, LILRB5, RAB35, OGFR, HPS5, TREX1, TFEC, RAB21, KDM6B, FAM175B, MGRN1, TDRD7, ABCA6, DNPEP, ACOT9, NUP62, BCL2L13, FBXL4, SIGLEC7, CYTH4, SIGLEC9, TOR1B, GPR132, IL19, CD209, ANKFY1, AZIN1, RIN2, TBC1D13, GPN2, C1orf27, ARL8B, ZNF654, TBC1D22B, MFN1, SCYL2, CHFR, KCNK13, C5orf15, RPGRIP1, CAMK1G, DENND1A, GPR107, HAMP, MIIP, NSUN3, MMP25, UBE2Z, TNIP2, OPA3, TMX1, SLCO5A1, NETO2, MED25, SLC25A28, ADPGK, MAGT1, DOT1L, LILRA5, TMEM41B
aDC_IRIS_3___6	Aran_et_al_2017	ACHE, ALOX15B, RHOG, ATP1B3, BLVRA, C1QB, CD80, CD86, EIF5, ENO1, ETV3, GNG5, RAPGEF1, HCK, IL2RA, IL3RA, IL9, IL10, IL10RA, IL12B, IRF4, RAB8A, CXCL9, MTF1, NFE2L2, NFKB1, P2RX7, PGK1, MAP2K1, PTGIR, RELA, CLIP1, CCL7, CCL8, CCL17, CCL19, CCL23, SLAMF1, SLC1A2, SLC6A12, SIGLEC1, SRC, STAT2, TRAF1, TNFRSF4, TXN, ZBTB17, ELL, CUL1, ALDH1A2, SOCS3, SPAG9, TMSB10, MAP3K13, WTAP, N4BP1, ARHGEF11, HS3ST3B1, ABI1, ARPC4, MTHFD2, SEC24A, NEU3, OGFR, TFEC, FAM175B, TDRD7, ACOT9, NUP62, ABTB2, TRPC4AP, FBXL4, CYTH4, TOR1B, SNX11, GPR132, IL19, CD209, CHMP5, AZIN1, C1orf27, ARL8B, ZNF654, MFN1, NECAP2, CHFR, C5orf15, CAMK1G, DENND1A, GPR107, MIIP, NSUN3, UBE2Z, TNIP2, SLCO5A1, ADPGK, DOT1L, LILRA5
Adipocytes_ENCODE_1___7	Aran_et_al_2017	ADH1B, DLAT, GPD1, LBP, PLIN1, PPP1R1A, PPP2R1B, PTGER3, CILP, ADIPOQ, COL5A3, PNPLA2
Adipocytes_ENCODE_2___8	Aran_et_al_2017	ADH1B, ATP5G3, DLAT, GPD1, HADHA, LBP, PLIN1, PPP1R1A, PPP2R1B, PTGER3, CILP, ADIPOQ, COL5A3, PNPLA2
Adipocytes_ENCODE_3___9	Aran_et_al_2017	ADH1B, ATP5G3, DBI, DLAT, GPD1, LBP, PLIN1, PPP2R1B, PTGER3, CILP, ADIPOQ, COL5A3, PNPLA2
Adipocytes_FANTOM_1___10	Aran_et_al_2017	ADH1B, ATP1A2, GPD1, HP, LBP, PLIN1, TF, ADIPOQ
Adipocytes_FANTOM_2___11	Aran_et_al_2017	ADH1B, ATP1A2, GPD1, HP, LBP, PLIN1, TF, ADIPOQ
Adipocytes_FANTOM_3___12	Aran_et_al_2017	ADH1B, ATP1A2, C6, GPD1, HP, LBP, PLIN1, TF, ADIPOQ
Adipocytes_HPCA_1___13	Aran_et_al_2017	ADH1B, ATP5G3, DLAT, GPD1, HADHA, LBP, PLIN1, PPP1R1A, PPP2R1B, PTGER3, CILP, ADIPOQ, COL5A3, PNPLA2
Adipocytes_HPCA_2___14	Aran_et_al_2017	ADH1B, DLAT, GPD1, LBP, PLIN1, PPP1R1A, PPP2R1B, PTGER3, CILP, ADIPOQ, COL5A3, PNPLA2
Adipocytes_HPCA_3___15	Aran_et_al_2017	ADH1B, SLC25A6, ATP5G3, DBI, DLAT, GPD1, LBP, PLIN1, PPP1R1A, PPP2R1B, PTGER3, CILP, ADIPOQ, COL5A3, ECHDC1, PNPLA2
Astrocytes_ENCODE_1___16	Aran_et_al_2017	CNN1, COL11A1, DNA2, FABP7, GPC4, CENPI, FZD2, GLI2, GNG3, GPX7, TNC, LOXL1, LRP4, NNAT, POU3F3, PTN, PTX3, RBP1, SDC2, TFAP2C, TRO, XRCC2, ADAM12, DPF3, ST8SIA2, ZNF239, DCHS1, KIF20B, REC8, PNMA2, CAND2, FBXL7, SYT11, DAPK2, ANKRD1, NT5DC3, PNMAL1, ASAP3, DOK5, GABRQ, AGPAT4, NCAPG, FSD1, KIF18A, MXD3, PSRC1, OLFML2A
Astrocytes_ENCODE_2___17	Aran_et_al_2017	CBR3, CLCN2, CNN1, COL11A1, FABP7, GPC4, FZD2, GRM7, HIST1H1D, TNC, KIFC1, LOXL1, MCM3, POU3F3, PTN, SCN5A, SDC2, STAC, TFAP2C, TRO, XRCC2,

		ADAM12, DPF3, ST8SIA2, ZNF239, DCHS1, RECQL4, REC8, TRIOBP, CAND2, FBXL7, DAPK2, RIBC2, FAM64A, PNMAL1, ASAP3, DOK5, GABRQ, AGPAT4, NT5DC2, TMEM135, FSD1, ACSS3, ATF7IP2, TRIM45, MXD3, GINS4, PSRC1, CBX2, H2AFV, OLFML2A
Astrocytes_ENCODE_3___18	Aran_et_al_2017	CNN1, COL11A1, FABP7, FZD2, GNG3, TNC, LRP4, POU3F3, PTN, ADAM12, DPF3, DAPK2, ANKRD1, PNMAL1, ASAP3, DOK5, GABRQ, AGPAT4, PSRC1, OLFML2A
Astrocytes_FANTOM_1___19	Aran_et_al_2017	ACTA2, ACTG2, APLP1, BGN, BST1, C1S, SERPINH1, CDH6, CDH11, CHN1, CLU, CNN1, COL1A1, COL1A2, COL3A1, COL5A1, COL5A2, COL6A1, COL6A2, COL6A3, COL8A2, COL13A1, CRABP2, VCAN, DCN, DIO2, DPYSL3, DUSP2, FAP, FBLN2, FBN1, FGFR1, FGG, FLNC, FN1, FZD2, GATA6, GBP2, GEM, GNG11, CFH, HOXB2, CFI, IGFBP2, IGFBP3, IGFBP5, IGFBP6, ITGA7, AFF3, LAMA4, LIF, LOX, LOXL1, LUM, MAP1A, MATN2, MDK, MEST, MFAP4, MGP, MMP2, MN1, MYLK, NAP1L3, CLDN11, OXTR, PALM, PAX6, PCOLCE, PDGFRA, PDGFRB, SERPINF1, PLN, PPP1R3C, PTGIS, PTN, PTPRN, RARB, RARRES1, RARRES2, RGS4, CCL2, CXCL12, SFRP4, SLC14A1, SPARC, TAGLN, NR2F1, TFPI, TGM2, THY1, TIMP1, TIMP2, TNS1, TPM1, TPM2, TNFSF4, UCHL1, VCAM1, WNT5A, ADAM12, MFAP5, FZD7, RGS5, PDE5A, ADAM19, CACNA1H, DIRAS3, CRLF1, DHRS3, ADAMTS2, SEMA3E, HEPH, MRC2, GFPT2, HS3ST3A1, EDIL3, LRRC17, BTN3A3, MYL9, SPON2, POSTN, NES, GLIPR1, PADI2, FILIP1L, SYNPO, NID2, NFASC, SULF1, MXRA5, MOXD1, PCOLCE2, GREM1, ANKRD1, SRPX2, EFEMP2, CPA4, COPZ2, SLC22A17, DACT1, TRPV2, SCARA3, MXRA8, SYTL2, PNMAL1, MEG3, OLFML3, RCN3, FKBP10, CLSTN2, XYLT1, C9orf16, GLT8D2, SLC12A8, CREB3L1, CHRDL1, LYPD1, ZCCHC24
Astrocytes_FANTOM_2___20	Aran_et_al_2017	ACTA2, ACTG2, BGN, CNN1, COL1A1, COL1A2, COL3A1, COL5A1, COL6A1, COL6A2, COL8A2, VCAN, DIO2, DPYSL3, FBLN2, FGG, FLNC, FN1, CFH, HOXB2, CFI, IGFBP2, IGFBP3, IGFBP5, LAMA4, LOXL1, LUM, MEST, MFAP4, MGP, MYLK, OXTR, PCOLCE, PDGFRA, PDGFRB, PLN, PPP1R3C, PTGIS, PTN, PTPRN, RARRES1, RARRES2, RGS4, CCL2, CXCL12, SFRP4, SLC14A1, SPARC, TAGLN, TGM2, THY1, TPM1, TPM2, TNFSF4, VCAM1, ADAM12, MFAP5, FZD7, RGS5, ADAM19, CACNA1H, DIRAS3, CRLF1, DHRS3, ADAMTS2, SEMA3E, GFPT2, LRRC17, MYL9, POSTN, GLIPR1, PADI2, NID2, SULF1, MXRA5, GREM1, ANKRD1, EFEMP2, CPA4, COPZ2, DACT1, MXRA8, OLFML3, RCN3, XYLT1, SLC12A8
Astrocytes_FANTOM_3___21	Aran_et_al_2017	ACTA2, ACTG2, APLP1, BGN, C1S, CDH11, CLU, CNN1, COL1A1, COL1A2, COL3A1, COL5A1, COL5A2, COL6A1, COL6A2, COL6A3, COL8A2, COL13A1, VCAN, DCN, DIO2, DPYSL3, DUSP2, FAP, FBLN2, FBN1, FGG, FLNC, FN1, CFH, HOXB2, CFI, IGFBP2, IGFBP3, IGFBP5, IGFBP6, LAMA4, LOX, LOXL1, LUM, MAP1A, MDK, MEST, MFAP4, MGP, MYLK, NAP1L3, CLDN11, OXTR, PCOLCE, PDGFRA, PDGFRB, PLN, PPP1R3C, PTGIS, PTN, PTPRN, RARRES1, RARRES2, RGS4, CCL2, CXCL12, SFRP4, SLC14A1, SPARC, TAGLN, NR2F1, TGM2, THY1, TPM1, TPM2, TNFSF4, VCAM1, ADAM12, MFAP5, FZD7, RGS5, PDE5A, ADAM19, CACNA1H, DIRAS3, CRLF1, DHRS3, ADAMTS2, SEMA3E, MRC2, GFPT2, HS3ST3A1, EDIL3, LRRC17, BTN3A3, MYL9, SPON2, POSTN, NES, GLIPR1, PADI2, FILIP1L, NID2, NFASC, SULF1, MXRA5, PCOLCE2, GREM1, ANKRD1, SRPX2, EFEMP2, CPA4, COPZ2, DACT1, SCARA3, MXRA8, OLFML3, RCN3, FKBP10, CLSTN2, XYLT1, GLT8D2, SLC12A8, CREB3L1, LYPD1
Bcells_FANTOM_1___22	Aran_et_al_2017	BLK, CD19, MS4A1, CD22, CD37, CD79A, PNOC, SNX2, MBD4, STAG3, PWP1, SP140, GGA2, STAP1, FCRL2, SMC6
Bcells_FANTOM_2___23	Aran_et_al_2017	BLK, CD19, CD22, CD37, CD79A, PNOC, MBD4, STAG3, SP140, GGA2, FCRL2, SMC6
Bcells_FANTOM_3___24	Aran_et_al_2017	BLK, BTK, CD19, MS4A1, CD22, CD37, CD79A, CSNK1G3, PHKB, PNOC, SNX2, TRAF3, MBD4, DEPDC5, STAG3, PWP1, SP140, GGA2, STAP1, FCRL2, SMC6
Bcells_HPCA_1___25	Aran_et_al_2017	BLK, BTK, CD19, MS4A1, CD22, CD37, CD79A, CSNK1G3, CD180, PHKB, PNOC, POU2F1, PRKCB, SNX2, TRAF3, UBE2G1, PRDM2, MBD4, SLC24A1, DEPDC5, BCL2L11, STAG3, PRDM4, PWP1, SP140, RRAS2, GGA2, SIPA1L3, STAP1, P2RY10, CDC40, MIOS, AFTPH, DEF8, ARHGAP17, MFN1, WDR11, FCRL2, SMC6, C12orf49, PIKFYVE, JMJD1C, MCM9, EGOT
Bcells_HPCA_2___26	Aran_et_al_2017	CD19, MS4A1, CD22, CD37, CD79A, SNX2, PRDM4, GGA2, FCRL2, SMC6
Bcells_HPCA_3___27	Aran_et_al_2017	BLK, CD19, HLA-DOA, SPIB, TNFRSF13B, STAP1, VPREB3, FCRL2
Bcells_NOVERSHTERN_1___28	Aran_et_al_2017	ACTN2, TNFRSF17, BLK, CXCR5, CD19, MS4A1, CD22, CD37, CD53, CD72, CD79A, CD79B, CNR1, CNR2, DAXX, DNASE1, AFF2, GDI2, GPR18, HLA-DOA, HTR3A, IFNW1, IL17A, INPP5B, KCNN3, CD180, MGAT5, PAX5, PGR, PNOC, POU2F2, SPIB, SYPL1, TERT, SEC62, SLC30A4, TCL1A, RNGTT, MBD4, S1PR2, RECQL5, LY86, MYOT, TCL1B, STAG3, LSM6, SP140, IKZF3, CNOT1, SIPA1L3, KIAA1033, TNFRSF13B, STAP1, TCL6, VPREB3, ITSN2, HDAC7, ARHGAP17, QRSL1, ATF7IP, UTP6, AICDA,

		DCLRE1C, FCRL2, PIKFYVE
Bcells_NOVERSHTERN_2___29	Aran_et_al_2017	TNFRSF17, BLK, CXCR5, BMP8B, CD19, MS4A1, CD37, CD53, CD72, CD79A, CD79B, CHAD, CCR6, COL19A1, CR1, CSNK1G3, GPR25, HLA-DOA, HLA-DPB1, HSPA4, HTR3A, LY9, CD180, MGAT5, CIITA, MAP3K9, MMP17, PAX5, RPS11, RPS16, SNX2, SPIB, TROVE2, ZNF37A, ZNF154, ZNF202, ZNF208, TCL1A, AP3B1, S1PR4, BAIAP3, LY86, TCL1B, KIAA0430, DEPDC5, CEPT1, SP140, IKZF3, GGA2, NUP160, KIAA1033, TNFRSF13B, ZZZ3, STAP1, PLA2G2D, ZNF638, P2RY10, VPREB3, KCNIP2, ANKMY1, TLR7, POLR3K, QRSL1, GPRC5D, NSUN5, WDR11, ZNF701, UTP6, C5orf15, DCLRE1C, FCRL2, RIC3, SMC6, MRM1, CEACAM21, ZNF688
Bcells_NOVERSHTERN_3___30	Aran_et_al_2017	ACTN2, TNFRSF17, BLK, CXCR5, CD19, MS4A1, CD22, CD37, CD53, CD72, CD79A, CD79B, CNR1, AFF2, GDI2, GPR18, HLA-DOA, HTR3A, IFNA2, IFNW1, IL17A, INPP5B, KCNN3, PAX5, PNOC, POU2F2, SPIB, SYPL1, TERT, SLC30A4, TCL1A, RNGTT, MBD4, SLC13A2, S1PR2, RECQL5, LY86, MYOT, TCL1B, STAG3, LSM6, SP140, IKZF3, CNOT1, STAP1, TCL6, VPREB3, ITSN2, QRSL1, ATF7IP, UTP6, AICDA, DCLRE1C, FCRL2, PIKFYVE
Basophils_FANTOM_1___31	Aran_et_al_2017	ALAS2, ARR3, ART3, C8A, CD72, DEFA5, EFNA2, GPR3, ONECUT1, HP, CXCR2, KCNA5, KCNJ13, LECT2, LEP, SMCP, MPO, MYH1, NPY5R, OMG, PRKACG, PRL, TGM3, TCL1A, AKAP4, C6orf10, FRS3, LAMB4, CLCA4, AHCTF1, BMP10, AMOTL2, ARHGAP17, TEX12, C11orf16, OTUD7B, SLC17A6, CASS4, LRTM1, HPSE2, FBRS, CXorf36, SLCO5A1, ATXN3L, GATC
Basophils_FANTOM_2___32	Aran_et_al_2017	ACTN2, ADCY2, ADH1A, ADPRH, ADRA1A, AGTR2, ALDOB, APOF, ARR3, ART1, ART3, BRDT, C8A, C9, CETP, CEACAM7, COL19A1, CPA1, CRHR2, CTRL, CYP2A7, CYP17A1, CYP19A1, CYP21A2, DAZL, DCT, DSP, EFNA2, EPHA3, F9, F13B, FCN2, GABRA1, GABRG3, GNG3, GPR3, GRB2, GRIK4, GRIN2A, GRM4, GRM7, GUCA1B, ONECUT1, HP, HTN3, HTR1D, HTR1E, HTR6, IBSP, IFNAR1, IFNW1, CXCR2, IL12RB1, INHA, IRF4, KCNA4, KCNC3, KCND3, KCNJ1, KCNJ13, KRT19, KRT33B, KRT83, KRT84, LECT2, LPO, MAGEB4, MCF2, SMCP, TRPM1, MOBP, MUC7, MYF6, MYOC, MYOD1, NKX6-1, NPY5R, NTRK1, NTRK2, PAX7, PDHA2, PLP1, PMP2, POU1F1, PPEF2, PRKACG, PRL, MAPK12, SCN7A, CCL21, SEMG2, SI, SLC5A4, SLC5A5, SLC6A1, SLC6A11, SLC10A2, SLC13A1, SSX5, TAC1, TLL2, CLEC3B, TNNT2, TNNT3, TNP2, USH2A, VPREB1, ZNF16, ZNF214, SLC30A4, FGF23, TCL1A, HIST1H2BL, UNC5C, ABCB11, ADAM7, AKAP4, ALDH1A2, PHOX2B, WASL, HTR3B, FHL5, NR1I3, NR2E3, SSX3, CACNG3, TACC3, RPP38, C6orf10, FRS3, SMR3B, ZPBP, KERA, INSL6, CYP4F8, RBPJL, CLCA4, FSTL4, CRB1, SPO11, AIPL1, SLC24A2, AHCTF1, CLEC4E, FBXO22, INVS, RBMXL2, ADAMDEC1, BMP10, PNMA3, PDZRN4, CALY, TAS2R14, PDE11A, MRPS7, MYO15A, TFDP3, SPTBN5, MS4A4A, AMOTL2, SLCO1C1, GPR85, DDX4, MAGEL2, DNAJC28, ACSM5, PDPR, HJURP, DNAH3, IL26, TEX12, C11orf16, MEPE, SLC17A6, CASS4, CD177, MYOZ1, HPSE2, NEUROD6, ELSPBP1, NPVF, FBRS, MRPS15, TRIM48, ADM2, TRPM3, ZKSCAN3, SLCO5A1, SPACA1, CCDC70, BRIP1, MYLK3, ATXN3L, ZXDB, KRT24, KHDRBS2, GATC
Basophils_FANTOM_3___33	Aran_et_al_2017	SCGB2A2, NPY5R, SSX5, NR2E3, SSX3, PCDHA2, C11orf16, TMEM212
Basophils_NOVERSHTERN_1___34	Aran_et_al_2017	CLK1, DEFA4, GPR183, ERN1, FCN1, FGR, GRK6, GTF3C1, GZMH, GZMB, HRH2, KLRD1, LAIR2, MMP8, NKG7, PF4V1, PI4KB, PLCB2, RFX2, RGS1, RNASE2, S100A12, XCL1, VIM, VAPA, CD101, GSTO1, BCL2L11, NMUR1, GNLY, LILRA1, LILRA2, JMJD6, PADI4, TBK1, WRAP53, RETN, CYSLTR2, DENND1A, NAA16, TUBB1, POLDIP3
Basophils_NOVERSHTERN_2___35	Aran_et_al_2017	CEACAM8, FCN1, PI4KB, NCR1, BCL2L11, NMUR1, NXT1, DERL2, WRAP53, DENND1A, POLDIP3
Basophils_NOVERSHTERN_3___36	Aran_et_al_2017	FCN1, NCR1, BCL2L11, NMUR1, NXT1, WRAP53, DENND1A, POLDIP3
CD4_memory_Tcells_FANTOM_1___37	Aran_et_al_2017	AAMP, ACTL6A, ADSL, AKT2, ANXA7, ARL2, ATF1, BTF3, CCNC, CD2, CD5, CD6, CD28, CDK9, CETN3, CCR4, COX7C, CSNK1A1, CSNK2A2, CTBP1, CTLA4, DAD1, DDX3X, DR1, GPR183, EIF4G2, ERH, ESD, FNTA, GABPA, GLUD1, HDAC1, HINT1, HMOX2, HNRNPH3, HNRNPU, DNAJB1, EIF3E, ITK, JAK3, KIF22, KTN1, LDHB, SH2D1A, SMAD2, MAGOH, NCL, NDUFS5, PFN1, PLP2, POLD2, PPID, PPP1CB, PPP1CC, PPP2R5D, PPP6C, PTPN11, RAD21, RANBP1, RBM3, RGS1, RPL4, RPL5, RPL8, RPL13, RPS3, RPS6, RPS19, SLAMF1, SOD1, SP3, SRP9, SURF2, THOP1, TPP2, TPT1, TSN, TSSC1, UBE2D2, UBE2D3, UBE2N, UQCRC2, CNBP, DAP3, FXR1, UXT, PKP4, API5, PRPF18, DENR, STK16, RUVBL1, SSNA1, PABPC4, SUCLG1, GALR2, DPM1, NAE1, EIF2B5, FUBP3, BUB3, WDR46, ATG5, PSMF1, BAG3, PREPL, TTC37, SAFB2, CEP57, MATR3, RANBP9, ARPC4, BCAS2, DNAJA2, MAEA, PPIH, PFDN6, HMGN4, EIF3M, ANP32B, TBL3, USP39, PTGES3, CLPX, PAPOLA, SUB1, DBF4, C11orf58, HNRNPA0, COPS5, CPSF6, NUPL2, PDCD10, CBX3, U2AF2, RRP1B, MRPS27, SMC5, METAP1, RPL13A, CD2AP, EID1, ATXN10, RPL36, AHCTF1, ZZZ3, PPA2,

		SERP1, MMADHC, PRPF19, MRPS18B, METTL5, ICOS, TRA2A, UBIAD1, GPR132, UBQLN2, GLOD4, FCF1, COPS4, RSL24D1, CDC40, EIF3L, RWDD1, TRMT112, CMPK1, ETAA1, GATAD2A, MRPL20, SMU1, IMP3, LIN7C, CDV3, CDKN2AIP, AMBRA1, OSGEP, INTS8, KBTBD4, EXOC2, PCID2, ZC3H15, UNC45A, NKRF, PCNP, THAP11, ZDHHC6, MRPL11, ACD, MRPL44, MRPS34, DDX50, SPAG16, CBLL1, RPF1, THOC7, GRPEL1, ISCA1, DOHH, C12orf29, ATPIF1
CD4_memory_Tcells_FANTOM_2___38	Aran_et_al_2017	CD6, CD28, GPR183, EIF3E, ITK, PKP4, PREPL, DNAJA2, PTGES3, CD2AP, ICOS
CD4_memory_Tcells_FANTOM_3___39	Aran_et_al_2017	ADSL, SLC25A6, ANXA7, ATF1, BTF3, CD5, CD6, CD28, CCR4, COX7C, CSNK2A2, CTBP1, DAD1, DDX3X, DR1, GPR183, EEF2, EIF4G2, ESD, FNTA, GLUD1, HDAC1, HINT1, HNRNPH3, DNAJB1, EIF3E, ITK, KARS, KIF22, KTN1, LDHB, SH2D1A, SMAD2, NDUFS5, PFN1, PLP2, PPP1CB, PPP1CC, PPP2R5D, PPP6C, RAD21, RBM3, RNF6, RPL5, RPL8, RPS3, RPS6, RPS19, SLAMF1, SOD1, SP3, SRP9, SURF2, TSN, UBE2D2, UBE2D3, CNBP, DAP3, FXR1, UXT, PKP4, PRPF18, DENR, RUVBL1, SSNA1, PABPC4, NAE1, FUBP3, BUB3, WDR46, ATG5, PSMF1, BAG3, PREPL, TTC37, CEP57, MATR3, THRAP3, ARPC4, BCAS2, DNAJA2, HMGN4, EIF3M, ANP32B, FARS2, USP39, PTGES3, PAPOLA, SUB1, C11orf58, HNRNPA0, CBX3, RBM34, RRP1B, SMC5, METAP1, RPL13A, CD2AP, EID1, RPL36, ZZZ3, TINF2, SERP1, PRPF19, ICOS, TRA2A, UBIAD1, GPR132, UBQLN2, GLOD4, FCF1, RSL24D1, CDC40, EIF3L, RWDD1, TRMT112, CMPK1, ETAA1, GATAD2A, SLC25A38, IMP3, CDV3, CDKN2AIP, AMBRA1, EXOC2, PCID2, UNC45A, NKRF, PCNP, THAP11, ZDHHC6, MRPL11, MRPS34, DDX50, CBLL1, RPF1, THOC7, ISCA1, C12orf29
CD4_memory_Tcells_IRIS_1___40	Aran_et_al_2017	CD3G, CD28, CD40LG, CCR4, CTLA4, GPR15, LIMS1, RBL2, DLEC1, CXCR6, PDCD10, TRAT1, ARHGAP15
CD4_memory_Tcells_IRIS_2___41	Aran_et_al_2017	CD28, CD40LG, CCR4, CTLA4, GPR15, GZMA, GZMK, HMGB2, PDCD1, RBL2, CD226, GPR171, TRAT1, UBASH3A, ARHGAP15
CD4_memory_Tcells_IRIS_3___42	Aran_et_al_2017	ADSL, CD28, CD40LG, CCR4, CTLA4, GZMA, GZMK, HMGB2, LIMS1, MEN1, PTPN4, ZNF236, DLEC1, CD96, HMGN4, SEC23IP, ICOS, TRAT1, AURKAIP1, ARHGAP15, RNF34
CD4_naive_Tcells_FANTOM_1___43	Aran_et_al_2017	CD3E, CD6, CD7, CCR7, DSC1, JAK3, PLCL1, PLCG1, ZAP70, CABIN1, PLXDC1, GPSM3, ANKRD55, LIMD2, CHMP7
CD4_naive_Tcells_FANTOM_2___44	Aran_et_al_2017	APBB1, CD3E, CD6, CD7, CD27, TNFSF8, CCR7, DSC1, GRK6, GZMM, PRMT2, IDUA, INSL3, ITK, JAK3, NUMA1, PHF1, PLCL1, PLCG1, RXRB, SELPLG, ZAP70, ZNF76, CUBN, PIP4K2B, NCK2, CDK10, TNK1, ACAP1, TSPAN32, ARFRP1, 44083, MSL3, WDR6, MLXIP, LEPROTL1, CABIN1, SH2B1, IPCEF1, KLHL3, SIT1, TRAT1, CRLF3, RAPGEF6, FAM193B, SIRPG, PACS1, PLXDC1, RNPEPL1, CREBZF, GPSM3, ANKRD55, NDFIP1, LIMD2, OBSCN, CHMP7
CD4_naive_Tcells_FANTOM_3___45	Aran_et_al_2017	CD27, CLC, CTSW, DNAJB1, RBL2, HAUS3, ANKRD55, ZNF394, CHMP7
CD4_naive_Tcells_HPCA_1___46	Aran_et_al_2017	CD6, CCR7, DSC1, GLG1, LY9, MAK, PLCL1, RBL2, RBMS1, ATXN7, CUL1, MTRF1, BMS1, CEPT1, AAK1, SORCS3, CLUAP1, IPCEF1, ICOS, COQ6, PHF20L1, POP5, RAPGEF6, NUDT9, UBASH3A, GIN1, SETD5, KDM3A, PLXDC1, NAA16, ANKRD55, TRAF3IP3, NDFIP1, CHMP7, TMEM30B
CD4_naive_Tcells_HPCA_2___47	Aran_et_al_2017	CCR7, FKTN, IL16, KRT2, NPAT, RBMS1, VPS52, MTRF1, CEPT1, SORCS3, UTP20, PHF20L1, POP5, RAPGEF6, RNF216, TUG1, PLXDC1, ANKRD55, TRAF3IP3, CHMP7
CD4_naive_Tcells_HPCA_3___48	Aran_et_al_2017	CD6, CCR7, FKTN, IL16, KRT2, NPAT, PLCL1, RBMS1, VPS52, MTRF1, CEPT1, AAK1, SORCS3, UTP20, PHF20L1, POP5, RAPGEF6, GIN1, TUG1, PLXDC1, NOL9, ANKRD55, TRAF3IP3, CHMP7
CD4_naive_Tcells_IRIS_1___49	Aran_et_al_2017	CD3G, CD4, CD5, CD7, HMOX2, NPAT, RPA3, RPLP2, RPL14, SNPH, TBC1D5, RAB3GAP1, MLH3, SIRPG, PARP11, MKL1, GIMAP6
CD4_naive_Tcells_IRIS_2___50	Aran_et_al_2017	CD4, CD5, CD7, CD40LG, INPP4A, NPAT, TRAF1, TBC1D5, SIRPG, PARP11, MKL1, GIMAP6
CD4_naive_Tcells_IRIS_3___51	Aran_et_al_2017	CD2, CD3G, CD247, CD4, CD5, CD7, CD27, CD40LG, CCR7, HMOX2, PRMT2, INPP4A, NPAT, RPA3, RPL38, RPLP2, RPS6, TRAF1, RPL14, ZNF264, SNPH, TBC1D5, ZBTB40, PRMT3, USP16, NUP50, RAB3GAP1, ZNF609, RPRD2, LEPROTL1, MLH3, TRAT1, FCF1, SIRPG, PARP11, PLXDC1, MKL1, COPS7B, DDX31, KRI1, DDX50, ACBD4, SLTM, RPAP2, WDR82, ZNF780B, GIMAP6
CD4_naive_Tcells_NOVERSHTERN_1___52	Aran_et_al_2017	CASP8, CD3E, CD4, CD27, CD28, CD40LG, CDK1, CCR7, CTLA4, HMOX2, LAIR2, PLCL1, RBL2, SUPV3L1, TPP2, GRAP2, SNPH, ZBTB40, ZNF263, USP16, CD226, CA5B, ZNF609, FNBP4, NUDCD3, LEPROTL1, STAP1, SIT1, ICOS, TRAT1, PHF20L1, TEX264, SIRPG, POLR3E, PLXDC1, MKL1, DPEP2, COPS7B, GIMAP6

CD4_naive_Tcells_NOVERSHTERN_2___53	Aran_et_al_2017	APBB1, PRMT2, TATDN2, DIDO1, REV1, RNF216, PLXDC1, NDFIP1, CHMP7
CD4_naive_Tcells_NOVERSHTERN_3___54	Aran_et_al_2017	CD3E, CD6, CD7, CCR7, DSC1, JAK3, PLCL1, PLCG1, ZAP70, CABIN1, PLXDC1, GPSM3, ANKRD55, LIMD2, CHMP7
CD4_Tcells_BLUEPRINT_1___55	Aran_et_al_2017	BAD, CD2, CD3G, CD5, CD28, CD40LG, CCR4, CCR7, CTLA4, GOLGA4, HMOX2, MGAT2, NFE2L2, PLCL1, PPP2CA, SON, SUPV3L1, TRAF1, TSPYL1, FBXO21, FNBP4, NUDCD3, LEPROTL1, ICOS, TRAT1, FOXP3, SIRPG, POLR3E, USP36, DDX31, RIC3, SLTM, HIPK1, ATXN7L1
CD4_Tcells_BLUEPRINT_2___56	Aran_et_al_2017	BAD, CD2, CD3E, CD3G, CD5, CD6, CD28, CD40LG, CCR4, CCR7, CTLA4, DNAH6, GOLGA4, HMOX2, HNRNPU, DNAJB1, MBNL1, MGAT2, NFE2L2, PLCL1, PPP2CA, SFPQ, SON, SUPV3L1, TPP2, TRAF1, TSPYL1, UBP1, ZAP70, OFD1, HERC1, RBM19, DLEC1, CCR9, FBXO21, ZCCHC11, FNBP4, NUDCD3, PHF3, LEPROTL1, LSM14A, TOR1AIP1, ICOS, IL21R, TRAT1, FOXP3, WBP11, USP47, THAP1, SIRPG, POLR3E, MKL1, USP36, RBM25, DDX31, RSRC2, ZXDC, HAUS3, RIC3, C14orf169, SLTM, ARID5B, TOE1, TUBGCP5, HIPK1, ATXN7L1, SNX19
CD4_Tcells_BLUEPRINT_3___57	Aran_et_al_2017	BAD, CD2, CD3G, CD5, CD28, CD40LG, CCR4, CTLA4, GOLGA4, HMOX2, MGAT2, NFE2L2, PLCL1, PPP2CA, SUPV3L1, TSPYL1, NUDCD3, LEPROTL1, ICOS, TRAT1, FOXP3, WBP11, SIRPG, POLR3E, USP36, DDX31, HAUS3, SLTM, HIPK1
CD4_Tcells_FANTOM_1___58	Aran_et_al_2017	APBB1, CD5, CD6, CD28, CD40LG, CCR7, CTLA4, ITK, PLCL1, PLCG1, PSD, SSTR3, TTN, CUBN, SNPH, SPEG, MSL3, FNBP4, KLHL3, ICOS, RAPGEF6, PLXDC1, NOL9, ANKRD55, OBSCN, CHMP7
CD4_Tcells_FANTOM_2___59	Aran_et_al_2017	APBB1, CD28, CTLA4, ITK, PLCL1, SNPH, PPWD1, PHF3, ICOS, TRAT1, RAPGEF6, NOL9, CHMP7
CD4_Tcells_FANTOM_3___60	Aran_et_al_2017	KRIT1, CCNT2, CD2, CD4, CD28, TNFSF8, CCR7, CCR8, DDX5, DSC1, GPR183, EZH1, FCN1, GP5, PRMT2, ITIH4, ITK, LY9, PABPC3, POU6F1, PPM1B, RBL2, RXRG, ATXN7, TPT1, NR2C1, CUBN, NCK2, CDC14A, FUBP1, UBA3, SGSM2, HMGN4, CCR9, MSL3, DIDO1, CA5B, AAK1, MORC2, SACM1L, USP33, LEPROTL1, MTO1, IPCEF1, ZBTB11, SIT1, PNMA3, UBQLN2, TRAT1, CRLF3, RAPGEF6, UBASH3A, LAX1, TUG1, PRPF38B, ASXL2, GIMAP4, SIRPG, THUMPD1, ARHGAP15, ZC3HAV1, PLXDC1, ANKRD55, ALG13, CBLL1, TRMT2B, TRIM46, FBXO11, TRAF3IP3, ZNF611, OBSCN, NFATC2IP, CHMP7, TMEM123, ZFC3H1, GIMAP6, CCR2
CD4_Tcells_HPCA_1___61	Aran_et_al_2017	ABCD2, BAD, CD2, CD3D, CD3E, CD3G, CD5, CD7, CD27, CD28, CD40LG, CCR4, CTLA4, GOLGA4, HMOX2, NFE2L2, PLCL1, PPP2CA, SON, SUPV3L1, TNFRSF4, ARHGEF1, TOMM20, CD96, FBXO21, NUDCD3, LEPROTL1, ICOS, GPR171, TRAT1, FOXP3, SIRPG, POLR3E, USP36, ZNF335, RIC3, HIPK1
CD4_Tcells_HPCA_2___62	Aran_et_al_2017	BAD, CD2, CD3D, CD3E, CD3G, CD5, CD6, CD7, CD27, CD28, CD40LG, CCR4, CTLA4, HMOX2, PLCL1, PPP2CA, SON, SUPV3L1, TSPYL1, ARHGEF1, CD96, NUDCD3, LEPROTL1, ICOS, GPR171, FOXP3, SIRPG, ARHGAP15, HAUS3, HIPK1
CD4_Tcells_HPCA_3___63	Aran_et_al_2017	ABCD2, BAD, CD2, CD3D, CD3E, CD3G, CD5, CD7, CD27, CD28, CD40LG, CCR4, CTLA4, GOLGA4, HMOX2, MGAT2, PLCL1, PPP2CA, SON, SUPV3L1, TRAF1, TNFRSF4, ARHGEF1, CD96, NUDCD3, LEPROTL1, ICOS, GPR171, TRAT1, FOXP3, ZNHIT6, SIRPG, HAUS3, RIC3, HIPK1
CD4_Tcm_BLUEPRINT_1___64	Aran_et_al_2017	ADSL, AK1, CD247, CD40LG, CSNK1D, CTLA4, DNMT1, GOLGB1, ITIH4, MAN2C1, NFRKB, PLCG1, POLR2A, PSMD2, RAD9A, SPTAN1, SUPT6H, NR2C1, TSC1, USP4, DGCR14, DHX16, RAE1, BAG3, SGSM2, HUWE1, RBM5, NXF1, SORCS3, MYO16, EDC4, MTO1, ZNF638, CNIH4, ICOS, FAM193B, FBXL8, EXOC1, KDM3A, USP36, CORO7, ANKRD55, CBLL1
CD4_Tcm_BLUEPRINT_2___65	Aran_et_al_2017	AK1, CD2, CD247, CD40LG, CCR7, CTLA4, DNMT1, GOLGB1, ITIH4, NFRKB, PLCG1, SPTAN1, TPR, NR2C1, TSC1, DHX16, TRADD, BAG3, SGSM2, RBM5, MSL3, AAK1, SORCS3, MYO16, EDC4, CNIH4, ICOS, FAM193B, FBXL8, EXOC1, KDM3A, ARHGAP15, YLPM1, USP36, ANKRD55, WDR59, CBLL1, TRAF3IP3, 44084
CD4_Tcm_BLUEPRINT_3___66	Aran_et_al_2017	ADSL, AK1, CD2, CD247, CD40LG, CCR7, CSNK1D, CTLA4, DNMT1, GOLGB1, ITIH4, LY9, MAN2C1, NAP1L4, NFRKB, NPAT, PLCL1, PLCG1, POLR2A, PSMD2, RAD9A, SLAMF1, SPTAN1, SUPT6H, TPR, NR2C1, TSC1, USP4, ZNF200, DGCR14, DHX16, RAE1, TRADD, EIF2B5, USP10, ITGB1BP1, BAG3, ACAP1, SGSM2, HUWE1, RBM5, NME6, NXF1, USP39, FASTK, MSL3, DIDO1, WDR6, CA5B, AAK1, TCF25, SORCS3, MYO16, MCF2L2, GGA3, SMG5, EDC4, MTO1, KBTBD2, IPCEF1, ZNF638, CNIH4, ICOS, UBQLN2, COL5A3, ANAPC5, UBASH3A, FAM193B, PIGG, OLAH, FBXL8, EXOC1, KDM3A, ARHGAP15, PCDHGA9, YLPM1, INPP5E, DDX24, USP36, CORO7, ANKRD55, WDR59, CBLL1, NUP85, TRAF3IP3, 44084, CAMSAP1

CD4_Tcm_HPCA_1___67	Aran_et_al_2017	BMPR1A, CD4, CD5, CD6, CD28, TNFSF8, CD40LG, CD48, CTLA4, DAB1, SARDH, DVL1, ERN1, GP5, GPR15, IDUA, ITK, KRT1, POU6F1, PSD, RPL38, SCNN1D, TCF20, TPO, TNFRSF4, XPC, NCK2, CDC14A, TRADD, SNPH, DLEC1, SPEG, HNRNPUL1, MORC2, NCDN, RRS1, GMEB2, ICOS, PNMA3, GLTSCR2, TRAT1, UBASH3A, FXYD7, FBXL8, KBTBD4, ARHGAP15, ZC3HAV1, SLC4A5, DNAI2, ANKRD55, TRIM46, OBSCN, ARID5B, TRMT61A, ZXDB, TMEM30B
CD4_Tcm_HPCA_2___68	Aran_et_al_2017	BMPR1A, CD4, CD5, CD6, CD28, TNFSF8, CD40LG, CD48, CCR4, CRY2, CTLA4, DAB1, SARDH, DVL1, ERN1, GP5, GPR15, DNAJB1, IDUA, ITK, KRT1, LTA, POU6F1, PSD, PURA, RPL38, RPS16, RPS21, SCNN1D, STK11, TCF20, TFAP4, TPO, TNFRSF4, XPC, ZRSR2, NCK2, CDC14A, TRADD, ACAP1, SNPH, JOSD1, DLEC1, SPEG, IKZF1, 44083, HNRNPUL1, MORC2, TAB2, NCDN, RRS1, GMEB2, ICOS, PNMA3, GLTSCR2, TRAT1, UBASH3A, FXYD7, SNTG2, TOMM7, FBXL8, SIRPG, CDKN2AIP, KBTBD4, ARHGAP15, ZC3HAV1, THAP11, NDRG3, SLC4A5, DNAI2, ANKRD55, TRIM46, OBSCN, ARID5B, TRMT61A, ZXDB, TMEM30B
CD4_Tcm_HPCA_3___69	Aran_et_al_2017	CD5, CD6, CD28, TNFSF8, CD40LG, CD48, CTLA4, DAB1, ERN1, GP5, GPR15, ITK, KRT1, POU6F1, PSD, RPL38, TPO, TNFRSF4, NCK2, CDC14A, TRADD, SNPH, DLEC1, MORC2, RRS1, ICOS, GLTSCR2, TRAT1, UBASH3A, FXYD7, FBXL8, ARHGAP15, DNAI2, ANKRD55, OBSCN, TRMT61A, TMEM30B
CD4_Tcm_NOVERSHTERN_1___70	Aran_et_al_2017	CD40LG, CCR4, CCR8, CTLA4, DAB1, ERN1, GPR15, DNAJB1, KRT1, POU6F1, TPO, CDC14A, TRADD, DLEC1, ICOS, TRAT1, FXYD7, FBXL8, SIRPG, SLC4A5, ANKRD55, OBSCN
CD4_Tcm_NOVERSHTERN_2___71	Aran_et_al_2017	CCR4, CCR8, CTLA4, DAB1, ERN1, GPR15, DNAJB1, KRT1, POU6F1, TPO, TRADD, DLEC1, ICOS, TRAT1, FXYD7, FBXL8, SIRPG, ANKRD55, OBSCN
CD4_Tcm_NOVERSHTERN_3___72	Aran_et_al_2017	APBB1, CD5, CD6, CD28, TNFSF8, CD40LG, CD48, CCR4, CCR6, CCR7, CCR8, CTLA4, DAB1, SARDH, DVL1, ERN1, GP5, GPR15, GPR25, DNAJB1, IL2RA, ITK, KRT1, PLCG1, POU6F1, PSD, RPL38, TPO, TNFRSF4, NCK2, CDC14A, TRADD, KALRN, SOCS3, ZFYVE9, PLCH2, SNPH, DLEC1, 44083, MORC2, RRS1, ICOS, PNMA3, GLTSCR2, COL5A3, TRAT1, UBASH3A, FXYD7, SNTG2, FBXL8, SIRPG, ARHGAP15, PLXDC1, DNAI2, ANKRD55, OBSCN, ZXDB, TMEM30B
CD4_Tem_BLUEPRINT_1___73	Aran_et_al_2017	RPN2, SLAMF1, SPTAN1, TRADD, MYO16, MCF2L2, ESYT1, TRAPPC2L, ARHGAP15, RIC8A
CD4_Tem_BLUEPRINT_2___74	Aran_et_al_2017	TNFSF8, GPR25, SELPLG, SMARCC2, LZTR1, TRADD, CCR9, SIT1, FBXL8, RNPEPL1
CD4_Tem_BLUEPRINT_3___75	Aran_et_al_2017	CD2, CTLA4, DNAJB1, RGS1, CLIP1, SLAMF1, SPTAN1, DYNLT1, COLQ, CDC14A, TRADD, RANBP9, CCR9, MYO16, MCF2L2, ICOS, TRAT1, FBXL8, SMAP1, SAMSN1, HAUS3, 44084
CD4_Tem_HPCA_1___76	Aran_et_al_2017	ARAF, CAPZB, CCNT1, CD2, CD3E, CD4, CD5, CD40LG, CD52, COPB1, CTLA4, DYNC1H1, E4F1, EMD, GOLGB1, GPI, CXCR3, GRM3, DNAJB1, IDE, IK, IL10RA, ITIH4, ITK, MARK3, NDUFB2, NFKB1, NFRKB, PABPC3, PGK1, PLCL1, PSMD9, RABGGTA, RBL1, RGS1, RPN2, RRM1, S100A11, SELPLG, SLAMF1, SOS1, SPTAN1, SSR2, TAF10, TBCC, TCF20, DYNLT1, TRAF1, TUFM, XPC, BRPF1, DGCR14, RBM10, COLQ, MAPKAPK5, CDC14A, MKNK1, EIF3A, EIF3G, TRADD, RIPK1, TAF1B, USP10, MTMR6, ZFYVE9, BAG3, APBA3, RNF7, SEC24C, N4BP1, MAML1, RANBP9, SLC35B1, BCAS2, SF3A1, SLC9A6, CGRRF1, DCTN6, GIPC1, 44083, CCR9, JTB, COPS6, SF3B2, CDC37, MORC2, TCF25, CNOT1, MYO16, MCF2L2, JMJD6, RRS1, ESYT1, KIAA0368, CABIN1, COG4, AHCTF1, IBTK, GORASP2, GIT1, C19orf53, ICOS, UBIAD1, COL5A3, TRAT1, GOLGA7, RWDD1, TRAPPC4, VPS54, SRRT, TRAPPC2L, DDX56, STX17, FBXL8, THUMPD1, NECAP2, ARHGAP15, ZC3HAV1, RNPEPL1, USP36, ELAC2, RIC8A, SMAP1, GPSM3, SAMSN1, NSD1, NOL6, PVRIG, TNIP2, HAUS3, FYCO1, CDC73, FBXO31, MUS81, ZNF394, SPSB3, SLC38A10, ASB6, 44084
CD4_Tem_HPCA_2___77	Aran_et_al_2017	AIRE, CD5, CD6, CD28, TNFSF8, CD40LG, CD48, CCR4, CCR6, CCR8, CTLA4, ERN1, GPR15, GZMK, DNAJB1, IL5RA, ITGB7, KLRB1, KRT1, LTA, LTK, OSM, PDCD1, RPL38, RPS21, SPTAN1, TPO, TNFRSF4, TKTL1, HIST1H3A, NCK2, CDC14A, TRADD, DLEC1, STUB1, HMGN4, CCR9, MCF2L2, NCDN, RRS1, ESYT1, GALNT8, GPR171, GLTSCR2, TRAT1, UBASH3A, FXYD7, FBXL8, KBTBD4, ARHGAP15, SAMSN1, DNAI2, CCR2
CD4_Tem_HPCA_3___78	Aran_et_al_2017	RPN2, SLAMF1, SPTAN1, TRADD, MYO16, MCF2L2, ESYT1, TRAPPC2L, ARHGAP15, RIC8A
CD4_Tem_NOVERSHTERN_1___79	Aran_et_al_2017	AIRE, CD5, CD6, CD28, TNFSF8, CD40LG, CD48, CCR4, CCR6, CCR8, CTLA4, ERN1, GPR15, GZMK, DNAJB1, IL5RA, ITGB7, KLRB1, KRT1, LTA, LTK, OSM, PDCD1, RPL38, RPS21, TPO, TNFRSF4, HIST1H3A, NCK2, CDC14A, TRADD, DLEC1, CCR9, MCF2L2, RRS1, GALNT8, GPR171, GLTSCR2, TRAT1, FXYD7, FBXL8, ARHGAP15, SAMSN1, CCR2

CD4_Tem_NOVERSHTERN_2___80	Aran_et_al_2017	AIRE, CD40LG, CD48, CCR6, CCR8, ERN1, KLRB1, LTK, TPO, CCR9, MCF2L2, GALNT8, GLTSCR2, TRAT1, CCR2
CD4_Tem_NOVERSHTERN_3___81	Aran_et_al_2017	AIRE, CD40LG, CD48, CCR6, CCR8, ERN1, KLRB1, LTK, TPO, CCR9, MCF2L2, GALNT8, GLTSCR2, TRAT1, CCR2
CD8_naive_Tcells_HPCA_1___82	Aran_et_al_2017	AMBN, CD8A, CD8B, CCR8, COX4I1, EEF1D, GPR15, HTR1B, SMCP, MYL1, NDUFA1, NDUFA4, NDUFS5, NKTR, OMG, PSG11, SKI, SON, TPP2, SHFM1, HIST1H3A, HIST1H4F, RNMT, BUD31, GPR52, ZNHIT3, RNF7, JOSD1, SLC17A4, BCAS2, RPP38, CGRRF1, RRH, SMR3B, JMJD6, RRP8, CA14, NGDN, C19orf53, SETD2, IL21R, MED31, SS18L2, LUZP4, CDK5RAP1, FXYD7, GDAP2, CCDC87, CWF19L1, DDX24, USP36, PCIF1, MS4A5, HAUS3, LIN28A, BTNL8, SLC35E1, MOGAT2, TNKS2, KIAA1109, GJB4, HIGD2A
CD8_naive_Tcells_HPCA_2___83	Aran_et_al_2017	CD8A, CD8B, EEF1D, GPR15, MYL1, NDUFA4, NDUFS5, PSG11, SKI, SON, HIST1H3A, BUD31, GPR52, ZNHIT3, CGRRF1, RRP8, NGDN, C19orf53, SETD2, MED31, SS18L2, CDK5RAP1, DDX24, PCIF1, MS4A5, HAUS3, LIN28A, TNKS2
CD8_naive_Tcells_HPCA_3___84	Aran_et_al_2017	CD8A, CD8B, EEF1D, GPR15, KRT1, MYL1, NDUFA4, NDUFS5, PSG11, RFX2, SKI, HIST1H3A, CILP, GPR52, SLC17A4, JMJD6, CA14, NGDN, C19orf53, SETD2, IL21R, MED31, FXYD7, DDX24, MS4A5, HAUS3, LIN28A, TNKS2, GJB4
CD8_Tcells_BLUEPRINT_1___85	Aran_et_al_2017	CD8A, CD8B, CD27, CCR7, DSC1, PRMT2, IL16, MMP19, NFKB1, NPAT, PCNT, PFN2, PURA, RING1, MTRF1, TSPAN32, CD96, CEPT1, MSL3, DIDO1, AAK1, RBM34, CLUAP1, CBY1, POP5, RAPGEF6, YLPM1, CRTAM, CIAPIN1, TRAF3IP3
CD8_Tcells_BLUEPRINT_2___86	Aran_et_al_2017	CA6, CD7, CD8A, CD8B, CD27, CCR7, CTSW, DSC1, FKTN, PRMT2, IL16, MMP19, NDUFS2, NFKB1, NPAT, PCNT, PFN2, PURA, RING1, S100B, ZNF200, MYOM1, MTRF1, TSPAN32, CD96, CEPT1, SDCCAG3, MSL3, DIDO1, BTN2A1, COG2, AAK1, RBM34, CLUAP1, CBY1, UTP20, UBQLN2, POP5, RAPGEF6, DPP8, CCDC25, POLR3E, NKRF, YLPM1, CRTAM, CIAPIN1, PLXDC1, GGNBP2, WDR82, TRAF3IP3, NDFIP1, TMEM41B
CD8_Tcells_BLUEPRINT_3___87	Aran_et_al_2017	CA6, CD7, CD8A, CD8B, CD27, CCR7, CTSW, DSC1, PCNT, PFN2, MYOM1, ARHGEF1, MTRF1, MSL3, CLUAP1, POP5, CRTAM, CIAPIN1, NAA16, TRAF3IP3, NDFIP1
CD8_Tcells_FANTOM_1___88	Aran_et_al_2017	CA6, CD7, CD8A, CD8B, CD27, CCR7, CTSW, DSC1, FKTN, PRMT2, IL16, MMP19, NDUFS2, NFKB1, NPAT, PCNT, PFN2, PURA, RING1, S100B, ZNF200, MYOM1, MTRF1, TSPAN32, CD96, CEPT1, SDCCAG3, MSL3, DIDO1, BTN2A1, COG2, AAK1, RBM34, CLUAP1, CBY1, UTP20, UBQLN2, POP5, RAPGEF6, DPP8, CCDC25, POLR3E, NKRF, YLPM1, CRTAM, CIAPIN1, PLXDC1, GGNBP2, WDR82, TRAF3IP3, NDFIP1, TMEM41B
CD8_Tcells_FANTOM_2___89	Aran_et_al_2017	CASP8, CD8A, CD8B, GZMK, PTGDR, SLC1A7, TSPAN32, KLRG1, NPRL2, GIMAP4, CRTAM, ZNF611
CD8_Tcells_FANTOM_3___90	Aran_et_al_2017	APBB1, CA6, CD8A, CD8B, DHX15, DSC1, GZMM, HNRNPL, PRMT2, KRT2, LY9, PCNT, PLCG1, PRL, PSD, RASA2, RBL2, RPL37A, S100B, SFPQ, SSTR3, TTN, ZNF154, PRPF4B, MED17, CD96, HNRNPA0, FNBP4, LSM14A, KLHL3, ZBTB11, SHANK1, ZNF639, USP47, CRTAM, ZC3HAV1, PLXDC1, GJC2, GGNBP2, NDFIP1
CD8_Tcells_HPCA_1___91	Aran_et_al_2017	CD8A, CD8B, GZMK, IRF3, LY9, TBCC, TSPAN32, KLRG1, DPP8, SDAD1, FTO
CD8_Tcells_HPCA_2___92	Aran_et_al_2017	CD3D, CD8A, CD8B, DSC1, GZMK, IRF3, LY9, PCNT, TBCC, RNF113A, TSPAN32, KLRG1, CD160, COG2, COPZ1, MKRN2, DPP8, SDAD1, UBE2Q1, C7orf26, FTO, EML3
CD8_Tcells_HPCA_3___93	Aran_et_al_2017	CD8A, CD8B, CD27, CTSW, CX3CR1, EEF1D, GZMH, GZMK, DNAJB1, KLRB1, LAIR2, LY9, PTGDR, PTPN4, RBL2, FBXW4, TSPAN32, KLRG1, CD160, RWDD3, IPCEF1, TOMM7, SIRPG, NAA16, FAM134C
CD8_Tcells_IRIS_1___94	Aran_et_al_2017	CD8A, CD8B, DSC1, PCNT, PLCG1, PSD, RBL2, SSTR3, CD96, PLXDC1
CD8_Tcells_IRIS_2___95	Aran_et_al_2017	CD8A, CD8B, CD27, DSC1, MMP19, MYOM1, MTRF1, CRTAM, CIAPIN1
CD8_Tcells_IRIS_3___96	Aran_et_al_2017	CD8A, CD8B, CD27, DSC1, MMP19, NFKB1, PCNT, RING1, MYOM1, MTRF1, COG2, CBY1, CCDC53, NKRF, CRTAM, CIAPIN1, GGNBP2
CD8_Tcells_NOVERSHTERN_1___97	Aran_et_al_2017	CA6, CD8A, CD8B, DHX15, DSC1, PRMT2, KRT2, LY9, PCNT, PLCG1, PRL, PSD, RASA2, RBL2, RPL37A, S100B, SSTR3, TTN, ZNF154, CD96, HNRNPA0, FNBP4, ZBTB11, SHANK1, USP47, CRTAM, ZC3HAV1, PLXDC1, GGNBP2, NDFIP1
CD8_Tcells_NOVERSHTERN_2___98	Aran_et_al_2017	CD8A, CD8B, GZMK, IRF3, LY9, TBCC, TSPAN32, KLRG1, DPP8, SDAD1, FTO

CD8_Tcells_NOVERSHTERN_3___99	Aran_et_al_2017	APBB1, CA6, CD8A, CD8B, DHX15, DSC1, GZMM, HNRNPL, PRMT2, KRT2, LY9, PCNT, PLCG1, PRL, PSD, RASA2, RBL2, RPL37A, S100B, SFPQ, SSTR3, TTN, ZNF154, PRPF4B, MED17, CD96, HNRNPA0, FNBP4, LSM14A, KLHL3, ZBTB11, SHANK1, ZNF639, USP47, CRTAM, ZC3HAV1, PLXDC1, GJC2, GGNBP2, NDFIP1
CD8_Tcm_BLUEPRINT_1___100	Aran_et_al_2017	CASP8, CD27, TNFSF8, GZMK, NCK1, GPR171, CRTAM, PARP11, TMEM30B
CD8_Tcm_BLUEPRINT_2___101	Aran_et_al_2017	ADCYAP1R1, ABCD2, ALK, CASP8, CD8A, CD8B, CD27, CD28, TNFSF8, CD48, ESR2, CXCR3, GZMK, LAG3, SH2D1A, MMP11, NCK1, SLC6A7, SYN2, TPP2, DUSP11, HELZ, CD96, ZMYND11, SEC24A, ZC3H13, LEPROTL1, ADAT1, CHST5, SIT1, GPR171, HAO2, GIMAP4, PCDHA10, CRTAM, PARP11, SMAP1, VPS33A, TMEM30B, GIMAP6
CD8_Tcm_BLUEPRINT_3___102	Aran_et_al_2017	ADCYAP1R1, CASP8, CD8B, CD27, TNFSF8, ESR2, GZMK, SH2D1A, NCK1, CD96, ZMYND11, SIT1, GPR171, CRTAM, PARP11, TMEM30B
CD8_Tcm_HPCA_1___103	Aran_et_al_2017	CASP8, CD8A, CD8B, CD27, GZMK, DNAJB1, LAG3, SH2D1A, RASA2, RGS1, DYNLT1, TPP2, CDC14A, CGRRF1, DCTN6, PPWD1, ADAT1, CRTAM, PARP11, USP36, HAUS3, NAA16, TMEM30B
CD8_Tcm_HPCA_2___104	Aran_et_al_2017	CD8A, CD8B, CTSW, GZMK, IL2RB, SH2D1A, GPR171, CRTAM, USP36, TMEM30B
CD8_Tcm_HPCA_3___105	Aran_et_al_2017	BMPR1A, C21orf2, CASP8, CD3E, CD8A, CD8B, CD27, CD28, CTSW, GZMH, GZMA, GZMK, DNAJB1, IL2RB, INPP4A, KIF2A, KLRD1, KRT1, LAG3, SH2D1A, PTPN4, RASA2, RGS1, RPS6KB1, ATXN7, DYNLT1, TPP2, ZAP70, DUSP11, CDC14A, TRADD, LRIG2, MED6, KLRG1, CD96, CGRRF1, DCTN6, ZBTB1, TNRC6B, PPWD1, ADAT1, GPR171, ATF7IP, CRTAM, PARP11, CYP20A1, USP36, HAUS3, NAA16, TNKS2, ISCA1, SFXN1, TMEM30B
CD8_Tcm_NOVERSHTERN_1___106	Aran_et_al_2017	ABCD2, CD3E, CD7, CD8A, CD8B, CXCR3, GZMK, LAG3, SH2D1A, PDCD1, PTPRCAP, S100B, XCL1, SGCD, TBCC, HIST1H4F, KLRG1, STUB1, HMGN4, AKAP3, GNLY, TRAF3IP1, GPR171, ELP3, GIMAP4, CRTAM, C21orf59, WDR18, INTS5, GIMAP6
CD8_Tcm_NOVERSHTERN_2___107	Aran_et_al_2017	ABCD2, CD3E, CD7, CD8A, CD8B, CXCR3, GZMK, LAG3, SH2D1A, PDCD1, PTPRCAP, S100B, XCL1, SGCD, HIST1H4F, KLRG1, STUB1, AKAP3, GNLY, TRAF3IP1, GPR171, ELP3, CRTAM, INTS5
CD8_Tcm_NOVERSHTERN_3___108	Aran_et_al_2017	CD8B, CXCR3, GZMK, PTPRCAP, SGCD, HIST1H4F, STUB1, ELP3, CRTAM, INTS5
CD8_Tem_BLUEPRINT_1___109	Aran_et_al_2017	DHX8, GZMH, GZMK, LAG3, ZAP70, COLQ, RGS9, CXCR6, PVRIG, PYHIN1
CD8_Tem_BLUEPRINT_2___110	Aran_et_al_2017	ABCF1, ACADVL, RHOG, SLC25A20, CAPZB, CD2, CD3D, CD3G, CD7, CD8A, CD8B, CCR5, COPB1, CTSW, CX3CR1, DAXX, DHX8, DIAPH1, DMWD, DYNC1H1, EMD, GOLGA1, GTF3C1, GYG1, GZMH, GZMA, GZMK, GZMM, HMOX2, DNAJB1, IDH3B, IFNG, IL10RA, ITGAL, KLRB1, KLRD1, LAG3, LAIR2, MARK3, MT2A, MYO1F, NDUFB1, NDUFB2, NDUFS6, NKG7, PABPC3, PCNT, PHKG2, PPP2R5C, PSMC5, PSME1, PTGDR, PTPN4, PTPRA, PZP, RGS1, RPN2, CCL4, SLAMF1, SNTB2, SSR2, STX4, TAF10, DYNLT1, TUFM, WAS, ZAP70, RNF113A, LZTR1, COLQ, DHX16, GPR65, MKNK1, B4GALT3, RIPK1, RGS9, IL18RAP, ARHGEF1, CIAO1, SEC24C, N4BP1, CROCC, MAML1, KIAA0196, SEC16A, PREB, KLRG1, SLC35B1, SF3A1, GNLY, CXCR6, SF3B2, WWP2, BTN2A1, CD160, PUF60, HMGXB3, SNRNP200, JMJD6, TMEM184B, RNF167, ERAL1, CYTH4, MRPL22, ABT1, UBN1, GPR171, TIMM22, NRBP1, COL5A3, RWDD1, TRAPPC4, SRRT, STX18, TRNAU1AP, IMP3, ABCF3, CHST12, UBE2Q1, AMBRA1, MAP7D1, EXOC2, WSB2, BIN3, CRTAM, MRPS22, RIC8A, NOL6, AHNAK, PVRIG, TNIP2, HAUS3, FYCO1, C14orf169, ZMYM1, MUS81, DEFB126, ARPC5L, ZNF394, RHOT2, SPSB3, PYHIN1, NCR3, CCDC85C
CD8_Tem_BLUEPRINT_3___111	Aran_et_al_2017	SLC25A20, CALM1, CD2, CD3D, CD3G, CD8A, CD8B, CTSW, DMWD, GYG1, GZMH, GZMA, GZMB, GZMK, GZMM, IFNG, IL2RB, ITGAL, KLRB1, KLRD1, LAG3, SH2D1A, MT2A, MYO1F, NKG7, PPP1CA, PRF1, MAPK13, PTGDR, PTPN4, PTPRA, PZP, CCL4, SLAMF1, SNTB2, STX4, COLQ, GPR65, RGS9, IL18RAP, CROCC, KLRG1, GNLY, NPRL2, CXCR6, CD160, CD300A, GPR171, TBX21, COL5A3, BIN2, SASH3, UBE2Q1, MAP7D1, CRTAM, CTDSP1, RIC8A, AHNAK, PVRIG, FYCO1, ZMYM1, CCDC130, ZNF394, PYHIN1
CD8_Tem_HPCA_1___112	Aran_et_al_2017	ABCD2, FASLG, ATR, BMPR1A, C8G, C21orf2, CACNB1, CAPN2, CASP8, CD2, CD3D, CD3G, CD8A, CD8B, CCR5, CSNK1G2, CX3CR1, DUSP8, E4F1, FLT4, GIPR, GOLGA4, CXCR3, GTF3C1, GZMH, GZMA, GZMB, GZMK, GZMM, HLA-A, HLCS, IFNG, KLRD1, KIF22, LAG3, LTK, SH2D1A, MAN2C1, MEN1, MAP3K10, MSH3, NKG7,

		PDCD1, PLCG1, PMS1, POLG, PPP2R5C, PRF1, PRKG2, PTGDR, PTPN4, PTPRC, PTPRCAP, PURA, RBL2, RFX1, S100B, XCL1, FBXW4, SLC1A7, TBCC, TCOF1, USP1, ZAP70, ZNF79, ZNF142, ARHGEF5, GPR68, LZTR1, COLQ, GPR65, IKBKAP, CDK10, SLC25A12, RIPK1, RGS9, DDX18, PSTPIP1, ARHGEF1, GPR52, OTOF, GRAP2, CTR9, CROCC, SART3, USP34, URB2, ZBTB39, DLEC1, ACTR1B, ARFRP1, KLRG1, CD96, STUB1, NMUR1, HMGN4, CXCR6, SRCAP, CD160, CAPN10, CEP250, IKZF3, AAK1, ZBTB1, SCAP, MDN1, CSTF2T, ADAT1, MTO1, FBXO3, TRMT2A, RPUSD2, VPS4A, GPKOW, SIT1, ASTE1, ANAPC2, GPR171, PNMA3, TBX21, COL5A3, ZNF639, LIPT1, PRMT7, KLHDC4, ALKBH4, CNNM2, DPP8, C2orf42, USP47, ELP3, KLHL11, IMP3, GIMAP4, CDKN2AIP, URGCP, LTB4R2, INPP5E, PRDM8, TULP4, PLXDC1, CHD8, CREBZF, ZNF335, RNF25, PAPOLG, KRI1, AHNAK, C7orf26, PVRIG, C1orf35, FYCO1, GCC1, CORO7, GSDMD, MUS81, WDR82, SLC38A1, SPSB3, ZNF276, PWWP2A, OSBPL7, MRFAP1L1, DNAJC24, ZNF428, PYHIN1, ZNF549, GIMAP6
CD8_Tem_HPCA_2___113	Aran_et_al_2017	ABCD2, FASLG, ATR, BMPR1A, C8G, C21orf2, CACNB1, CAPN2, CASP8, CD2, CD3D, CD3E, CD3G, CD8A, CD8B, CCR5, CTBP1, CX3CR1, DUSP8, E4F1, ELK4, ERN1, FLT4, GIPR, GOLGA4, CXCR3, GTF3C1, GZMH, GZMA, GZMB, GZMK, GZMM, HLA-A, HLCS, IFNG, IL12RB1, IRF3, ITGAL, KLRB1, KLRD1, LAG3, LTK, SH2D1A, MAN2C1, MEN1, MAP3K10, NKG7, PDCD1, PLCG1, POLG, PPP2R5C, PRF1, PTGDR, PTPN4, PTPRC, PTPRCAP, PURA, RBL2, RFX1, SBF1, XCL1, FBXW4, SLAMF1, SLC1A7, TBCC, TCOF1, UBTF, ZAP70, ZNF142, ARHGEF5, GPR68, LZTR1, COLQ, GPR65, IKBKAP, CDC14A, RIPK1, RGS9, IL18RAP, PSTPIP1, ARHGEF1, GPR52, OTOF, GRAP2, NCR1, CTR9, CROCC, URB2, ZBTB39, SGSM2, DLEC1, ACTR1B, ARFRP1, KLRG1, CD96, STUB1, NMUR1, HMGN4, STK25, GNLY, CXCR6, DIDO1, CD160, CAPN10, CEP250, IKZF3, AAK1, SACM1L, MDN1, CSTF2T, ADAT1, TRMT2A, RPUSD2, VPS4A, SIT1, ASTE1, ANAPC2, GPR171, PNMA3, TBX21, COL5A3, ZNF639, KLHDC4, TTC22, KLHL11, PANK4, GIMAP4, CDKN2AIP, RNF126, TRMU, EXOC2, LTB4R2, OTUD7B, PRDM8, TULP4, PLXDC1, CREBZF, HIVEP3, ZNF335, KRI1, PVRIG, FYCO1, GCC1, CORO7, FBXO31, GSDMD, ZNF696, MUS81, WDR82, TRAF3IP3, SLC38A1, KIAA1109, RHOT2, ANGEL2, SPSB3, ZNF276, OSBPL7, MRFAP1L1, DNAJC24, ZNF428, PYHIN1, NCR3
CD8_Tem_HPCA_3___114	Aran_et_al_2017	ABCD2, BMPR1A, C8G, CACNB1, CASP8, CD8A, DUSP8, GZMH, IFNG, LAG3, LTK, SH2D1A, MSH3, PDCD1, PPP2R5C, PURA, S100B, XCL1, FBXW4, TBCC, TCOF1, ARHGEF5, CDK10, SLC25A12, KLRG1, HMGN4, CXCR6, CEP250, IKZF3, ADAT1, RPUSD2, ASTE1, GPR171, COL5A3, GIMAP4, PVRIG, FYCO1, GCC1, MRFAP1L1, ZNF428, GIMAP6
CD8_Tem_NOVERSHTERN_1___115	Aran_et_al_2017	FASLG, C8G, CACNB1, CD8A, CD8B, CX3CR1, GZMH, GZMB, GZMK, IFNG, LAG3, LTK, SH2D1A, PDCD1, PRF1, PTGDR, RBL2, SLC1A7, ZAP70, KLRG1, NMUR1, CXCR6, TBX21, COL5A3, PYHIN1
CD8_Tem_NOVERSHTERN_2___116	Aran_et_al_2017	FASLG, C8G, CD8A, CD8B, CX3CR1, GZMH, GZMB, GZMK, IFNG, PDCD1, PRF1, PTGDR, SLC1A7, ZAP70, KLRG1, NMUR1, CXCR6, TBX21, COL5A3, PYHIN1
CD8_Tem_NOVERSHTERN_3___117	Aran_et_al_2017	FASLG, C8G, CACNB1, CD8A, CD8B, CX3CR1, GZMH, GZMB, GZMK, IFNG, SH2D1A, PDCD1, PRF1, PTGDR, SLC1A7, ZAP70, KLRG1, NMUR1, CXCR6, TBX21, COL5A3, PYHIN1
cDC_HPCA_1___118	Aran_et_al_2017	ALCAM, ANXA1, CD1C, CD1E, DBI, FCER1A, ITGAX, PITPNA, SSR1, RAB7A, CLEC10A, TCTN3, CCDC88A, SLAMF8
cDC_HPCA_2___119	Aran_et_al_2017	ALCAM, CD1C, CD1E, DBI, FCER1A, ITGAX, SSR1, RAB7A, ACTR3, CLEC10A, CCDC88A, SLAMF8
cDC_HPCA_3___120	Aran_et_al_2017	CD1C, CD86, FCER1A, FLT3, S100A10, CD163, CLEC10A, FGL2, CD93, WDFY3, CLEC4A
cDC_NOVERSHTERN_1___121	Aran_et_al_2017	CD1A, CD1B, CD1C, CD1E, CD80, DNASE1L3, FCER1A, GFRA2, CCL17, CCL24, RRP1B, CD209, KCNK13
cDC_NOVERSHTERN_2___122	Aran_et_al_2017	CD1A, CD1B, CD1C, CD1E, CD80, DNASE1L3, FCER1A, GFRA2, CCL17, CCL24, CD209, KCNK13
cDC_NOVERSHTERN_3___123	Aran_et_al_2017	ALOX15, CD1A, CD1B, CD1C, CD1E, CD80, CD86, CRH, DNASE1L3, FCER1A, GFRA2, CCL13, CCL17, CCL23, CCL24, ALDH1A2, CLEC10A, RRP1B, CD209, KCNK13
Chondrocytes_ENCODE_1___124	Aran_et_al_2017	ACAN, ARL1, COMP, ERG, ISLR, OGN, PGK1, PRELP, COL14A1, RGS11, TBX4, MORF4L2, LAPTM4A, SNUPN, MPHOSPH6, SCRG1, FBXW11, REM1, ZNF471, PODNL1
Chondrocytes_ENCODE_2___125	Aran_et_al_2017	ACAN, ARL1, COMP, ERG, GLG1, ISLR, OGN, PGK1, PRELP, COL14A1, PTP4A2, RGS11, TBX4, GOSR2, MORF4L2, LAPTM4A, SNUPN, MPHOSPH6, SCRG1, FBXW11, REM1, ZNF471, PODNL1

Chondrocytes_ENCODE_3___126	Aran_et_al_2017	ACAN, CCNB1, COL10A1, COMP, CSNK1A1, ERG, FOXD2, LPAR4, ISLR, MYOC, NFATC4, NKX3-1, OMD, PRELP, STAT2, TNXB, COL14A1, PTP4A2, GDF5, FZD9, CILP, TNFSF11, WASL, TBX4, TMEM59, MORF4L2, LAPTM4A, SNUPN, MPHOSPH6, PRG4, ANGPTL7, SLC38A3, DSTN, WWP2, SCRG1, IQSEC2, FBXW11, HSPB7, REM1, COL5A3, CRTAC1, NPLOC4, ZNF471, PODNL1, NDFIP1, DYNLRB1, LRRC15
Chondrocytes_FANTOM_1___127	Aran_et_al_2017	ADRA1D, ACAN, AK1, ABCD1, APOC3, ARCN1, ARL1, ARNT, CACNA1C, CACNB1, CAMLG, RUNX2, RUNX1, COL10A1, COMP, COPA, COPB1, CYP19A1, DMWD, DPT, DVL1, ELN, ENO1, ERF, ETF1, FGF7, FOXC2, FSHB, GOLGA4, MCHR1, GRIA3, HAS1, HDLBP, HOXD3, IBSP, IDUA, IFNB1, IGF1, ISLR, KRT10, LEP, LTBR, SMAD5, MIF, MLN, MLLT1, NFATC4, NFKBIL1, OCRL, OMD, OGN, P4HB, PCDHGC3, PDE3A, PFN2, PGM1, PRELP, PRL, PYY, PTH1R, PTPN11, RAB3A, RGR, ROS1, S100A6, SGCD, SNTB2, SSX1, TACR3, TF, CLEC3B, TNNT3, TUB, COL14A1, ZFPL1, ZBTB16, CDK2AP1, GDF5, CILP, PRKRA, MYH13, WISP1, SPAG9, MAP3K6, OTOF, CABP1, TMEM59, BAG3, NCOR2, TTC37, LAPTM4A, CUL7, PJA2, NAALADL1, RANBP9, ABCC9, ABI2, PRG4, ANGPTL7, CALCOCO2, YAP1, CDIPT, TM9SF1, PRDX4, TRIM3, CORIN, ZMYND11, NCKAP1, CCL27, YIF1A, COPS8, SPIN1, KDELR1, SERINC3, OS9, TMED10, EMILIN1, MAP4K5, NXPH3, SCRG1, FNDC3A, TBC1D9B, ERC1, MAST2, SNX13, PHLDB1, GANAB, SCFD1, POFUT2, KIAA0368, MKRN2, EID1, BCL2L13, TMEM59L, SNED1, GORASP2, SLC13A4, RNF11, HSPB7, TUBG2, IL17B, KLF15, CNIH4, GMPPA, SEC61A1, POMT2, NRBF2, COL5A3, TMED7, CELA2B, NGRN, CMPK1, MIOS, MIER2, SLC41A3, WDR41, KLHL26, OLAH, SPATA7, GLT8D1, IRGC, IFT46, OTUD7B, ZNF471, RIC8A, ZFAND3, PKNOX2, NEUROG2, ACBD3, TTC23, SLC26A10, C11orf95, YIPF2, FTO, PODNL1, HDAC11, TCEAL4, ADM2, SVEP1, UBXN6, TM2D1, TSSK1B, IL17RC, TBC1D16, LRRC15, PRRC1, ZNF358, 44084, SLC5A12, NPHP4, PPIL6, DPY19L4, CTRB2
Chondrocytes_FANTOM_2___128	Aran_et_al_2017	ADRA1D, ACAN, AK1, APOC3, ARNT, CACNA1C, CAMLG, RUNX2, RUNX1, COL10A1, COMP, COPA, CYP19A1, DPT, DVL1, ELN, ERF, FGF7, GRIA3, HAS1, HDLBP, IBSP, IFNB1, ISLR, MIF, MLLT1, NFATC4, NFKBIL1, OMD, OGN, P4HB, PCDHGC3, PRELP, PYY, PTH1R, CLEC3B, TUB, COL14A1, ZFPL1, ZBTB16, GDF5, CILP, PRKRA, WISP1, MAP3K6, CABP1, BAG3, TTC37, LAPTM4A, CUL7, PJA2, ABCC9, PRG4, ANGPTL7, CALCOCO2, CDIPT, TM9SF1, CORIN, ZMYND11, NCKAP1, YIF1A, COPS8, SPIN1, KDELR1, TMED10, EMILIN1, MAP4K5, NXPH3, SCRG1, PHLDB1, GANAB, MKRN2, EID1, BCL2L13, TMEM59L, SNED1, HSPB7, TUBG2, IL17B, POMT2, COL5A3, TMED7, WDR41, KLHL26, OLAH, SPATA7, GLT8D1, IFT46, ZNF471, RIC8A, ZFAND3, PKNOX2, TTC23, C11orf95, YIPF2, FTO, PODNL1, TCEAL4, ADM2, SVEP1, UBXN6, IL17RC, LRRC15, PRRC1, ZNF358, 44084, NPHP4
Chondrocytes_FANTOM_3___129	Aran_et_al_2017	ADRA1D, ACAN, AK1, ARL1, CACNA1C, CAMLG, RUNX1, COL10A1, COMP, CYP19A1, DPT, DVL1, ELN, ENO1, ERF, FGF7, FSHB, GOLGA4, MCHR1, GRIA3, HAS1, HDLBP, IBSP, IDUA, IGF1, ISLR, KRT10, LEP, MLLT1, NFATC4, NFKBIL1, OMD, OGN, P4HB, PDE3A, PGM1, PRELP, PYY, SGCD, SNTB2, TF, CLEC3B, TNNT3, COL14A1, ZFPL1, ZBTB16, CDK2AP1, GDF5, CILP, WISP1, MAP3K6, BAG3, TTC37, LAPTM4A, CUL7, PJA2, ABCC9, PRG4, ANGPTL7, CALCOCO2, YAP1, CDIPT, CORIN, NCKAP1, COPS8, KDELR1, SERINC3, OS9, TMED10, EMILIN1, MAP4K5, NXPH3, SCRG1, FNDC3A, SNX13, PHLDB1, GANAB, MKRN2, EID1, BCL2L13, TMEM59L, SNED1, RNF11, HSPB7, TUBG2, KLF15, SEC61A1, POMT2, NRBF2, COL5A3, WDR41, KLHL26, OLAH, SPATA7, GLT8D1, PKNOX2, C11orf95, YIPF2, PODNL1, HDAC11, TCEAL4, UBXN6, IL17RC, LRRC15, PRRC1, ZNF358, 44084, NPHP4
Chondrocytes_HPCA_1___130	Aran_et_al_2017	ACAN, COMP, CSNK1A1, ERG, ISLR, NFATC4, PRELP, COL14A1, TBX4, MORF4L2, MPHOSPH6, DSTN, SCRG1, HSPB7, REM1, PODNL1
Chondrocytes_HPCA_2___131	Aran_et_al_2017	ACAN, COMP, ERG, ISLR, LBP, PRELP, COL14A1, TBX4, MPHOSPH6, SCRG1, HSPB7, REM1
Chondrocytes_HPCA_3___132	Aran_et_al_2017	ACAN, COMP, ERG, ISLR, PRELP, COL14A1, RGS11, TBX4, MPHOSPH6, SCRG1, REM1
Classswitched_memory_Bcells_BLUEPRINT_1___133	Aran_et_al_2017	TNFRSF17, BLK, CR1, EPS15, RAPGEF1, PTPN6, RAD17, TRAF3, UBE2G1, UBE2I, BAIAP3, DEPDC5
Classswitched_memory_Bcells_BLUEPRINT_2___134	Aran_et_al_2017	TNFRSF17, BLK, CR1, EPS15, RAPGEF1, NDUFA9, PTPN6, RAD17, SLC12A3, TAF6, TRAF3, UBE2G1, UBE2I, UBE2N, TRRAP, BAIAP3, DEPDC5, SCRN1, ABI1, CPSF4, SUB1, SP140, TNFRSF13B, ADAMDEC1, AFTPH, PRDM10, NARFL, PIKFYVE
Classswitched_memory_Bcells_BLUEPRINT_3___13	Aran_et_al_2017	TNFRSF17, BLK, CR1, EPS15, RAPGEF1, NDUFA9, PTPN6, RAD17, TRAF3, UBE2G1, UBE2I, BAIAP3, DEPDC5, ABI1, SEC24A, TNFRSF13B, AFTPH, NGLY1, PRDM10,

5		PIKFYVE
Classswitched_memory_Bcells_NOVERSHTERN_1___136	Aran_et_al_2017	TNFRSF17, BLK, CXCR5, MS4A1, CD80, COL19A1, GPR25, HLA-DQB2, PAX5, SPIB, BAIAP3, TNFRSF13B, SNED1, ZBTB32, FCRL2, KHDRBS2
Classswitched_memory_Bcells_NOVERSHTERN_2___137	Aran_et_al_2017	BLK, CXCR5, MS4A1, CD80, COL19A1, GPR25, HLA-DQB2, PAX5, SPIB, BAIAP3, TNFRSF13B, SNED1, ZBTB32, FCRL2
Classswitched_memory_Bcells_NOVERSHTERN_3___138	Aran_et_al_2017	BLK, CXCR5, MS4A1, CD80, COL19A1, GPR25, HLA-DQB2, PAX5, SPIB, BAIAP3, TNFRSF13B, SNED1, ZBTB32, FCRL2
CLP_BLUEPRINT_1___139	Aran_et_al_2017	COX6C, DNTT, IGLL1, PLP2, PSMA6, VPREB1, AIMP1, TOMM20, GPN3, HIVEP3, MYL12B
CLP_BLUEPRINT_2___140	Aran_et_al_2017	COX6C, DNTT, H3F3B, IDH3A, IGLL1, OXA1L, PLP2, PSMA6, SNRPD1, VPREB1, AIMP1, ADNP, C19orf53, GPN3, C11orf57, HIVEP3, FAM76A
CLP_BLUEPRINT_3___141	Aran_et_al_2017	CALM1, COX6C, DNTT, GAPDH, H3F3B, IDH3A, IGBP1, IGLL1, OXA1L, PLP2, PSMA6, PSMB3, RFC4, RPL8, SNRPD1, VPREB1, AIMP1, ATP6V1G1, TOMM20, METAP2, DSTN, ADNP, C19orf53, ASCC1, GPN3, C11orf57, WDR33, NGLY1, HIVEP3, MYL12B, FAM76A
CMP_BLUEPRINT_1___142	Aran_et_al_2017	AZU1, MS4A3, CPA3, CRHBP, CRYGD, CTSG, ELANE, MS4A2, IGLL1, MPO, SERPINB10, PRG2, PRTN3, RNASE2, RNASE3, STAR, EPX, NAALADL1
CMP_BLUEPRINT_2___143	Aran_et_al_2017	AZU1, MS4A3, CLC, CPA3, CRHBP, CTSG, ELANE, MS4A2, FLT3, HDC, MPO, SERPINB10, PRG2, PRTN3, RNASE2, RNASE3, EPX, NAALADL1, HPGDS
CMP_BLUEPRINT_3___144	Aran_et_al_2017	AZU1, MS4A3, CLC, CPA3, CRHBP, CTSG, ELANE, FLT3, MPO, PRG2, PRTN3, RNASE2, RNASE3, EPX, HPGDS
CMP_HPCA_1___145	Aran_et_al_2017	ALOX15, ANXA3, AZU1, BYSL, C1QB, CACNA1E, CAMK2A, RUNX1, CCNC, CD1E, MS4A3, CD63, CDSN, CLC, CLIC1, CNGA3, CPA3, CPB1, CPN1, CRYGA, CSF2RB, CTSG, DAB1, DEFA6, EDN3, EYA3, MS4A2, FMO2, AFF2, G6PC, GALR1, GDF9, GNAT1, GPD1, GRIA4, GRM2, GUCA2B, H2AFX, HDC, HSF4, IDUA, IL5RA, IL13, IMPG1, PDX1, ITIH3, ITIH4, KCNJ10, KCNN1, LCT, MEA1, MKI67, MPL, MPO, MYF5, MYH11, NDUFB4, OXT, P2RY4, PAX1, PAX3, PGR, SERPINB10, SERPINI2, PRG2, PRKG2, PYY, PTPRS, RCVRN, RHO, RORB, SCN1A, CCL18, CCL23, SIM1, SLC5A2, SLC18A2, SNAPC4, STAR, TNR, TP73, TRPC3, TYR, COL14A1, UNG, VPREB1, ZNF174, MADCAM1, CLPP, GDF5, HIST1H2BO, PLA2G6, BFSP2, IRS4, LIPF, BRSK2, SLC13A2, CCNB2, LONP1, NTN1, FHL5, FRMPD4, DLEC1, NAALADL1, CHAF1A, GLYAT, TRAIP, CACNG3, TUBA1B, PRG3, TAB1, ERLIN1, SPAG5, RASL10A, NEU3, RUVBL2, ACTL7A, PTPRT, SNW1, FAIM2, POLA2, IRF2BP1, DAZAP1, DNAI1, FOXB1, SNX5, NDOR1, GNMT, CNTN6, HPGDS, MYLPF, SMARCAL1, UBQLN3, RASL12, TOLLIP, MED18, COMMD4, CRTAC1, KLHL11, DHX32, SPATA7, CENPN, ZMAT5, C21orf62, CYSLTR2, VN1R1, SLC4A5, CADM3, PRODH2, DNASE2B, ALX4, XPNPEP3, DPEP3, GPR135, PDIA2, CYP3A43, LRRC19, C22orf46, LIN28A, ZBBX, RERGL, L2HGDH, TSGA10, LRRC3, NRIP2, COG7, TIMM50, TBC1D16, ZNF428, RAB40A, FAM76A, ATXN7L1, ANKRD34C
CMP_HPCA_2___146	Aran_et_al_2017	ADSS, MS4A3, MAPK14, TOR1A, FANCG, MS4A2, LPAR4, GPR27, LTC4S, LYL1, MPO, MYO9A, PGM1, POLE, MAP2K5, TRIM27, RNASE2, RREB1, SPN, STAR, TFCP2, TOP2B, TPSAB1, WHSC1, XPO1, ZNF221, HIST1H2BL, HIST1H2BO, HIST1H3C, HIST1H4C, NSMAF, JRK, ZMYM4, TGM5, SCAMP1, HDAC6, ZNF197, ERLIN1, TAF6L, USP19, EHMT2, HMGXB3, SMC5, MGA, KIAA1033, ZNF629, TNPO3, ZKSCAN5, ZZZ3, SPAG8, REV1, PHF7, VPS54, EXD3, SETD5, RHOT1, ZNF701, CENPJ, KLHL9, ZNF471, BAHCC1, HRH4, FAM111A, NARFL, S100PBP, MTHFSD, ZNF747, CCDC121, ZNF768, ATP8B4, ZKSCAN3, TSGA10, CDT1, USP48, ARHGAP33, ZNF780B, PIKFYVE, ZKSCAN4, ZNF324B
CMP_HPCA_3___147	Aran_et_al_2017	MS4A3, MS4A2, GPR27, LTC4S, LYL1, MPO, STAR, TPSAB1, ZNF221, HIST1H2BO, HIST1H3C, NAT6, CENPJ, BAHCC1
CMP_NOVERSHTERN_1___148	Aran_et_al_2017	MS4A3, CD33, TOR1A, LPAR4, GPR27, MPO, TFCP2, TPSAB1, HIST1H4C, ZMYM4, TGM5, HDAC6, ZNF197, ERLIN1, TNPO3, NAT6, ZZZ3, SPAG8, FBXL4, ZNF701, CENPJ, ZNF471, S100PBP, ZKSCAN3, TSGA10, CDT1, ATP8B3, ZNF324B
CMP_NOVERSHTERN_2___149	Aran_et_al_2017	TOR1A, LPAR4, GPR27, MPO, ZMYM4, TGM5, HDAC6, ZNF197, ERLIN1, TNPO3, SPAG8, ZNF701, ZNF471, ZKSCAN3
CMP_NOVERSHTERN_3___150	Aran_et_al_2017	MS4A2, LPAR4, GPR27, LTC4S, LYL1, MPO, TRIM27, STAR, TPSAB1, ZNF221, HIST1H2BO, HIST1H3C, ZNF197, ZNF629, SPAG8, PHF7, CENPJ, BAHCC1, ATP8B4,

		ZKSCAN3
DC_BLUEPRINT_1___151	Aran_et_al_2017	ALOX15, CD1A, CD1B, CD1E, CCL13, CCL17, ALDH1A2, CD209
DC_BLUEPRINT_2___152	Aran_et_al_2017	ALOX15, CD1A, CD1B, CD1E, HLA-DQA1, CCL13, CCL17, ALDH1A2, CD209
DC_BLUEPRINT_3___153	Aran_et_al_2017	ALOX15, CD1A, CD1B, CD1E, FPR3, CCL13, CCL17, CD209
DC_FANTOM_1___154	Aran_et_al_2017	C1QA, C1QB, CD1A, CD1B, CD1E, CD9, FPR3, CCL13, CCL17, CCL22, CLEC10A, TFEC, TREM2, SLAMF8
DC_FANTOM_2___155	Aran_et_al_2017	ACHE, ALOX15B, CD1A, CD1B, CD1E, CD80, CD86, CCR7, DPYS, ETV3, GRIN1, GRSF1, HCRTR2, IL12B, IRF4, KCNC3, KCNN1, LOR, MCF2, RAB8A, NFKB1, PLD2, PRRG2, PTGIR, RNF2, CCL13, CCL17, CCL18, CCL22, CCL23, SLAMF1, SIGLEC1, TRAF1, TNFRSF4, TXN, VAV2, SLC30A4, CUL1, MAP3K6, TMSB10, MAP3K13, CEP350, BCL2L11, MPHOSPH6, SPINT2, HPS5, NXPH3, TDRD7, TMEM131, SUZ12, BCL2L13, FBXL4, SNX11, IL21R, TBC1D13, ARL8B, NECAP2, CAMK1G, CCDC81, SAMSN1, UBE2Z, PTGES2, SLCO5A1
DC_FANTOM_3___156	Aran_et_al_2017	ALCAM, C1QA, C1QB, CD1A, CD1B, CD1C, CD1E, F13A1, FCER2, FPR3, TACSTD2, CCL13, CCL17, CCL22, CLEC10A, SPINT2, STAB1, CD209, TREM2, SLAMF8, MS4A6A
DC_HPCA_1___157	Aran_et_al_2017	ALOX15, CD1B, CD1E, CD80, CCL13, CCL17, CCL18, CCL19, CCL22, CD209, SLCO5A1
DC_HPCA_2___158	Aran_et_al_2017	ALOX15, CD1B, CD1E, CD80, CCL13, CCL17, CCL18, CCL19, CCL22, CD209, SLCO5A1
DC_HPCA_3___159	Aran_et_al_2017	ALOX15, CD1B, CD1E, CCL13, CCL17, CCL18, CCL19, SLCO5A1
DC_IRIS_1___160	Aran_et_al_2017	CD1B, CD1E, CD86, FCER2, CCL17, ALDH1A2, RRP1B, KCNK13
DC_IRIS_2___161	Aran_et_al_2017	ALOX15, C1QB, CD1A, CD1B, CD1E, CD80, CD86, DNASE1L3, F13A1, FCER2, FPR3, GUCA1A, HK3, HLA-DQA1, CCL8, CCL13, CCL17, CCL18, CCL22, CCL23, CCL24, ALDH1A2, HS3ST2, CLEC10A, SPINT2, FGL2, CD209, NAGPA, MS4A4A, KCNK13, MS4A6A
DC_IRIS_3___162	Aran_et_al_2017	ALOX15, C1QB, CD1A, CD1B, CD1E, CD86, FPR3, HLA-DQA1, CCL13, CCL17, CCL18, CCL22, CCL23, ALDH1A2, HS3ST2, CLEC10A, CD209
Endothelial_cells_BLUEPRINT_1___163	Aran_et_al_2017	ACVRL1, TIE1, VWF, HYAL2, ARHGEF15, ROBO4, MMRN2, FAM124B
Endothelial_cells_BLUEPRINT_2___164	Aran_et_al_2017	ACVRL1, TIE1, VWF, HYAL2, ARHGEF15, ROBO4, MMRN2, FAM124B
Endothelial_cells_BLUEPRINT_3___165	Aran_et_al_2017	ACVRL1, ADSS, AP2A2, ANGPT2, ANXA2, ANXA3, RHOC, BMX, PTTG1IP, CANX, CAV1, CCT6A, CD9, CDC27, AP2S1, CLTA, DAD1, DDX10, EIF4G2, ERG, FOXC2, FLT1, FLT4, GJA4, GNB1, GOT2, GPR4, HSPA4, HTR2B, KDR, TNPO1, MNAT1, MYL6, NEDD8, NNAT, NOTCH4, PNP, PDE3A, PIK3C2A, PLS3, PPP2R2A, PSMB7, PSMD1, PSMD2, PSMD10, RALA, RANGAP1, RARS, RCN2, S100A6, MAPK12, SELE, SH3GL1, SLC16A1, SNTB2, SSBP1, TARBP2, TEK, TIE1, TJP1, CLDN5, TPD52L2, HSP90B1, UFD1L, VWF, FXR1, SCARF1, DYNLL1, HYAL2, CDC123, EIF2B2, MTMR2, BCL10, TNFSF18, ITGB1BP1, TAOK2, EI24, GDF3, FEZ2, PPM1F, SAE1, KIF20A, ACTR1A, PDIA6, TXNDC9, PCGF3, TIMM17A, CARM1, SEMA6B, IPO7, PITRM1, ARPC1A, IGF2BP3, YKT6, LYVE1, COPS6, PWP1, CDC37, FAM107A, ECD, TUSC2, ARHGEF15, MMRN1, NCBP2, CD93, ATF6, TTLL5, STAB1, ARL2BP, MTCH1, TMEM184B, CLEC1A, BFAR, PCDH12, SPTBN5, EMCN, BTBD1, SOX18, ROBO4, TMED9, DEF8, RASIP1, TMEM39B, LRRC59, NPLOC4, CISD1, KLHL9, ANO2, MRPL17, SPATS2, CXorf36, MMRN2, FAM124B, EDC3, MYCT1, NETO2, PLVAP, DCTN5, G6PC3, MYL12B, KANK3
Endothelial_cells_ENCODE_1___166	Aran_et_al_2017	ACVRL1, ANGPT2, ANXA2, RHOC, BMX, PTTG1IP, CAV1, CLTA, DAD1, ERG, FOXC2, GPR4, KDR, MYL6, PIK3C2A, PLS3, PSMD10, RALA, MAPK12, SLC16A1, TEK, TIE1, CLDN5, VWF, HYAL2, FEZ2, ACTR1A, TXNDC9, PCGF3, COPS6, PWP1, FAM107A, ECD, ARHGEF15, MMRN1, CD93, TTLL5, MTCH1, CLEC1A, PCDH12, EMCN, SOX18, ROBO4, RASIP1, LRRC59, MRPL17, CXorf36, MMRN2, MYCT1, PLVAP, KANK3
Endothelial_cells_ENCODE_2___167	Aran_et_al_2017	ACVRL1, ANGPT2, ANXA2, RHOC, BMX, PTTG1IP, CAV1, CLTA, ERG, GPR4, KDR, PLS3, PSMD10, RALA, MAPK12, SLC16A1, TIE1, CLDN5, VWF, HYAL2, ACTR1A, PCGF3, COPS6, PWP1, FAM107A, ARHGEF15, MMRN1, CD93, TTLL5, MTCH1, CLEC1A, PCDH12, EMCN, SOX18, ROBO4, RASIP1, MRPL17, CXorf36, MMRN2, MYCT1, PLVAP, KANK3

Endothelial_cells_ENCODE_3___168	Aran_et_al_2017	ACVRL1, ANGPT2, RHOC, BMX, CLTA, GPR4, KDR, PLS3, PSMD10, RALA, MAPK12, TIE1, VWF, HYAL2, ACTR1A, ARHGEF15, CLEC1A, EMCN, SOX18, ROBO4, RASIP1, CXorf36, MMRN2, MYCT1, KANK3
Endothelial_cells_FANTOM_1___169	Aran_et_al_2017	ACVRL1, ANGPT2, KDR, TIE1, HYAL2, ARHGEF15, EMCN, ROBO4, MMRN2, MYCT1
Endothelial_cells_FANTOM_2___170	Aran_et_al_2017	ACVRL1, ANGPT2, BMX, KDR, TIE1, HYAL2, ARHGEF15, EMCN, ROBO4, MMRN2, MYCT1
Endothelial_cells_FANTOM_3___171	Aran_et_al_2017	ACVRL1, ANGPT2, ANXA3, ART4, BMX, CAV1, ERG, FLT1, GJA4, GPR4, HTR2B, KDR, KRT19, TACSTD2, PDE3A, RALA, MAPK12, SELE, SNTB2, TIE1, CLDN5, VWF, HYAL2, EIF2B2, TNFSF18, GDF3, IGF2BP3, ARHGEF15, MMRN1, CD93, CLEC1A, EMCN, SOX18, ROBO4, RASIP1, CXorf36, MMRN2, FAM124B, MYCT1, KANK3
Endothelial_cells_HPCA_1___172	Aran_et_al_2017	ANGPT2, BMX, KDR, TIE1, CLDN5, VWF, MMRN1, CD93, STAB1, EMCN, ROBO4, GIMAP4
Endothelial_cells_HPCA_2___173	Aran_et_al_2017	ANGPT2, BMX, FLT4, KDR, TIE1, VWF, SEMA6B, MMRN1
Endothelial_cells_HPCA_3___174	Aran_et_al_2017	ACVRL1, CDC27, AP2S1, ERCC1, FBL, FDPS, FOXC2, FLT4, GJA4, GPR4, HTR1B, LYL1, NOTCH4, NOVA2, PNP, PPP2R2A, PSMC5, RALA, RELA, RPS14, MAPK12, TIE1, CLDN5, VWF, CLPP, SCARF1, HYAL2, EIF2B2, MTMR2, BCL10, TNFSF18, BUB3, TAOK2, DLGAP5, ACTR1A, LYPLA1, STK25, SEMA6C, SEMA6B, PTTG2, WDR4, LYVE1, STRAP, TUSC2, ARHGEF15, CD93, TTLL5, N4BP3, STAB1, EDC4, PRKD2, PRPF19, GIT1, CLEC1A, ROBO4, TMEM39B, CEP55, NPLOC4, CISD1, METTL3, TUT1, SPATS2, FAM65A, MMRN2, FAM124B, MYCT1, MTG1, KANK3, GIMAP6
Eosinophils_BLUEPRINT_1___175	Aran_et_al_2017	C3AR1, CLC, CCR3, DRP2, IL5RA, KCNA5, KIF5A, TACSTD2, NPY2R, PLXNB3, RGS13, TNFSF11, ADAM18, CUX2, CDH19, RASL12, HRH4, NYX
Eosinophils_BLUEPRINT_2___176	Aran_et_al_2017	CCR3, DRP2, GIPR, TACSTD2, RGS13, SYCP1, PHLDA2, TNFSF11, NXPH3, LMTK2, AP4E1, FBXO40, HRH4, TRIM48, MMRN2
Eosinophils_BLUEPRINT_3___177	Aran_et_al_2017	AGTR2, ASPA, ATOH1, BMX, C3AR1, MS4A3, CLC, CCR1, CCR3, DRP2, FCER1A, FMO3, GAST, GIPR, HDC, IL5RA, KCNA5, KIF5A, LECT2, TACSTD2, MOG, NPY2R, PLXNB3, PRL, RGS13, STATH, TNFSF11, ADAM21, ADAM18, HS3ST2, ZNF197, POLQ, NXPH3, LMTK2, CUX2, CDH19, PURG, RASL12, FBXO40, MYO3A, FEV, LRRC36, MMP26, HRH4, NYX, TRIM48
Eosinophils_FANTOM_1___178	Aran_et_al_2017	ADORA3, ALOX15, CA4, ENTPD2, CEBPE, CEACAM8, CLC, CCR3, DEFA4, GIPR, HIC1, IL5RA, LTF, MNT, RARA, SLC19A1, KSR1, PGLYRP1, CD101, P2RY14, KCNK7, OLIG2, PTTG2, EPN2, SETD1B, KDM6B, ABTB2, SIGLEC8, GMIP, CYSLTR2, MKL1, HRH4, DPEP2, MMP25, MBOAT7, CORO7, ARHGAP33, TRPM6
Eosinophils_FANTOM_2___179	Aran_et_al_2017	ADORA3, ALOX15, CA4, ENTPD2, CEBPE, CLC, CCR3, DEFA4, GIPR, IL5RA, LTF, MNT, RARA, SLC19A1, KSR1, PGLYRP1, KCNK7, OLIG2, PTTG2, EPN2, SETD1B, KDM6B, ABTB2, SIGLEC8, GMIP, CYSLTR2, MKL1, HRH4, DPEP2, MMP25, MBOAT7, CORO7, ARHGAP33, TRPM6
Eosinophils_FANTOM_3___180	Aran_et_al_2017	ADORA3, ALOX15, BPI, CA4, CAMP, ENTPD2, CEBPE, CEACAM8, CLC, CCR3, CSF2RB, DEFA4, GIPR, HIC1, IL5RA, LTF, MNT, RARA, CCL23, SLC19A1, KSR1, PGLYRP1, CD101, P2RY14, KCNK7, OLIG2, PTTG2, EPN2, SETD1B, KDM6B, SRRM2, ABTB2, SIGLEC8, GMIP, CYSLTR2, MKL1, HRH4, DPEP2, DPEP3, MMP25, MBOAT7, CORO7, ARHGAP33, TRPM6
Eosinophils_NOVERSHTERN_1___181	Aran_et_al_2017	ALOX15, CEBPE, CLC, CCR3, DEFA4, IL5RA, RARA, KCNK7, EPN2, KDM6B, ABTB2, SIGLEC8, HRH4, DPEP2, MMP25, TRPM6
Eosinophils_NOVERSHTERN_2___182	Aran_et_al_2017	ALOX15, CEBPE, CLC, CCR3, DEFA4, IL5RA, KSR1, ABTB2, SIGLEC8, HRH4, DPEP2, MMP25, TRPM6
Eosinophils_NOVERSHTERN_3___183	Aran_et_al_2017	ENTPD2, CEBPE, CLC, CCR3, GIPR, HIC1, IL5RA, LTF, KSR1, OLIG2, SIGLEC8, HRH4, DPEP2, CORO7, ARHGAP33
Epithelial_cells_ENCODE_1___184	Aran_et_al_2017	SFN, TACSTD2, PRSS8, AP1M2, B3GNT3, CBLC, HES2, RAB25, S100A14
Epithelial_cells_ENCODE_2___185	Aran_et_al_2017	FLNB, SFN, GRB7, LAD1, LAMA5, PRSS8, SEMA3F, SOX15, TUFT1, SLC10A3, IER3, SH2D3A, AP1M2, CNKSR1, B3GNT3, RHOD, HES2, TMEM40, TBC1D2, RAB25, S100A14
Epithelial_cells_ENCODE_3___186	Aran_et_al_2017	BIK, AP1S1, DFNA5, DSG3, HBEGF, EFNB1, NR2F6, EVPL, EXT2, F3, FLNB, GJB3, GJB5, SFN, GRB7, IRF6, JUP, LAD1, LAMA5, LIMK2, TACSTD2, MST1R, PI3, PLAGL2, PRRG2, PRSS8, RELB, SEMA3F, SHC1, SLC12A4, SOX15, SPINT1, ST14, STXBP2, TAPBP, TUFT1, CORO2A, BTG2, SLC10A3, AXIN1, TNK1, TNFSF9, ADAM15,

		TNFRSF10B, IER3, RPS6KA4, PDLIM1, CELSR1, FGFBP1, SH2D3A, AP1M2, CNKSR1, B3GNT3, CDC42EP2, FST, PPP1R13L, ARHGEF18, ETHE1, TNFRSF21, TFCP2L1, RHOD, GLTP, A4GALT, RHOF, HES2, SSH3, TMEM40, TBC1D2, DOK4, FGD6, TMPRSS4, RAB25, SMAGP, S100A14, XYLT2, PORCN, ZFYVE21, CHAC1, C1orf116, ZBED2, RHBDF2, PIP4K2C, FBXL18, LRRC8E, EPPK1
Epithelial_cells_FANTOM_1___187	Aran_et_al_2017	CLDN4, DSC2, SFN, ITGB4, ITGB6, KRT7, LAD1, LAMA3, LAMB3, SPINT1, THBD, SH2D3A, AP1M2, MPZL2, G0S2, LSR, RAB25
Epithelial_cells_FANTOM_2___188	Aran_et_al_2017	ADM, CLDN4, DSC2, EFNA1, EGFR, EVPL, SFN, ITGB4, ITGB6, KRT7, KRT17, LAD1, LAMA3, LAMB3, LLGL2, TACSTD2, PCDH1, PDGFB, PPL, PRSS8, S100A2, SCNN1A, SPINT1, ST14, THBD, HMGA2, PTGES, RAB3D, SH2D3A, AP1M2, TSPAN1, MPZL2, PPP1R13L, RAP1GAP2, DAPP1, G0S2, ANGPTL4, LSR, SLC35F2, EPS8L1, FERMT1, RAB25, C1orf116, RAB11FIP1, ALS2CL
Epithelial_cells_FANTOM_3___189	Aran_et_al_2017	CLDN4, DSC2, ELF3, SFN, ITGB4, ITGB6, KRT6A, KRT7, LAD1, LAMA3, LAMB3, SLPI, ST14, TGFA, SCEL, PTGES, SH2D3A, AP1M2, MPZL2, EHF, G0S2, LSR, S100A14
Epithelial_cells_HPCA_1___190	Aran_et_al_2017	F3, SFN, TACSTD2, PRSS8, SLPI, AP1M2, CBLC, RAB25, S100A14
Epithelial_cells_HPCA_2___191	Aran_et_al_2017	F3, SFN, PRSS8, SLPI, STXBP2, AP1M2, CBLC, RAB25, S100A14
Epithelial_cells_HPCA_3___192	Aran_et_al_2017	NQO1, F3, FLNB, SFN, TACSTD2, PRSS8, SDC4, SLPI, STXBP2, RASSF7, AP1M2, B3GNT3, SH3BP1, CBLC, APOBEC3C, RIPK4, HES2, RAB25, S100A14
Erythrocytes_BLUEPRINT_1___193	Aran_et_al_2017	ALAS2, CA1, EPB42, GYPE, HBD, HMBS, MYL4, RHAG, EPX, KLF1, XPO7, AHSP
Erythrocytes_BLUEPRINT_2___194	Aran_et_al_2017	ALAS2, CA1, EPB42, GYPE, HBD, HMBS, MYL4, RHAG, EPX, KLF1, XPO7, AHSP
Erythrocytes_BLUEPRINT_3___195	Aran_et_al_2017	ALAS2, CA1, EPB42, GYPE, HBD, HMBS, MYL4, RHAG, EPX, KLF1, XPO7, AHSP
Erythrocytes_FANTOM_1___196	Aran_et_al_2017	ALAS2, CA1, EPB42, GYPA, GYPE, KRT1, MYL4, SLC4A1, AHSP
Erythrocytes_FANTOM_2___197	Aran_et_al_2017	ALAS2, EPB42, GYPA, GYPB, GYPE, HBB, HBD, MYL4, PKLR, PRG2, RHAG, RHCE, RHD, SLC4A1, KLF1, AHSP, TSPO2
Erythrocytes_FANTOM_3___198	Aran_et_al_2017	ALAS2, ART4, EPB42, GYPA, GYPB, GYPE, HBB, HBD, HMBS, MYL4, PKLR, RHAG, RHD, KLF1, AHSP
Erythrocytes_HPCA_1___199	Aran_et_al_2017	CENPA, GATA1, RHAG, SPTA1, AURKA, KLF1, GLRX5, AHSP
Erythrocytes_HPCA_2___200	Aran_et_al_2017	ALAS2, CA1, EPB42, GYPE, HBD, HMBS, MYL4, RHAG, EPX, KLF1, XPO7, AHSP
Erythrocytes_HPCA_3___201	Aran_et_al_2017	ALAS2, CA1, EPB42, GYPE, HBD, HMBS, MYL4, RHAG, EPX, KLF1, XPO7, AHSP
Erythrocytes_NOVERSHTERN_1___202	Aran_et_al_2017	APLNR, ALAS2, ATP1B2, BRCA1, BUB1B, CDC20, CDKN3, CENPF, CHEK1, DES, EPB42, FEN1, GYPA, GYPB, GYPE, HBB, HMBS, HMGB2, KEL, KIF22, EPCAM, MCM4, MYL4, NEK2, OAT, PCNA, PKLR, POLE2, PRG2, RHD, RRM1, RRM2, STIL, SLC2A4, SLC4A1, AURKA, TOP2A, TUBG1, ST7, HIST1H4C, RAD54L, CCNB2, MINPP1, DLGAP5, MELK, GINS1, TROAP, TRIM10, RCL1, PRMT3, UBAC1, KLF1, KIF2C, ZWINT, OIP5, PAXIP1, KIF4A, HTRA2, GMNN, NUSAP1, GLRX5, AHSP, DTL, FANCI, HJURP, MCM10, C1orf112, CENPN, KIF15, SPC25, FKBPL, GINS3, CENPO, CDCA3, TSPO2
Erythrocytes_NOVERSHTERN_2___203	Aran_et_al_2017	ALAS2, BRCA1, CENPF, DES, EPB42, FEN1, GYPA, GYPB, GYPE, HBB, HMBS, HMGB2, KIF22, MYL4, NEK2, PCNA, PKLR, POLE2, RHD, RRM1, TOP2A, TUBG1, CCNB2, MINPP1, DLGAP5, MELK, GINS1, TROAP, PRMT3, UBAC1, KLF1, GMNN, NUSAP1, AHSP, DTL, FANCI, MCM10, CENPN, FKBPL, CDCA3
Erythrocytes_NOVERSHTERN_3___204	Aran_et_al_2017	ALAS2, AMHR2, BIRC5, ART4, ATP1B2, BRCA1, BUB1, CA1, CAST, CCNA2, CCNB1, CDK1, CDC20, CDKN3, CENPE, CENPF, CSE1L, DES, EPB42, EPRS, FEN1, GATA1, GYPA, GYPB, GYPE, HBB, HBD, HMBS, HMGB2, HPS1, KEL, KIF22, EPCAM, MYL4, NEK2, PCNA, PKLR, PNMT, POLE2, PRG2, RAD51, RFC4, RHAG, RHD, RPA3, RRM1, RRM2, STIL, SLC2A4, SLC16A1, SPTA1, TAL1, TARS, TOP2A, TUBG1, UMPS, ST7, CHAF1B, GFI1B, HIST1H4C, DPM2, EIF2S2, CCNB2, PTTG1, MINPP1, RGS6, DLGAP5, MELK, GINS1, DCLRE1A, RBX1, TROAP, SMC4, RCL1, PRMT3, UBAC1, SMC2, KLF1, DBF4, METAP2, KIF2C, ZWINT, WBP4, RACGAP1, GMNN, NUSAP1, AHSP, DTL, NCAPG2, CDCA8, FANCI, HJURP, MCM10, CENPN, PBK, SPC25, FKBPL, NUP37, CDCA3
Fibroblasts_ENCODE_1___205	Aran_et_al_2017	ADH5, ARF4, ARL1, ASPA, ATP2A2, BAD, BMPR1A, CACNA1C, AP2M1, CSNK1A1, CSNK1G3, DCTD, DPT, ECT2, ELN, ETF1, FKTN, FGF7, GARS, GOLGA4, GRIA1,

		GRIA3, HIF1A, HLCS, HTR2A, HTR2B, ITIH3, IPO5, KRT19, LGALS1, SMAD5, MARS, MYH1, MYH2, 44076, NFATC4, OCRL, PPIB, PRKG1, PTGIR, RAD23B, RCN2, PRPH2, RYK, ATXN2, SGCD, SGCG, SHMT2, SIM1, SNTB2, TBX5, CLEC3B, TPD52L2, TSPYL1, SLC35A2, VCL, WNT2, ZFPL1, B4GALT2, MPZL1, ZMYM4, ZFYVE9, GGPS1, SCAMP1, BAG2, PRDX6, RNF14, SCRN1, RNF41, TFG, YAP1, SPTLC1, CORIN, FRS2, SPIN1, TMED10, KDELR2, DSTN, EMILIN1, MAP4K5, XPOT, RRAS2, RAB11FIP2, RAB3GAP1, TRIM32, GANAB, DNAJC13, ICMT, LMOD1, MYOF, TBL2, SEC22A, NPTN, HSPB7, SEC61A1, TNFRSF12A, NGRN, MBTPS2, AMOTL2, GPR85, TMED9, ASPN, ST7L, SLC35A5, KIF26B, GPATCH2, IMPACT, POMGNT1, CAND1, ACTR10, SAR1A, THAP10, CCDC90B, MAGEF1, ACBD3, C7orf25, AHNAK, FTO, ZC3H14, PODNL1, TCEAL4, SVEP1, TTC26, SLC25A32, ADAMTS12, TM2D1, ANKRD40, MYL12B, LRRC42, HSPB6, 44084, TOR1AIP2, TXLNA, RNASEH1, DPY19L4, SNX19
Fibroblasts_ENCODE_2___206	Aran_et_al_2017	ARF4, ATP2A2, BMPR1A, CACNA1C, CSNK1A1, CSNK1G3, DPT, ELN, FKTN, FGF7, GRIA1, GRIA3, HIF1A, HTR2B, ITIH3, IPO5, LGALS1, MYH2, 44076, NFATC4, PRKG1, RAD23B, PRPH2, RYK, SGCD, SGCG, SIM1, TBX5, SLC35A2, WNT2, ZFPL1, SCAMP1, BAG2, PRDX6, SCRN1, RNF41, YAP1, SPTLC1, CORIN, FRS2, SPIN1, KDELR2, MAP4K5, XPOT, RRAS2, RAB11FIP2, TRIM32, MYOF, SEC22A, HSPB7, MBTPS2, AMOTL2, GPR85, ASPN, SLC35A5, KIF26B, GPATCH2, CAND1, THAP10, CCDC90B, ACBD3, AHNAK, PODNL1, SVEP1, TM2D1, MYL12B, LRRC42, 44084, TXLNA, RNASEH1, DPY19L4
Fibroblasts_ENCODE_3___207	Aran_et_al_2017	BMPR1A, DPT, ELN, FKTN, FGF7, GRIA3, HTR2B, ISLR, ITIH3, KRT19, MYH2, PRKG1, SGCD, SGCG, SIM1, SNTB2, TBX5, WNT2, BAG2, YAP1, CORIN, TRIM32, LMOD1, MYOF, TNFRSF12A, AMOTL2, ASPN, KIF26B, THAP10, HSPB6, DPY19L4
Fibroblasts_FANTOM_1___208	Aran_et_al_2017	ADD1, ADH1B, ALDH9A1, ANXA11, ARF4, ARHGAP6, C7, CACNA1C, CAMLG, CIRBP, CSF1, CYBA, DPT, DUT, FGF7, FMO2, FTL, GARS, GOLGA1, GSTM5, HEXA, HIC1, HPS1, HTR2B, IDUA, ISLR, JAK3, IPO5, LTBR, LTC4S, SMAD5, MGMT, MGST3, MMP17, MMP19, NFATC4, NFE2L2, P4HB, PDE4A, PFDN5, PGK1, PIK3R2, PRKG2, MASP1, PTGIR, RASA2, RNF4, RNH1, ROM1, RPL37A, SH3BP2, SNAPC2, TADA2A, TBX5, TCF21, TFDP1, CLEC3B, TNXB, VIM, ZNF32, DEK, NDST2, CGGBP1, WISP1, S1PR2, RPL23, HAND2, BAG2, LAPTM4A, HDAC5, MPHOSPH10, TFG, PRDX4, MTHFD2, SEC24A, KDELR1, KDELR2, EMILIN1, LSM6, XPOT, ZBTB1, FAIM2, GANAB, CSTF2T, ABCA6, SNED1, TOR1AIP1, CECR5, RASL12, ZNF771, AMOTL2, HDAC7, UBE2D4, ASPN, TXNL4B, SLC35A5, SHQ1, ADI1, DDX19A, ZNF444, FBXL8, ZNF446, EXOC1, SPATA7, TMEM165, PCDHGA11, GPR137, CASS4, ATP13A1, KIAA1614, EDA2R, PAPPA2, MOSPD3, C11orf95, C7orf25, ATG9A, TBC1D17, SLC35E1, SVEP1, SLC25A32, C6orf62, WDR73, MFSD5, ZNF358, EML3, DPY19L4
Fibroblasts_FANTOM_2___209	Aran_et_al_2017	CAMLG, FTL, GSTM5, ISLR, JAK3, MMP19, NFATC4, PRKG2, PTGIR, S1PR2, HAND2, ZNF771, GPR137, KIAA1614, SPAG16, SVEP1, C6orf120
Fibroblasts_FANTOM_3___210	Aran_et_al_2017	ADH1B, ARHGAP6, C7, DPT, FGF7, FMO2, FTL, HIC1, HTR2B, ISLR, JAK3, MGMT, MIF, MMP17, MMP19, POLR2E, PRKG2, MASP1, PTGIR, ROM1, MRPL12, TBX5, TCF21, TNXB, COL14A1, GDF5, WISP1, S1PR2, HAND2, PRDX4, KDELR1, EMILIN1, ABCA6, ASPN, C19orf24, ADI1, GPR137, KIAA1614, PODNL1, ADM2, SVEP1, ZNF358, SIX5
Fibroblasts_HPCA_1___211	Aran_et_al_2017	ADH1B, C7, CACNA1C, CAMLG, FTL, HEXA, IDUA, ISLR, JAK3, IPO5, MGST3, NFATC4, NFE2L2, PRKG2, MASP1, PTGIR, RNH1, TBX5, TCF21, TFDP1, HAND2, BAG2, PRDX4, MTHFD2, XPOT, CSTF2T, ZNF771, ADI1, FBXL8, PCDHGA11, GPR137, KIAA1614, PAPPA2, C7orf25
Fibroblasts_HPCA_2___212	Aran_et_al_2017	ADH1B, ANXA11, ARF4, C7, CACNA1C, CAMLG, FTL, GOLGA1, HEXA, IDUA, ISLR, JAK3, IPO5, LTBR, SMAD5, MGST3, NFATC4, NFE2L2, PFDN5, PRKG2, MASP1, PTGIR, RNH1, TADA2A, TBX5, TCF21, TFDP1, VIM, CGGBP1, HAND2, BAG2, HDAC5, PRDX4, MTHFD2, KDELR1, TMEM115, XPOT, CSTF2T, DNPEP, MKRN2, TOR1AIP1, ZCCHC4, RASL12, ZNF771, ADI1, FBXL8, PCDHGA11, GPR137, CASS4, ATP13A1, CYP20A1, ZNF471, KIAA1614, PAPPA2, MOSPD3, C7orf25, UBE3B, TEX261, ZNF358, EML3, C6orf120
Fibroblasts_HPCA_3___213	Aran_et_al_2017	ADD1, ADH1B, ALDH9A1, ANXA11, ARF4, ARHGAP6, C7, CACNA1C, CAMLG, CIRBP, CSF1, CYBA, DPT, DUT, FGF7, FMO2, FTL, GARS, GOLGA1, GSTM5, HEXA, HIC1, HPS1, HTR2B, IDUA, ISLR, JAK3, IPO5, LTBR, LTC4S, SMAD5, MGMT, MGST3, MMP17, MMP19, NFATC4, NFE2L2, P4HB, PDE4A, PFDN5, PGK1, PIK3R2, PRKG2, MASP1, PTGIR, RASA2, RNF4, RNH1, ROM1, RPL37A, SH3BP2, SNAPC2, TADA2A, TBX5, TCF21, TFDP1, CLEC3B, TNXB, VIM, ZNF32, DEK, NDST2, CGGBP1, WISP1,

		S1PR2, RPL23, HAND2, BAG2, LAPTM4A, HDAC5, MPHOSPH10, TFG, PRDX4, MTHFD2, SEC24A, KDELR1, KDELR2, EMILIN1, LSM6, XPOT, ZBTB1, FAIM2, GANAB, CSTF2T, ABCA6, SNED1, TOR1AIP1, CECR5, RASL12, ZNF771, AMOTL2, HDAC7, UBE2D4, ASPN, TXNL4B, SLC35A5, SHQ1, ADI1, DDX19A, ZNF444, FBXL8, ZNF446, EXOC1, SPATA7, TMEM165, PCDHGA11, GPR137, CASS4, ATP13A1, KIAA1614, EDA2R, PAPPA2, MOSPD3, C11orf95, C7orf25, ATG9A, TBC1D17, SLC35E1, SVEP1, SLC25A32, C6orf62, WDR73, MFSD5, ZNF358, EML3, DPY19L4
GMP_BLUEPRINT_1___214	Aran_et_al_2017	ARHGAP6, ATP5J, CD5L, MS4A3, CDH9, CPA3, CRHBP, CRYGD, CTSG, DNTT, FLT3, GABPA, LPAR4, H2AFZ, HDC, HMGB2, HNRNPA1, ITGA9, KCNJ14, LDHB, MPO, MTIF2, HNRNPM, NPM1, SERPINB10, PNN, PRG2, PRKG2, PSMA4, RAG2, RNASE2, RNASE3, RPL5, RYR3, SELP, STIL, SRP9, SSB, STXBP3, TEC, TOP2B, TRH, UBB, UMPS, VPREB1, XPO1, DEK, NCOA4, TTF2, UBA3, UBA2, SMNDC1, ARPP21, MRPL3, PARK7, CBX3, SMC5, GTPBP4, HPGDS, GNL2, CLEC1B, NOL7, UFC1, VPS54, GAR1, MRPL20, TTC27, WDR12, KIF17, MAP7D3, CXorf21
GMP_BLUEPRINT_2___215	Aran_et_al_2017	APLNR, ARHGAP6, ATP5J, CD5L, MS4A3, CDH9, CPA3, CRHBP, CRYGD, CTSG, DHX9, DNTT, EIF4E, ELANE, EWSR1, FOXI1, FLT3, GABPA, GLRA2, LPAR4, GUCY2D, H2AFZ, HDC, HMGB2, HNRNPA1, HNRNPD, ITGA9, KARS, KCNJ14, KPNA2, LDHB, MC4R, MPO, MTIF2, HNRNPM, NDUFC2, NPM1, SERPINB10, PNN, PRG2, PRKG2, PSMA4, RAG2, RNASE2, RNASE3, RPL5, RPL15, RYR3, SELP, STIL, SLN, SRP9, SSB, STXBP3, TEC, TOP2B, TRH, UBB, UMPS, VPREB1, XPO1, DEK, NCOA4, ANP32A, TAF15, TTF2, UBA3, COX7A2L, DDX21, UBA2, SMNDC1, ARPP21, MRPL3, PARK7, CBX3, SMC5, METAP1, GTPBP4, CLDN17, HPGDS, GNL2, CLEC1B, RWDD1, NOL7, NOP16, UFC1, VPS54, LUC7L3, GAR1, COMMD8, MRPL20, TTC27, WDR12, KIF17, MAP7D3, CXorf21
GMP_BLUEPRINT_3___216	Aran_et_al_2017	ATP5J, CD5L, MS4A3, CDH9, CPA3, CRHBP, CRYGD, CTSG, DNTT, FOXI1, FLT3, LPAR4, H2AFZ, HDC, HMGB2, HNRNPA1, LDHB, MPO, MTIF2, HNRNPM, NPM1, SERPINB10, PNN, PRG2, PSMA4, RAG2, RNASE2, RNASE3, RPL5, RPL15, RYR3, STIL, SRP9, SSB, TEC, TOP2B, TRH, UBB, UMPS, VPREB1, DEK, TTF2, UBA3, UBA2, ARPP21, MRPL3, PARK7, CBX3, SMC5, GTPBP4, GNL2, NOL7, UFC1, VPS54, GAR1, MRPL20, TTC27, WDR12, KIF17
GMP_HPCA_1___217	Aran_et_al_2017	ALOX12, CD33, CEACAM4, CTSG, DNTT, ERCC3, GPR3, GSTM5, H3F3B, HNRNPA2B1, INSL3, KARS, KCNJ14, LPO, MPO, NCL, OMD, PARK2, SERPINI2, POU2F1, PRTN3, PEX2, SLC5A5, SUPV3L1, SUV39H1, TRH, DNAJC7, VPREB1, ZNF35, ZNF207, LUZP1, SLC25A14, TRIP11, CNOT8, NCOR1, PUM1, BMS1, SMG7, ARPP21, CLP1, GLMN, LAMB4, GPATCH8, WDR43, RPRD2, GTPBP4, ZNF593, CDK5RAP1, SETD4, POLE3, WDR60, RNF220, ENOSF1, PRPF40A, DDX27, IQCC, NGLY1, ZC3H15, C21orf59, KIF17, RBM25, MRPL9, B3GNT4, MUL1, TCTN2, TTC26, CHD9, CEP63, LAS1L, RBM4B, MYOZ3, IQSEC3
GMP_HPCA_2___218	Aran_et_al_2017	CEACAM8, CLC, CSF1R, DEFA4, FEN1, LPAR4, MEFV, CXCL9, MPO, CFP, PRG2, RNASE2, CLEC1B, ZNF710
GMP_HPCA_3___219	Aran_et_al_2017	CCNT2, CD33, CHD4, AP2M1, DDOST, DHX9, DNMT1, ENO1, ERCC3, FLT3, GAS8, GGCX, GPR3, H3F3B, HNRNPA1, HNRNPA2B1, HNRNPH3, IK, IL3RA, IMPDH2, INSL3, KARS, KCNJ14, IPO5, LPO, MPO, MTIF2, NCL, OMD, PAFAH1B2, SERPINI2, POU2F1, PRTN3, RFC1, SLC5A5, SUPV3L1, SUV39H1, TCP1, NR2C1, TRH, DNAJC7, UBE2G2, ZNF35, ZNF207, LUZP1, ARID1A, TTF2, EIF3A, HDAC3, SLC24A1, CNOT8, RBM39, NCOR1, BMS1, ZBTB39, SMG7, RBM19, THRAP3, HMGXB4, HNRNPR, CALCOCO2, DNAJA2, ARPP21, ERP29, CLP1, SF3B2, DUSP12, GPN1, LAMB4, WDR43, RPRD2, PPIP5K2, GTPBP4, ATXN10, COG4, APPL1, DNAJC2, GNL2, GOLGA7, ANAPC5, LUC7L3, TXNL4B, MED9, WDR60, HEATR1, RNF220, SLC25A36, WDR41, ENOSF1, DDX27, NGLY1, ZC3H15, NIT2, KIF17, USP36, RBM25, MRPL9, B3GNT4, MUL1, MAP7D3, CSPP1, TCTN2, CPSF7, LAS1L, HPS4, G6PC3, IRGQ, PRRC1, HNRNPA3, IQSEC3
GMP_NOVERSHTERN_1___220	Aran_et_al_2017	CLC, CPA3, CRHBP, DNTT, FLT3, MPO, PRG2, RNASE2
GMP_NOVERSHTERN_2___221	Aran_et_al_2017	CLC, CPA3, CRHBP, DNTT, FLT3, MPO, PRG2, RNASE2
GMP_NOVERSHTERN_3___222	Aran_et_al_2017	CLC, CPA3, CRHBP, DNTT, FLT3, MPO, PRG2, RNASE2
Hepatocytes_FANTOM_1___223	Aran_et_al_2017	ABAT, ACADL, ADH1A, ADH6, AFM, AGT, AGXT, AHSG, ALB, ALDOB, AMBP, ANPEP, AOX1, APCS, APOA1, APOA2, APOC1, APOC3, APOE, APOH, ARSE, ASGR1, SERPINC1, BAAT, BHMT, SERPING1, C1R, C1S, C2, C4BPA, C4BPB, C5, C8A, C8B, C8G, C9, SERPINA6, CD14, CDO1, AKR1C4, ABCC2, CPB2, CPN2, CPS1, CYP2C19, CYP2C8, CYP2C9, CYP2E1, CYP3A4, CYP3A5, CYP7A1, DAO, DIO1, DPYS, EHHADH, ENPEP, EPHX2, F2, F5, F9, F13B, FABP1, FGA, FGB, FGG, FGL1, GAS2, GATM, GC,

		GHR, GLDC, GRB14, GYS2, HABP2, SERPIND1, HGD, HHEX, HLF, HPD, HPN, HPX, HRG, IGFBP1, ITIH2, ITIH3, KCNJ8, KNG1, LECT2, LGALS4, LIPC, MAN1A1, MAOB, MAT1A, MBL2, MT1G, MT1M, MTTP, MYLK, NNMT, ORM1, OTC, PAH, SERPINA5, PCK1, ABCB1, ABCB4, SERPINA1, SERPINA4, SERPINF2, FXYD1, PON1, PPP1R3C, PROX1, PXMP2, RARRES2, RBP4, SALL1, SDC2, SLC2A2, SLC6A1, SLC10A1, SLC22A1, SPINK1, SPP1, AKR1D1, SULT2A1, TAT, SERPINA7, TDO2, TFPI, TFR2, TM4SF4, TTR, UGT2B4, UGT2B15, VIL1, VTN, ACOX2, KMO, PLA2G4C, HSD17B6, SKAP1, VNN1, NAT8, TM4SF5, PAPSS2, RGN, NR1I3, NR1H4, SLC17A2, ABCA8, UBD, SLCO1B1, SLC17A3, IQGAP2, FERMT2, SLC7A9, ANXA10, ATF5, CUX2, QPRT, BHMT2, MYRIP, PCOLCE2, GNMT, ANGPTL3, SLCO1B3, TMEM176B, SNX10, A1CF, SERPINA10, HSD17B11, HAO2, PIPOX, HAO1, FAM134B, ACSM5, SLC38A4, SLC47A1, TMEM176A, SLC30A10, APOM, GBA3, PBLD, ABCG5, ALDH8A1, TRPM8, ECHDC3, UGT2A3, AGMAT, INHBE, RUNDC3B
Hepatocytes_FANTOM_2___224	Aran_et_al_2017	ABAT, ADH1A, ADH1C, ADH6, AFM, AGT, AGXT, AHSG, ALB, ALDH1A1, ALDOB, AMBP, ANPEP, AOX1, APCS, APOA1, APOA2, APOC1, APOC3, APOE, APOH, ARSE, ASGR1, SERPINC1, BAAT, BHMT, SERPING1, C1R, C1S, C4BPA, C4BPB, C5, C8A, C8B, C8G, C9, SERPINA6, CDO1, AKR1C4, CPB2, CPS1, CRYM, CYP2C8, CYP2C9, CYP2E1, CYP3A4, CYP3A5, CYP7A1, DIO1, DPYS, EHHADH, ENPEP, F2, F5, F9, F13B, FABP1, FGA, FGB, FGG, FGL1, GAS2, GATM, GC, GHR, GRB14, GSTA1, GYS2, HABP2, SERPIND1, HGD, HHEX, HPD, HPN, HPX, HRG, IGFBP1, ITIH2, ITIH3, KNG1, LECT2, LGALS4, LIPC, MAOB, MAT1A, MBL2, MT1G, MT1M, MTTP, MYLK, NNMT, ORM1, OTC, PAH, SERPINA5, PCK1, ABCB1, ABCB4, SERPINA1, SERPINA4, SERPINF2, FXYD1, PON1, RARRES2, RBP4, SALL1, SDC2, SEPP1, SLC2A2, SLC6A1, SLC10A1, SLC22A1, SPINK1, SPP1, AKR1D1, SULT2A1, TAT, SERPINA7, TDO2, TM4SF4, TTR, UGT2B4, UGT2B15, VIL1, VTN, ACOX2, PLA2G4C, HSD17B6, SKAP1, VNN1, NAT8, PAPSS2, NR1I3, NR1H4, ABCA8, UBD, SLCO1B1, SLC17A3, IQGAP2, ANXA10, QPRT, BHMT2, MYRIP, PCOLCE2, GNMT, ANGPTL3, SLCO1B3, TMEM176B, SNX10, A1CF, PIPOX, HAO1, ACSM5, SLC38A4, TMEM176A, SLC30A10, APOM, PLSCR4, GBA3, ABCG5, ALDH8A1, TRPM8, UGT2A3, AGMAT, EFHD1, INHBE, RUNDC3B
Hepatocytes_FANTOM_3___225	Aran_et_al_2017	ACADL, ADH1A, ADH6, AFM, AGT, AGXT, AHSG, ALB, ALDOB, AMBP, ANPEP, AOX1, APCS, APOA1, APOA2, APOC1, APOC3, APOE, APOH, ARSE, ASGR1, SERPINC1, BAAT, BHMT, SERPING1, C1R, C1S, C2, C4BPA, C4BPB, C5, C8A, C8G, C9, SERPINA6, CDO1, AKR1C4, ABCC2, CPB2, CPS1, CYP2C8, CYP2C9, CYP2E1, CYP3A4, CYP3A5, DIO1, DPYS, EHHADH, ENPEP, F2, F5, F9, F13B, FABP1, FGA, FGB, FGG, FGL1, GAS2, GATM, GC, GHR, GLDC, GRB14, GYS2, HABP2, SERPIND1, HGD, HHEX, HLF, HPD, HPN, HPX, HRG, IGFBP1, ITIH2, ITIH3, KNG1, LGALS4, LIPC, MAOB, MAT1A, MBL2, MT1G, MT1M, MTTP, MYLK, NNMT, ORM1, OTC, PAH, SERPINA5, PCK1, ABCB1, ABCB4, SERPINA1, SERPINA4, SERPINF2, FXYD1, PON1, PXMP2, RARRES2, RBP4, SALL1, SDC2, SLC2A2, SLC6A1, SLC10A1, SLC22A1, SPINK1, SPP1, AKR1D1, SULT2A1, TAT, SERPINA7, TDO2, TFPI, TM4SF4, TTR, UGT2B4, UGT2B15, VIL1, VTN, ACOX2, KMO, PLA2G4C, HSD17B6, SKAP1, VNN1, NAT8, PAPSS2, NR1I3, NR1H4, ABCA8, SLCO1B1, SLC17A3, IQGAP2, ANXA10, BHMT2, MYRIP, PCOLCE2, GNMT, ANGPTL3, TMEM176B, A1CF, SERPINA10, HAO2, PIPOX, HAO1, ACSM5, SLC38A4, TMEM176A, APOM, GBA3, ABCG5, ALDH8A1, TRPM8, UGT2A3, AGMAT, INHBE, RUNDC3B
Hepatocytes_HPCA_1___226	Aran_et_al_2017	AADAC, ACADS, ACAT1, ADH1A, ADH1B, ADH1C, AFM, AHSG, ALDH2, ALDH3A2, AMBP, APOF, APCS, APOA1, APOA2, APOC3, APOE, ARG1, SERPINC1, BCHE, CEACAM1, BHMT, SERPING1, C1R, C4BPA, C5, C6, C8A, C8B, C8G, SERPINA6, CDO1, CEBPA, AKR1C4, CPB2, CPN2, CRP, CYB5A, CYP2A6, CYP2A7, CYP2C8, CYP2C9, CYP2J2, CYP3A5, DDC, DEFB1, DIO1, DPYS, EHHADH, ELF3, EPHX1, EPHX2, F2, F5, F7, F9, F10, F11, F13B, FABP1, FCN2, FGA, FGB, FGG, FGL1, G6PC, GC, GCGR, GCH1, GCKR, CXCL2, GSTA1, HAL, SERPIND1, CFH, HGFAC, HMGCS2, HP, HPX, HRG, HYAL1, IGFALS, ITIH3, KHK, KLKB1, KNG1, LBP, LCAT, LGALS4, MAN1A1, ALDH6A1, MT1F, MT1G, MT1X, ORM1, PCSK6, PCCA, PCK1, SERPINF1, SERPINA1, PLA2G2A, SERPINF2, PON1, PZP, CCL16, SLC2A2, SLC6A1, SLC7A2, SLC22A1, SPINK1, TAT, SERPINA7, UGT2B4, APOL1, HSD17B6, ABCB11, PROZ, KYNU, SELENBP1, TM4SF5, NR1H4, SLC17A4, SLC17A2, SLCO1B1, SLC22A7, GADD45G, SLC38A3, SDS, SLC27A5, SLC27A2, QPRT, HAAO, SHC2, CCDC69, ANGPTL3, SLCO1B3, NPC1L1, OSGIN1, A1CF, MLXIPL, SERPINA10, UPB1, HAO1, ACSM5, PID1, SLC38A4, C14orf105, SLC47A1, C4orf19, SLC30A10, IL17RB, OGDHL, SEMA4G, CYP4F12, AGMAT, ATF7IP2, MIA2, SEC14L4
Hepatocytes_HPCA_2___227	Aran_et_al_2017	AADAC, ACAT1, ADH1A, ADH1C, AFM, AGT, AHSG, ALDH2, AMBP, APOF, APCS, APOA1, APOA2, APOB, APOC3, APOE, APOH, ASGR2, BAAT, BDH1, CFB, BHMT,

		SERPING1, C2, C4BPA, C4BPB, C5, C6, C8A, C8B, C8G, CAT, SERPINA6, ENTPD5, CEBPA, AKR1C4, CPB2, CPN2, CTSO, CYP2A6, CYP2B6, CYP2C8, CYP2C9, CYP2J2, CYP3A5, DAO, DDC, DIO1, DPYS, EHHADH, EPHX1, EPHX2, F2, F5, F7, F9, F10, F11, F13B, FABP1, FCN2, FGA, FGL1, NR5A2, G6PC, GC, GCGR, GCH1, GCKR, GHR, GYS2, HABP2, HAL, SERPIND1, CFH, HGFAC, HMGCS2, HRG, HSD11B1, HSD17B2, HYAL1, IGFALS, INHBC, INSR, ITIH2, ITIH3, KHK, KLKB1, KNG1, LBP, LCAT, LECT2, LGALS4, LIPC, MAN1A1, MAT1A, ALDH6A1, MT1F, MT1G, PC, PCCA, PCK1, ENPP1, SERPINA1, SERPINF2, PON1, MASP1, PZP, RET, RNASE4, CCL16, SEL1L, SLC1A1, SLC2A2, SLC10A1, SLC22A1, SPINK1, SPP2, SULT2A1, TAT, SERPINA7, TFR2, THPO, TMPRSS2, TTPA, UGT2B4, DDO, APOL1, FCN3, HSD17B6, KCNK5, ABCB11, CREG1, PROZ, TM4SF5, LPIN2, NR1I3, NR1H4, SLC17A4, SLC25A13, SEC23A, UBD, SLCO1B1, SLC26A1, SLC22A7, SLC27A5, SLC27A2, SLC7A9, SEPHS2, SLC35D1, HAAO, BHMT2, GNMT, ANGPTL3, SLCO1B3, NPC1L1, A1CF, MLXIPL, SERPINA10, DCXR, UPB1, HAO1, ACSM5, PID1, SLC38A4, SLC47A1, C4orf19, SLC35C1, SLC30A10, IL17RB, RAB20, OGDHL, CYP4F11, GREM2, ALDH8A1, CYP4F12, COLEC11, GRTP1, AGMAT, ATF7IP2, COL18A1, CFHR5, INHBE, MIA2, SEC14L4
Hepatocytes_HPCA_3___228	Aran_et_al_2017	AADAC, ACAT1, ADH1A, ADH1B, ADH1C, AFM, AHSG, ALDH2, ALDH3A2, AMBP, APOF, APCS, APOA1, APOA2, APOC3, APOE, ARG1, SERPINC1, CEACAM1, BHMT, SERPING1, C1R, C4BPA, C5, C6, C8A, C8B, C8G, SERPINA6, CDO1, CEBPA, AKR1C4, CPB2, CPN2, CRP, CYB5A, CYP2A6, CYP2A7, CYP2C8, CYP2C9, CYP2J2, CYP3A5, DDC, DEFB1, DIO1, DPYS, EHHADH, ELF3, EPHX1, EPHX2, F2, F5, F7, F9, F10, F11, F13B, FABP1, FCN2, FGA, FGB, FGG, FGL1, G6PC, GC, GCGR, GCH1, GCKR, CXCL2, GSTA1, HAL, SERPIND1, CFH, HGFAC, HMGCS2, HP, HPX, HRG, HYAL1, IGFALS, ITIH3, KHK, KLKB1, KNG1, LBP, LCAT, LGALS4, MAN1A1, MT1F, MT1G, MT1X, ORM1, PCSK6, PCK1, SERPINA1, PLA2G2A, SERPINF2, PON1, PZP, CCL16, SLC2A2, SLC6A1, SLC7A2, SLC22A1, SPINK1, TAT, SERPINA7, UGT2B4, APOL1, HSD17B6, ABCB11, PROZ, KYNU, TM4SF5, NR1H4, SLC17A4, SLC17A2, SLCO1B1, SLC22A7, GADD45G, SLC38A3, SLC27A5, SLC27A2, HAAO, SHC2, CCDC69, ANGPTL3, SLCO1B3, NPC1L1, OSGIN1, A1CF, MLXIPL, SERPINA10, UPB1, HAO1, ACSM5, SLC38A4, C14orf105, SLC47A1, C4orf19, SLC30A10, IL17RB, OGDHL, CYP4F12, AGMAT, MIA2, SEC14L4
HSC_BLUEPRINT_1___229	Aran_et_al_2017	CD34, CRHBP, ERG, GSTM5, PLS3, MMRN1, LAPTM4B, FAM124B
HSC_BLUEPRINT_2___230	Aran_et_al_2017	CD34, CRHBP, ERG, FLT3, GSTM5, PLS3, SLC4A1, SCARF1, ZMYM3, MMRN1, XPO7, EXD2, LAPTM4B, KLHL9, LSM2, CCDC121, FAM124B, ATP8B4, TCEAL4, MYCT1
HSC_BLUEPRINT_3___231	Aran_et_al_2017	ALAS2, CA1, CD34, CRHBP, ERG, FLT3, GSTM5, GYPA, GYPE, PLS3, SLC4A1, SCARF1, MMRN1, AHSP, EXD2, LAPTM4B, KLHL9, FAM124B, ATP8B4, TCEAL4
HSC_FANTOM_1___232	Aran_et_al_2017	CRHBP, CRYGD, ELN, ERG, KCNJ13, MPL, PDE6G, PLA2G1B, SYPL1, ZNF3, RNF7, LECT1, CTNNA3, PHF20, EMCN, MYCT1
HSC_FANTOM_2___233	Aran_et_al_2017	CRHBP, ELN, GSTM5, PLA2G1B, SYPL1, LECT1, CTNNA3, EMCN
HSC_FANTOM_3___234	Aran_et_al_2017	CRHBP, ELN, GSTM5, SYPL1, CLEC3B, LECT1, CTNNA3, EMCN
HSC_NOVERSHTERN_1___235	Aran_et_al_2017	CRHBP, ELN, GSTM5, PDE6G, SYPL1, LECT1, CTNNA3, EMCN, VN1R1
HSC_NOVERSHTERN_2___236	Aran_et_al_2017	CRHBP, ELN, GSTM5, SYPL1, CLEC3B, LECT1, CTNNA3, EMCN
HSC_NOVERSHTERN_3___237	Aran_et_al_2017	CRHBP, CRYGD, ELN, PDE6G, SYPL1, LECT1, CTNNA3, PHF20, EMCN
iDC_HPCA_1___238	Aran_et_al_2017	ALOX15, F13A1, FCER2, IL3RA, CCL13, CCL17, CCL18, CCL23, CCL24, ALDH1A2, CD209
iDC_HPCA_2___239	Aran_et_al_2017	ALOX15, CD86, FCER2, IL3RA, CCL13, CCL17, CCL24, ALDH1A2, CLEC10A, SPINT2
iDC_HPCA_3___240	Aran_et_al_2017	ALOX15, F13A1, CCL13, CCL17, CCL18, CCL23, CCL24, CD209
Keratinocytes_ENCODE_1___241	Aran_et_al_2017	CALML3, CSTA, DSG3, F3, GJB5, SFN, IL1A, JAG2, KRT5, KRT6B, LAD1, PI3, SERPINB5, SOX15, SULT2B1, LY6D, FGFBP1, AP1M2, FST, HES2, TMEM40, RAB25, S100A14, MMP28, ZBED2, KRT6C
Keratinocytes_ENCODE_2___242	Aran_et_al_2017	CALML3, CSTA, DSG3, GJB5, SFN, IL1A, KRT6B, PI3, SOX15, FGFBP1, AP1M2, FST, HES2, TMEM40, RAB25, S100A14, ZBED2, KRT6C
Keratinocytes_ENCODE_3___243	Aran_et_al_2017	DSG3, GJB5, SFN, IL1A, KRT6B, SOX15, FGFBP1, AP1M2, TMEM40, S100A14

Keratinocytes_FANTOM_1___244	Aran_et_al_2017	ADAM8, AIM1, ALDH1A3, CALML3, CDH3, COL17A1, CSTA, CYP27B1, DSC3, DSG3, EPHA1, EREG, F3, FAT2, FGFR3, GJB3, GJB5, GNA15, SFN, CXCL3, IL1A, IL1B, ITGA6, ITGB4, ITGB6, JAG2, KCNJ15, KRT5, KRT6A, KRT6B, KRT14, KRT16, KRT17, LAD1, LAMA3, LAMB3, LAMC2, MMP9, NGFR, SERPINB2, PGF, SERPINB5, PKP1, FXYD3, PPL, PRSS8, PTHLH, S100A2, S100A8, S100A9, SDC1, SORL1, SOX15, SPRR1B, ST14, TGFA, XDH, TP63, ARTN, CLCA2, PLCH2, KIAA0040, FGFBP1, SH2D3A, AP1M2, TSPAN1, MPZL2, NDRG1, FST, ST6GALNAC2, PPP1R13L, PKP3, KLK8, LPAR3, CBLC, TRIM29, KLK5, G0S2, DEF6, IRX4, ANGPTL4, LSR, GPR87, HES2, ESRP1, EPN3, TMEM40, FERMT1, SLC2A9, RAB25, S100A14, DLK2, C1orf116, MMP28, ZBED2, ELMO3, TNS4, ALS2CL
Keratinocytes_FANTOM_2___245	Aran_et_al_2017	ADAM8, COL17A1, CSTA, CYP27B1, DSC3, DSG3, EREG, FAT2, GJB3, GJB5, GNA15, SFN, IL1A, IL1B, ITGB4, KRT5, KRT6A, KRT6B, KRT14, KRT16, KRT17, LAD1, LAMA3, LAMB3, MMP9, SERPINB5, PKP1, FXYD3, PRSS8, S100A2, S100A8, S100A9, SOX15, ST14, TP63, ARTN, CLCA2, PLCH2, FGFBP1, SH2D3A, AP1M2, MPZL2, FST, PPP1R13L, PKP3, KLK8, CBLC, TRIM29, KLK5, G0S2, ANGPTL4, LSR, GPR87, ESRP1, EPN3, FERMT1, SLC2A9, RAB25, S100A14, C1orf116, ZBED2, ELMO3, TNS4
Keratinocytes_FANTOM_3___246	Aran_et_al_2017	ADAM8, CALML3, COL17A1, CSTA, CYP27B1, DSC3, DSG3, EREG, FAT2, GJB3, GJB5, GNA15, SFN, IL1A, IL1B, ITGB4, KRT5, KRT6A, KRT6B, KRT14, KRT16, KRT17, LAD1, LAMA3, LAMB3, LAMC2, MMP9, SERPINB5, PKP1, FXYD3, PRSS8, S100A2, S100A8, S100A9, SOX15, ST14, TP63, ARTN, CLCA2, PLCH2, FGFBP1, SH2D3A, AP1M2, MPZL2, FST, PPP1R13L, PKP3, KLK8, CBLC, TRIM29, KLK5, G0S2, ANGPTL4, LSR, GPR87, ESRP1, EPN3, FERMT1, SLC2A9, RAB25, S100A14, C1orf116, ZBED2, ELMO3, TNS4
Keratinocytes_HPCA_1___247	Aran_et_al_2017	DSG3, IL1A, KRT5, KRT14, SERPINB5, PTK6, SULT2B1, LY6D, CLCA2, FGFBP1, PRMT5, PKP3, FLRT3, TP53AIP1, DLK2, MMP28, GJB4, KRT6C
Keratinocytes_HPCA_2___248	Aran_et_al_2017	BDKRB2, CALML3, CSTA, DSC3, DSG3, F3, GJB3, GJB5, SFN, IL1A, IRF6, JAG2, KRT5, KRT6B, KRT14, LAD1, PGF, PI3, SERPINB5, PKP1, PLA2G4A, PRSS8, PTK6, SLC6A11, SLC12A4, SOX15, SULT2B1, CORO2A, LY6D, CLCA2, FGFBP1, AP1M2, CNKSR1, B3GNT3, PRMT5, FST, DUSP14, LPAR3, FLRT3, TFCP2L1, HES2, TMEM40, FGD6, LTB4R2, RAB25, S100A14, DLK2, C1orf116, MMP28, ZBED2, ZNF750, GJB4, KRT6C
Keratinocytes_HPCA_3___249	Aran_et_al_2017	BDKRB2, CALML3, CSTA, DSC3, DSG3, F3, GJB3, GJB5, SFN, IL1A, IRF6, JAG2, KRT5, KRT6B, KRT14, LAD1, MST1R, PI3, SERPINB5, PKP1, PTK6, SOX15, SULT2B1, CORO2A, LY6D, CLCA2, FGFBP1, AP1M2, CNKSR1, B3GNT3, PRMT5, FST, LPAR3, TFCP2L1, HES2, TMEM40, FGD6, LTB4R2, RAB25, S100A14, C1orf116, MMP28, ZBED2, ZNF750, GJB4, KRT6C
ly_Endothelial_cells_FANTOM_1___250	Aran_et_al_2017	FLT4, HYAL2, CLEC1A, SOX18, ROBO4, CXorf36, MYCT1, KANK3
ly_Endothelial_cells_FANTOM_2___251	Aran_et_al_2017	ACVRL1, ANGPT2, RHOC, BMX, CETP, ERG, FLT4, FUS, GPR4, KDR, MYL2, NOTCH4, RALA, MAPK12, TEK, TIE1, CLDN5, HYAL2, TNFSF18, KALRN, FEZ2, SEMA6B, LYVE1, ARHGEF15, MMRN1, CD93, N4BP3, CLEC1A, EMCN, SOX18, ROBO4, RASIP1, TMEM39B, POMGNT1, MRPL17, CXorf36, MMRN2, MYCT1, NETO2, PLVAP, KANK3
ly_Endothelial_cells_FANTOM_3___252	Aran_et_al_2017	ANGPT2, FLT4, MAPK12, TEK, HYAL2, KALRN, FEZ2, CLEC1A, SOX18, ROBO4, CXorf36, MYCT1, KANK3
ly_Endothelial_cells_HPCA_1___253	Aran_et_al_2017	GJA4, SELE, TIE1, CLDN5, VWF, HYAL2, TNFSF18, ARHGEF15, CLEC1A, ROBO4, MMRN2, KANK3
ly_Endothelial_cells_HPCA_2___254	Aran_et_al_2017	GJA4, SELE, TIE1, CLDN5, VWF, TNFSF18, ROBO4, MMRN2, KANK3
ly_Endothelial_cells_HPCA_3___255	Aran_et_al_2017	FLT4, GJA4, SELE, TIE1, CLDN5, TPM3, VWF, HYAL2, TNFSF18, ARHGEF15, CLEC1A, ROBO4, MMRN2, KANK3
Macrophages_M1_BLUEPRINT_1___256	Aran_et_al_2017	ACP2, ABCD1, C1QA, FDX1, CCL22, CD163, SCAMP2, ADAMDEC1, ARL8B, HAMP
Macrophages_M1_BLUEPRINT_2___257	Aran_et_al_2017	ACP2, ABCD1, FDX1, CCL8, CCL22, CD163, ADAMDEC1, TREM2, HAMP
Macrophages_M1_BLUEPRINT_3___258	Aran_et_al_2017	ACP2, ADRA2B, ALCAM, ABCD1, ATOX1, ATP6V0C, ATP6V1E1, BLVRA, C1QA, CD48, CD63, CLCN7, TPP1, CLTC, CCR1, CMKLR1, SLC31A1, COX5B, FCER1G, FDX1, FOLR2, FPR3, FTL, HEXB, HK3, IL10, IL12B, ITGAE, LAIR1, CXCL9, MMP19, NARS, NDUFS2, P2RX7, PDCL, MAPK13, PTGIR, PTPRA, RELA, CCL7, CCL8, CCL19, CCL22, SRC, STX4, TCEB1, TFRC, AGPS, MARCO, SNX3, CD84, USP14, ITGB1BP1, ATP6V1F, TRIP4, CD163, CIAO1, WTAP, ARHGEF11, ABI1, SCAMP2, ACTR2, BCAP31, ZMPSTE24, BCKDK, EXOC5, STIP1, UQCR11, SDS, LILRB4, OGFR, TFEC, FKBP15, DNAJC13, TDRD7, STX12, IL17RA, ABTB2, FAM32A, SIGLEC7, SIGLEC9, ADAMDEC1,

		CECR5, SLC25A24, NRBP1, MS4A4A, TREM2, OTUD4, PQLC2, HAUS2, ARL8B, NECAP2, WDR11, ZC3H15, CCDC47, UTP3, MRS2, HAMP, MRPL40, VPS33A, CORO7, LIMD2, TMX1, DOT1L, ADO, ADCK2
Macrophages_M1_FANTOM_1___259	Aran_et_al_2017	ACP2, ADRA2B, ALCAM, TSPO, C3AR1, DAGLA, CALR, CHIT1, CYBB, CYC1, CYP19A1, DLAT, FCER1G, GP1BA, GPD1, IFNAR1, IL10, KCNJ5, KIFC3, MT2A, MYBPH, MYH11, MYO7A, P2RX7, PRDX1, RAB3IL1, RNH1, MRPL12, CCL1, CCL7, CCL8, CCL24, SRC, VIM, RRP1, MARCO, S1PR2, AP1M2, ACTR3, LILRB1, AFG3L2, SDS, LILRB4, EMILIN1, VSIG4, HSPB7, COQ2, ADAMDEC1, CECR5, WSB2, SLAMF8, DNASE2B, CLPB, MFSD7, ADCK2
Macrophages_M1_FANTOM_2___260	Aran_et_al_2017	ACP2, ADCY3, ADRA2B, ALCAM, TSPO, C1QA, C1QB, C3AR1, DAGLA, CD63, CHIT1, CMKLR1, SLC31A1, CSF1, CSF1R, CYBB, CYC1, CYP19A1, FANCE, FCER1G, FDX1, FPR3, FTL, GP1BA, GPD1, HEXB, IL10, KCNJ1, KCNJ5, KIFC3, LAMP1, MMP19, MSR1, MT2A, MYBPH, MYO7A, P2RX7, PRDX1, RAB3IL1, MRPL12, CCL1, CCL7, CCL8, CCL18, CCL19, CCL24, SLC6A12, SPR, SRC, RRP1, MARCO, PKD2L1, S1PR2, CD163, LONP1, AP1M2, IGSF6, LILRB1, SDS, LILRB4, EMILIN1, VSIG4, TFEC, PHLDB1, CYFIP1, FKBP15, NCAPH, MYOF, HSPB7, ADAMDEC1, GLRX2, NDUFAF1, SPG21, MS4A4A, ATP6V1D, ATP6V1H, TREM2, PQLC2, TMEM70, PLEKHB2, TMEM33, SLAMF8, HAMP, DNASE2B, MYOZ1, LONRF3, CLPB, MFSD7, ADCK2
Macrophages_M1_FANTOM_3___261	Aran_et_al_2017	ACP2, ADCY3, ADRA2B, ALCAM, ABCD1, ANXA2, ATP6V1A, C1QA, C1QB, C3AR1, DAGLA, CD80, CD63, CHIT1, CMKLR1, SLC31A1, CSF1, CSF1R, CYBB, CYC1, CYP19A1, FANCE, FDX1, FPR2, FPR3, GPD1, HEXB, KCNJ1, KCNJ5, KIFC3, MMP19, MSR1, MT2A, MYBPH, P2RX7, MAPK13, S100A11, CCL1, CCL7, CCL8, CCL18, CCL19, CCL22, CCL24, SLC1A2, SLC6A12, SLC11A1, SIGLEC1, SRC, TIE1, MARCO, HYAL2, CD163, LONP1, IGSF6, LILRB1, CD300C, SDS, LILRB4, EMILIN1, VSIG4, PHLDB1, NCAPH, CLEC4E, MYOF, HSPB7, ADAMDEC1, GLRX2, MS4A4A, ATP6V1H, TREM2, TMEM70, TMEM33, KCNK13, SLAMF8, HAMP, DNASE2B, MYOZ1, MFSD7, ADO, ADCK2, TBC1D16
Macrophages_M2_BLUEPRINT_1___262	Aran_et_al_2017	ACP2, ADCY3, ABCD1, ALK, ARSB, ATP2A2, ATP6V1C1, ATP6V0A1, TSPO, CAMP, CANX, CD63, CD81, CLCN7, TPP1, SLC31A1, FDX1, FGR, FTL, GLB1, HADHB, NCKAP1L, HEXA, HEXB, HPS1, IFNAR1, ITGAX, KCNJ5, LAIR1, LAMP1, MSR1, MYO9B, P2RX7, SDCBP, SNX1, SNX2, STX4, MARCO, CDS2, PABPC4, ATP6V0D1, PICK1, ARHGEF11, HS3ST2, PDCD6IP, SCAMP2, COL4A3BP, HSPH1, OS9, SDS, LILRB4, VSIG4, GABARAP, TFEC, WDFY3, TBC1D9B, ZC3H3, CYFIP1, PLEKHM2, FKBP15, SMG5, UNC50, GGA1, SNX5, SLC39A1, ADAMDEC1, COMMD9, SLC25A24, SPG21, MS4A4A, ANKFY1, BTBD1, STX18, RIN2, PQLC2, TMEM70, ACSM5, AGGF1, SLC38A7, VPS53, NOP10, IARS2, CCDC88A, VPS35, TMEM184C, EXOC1, SLAMF8, C16orf62, POGK, HAMP, DNASE2B, MTMR14, GORASP1, C10orf76, LONRF3, UBXN6
Macrophages_M2_BLUEPRINT_2___263	Aran_et_al_2017	CLCN7, FGR, GLB1, HEXA, HEXB, HS3ST2, FKBP15, PQLC2, TMEM70, SLC38A7
Macrophages_M2_BLUEPRINT_3___264	Aran_et_al_2017	ACP2, ALK, ARSB, ATP6V0A1, CD63, CLCN7, TPP1, SLC31A1, FGR, GLB1, HADHB, NCKAP1L, HEXA, HEXB, IFNAR1, KCNJ5, MYO9B, P2RX7, SDCBP, MARCO, ATP6V0D1, HS3ST2, PDCD6IP, COL4A3BP, CYFIP1, PLEKHM2, FKBP15, SMG5, SLC39A1, COMMD9, MS4A4A, STX18, PQLC2, TMEM70, SLC38A7, VPS53, HAMP, LONRF3
Macrophages_M2_HPCA_1___265	Aran_et_al_2017	ADRA2B, AP1B1, ALDH9A1, ANXA11, AQP8, CD52, ELK1, FH, FLT1, GPD1, NCKAP1L, HEXB, KCNJ1, LAMP1, MMP19, MSR1, NDUFB1, NPR1, PDE1B, PEX19, S100A6, SLC6A7, SLC6A12, SNAPC2, SNX1, TAF10, UCP3, UGP2, USF2, XPNPEP2, AKR7A2, SNX3, TNFSF14, NFS1, GSTO1, KIAA0196, HS3ST2, BCAP31, CEPT1, BAIAP2, SLC9A6, VTI1B, ARFGEF2, HSPH1, LILRA2, EFR3A, FKBP15, NCAPH, ZCCHC4, TMED5, MYO15A, NAGPA, ZNF219, MS4A4A, ANGPT4, ATP6V1D, TREM2, KCTD5, PQLC2, AGGF1, MFN1, IARS2, CCDC88A, KCNK13, TMEM9B, POGK, MYOZ1, IPPK, CARD14, ALG9, MRM1, DHX57, SLC25A46, OSBPL11, CCDC85C
Macrophages_M2_HPCA_2___266	Aran_et_al_2017	ADRA2B, DNASE1L3, FDX1, GPD1, GUCA1A, KCNJ1, MSR1, PDE1B, UCP3, HS3ST2, SDS, MS4A4A, TREM2, DNASE2B, MYOZ1
Macrophages_M2_HPCA_3___267	Aran_et_al_2017	ADRA2B, CD52, GPD1, MSR1, NPR1, UCP3, HS3ST2, MYO15A
Macrophages_BLUEPRINT_1___268	Aran_et_al_2017	ACP2, ATOX1, ATP6V0C, ATP6V1E1, C1QA, CD9, CD48, CLCN7, CCR1, CRYBB1, CYBB, FCER1G, FDX1, FOLR2, FPR3, FTL, HEXA, HEXB, KCNJ5, LAIR1, M6PR, MDH1, NUBP1, P2RX7, RAC1, CCL8, CCL22, STX4, TCEB1, TYROBP, VAMP8, MARCO, CD84, ATP6V0E1, ATP6V1F, CD163, CIAO1, LY86, ARHGEF11, HS3ST2, ARPC4,

		ATP6AP2, BAIAP2, PRDX3, ERP29, LILRB5, SDS, LILRB4, VSIG4, FKBP15, ZZZ3, SIGLEC7, SIGLEC9, ADAMDEC1, ORMDL2, COMMD9, SPG21, MS4A4A, UBE2D4, TRAPPC2L, TREM2, TMEM70, ARL8B, TMEM126B, SLAMF8, C12orf4, HAMP, GUF1, YIF1B
Macrophages_BLUEPRINT_2___269	Aran_et_al_2017	ACP2, ATOX1, ATP6V0C, ATP6V1E1, C1QA, CD9, CD48, CD63, CLCN7, TPP1, CCR1, CMKLR1, SLC31A1, COX5B, COX7B, COX8A, CRYBB1, CYBA, CYBB, FCER1G, FDX1, FOLR2, FPR3, FTL, GLB1, HEXA, HEXB, KCNJ5, LAIR1, LAMP1, NUBP1, NDUFB3, NDUFS3, NDUFS6, P2RX7, PCMT1, MAPK13, PSME1, RB1, CCL8, CCL22, SDHD, SUMO3, SNX2, TCEB1, TYROBP, UQCRC2, VAMP8, MARCO, SNX3, CD84, ATP6V0E1, ITGB1BP1, S1PR2, ATP6V1F, CD163, COX5A, CIAO1, LY86, ARHGEF11, HS3ST2, SCAMP2, ARPC4, ATP6AP2, BAIAP2, PRDX3, ERP29, UQCR11, LILRB5, SDS, LILRB4, VSIG4, STAB1, FKBP15, DNAJC13, CLEC5A, ZZZ3, SIGLEC7, SIGLEC9, ADAMDEC1, ORMDL2, COMMD9, UQCR10, SPG21, MS4A4A, UBE2D4, TRAPPC2L, TREM2, KCTD5, PQLC2, COMMD8, TMEM70, ARL8B, SLC38A7, NOP10, WDR11, TMEM126B, TMEM9B, SLAMF8, C12orf4, MRS2, HAMP, DNASE2B, GUF1, MS4A6A, MRPL40, PPCS, PMFBP1, YIF1B, ADCK2, HIGD2A
Macrophages_BLUEPRINT_3___270	Aran_et_al_2017	ACP2, ATOX1, ATP6V0C, ATP6V1E1, C1QA, CD9, CD48, CD63, CLCN7, CCR1, COX8A, CRYBB1, CYBB, FCER1G, FDX1, FOLR2, FPR3, FTL, HEXA, HEXB, KCNJ5, LAIR1, LAMP1, NDUFB3, NDUFS6, P2RX7, MAPK13, CCL8, CCL22, SNX2, TCEB1, TYROBP, UQCRC2, VAMP8, MARCO, SNX3, CD84, ATP6V0E1, ITGB1BP1, ATP6V1F, CD163, COX5A, CIAO1, LY86, ARHGEF11, HS3ST2, SCAMP2, ARPC4, ATP6AP2, ERP29, UQCR11, LILRB5, SDS, LILRB4, VSIG4, STAB1, FKBP15, DNAJC13, CLEC5A, ZZZ3, SIGLEC7, SIGLEC9, ADAMDEC1, ORMDL2, COMMD9, SPG21, MS4A4A, UBE2D4, TREM2, KCTD5, TMEM70, ARL8B, NOP10, WDR11, TMEM126B, SLAMF8, C12orf4, HAMP, GUF1, MS4A6A, MRPL40, PPCS, PMFBP1, YIF1B, ADCK2
Macrophages_FANTOM_1___271	Aran_et_al_2017	ACP2, CHIT1, CSF1, CYP19A1, FDX1, HK3, MSR1, CCL22, SLC6A12, CD84, SDS, VSIG4, CLEC5A, ADAMDEC1, HAMP, DNASE2B, MYOZ1
Macrophages_FANTOM_2___272	Aran_et_al_2017	ACP2, ADCY3, ALCAM, ABCD1, BPI, CHIT1, CYP19A1, HK3, KCNJ1, KCNMB1, CXCL9, MMP8, MSR1, CCL1, CCL7, CCL22, SLC1A2, SLC6A12, CD84, CD163, SDS, LILRB4, VSIG4, FKBP15, NCAPH, CLEC5A, ADAMDEC1, ATP6V1H, PQLC2, SLAMF8, HAMP, DNASE2B, MYOZ1, LONRF3, MFSD7
Macrophages_FANTOM_3___273	Aran_et_al_2017	ACP2, ADCY3, ALCAM, ABCD1, ATP6V1A, ATP6V0A1, BPI, CHIT1, SLC31A1, CSF1, CYBB, CYP19A1, FDX1, FGR, HK3, ITGAX, KCNJ1, KCNMB1, CXCL9, MMP8, MMP19, MSR1, P2RX7, MAPK13, CCL1, CCL7, CCL22, SLC1A2, SLC6A12, SLC11A1, STX4, NUMB, MARCO, CD164, CD84, CD163, CIR1, ARHGEF11, BCAP31, ATP6AP2, SDS, LILRB4, VSIG4, FKBP15, NCAPH, CLEC5A, ADAMDEC1, ATP6V1H, PQLC2, CCDC88A, PCDHB11, SLAMF8, HAMP, DNASE2B, MYOZ1, LONRF3, MFSD7
Macrophages_HPCA_1___274	Aran_et_al_2017	ACADVL, ACP2, ARSB, ATP6V1A, ATP6V0C, ATP6V1C1, CD63, CETN2, CCR1, SLC31A1, COX5B, COX15, CYBB, DBI, ECHS1, FDX1, GRB2, HADHB, HEXA, HEXB, HK3, HMGCL, ITGAX, KIFC3, TNPO1, LAIR1, LAMP1, MGST3, MSR1, NARS, NDUFA8, NDUFB6, NDUFS8, PRDX1, PEX14, MAPK13, PSMD10, PTPN12, PEX19, QDPR, RAB1A, RAB5C, RALA, RENBP, CLIP1, CCL7, CCL18, SDHB, SRC, STX4, TCEB1, MLX, TRAF3, NSMAF, AGPS, MARCO, SNX4, ATP6V1F, LONP1, GSTO1, BAG3, CIR1, FEZ2, PDCD6IP, SNUPN, BCAP31, STAM2, IGSF6, ZMPSTE24, TMEM147, VTI1B, TGOLN2, SPIN1, LILRB4, EMILIN1, VSIG4, EFR3A, FKBP15, CLEC5A, IBTK, NPTN, ATP2C1, SIGLEC9, ADAMDEC1, CNIH4, GLRX2, DERA, NDUFAF1, HSD17B12, ZDHHC3, TNFRSF12A, MS4A4A, ATP6V1D, ATP6V1H, TMBIM4, STYXL1, BTBD1, NUDT9, TMEM33, NOP10, TMEM127, ACTR10, KCMF1, TULP4, C12orf4, RTN4, MKL2, HAMP, DNASE2B, KLHL12, NSUN3, ELOVL1, SLC30A5, MAPKAP1, LONRF3, TCEAL4, CHD9, TM2D1, MFSD7, G6PC3, TBC1D16, 44084, ZDHHC24
Macrophages_HPCA_2___275	Aran_et_al_2017	ACP2, ARSB, ATP6V1A, ATP6V0C, ATP6V1C1, CD63, CETN2, CCR1, SLC31A1, COX5B, COX15, CYBB, DBI, ECHS1, FDX1, HEXB, LAIR1, LAMP1, MGST3, MSR1, NDUFA8, NDUFS8, PRDX1, MAPK13, PSMD10, PTPN12, PEX19, RAB1A, RALA, RENBP, CCL7, SDHB, STX4, TCEB1, MLX, TRAF3, NSMAF, AGPS, MARCO, ATP6V1F, GSTO1, BCAP31, IGSF6, TMEM147, VTI1B, LILRB4, EMILIN1, VSIG4, EFR3A, CLEC5A, NPTN, ATP2C1, SIGLEC9, ADAMDEC1, CNIH4, GLRX2, DERA, NDUFAF1, ZDHHC3, TNFRSF12A, MS4A4A, ATP6V1D, ATP6V1H, NUDT9, TMEM33, C12orf4, RTN4, HAMP, DNASE2B, ELOVL1, SLC30A5, MAPKAP1, LONRF3, MFSD7, G6PC3, TBC1D16, 44084, ZDHHC24
Macrophages_HPCA_3___276	Aran_et_al_2017	ACADVL, ACP2, ARSB, ATP6V1A, ATP6V0C, ATP6V1C1, CD63, CETN2, CCR1, SLC31A1, COX5B, COX15, CYBB, DBI, ECHS1, FDX1, GRB2, HADHB, HCCS, HEXA, HEXB, HK3, HMGCL, ITGAX, TNPO1, LAIR1, LAMP1, MGST3, MSR1, NDUFA8, NDUFS8, PRDX1, MAPK13, PSMD10, PTPN12, PEX19, QDPR, RAB1A, RALA, RENBP, CLIP1,

		CCL7, SDHB, SRC, STX4, TCEB1, MLX, TRAF3, UQCRC2, USF2, NSMAF, AGPS, MARCO, SNX4, ATP6V1F, GSTO1, PDCD6IP, BCAP31, STAM2, IGSF6, ZMPSTE24, CEPT1, TMEM147, VTI1B, TGOLN2, SPIN1, LILRB4, TMEM115, EMILIN1, VSIG4, EFR3A, FKBP15, CLEC5A, NPTN, ATP2C1, TRAPPC3, SIGLEC9, ADAMDEC1, CNIH4, SLC25A24, GLRX2, DERA, NDUFAF1, ZDHHC3, TNFRSF12A, MS4A4A, ATP6V1D, ATP6V1H, BTBD1, NUDT9, TMEM33, TMEM127, ACTR10, KCMF1, TULP4, C12orf4, RTN4, MKL2, HAMP, DNASE2B, NSUN3, ELOVL1, SLC30A5, C7orf25, MAPKAP1, PANK3, LONRF3, TM2D1, MFSD7, SETD3, G6PC3, TBC1D16, 44084, ZDHHC24
Macrophages_IRIS_1___277	Aran_et_al_2017	ACP2, ADCY3, ABCD1, ATP6V1A, CHIT1, CLCN7, TPP1, SLC31A1, COX5B, CYP19A1, FDX1, HEXA, HK3, KCNJ1, M6PR, MSR1, SLC6A12, CD164, CD84, LONP1, ATP6AP2, TGOLN2, SDS, LILRB4, VSIG4, FKBP15, CLEC5A, ADAMDEC1, ATP6V1H, STX18, PQLC2, DNASE2B, MYOZ1, SLC30A5, LONRF3, MFSD7, LDHAL6B
Macrophages_IRIS_2___278	Aran_et_al_2017	ACP2, ADCY3, ALCAM, ABCD1, ARSB, ATOX1, ATP6V1A, ATP6V0C, ATP6V1E1, ATP6V0A1, BPI, TSPO, CD63, CHIT1, CLCN7, TPP1, SLC31A1, COX5B, CSF1, CYBB, CYP19A1, FDX1, HTT, HEXA, HK3, ITGAX, KCNJ1, LAMP1, M6PR, MMP19, MSR1, MTHFR, P2RX7, MAPK13, PTPRA, RABGGTA, SLC1A2, SLC6A12, STX4, TCEB1, NUMB, CD164, CD84, LONP1, CPNE6, CIR1, TTLL4, ARHGEF11, BCAP31, ATP6AP2, TGOLN2, SDS, LILRB4, VSIG4, FKBP15, CLEC5A, IL17RA, ADAMDEC1, NRBP1, SH3GLB1, ATP6V1H, STX18, PQLC2, TMEM33, SLC38A7, VPS53, CCDC88A, SLAMF8, HAMP, DNASE2B, MYOZ1, MTMR14, SLC30A5, MUL1, C12orf49, LONRF3, MFSD7, LDHAL6B, ZDHHC24
Macrophages_IRIS_3___279	Aran_et_al_2017	ACP2, CHIT1, CSF1, CYP19A1, FDX1, HK3, MSR1, CCL22, SLC6A12, CD84, SDS, VSIG4, CLEC5A, ADAMDEC1, HAMP, DNASE2B, MYOZ1
Mast_cells_FANTOM_1___280	Aran_et_al_2017	AMHR2, ANXA1, ANXA11, ATP6V1C1, BMPR1A, BTK, C3AR1, C8G, CASP10, CD22, CD33, SIGLEC6, CMA1, CPA3, CTSG, DIAPH1, DR1, MS4A2, GATA1, HDC, IL5, IL5RA, ITGA2B, KCNJ5, KRT1, LCP2, LTC4S, LYL1, MTR, NTRK1, OSBP, P2RX1, PAK2, PDE4A, PIK3R2, POLR2A, PPP3R1, PRG2, PRKAR1A, PTGDR, PTGER3, RAD23B, RENBP, RGS13, RXRB, SLC18A2, SNRNP70, SOS2, SYPL1, TADA2A, MAP3K7, TAL1, TEC, TPSAB1, RNF103, ZNF212, STAM, NDST2, AGPS, PABPC4, RGS11, CD84, CDC16, USP10, ZMYM4, WDR46, ZNF264, CTR9, DEPDC5, LRIG2, THRAP3, SNUPN, KLRG1, BAIAP2, HSPH1, SERINC3, IFT27, ATXN2L, U2AF2, ZBTB1, BAHD1, TRIM32, FAM120A, DNAJC13, ESYT1, ARHGEF12, ATXN10, UPF2, SPAG8, STAP1, HIBCH, SIGLEC7, SNX5, SIGLEC8, HPGDS, REM1, MRPS28, CPSF1, BET1L, ZBTB7A, NBAS, LAX1, DNAJC28, AGGF1, BTBD7, TTC17, CENPJ, ZNF471, CTDSP1, HRH4, ACBD3, DCLRE1B, ZNF426, MBOAT7, ZMYM1, FBXO11, CHD9, ZKSCAN3, C6orf25, FBXO38, MAGT1, USP48, OSBPL9, ZNF549, TIPRL
Mast_cells_FANTOM_2___281	Aran_et_al_2017	AMHR2, ANXA1, ANXA11, ATP6V1C1, BMPR1A, BTK, C3AR1, C8G, CASP10, CD22, SIGLEC6, CPA3, CTSG, DIAPH1, DR1, MS4A2, GATA1, HDC, IL5, ITGA2B, KCNJ5, KRT1, LCP2, LTC4S, LYL1, MTR, NTRK1, OSBP, P2RX1, PAK2, PIK3R2, POLR2A, PPP3R1, PRG2, PRKAR1A, PTGER3, RENBP, RGS13, RXRB, SLC18A2, SNRNP70, SYPL1, TADA2A, MAP3K7, TAL1, TEC, TPSAB1, RNF103, STAM, NDST2, AGPS, PABPC4, RGS11, CD84, CDC16, USP10, WDR46, ZNF264, CTR9, DEPDC5, SNUPN, KLRG1, BAIAP2, HSPH1, SERINC3, IFT27, ATXN2L, U2AF2, ZBTB1, FAM120A, DNAJC13, ESYT1, ARHGEF12, ATXN10, UPF2, STAP1, SIGLEC7, SNX5, SIGLEC8, HPGDS, REM1, CPSF1, BET1L, NBAS, LAX1, BTBD7, CENPJ, CTDSP1, HRH4, ACBD3, DCLRE1B, MBOAT7, CHD9, ZKSCAN3, C6orf25, USP48, OSBPL9, ZNF549, TIPRL
Mast_cells_FANTOM_3___282	Aran_et_al_2017	AMHR2, ANXA1, ANXA11, BMPR1A, BTK, C3AR1, C8G, CASP10, CD22, SIGLEC6, CPA3, CTSG, DIAPH1, MS4A2, GATA1, HDC, IL5, ITGA2B, KRT1, LTC4S, LYL1, NTRK1, OSBP, P2RX1, PAK2, PIK3R2, PRG2, PTGER3, RENBP, RGS13, RXRB, SLC18A2, SNRNP70, TADA2A, TAL1, TPSAB1, NDST2, AGPS, PABPC4, RGS11, USP10, WDR46, CTR9, DEPDC5, KLRG1, HSPH1, IFT27, ATXN2L, U2AF2, FAM120A, UPF2, STAP1, SNX5, SIGLEC8, HPGDS, REM1, CPSF1, BET1L, NBAS, LAX1, HRH4, ACBD3, DCLRE1B, MBOAT7, ZKSCAN3, C6orf25, OSBPL9, TIPRL
Megakaryocytes_BLUEPRINT_1___283	Aran_et_al_2017	ARHGAP6, GP1BA, HTR2A, MPL, PF4V1, SELP, GP6, CLEC1B, RUFY1
Megakaryocytes_BLUEPRINT_2___284	Aran_et_al_2017	ANXA3, ARHGAP6, GP1BA, MPL, PF4V1, SELP, NCKAP1, CLEC1B, TUBB1
Megakaryocytes_BLUEPRINT_3___285	Aran_et_al_2017	ANXA3, ARHGAP6, GP1BA, MPL, SELP, CLEC1B, LRP2BP, TUBB1
Megakaryocytes_NOVERSHTERN_1___286	Aran_et_al_2017	GP1BA, HBD, PF4V1, SELP, VWF, KALRN, RGS6, GP6, CLEC1B
Megakaryocytes_NOVERSHTERN_2___287	Aran_et_al_2017	GP1BA, HBD, PF4V1, SELP, VWF, KALRN, RGS6, GP6, CLEC1B, TUBB1

Megakaryocytes_NOVERSHTERN_3___288	Aran_et_al_2017	GP1BA, PF4V1, SELP, VWF, KALRN, RGS6, GP6, CLEC1B
Melanocytes_ENCODE_1___289	Aran_et_al_2017	CA8, MLANA, GJA3, OCA2, S100B, SOX10, GPR137B, TYR, KCNAB2, PIR, FARP2, KCNE4, QPCT, SLC45A2, TBC1D16
Melanocytes_ENCODE_2___290	Aran_et_al_2017	CA8, CDH3, MLANA, GJA3, MMP17, OCA2, CLEC11A, SOX10, GPR137B, TYR, TYRP1, KCNAB2, PIR, TNFRSF14, FARP2, QPCT, SLC45A2, WIPI1, MCOLN1, WFDC1, APH1B, UAP1L1, TBC1D16
Melanocytes_ENCODE_3___291	Aran_et_al_2017	CA8, CBR3, NOV, OCA2, SOX10, TYR, PIR, FARP2, QPCT, SLC45A2, UAP1L1, TBC1D16
Melanocytes_FANTOM_1___292	Aran_et_al_2017	DCT, MLANA, TRPM1, OCA2, SOX10, TYR, TYRP1, SLC16A6, PLXNC1, SLC45A2
Melanocytes_FANTOM_2___293	Aran_et_al_2017	ACACB, ACP5, ASPA, BCL2A1, CEACAM1, CAPN3, RUNX3, CDK2, LYST, CLCN5, ABCC2, CYP27A1, DAB2, DCT, EDNRB, ERBB3, ESR2, MLANA, IFI6, GCNT2, GJB1, GK, GMPR, GNAL, HLA-DRA, HLA-DRB1, HLA-F, IFI16, IFI27, IFI35, SP110, IFIT2, IFIT1, IFIT3, IRF4, ISG20, ITGB8, ITPKB, KCNJ13, KIT, L1CAM, MBP, MITF, TRPM1, MMP8, MX1, MX2, MYO5A, NOV, GPR143, OAS1, OAS2, OCA2, PAEP, PAX3, PDE3A, PDE3B, PDE4B, PLP1, PLSCR1, PROS1, RAB27A, SGK1, SLC1A4, SOX10, STAT1, TAP1, TCN1, TLR1, TYR, TYRP1, UBA7, WARS, ALX1, KCNAB2, GAS7, PIR, OASL, TNFRSF14, INPP4B, GYG2, KYNU, SLC16A6, ITM2A, SOX13, AATK, ISG15, GREB1, ZFYVE16, RNF144A, ZEB2, FARP2, XYLB, PLXNC1, HMG20B, TESK2, GPNMB, IFI44, IQGAP2, IFI44L, LZTS1, NLGN1, ATP10B, ANKRD28, MCF2L, NEDD4L, TDRD7, DAAM2, DDX58, CA14, SLC7A11, PRKD3, IFIT5, QPCT, SLC39A6, GAPDHS, FOXD3, CDH19, OSTM1, SLC43A3, ATP6V0A4, CDON, PI15, LAP3, SLC45A2, LEF1, HERC5, PLA1A, TRPV2, C21orf91, PLEKHA5, XAF1, BNC2, SAMD9, HERC6, MOCOS, WIPI1, TMEM140, MCOLN3, DDX60, DOCK10, BIN3, CYSLTR2, SEMA6A, AVPI1, RTP4, IFIH1, RAB17, GNPTAB, TMPRSS5, ARHGAP24, RSAD2, TBC1D16, ASB9, PRUNE2, PHACTR1, ZNF749
Melanocytes_FANTOM_3___294	Aran_et_al_2017	ABL2, ACACB, ACP5, APOD, ASPA, BCL2A1, CEACAM1, CAPN3, RUNX3, CBR3, CD36, CDK2, LYST, CLCN5, CLCN7, ABCC2, CTSK, CYP27A1, DAB2, DCT, NQO1, EDNRB, ERBB3, ESR2, ETV5, ACSL3, MLANA, IFI6, GCNT2, GJB1, GK, GMPR, GNAL, HLA-DPA1, HLA-DRA, HLA-DRB1, HLA-F, IFI16, IFI27, IFI35, SP110, IFIT2, IFIT1, IFIT3, IRF4, IRF7, ISG20, ITGB8, ITPKB, KCNJ13, KIT, L1CAM, LGALS3, MBP, MITF, TRPM1, MME, MMP8, MX1, MX2, MYO5A, NOV, GPR143, OAS1, OAS2, OCA2, PAEP, PAX3, PDE3A, PDE3B, PDE4B, PDE4D, PDK4, PLP1, PLSCR1, PRKCE, PROS1, RAB27A, SGK1, SLC1A4, SLC12A2, SOX10, SP100, STAT1, STAT5A, STX3, TAP1, TBX2, TCN1, TLR1, TYR, TYRP1, UBA7, VGF, WARS, ALX1, SORBS2, PPFIBP2, KCNAB2, IFITM1, GAS7, PIR, OASL, TNFRSF14, INPP4B, GYG2, KYNU, SLC16A6, UBE2L6, ITM2A, SOX13, AATK, ISG15, GREB1, ZFYVE16, RNF144A, TRIM14, ZEB2, FARP2, XYLB, NAMPT, PLXNC1, HMG20B, TESK2, GPNMB, IFI44, IQGAP2, IFI44L, SLC27A3, CIT, LZTS1, IRAK3, NLGN1, PDZRN3, ATP10B, ANKRD28, MCF2L, NEDD4L, TDRD7, DAAM2, ZFYVE26, DDX58, CA14, SLC7A11, PRKD3, IFIT5, QPCT, SLC39A6, SAMHD1, GAPDHS, FOXD3, CDH19, TRIB2, OSTM1, SLC43A3, ATP6V0A4, CDON, PI15, LAP3, SLC45A2, LEF1, HERC5, PLA1A, TRPV2, C21orf91, PLEKHA5, SPATA6, EGLN1, XAF1, BNC2, SAMD9, RPP25, HERC6, MOCOS, WIPI1, TMEM140, MCOLN3, DDX60, DOCK10, BIN3, CYSLTR2, SEMA6A, AVPI1, BCAN, RTP4, IFIH1, POPDC3, RAB17, GNPTAB, APOL6, TMPRSS5, APOLD1, ARHGAP24, RSAD2, TBC1D16, ASB9, PYHIN1, PRUNE2, PHACTR1, ZNF749
Memory_Bcells_BLUEPRINT_1___295	Aran_et_al_2017	ACRV1, ADCY2, AQP8, ART1, TNFRSF17, BLK, C4BPA, S100G, CASQ2, CD1C, CD19, MS4A1, CD22, SIGLEC6, CD37, CD72, CD79A, CD79B, CETP, CHRM2, CHRNA2, CCR6, CNR2, COX6A2, CPA2, CPB1, CSN1S1, NCAN, CYLC2, CYP2A7, CYP2C19, DCC, DPP6, DSP, FMO1, FSHR, GABRA4, GAD2, GK2, GNAT2, GNRHR, GPX5, GRIN2B, GRM6, HCRTR2, HLA-DPB1, HSD3B2, HTN3, INHBC, KCNA5, KCNJ10, KIF5A, KRT2, LECT2, CD180, MBL2, MC4R, MEFV, MEP1B, MAP3K9, NPY5R, NTRK3, OTC, PAX5, SERPINA4, PLIN1, PNOC, PNLIPRP1, POU4F2, PRKCB, PTH1R, PRPH2, SELP, SLC12A3, SLC17A1, SLN, SPIB, SYN2, SYPL1, TSHB, WNT2, ZIC3, NPHS2, UNC5C, ADAM20, PROZ, BAIAP3, KALRN, KRT75, CER1, TMPRSS11D, LY86, KIAA0125, AP1M2, SSX3, GLYAT, DSCR4, COLEC10, RRH, CCR9, CD3EAP, ADAM30, SP140, GGA2, HECW1, CRB1, TNFRSF13B, CHST5, AIPL1, SLC24A2, STAP1, CLDN17, TSPAN13, VPREB3, FSCN3, TAS2R14, WNT16, SLCO1C1, DDX4, ULK4, QRSL1, GPRC5D, SLC30A10, SLC17A7, PGLYRP4, VN1R1, HRH4, SLC5A7, CHP2, MS4A5, ZNF747, FCRL2, RIC3, TCTN2, TRPM3, MOGAT2, ADAMTS12, OBSCN, MYOZ3, ZNF548, KHDRBS2, SHISA6, FMO6P, ANKRD34C
Memory_Bcells_BLUEPRINT_2___296	Aran_et_al_2017	TNFRSF17, BLK, CAPN3, CD19, MS4A1, CD22, CD37, CD72, CD79A, CD79B, CCR6, CNR2, CPA2, CPB1, CSN1S1, DPP6, DSP, FMO1, GK2, GRM6, HNRNPL, INHBC,

		KCNA5, CD180, MC4R, PAX5, PNOC, POU4F2, PRKCB, SLC17A1, SPIB, SYPL1, TSHB, ZIC3, ADAM21, LY86, SCRN1, KIAA0125, RRH, CCR9, CD3EAP, SP140, GGA2, TNFRSF13B, AIPL1, STAP1, VPREB3, WNT16, SLCO1C1, DDX4, ULK4, QRSL1, SLC30A10, PGLYRP4, HRH4, SLC5A7, MS4A5, FCRL2, TCTN2, TRPM3, MOGAT2, OBSCN, ZNF548, PIKFYVE, SHISA6, FMO6P, ANKRD34C
Memory_Bcells_BLUEPRINT_3___297	Aran_et_al_2017	ART1, BLK, S100G, CASQ2, CD1C, CD19, MS4A1, CD22, SIGLEC6, CD37, CD72, CD79A, CD79B, CHRM2, CCR6, CNR2, CPB1, CSN1S1, CYP2A7, FMO1, GABRA4, GAD2, GK2, GNAT2, GRM6, INHBC, KCNJ10, KRT2, CD180, MEFV, MAP3K9, NPY5R, PAX5, PNOC, PNLIPRP1, POU4F2, PRKCB, SELP, SPIB, TSHB, KALRN, CER1, LY86, KIAA0125, SSX3, RRH, CD3EAP, SP140, GGA2, HECW1, TNFRSF13B, CHST5, AIPL1, SLC24A2, STAP1, TSPAN13, VPREB3, TAS2R14, SLCO1C1, DDX4, QRSL1, PGLYRP4, VN1R1, SLC5A7, ZNF747, FCRL2, TRPM3, MOGAT2, ZNF548, SHISA6
Memory_Bcells_HPCA_1___298	Aran_et_al_2017	BLK, CD19, MS4A1, CD22, CD79A, CD79B, GRM6, INHBC, PNOC, SPIB, TSHB, KIAA0125, CD3EAP, TNFRSF13B, TSPAN13, VPREB3, QRSL1, FCRL2, MOGAT2
Memory_Bcells_HPCA_2___299	Aran_et_al_2017	TNFRSF17, BLK, CD19, MS4A1, CD22, CD37, CD72, CD79A, CD79B, CSN1S1, DSP, FMO1, GK2, GRM6, INHBC, CD180, PNOC, POU4F2, PRKCB, SPIB, TSHB, LY86, KIAA0125, RRH, CCR9, CD3EAP, SP140, GGA2, TNFRSF13B, STAP1, VPREB3, QRSL1, HRH4, SLC5A7, FCRL2, ZNF548, SHISA6, FMO6P, ANKRD34C
Memory_Bcells_HPCA_3___300	Aran_et_al_2017	BLK, CD19, MS4A1, CD22, CD79A, CD79B, INHBC, PNOC, SPIB, KIAA0125, TSPAN13, VPREB3, QRSL1, FCRL2
Memory_Bcells_IRIS_1___301	Aran_et_al_2017	BLK, CD1C, CD79B, ODC1, SPIB, MBD4, LY86, SP140, TNFRSF13B, FSCN2, ZBTB32, NT5C, WNT16, TRMT61A
Memory_Bcells_IRIS_2___302	Aran_et_al_2017	BLK, CXCR5, CD19, MS4A1, CD22, CCR6, HTR3A, MGAT5, SPIB, RNGTT, MBD4, LY86, CXCL13, SP140, TNFRSF13B, ZBTB32, MIOS, AICDA, FCRL2
Memory_Bcells_IRIS_3___303	Aran_et_al_2017	BLK, CD1C, CD79B, ODC1, SPIB, MBD4, SP140, TNFRSF13B, FSCN2, ZBTB32, NT5C, WNT16
MEP_BLUEPRINT_1___304	Aran_et_al_2017	AHCY, CDK4, CPA3, CRHBP, ERG, GSTM5, HDC, EPCAM, PRG2, RYR3, MINPP1, PAICS, HPGDS, LAPTM4B
MEP_BLUEPRINT_2___305	Aran_et_al_2017	AHCY, CDK4, CPA3, CRHBP, GSTM5, HDC, EPCAM, POLE2, HPGDS, LAPTM4B, TCEAL4
MEP_BLUEPRINT_3___306	Aran_et_al_2017	BUB1B, CPA3, CRHBP, ERG, HDC, POLE2, RYR3, STIL, KIAA0101, SNX5, HPGDS, MRPL15, C11orf95, FAM124B
MEP_HPCA_1___307	Aran_et_al_2017	FXN, GATA1, HBD, KEL, MYL4, PCCB, PNMT, PRG2, RHAG, SURF2, TPSAB1, UNG, AIMP2, CHAF1B, RUVBL1, NAT6, ERAL1, HPGDS, MRPS2, CTNNBL1, EXOSC5, ACD, DCTPP1, MTG1
MEP_HPCA_2___308	Aran_et_al_2017	FCER1A, FMO1, FXN, KRT1, NTRK1, PCCB, PNMT, UNG, IFRD2, AIMP2, CHAF1B, RUVBL1, MRPL28, TMED1, FBXO7, ERAL1, HPGDS, MECR, MRPS2, DDX41, CTNNBL1, C21orf59, EXOSC5, ACD, DCTPP1, NOL12, MTG1
MEP_HPCA_3___309	Aran_et_al_2017	FXN, PCCB, PNMT, TMED1, HPGDS, MRPS2, CTNNBL1, ACD, NOL12
MEP_NOVERSHTERN_1___310	Aran_et_al_2017	ATIC, BCS1L, CCT6A, DDX1, DDX10, FXN, HNRNPAB, ITGA2B, PCCB, POLE2, RYR3, IFRD2, RUVBL1, PSMG1, LDB1, VAPA, TXNL1, KEAP1, URB1, LRPPRC, TRAP1, PRMT3, NUDC, FASTKD2, PDCD11, MYO16, PPRC1, WDR43, MLC1, SERBP1, ERAL1, GNL3, NUFIP1, RPUSD2, RRP15, MRPS2, MRTO4, NOP16, MKS1, PAK1IP1, CDKN2AIP, TSR1, WDR12, CTNNBL1, BCCIP, GUF1, CARS2, FN3KRP, OGFOD2, MRM1, NOL10, NAA15, ZKSCAN3, MED25, TIMM50
MEP_NOVERSHTERN_2___311	Aran_et_al_2017	CA1, FMO1, FXN, HDC, NTRK1, PNMT, PRG2, HPGDS, MRPS2, COQ3, CTNNBL1, ACD
MEP_NOVERSHTERN_3___312	Aran_et_al_2017	FCER1A, FMO1, FXN, KRT1, NTRK1, PCCB, PNMT, UNG, IFRD2, AIMP2, CHAF1B, RUVBL1, MRPL28, TMED1, FBXO7, ERAL1, HPGDS, MECR, MRPS2, DDX41, CTNNBL1, C21orf59, EXOSC5, ACD, DCTPP1, NOL12, MTG1
Mesangial_cells_ENCODE_1___313	Aran_et_al_2017	CDH6, CDKN1C, EDN2, FOXF1, FOXD1, HOXA11, HOXD1, LHX1, MICB, TLL2, DOC2B, PADI2, RHOF, KIRREL, CLSTN2, CRISPLD2
Mesangial_cells_ENCODE_2___314	Aran_et_al_2017	CDKN1C, EDN2, FOXD1, HOXA11, HOXD1, LHX1, MICB, TLL2, DOC2B, ARL4C, KIRREL, CLSTN2
Mesangial_cells_ENCODE_3___315	Aran_et_al_2017	CDH6, CDH16, CDKN1C, COL4A1, ARID3A, EDN2, EPHB2, FOXD1, GPI, HOXA3, HOXA11, HOXB2, HOXB3, HOXC10, HOXD1, HOXD11, ILK, INPP5A, ITGA3, ITGB3, LAMA5, LHX1, MICB, NME3, NTHL1, PAX2, PDCD2, PFDN1, PLEC, PVR, HNF1B, NR2F2, TLL2, UBA1, UCP2, UROD, WNT7B, PXDN, DOC2B, RNASET2, FGF18, CLDN6,

		DHRS3, HS3ST3A1, REC8, ARL4C, PROCR, PNPLA6, RBPMS, PLA2G16, MGAT4B, CSDC2, CPA4, ISYNA1, SMOX, RHOF, KIRREL, LAPTM4B, RBM38, CORO1B, CLSTN2, PCYOX1L, GALNT14, PDZD7, BICC1, ORAI2, THAP7, CRISPLD2, APOBEC3F, ASPHD1, HSPA12A
Mesangial_cells_FANTOM_1___316	Aran_et_al_2017	BGN, BST2, CD70, CNN1, COL1A1, COL3A1, COL4A1, COL5A1, COL6A3, CLDN4, VCAN, CTGF, DCN, DPYSL3, ELF3, SLC29A1, EPHB2, F2RL2, FGB, FOXC1, FOXD1, FLNC, FN1, GATA6, GLI2, SFN, HOXA5, HOXA7, HOXA10, HOXA11, HOXB2, HOXB3, HOXB6, HOXB9, HOXD11, IGFBP2, IGFBP4, IGFBP5, ITGB3, KCNJ15, LAD1, LAMA5, LHX1, LIF, SMAD6, MITF, MMP7, MYLK, PDGFB, PDGFRB, ABCB1, PLAT, PODXL, PPARG, PTGER2, PTX3, RAB3B, RGS4, TAGLN, HNF1B, TFPI, TGFB2, TGM2, UCP2, VCAM1, WNT7B, PAX8, HMGA2, DYSF, DOC2B, CLDN6, CLDN1, DIRAS3, IL32, SPOCK2, HS3ST3A1, REC8, LRRC17, DLC1, MYL9, POSTN, PPP1R13L, MMP24, NID2, RAP1GAP2, NUP210, GREM1, ANKRD1, LMCD1, LSR, RHOF, MXRA8, RCN3, ALPK3, HKDC1, NUAK2, CRISPLD2, CREB3L1, LYPD1, OLFML2A
Mesangial_cells_FANTOM_2___317	Aran_et_al_2017	CD70, F2RL2, FGB, FOXC2, HOXA3, HOXA7, HOXA10, HOXA11, HOXB8, HOXB9, HOXD10, HOXD11, PAX2, ABCB1, PODXL, RAB3B, SALL1, HNF1B, UCP2, PAX8, DOC2B, CLDN6, REC8, B3GALT5, IFITM2, NUP210, RHOF, C14orf105, CDHR1
Mesangial_cells_FANTOM_3___318	Aran_et_al_2017	ACTA2, ADORA1, AEBP1, ARHGAP4, ATP7B, BGN, BMP4, CD70, CDH6, CNN1, COL1A1, COL1A2, COL3A1, COL5A1, COL6A1, COL6A2, COL6A3, CLDN4, CLDN3, CRIP1, CYBA, DCN, DNM1, DPYSL3, EDN2, ELF3, ENPEP, SLC29A1, F2RL2, FGB, FOXC1, FOXD1, FOXC2, FN1, FZD2, GATA6, GLI2, CCR10, GRB14, HOXA3, HOXA4, HOXA6, HOXA7, HOXA10, HOXA11, HOXB6, HOXB8, HOXB9, HOXC10, HOXD9, HOXD10, HOXD11, HPGD, HSPA2, IGFBP4, ITGB3, KRT19, LFNG, LHX1, SMAD6, MEIS2, MGP, MITF, MMP7, NCAM1, NOV, NPPB, OXTR, PAPPA, PAX2, PCOLCE, PDGFB, PDGFRB, ABCB1, PLAT, PODXL, PPARG, PTGER2, PTPRJ, RAB3B, RGS4, RRAD, SALL1, SDC2, SIX1, SLC22A3, SPP1, SST, TAGLN, TBL1X, TBXAS1, HNF1B, NR2F1, TGFB1I1, TGFB2, TRPC4, UCP2, WT1, PAX8, DYSF, DOC2B, ADAM19, TNFSF10, PROM1, CACNA1H, CLIC3, CLDN6, CLDN1, DIRAS3, IL32, PDLIM7, RPH3AL, ADAMTS3, GAL3ST1, SPOCK2, KBTBD11, HS3ST3A1, REC8, ARL4C, MSLN, B3GALT5, DLC1, MYL9, IFITM2, POSTN, SIX2, PLK2, MMP24, RBPMS, PLA2G16, NID2, DENND3, PLXND1, SULF1, NUP210, QPRT, OLFML2B, GREM1, CPNE7, HIPK2, TRHDE, EFEMP2, ZNF580, CPA4, SHC3, FXYD6, RHOF, HCFC1R1, C14orf105, LRRC20, KIRREL, GPRC5C, OLFML3, SDR39U1, RCN3, ALPK3, SYT13, GALNT14, DNAJC22, BICC1, HKDC1, RAB11FIP1, COL18A1, LBH, CRISPLD2, CREB3L1, CDHR1, ATP6V0E2, CADM4
Monocytes_BLUEPRINT_1___319	Aran_et_al_2017	ASGR2, FCN1, CFP, RNASE2, CD300C, LILRA1, TLR7, MS4A6A, LILRA5, CCR2
Monocytes_BLUEPRINT_2___320	Aran_et_al_2017	AIF1, ASGR2, CYBB, FCAR, FCN1, KCNMB1, MEFV, MNDA, CFP, S100A12, CD163, CD101, CLEC5A, FBXL5, TREM1, RETN, CCR2
Monocytes_BLUEPRINT_3___321	Aran_et_al_2017	ASGR2, CSF3R, F13A1, FCN1, CFP, RNASE2, S100A12, LILRB2, CD93, PADI4, P2RY13, MS4A6A
Monocytes_FANTOM_1___322	Aran_et_al_2017	AIF1, APAF1, RHOA, RHOG, ARNT, ASGR2, C3AR1, CASP5, TNFRSF8, CD33, CSF1R, CSF3R, CYBB, FCAR, FCN1, FPR2, HCK, HK3, HRH2, HSPA6, KCNMB1, MEFV, MAP3K3, MNDA, MYO1F, CFP, PHKG2, PTGIR, RARA, S100A12, TYROBP, UBE2D1, UPK3A, VASP, BEST1, LST1, NUP214, IQGAP1, ATP6V0D1, CD163, CD101, EIF4E2, CALCOCO2, LILRB2, TGOLN2, CAMKK2, LILRB1, CD300C, FGL2, LILRA1, LILRB3, LILRA2, TREX1, GABARAP, RPH3A, ACAP2, CLEC5A, IL17RA, FBXL5, CLEC4E, VENTX, COMMD9, PILRA, METTL9, TLR7, TLR8, P2RY13, TREM1, RIN2, RHOT1, TMEM127, TMEM9B, RETN, RPGRIP1, DENND1A, DPEP2, MS4A6A, OSBPL11, LILRA5, CCR2
Monocytes_FANTOM_2___323	Aran_et_al_2017	ABCB7, AIF1, ANXA1, ARF5, ASGR2, BPI, BTK, TSPO, C3AR1, CAPN2, CAPN3, CAPNS1, CAST, CD4, CD33, CEACAM4, MAPK14, CSF1R, CSF3R, CTBP2, CX3CR1, CYBB, DHX8, F13A1, FCAR, FCER1G, FCN1, FGR, FOLR2, FPR2, HADHA, HCK, HK3, AGFG1, HSPA6, CXCR2, IL10RA, IMPDH1, KCNMB1, LTBR, LYL1, MAN2C1, MARK3, MAP3K3, MAP3K11, MNDA, MNT, MYO1F, NUBP1, NCF4, CFP, PLP2, PPP1CB, PRKACA, PTEN, PTGIR, RARA, RNASE2, S100A6, S100A10, S100A12, SH3BP2, SLC11A1, NEK4, CLEC3B, TPD52L2, UBE2D1, USP4, BEST1, WAS, LST1, NUP214, NCOA4, NDST2, PIAS1, MARCO, TMEM11, MTMR3, BTAF1, DOK2, PSTPIP1, CD163, CD101, QKI, LY86, EIF4E2, H2AFY, PPM1F, SNX17, GIT2, USP15, COL4A3BP, CALCOCO2, RGS19, LILRB2, RTN3, CEPT1, CLEC10A, CAMKK2, LILRB1, CD300C, LILRA1, LILRB3, LILRA2, OGFR, WWP2, AKAP13, TREX1, GABARAP, RPH3A, CD93, SPEN, SETX, KDM6B, STAB1, KLHL18, ANKS1A, UBR2, SIN3B, DNAJC13, KIAA1033, SUN1, SIK3, MED13L, SEC11A, ACAP2, PADI4, STX12, IL17RA, PGLS, FAM32A, FBXL5, CLEC4E, PTPN18, SIGLEC9, COQ2, VENTX, CECR5, NKIRAS2, COMMD9, STRN4, PILRA, CLEC4A, METTL9, TLR7, ZDHHC3, TLR8, TMBIM4, P2RY13, TREM1, RIN2, RBM41, RHOT1, NPLOC4, WDR11, NSFL1C, RETN, DENND1A, PCTP, DPEP2,

		MS4A6A, MTMR14, ATG3, AHNAK, TSEN34, MBOAT7, CARS2, DOK3, PANK2, FBXO11, SPG11, CXorf21, DHX57, SLC38A10, UBXN2B, HIPK1, YTHDF3, LILRA5, CCR2
Monocytes_FANTOM_3___324	Aran_et_al_2017	ABCB7, AIF1, ANXA1, ARF5, ASGR2, BPI, BTK, TSPO, C3AR1, CAPN2, CAPN3, CAPNS1, CAST, CD4, CD33, CEACAM4, MAPK14, CSF1R, CSF3R, CTBP2, CX3CR1, CYBB, DHX8, F13A1, FCAR, FCER1G, FCN1, FGR, FPR2, HCK, HK3, AGFG1, HSPA6, IL10RA, IMPDH1, KCNMB1, LTBR, LYL1, MAN2C1, MARK3, MAP3K3, MAP3K11, MNDA, MNT, MYO1F, CFP, PLP2, PPP1CB, PRKACA, PTEN, RARA, RNASE2, S100A6, S100A10, S100A12, SH3BP2, CLEC3B, TPD52L2, UBE2D1, USP4, BEST1, WAS, LST1, NUP214, NCOA4, CUL5, NDST2, PIAS1, MARCO, TMEM11, MTMR3, BTAF1, DOK2, PSTPIP1, ZMYM4, CD163, CD101, LY86, EIF4E2, H2AFY, PPM1F, SNX17, GIT2, USP15, CALCOCO2, RGS19, LILRB2, RTN3, CLEC10A, CAMKK2, LILRB1, CD300C, LILRA1, LILRB3, LILRA2, OGFR, WWP2, AKAP13, TREX1, GABARAP, RPH3A, CD93, SPEN, KDM6B, STAB1, KLHL18, ANKS1A, SIN3B, DNAJC13, KIAA1033, SUN1, SIK3, SEC11A, ACAP2, PADI4, STX12, PGLS, FAM32A, FBXL5, PTPN18, SIGLEC9, COQ2, VENTX, CECR5, NKIRAS2, COMMD9, STRN4, PILRA, CLEC4A, METTL9, TLR7, TLR8, P2RY13, TREM1, RIN2, RBM41, RHOT1, NPLOC4, WDR11, NSFL1C, RETN, DENND1A, PCTP, DPEP2, MS4A6A, MTMR14, ATG3, AHNAK, TSEN34, MBOAT7, CARS2, DOK3, PANK2, FBXO11, SPG11, USP48, DHX57, SLC38A10, PIKFYVE, HIPK1, JMJD1C, LILRA5, CCR2
Monocytes_HPCA_1___325	Aran_et_al_2017	CSNK1A1, FCN1, FGR, HUS1, NUBP1, PNP, CFP, SH3BP2, SRC, LRRFIP1, LILRB2, EIF1B, FNDC3A, FKBP15, CLEC5A, RPS6KC1, CD244, ARL8B, RETN
Monocytes_HPCA_2___326	Aran_et_al_2017	CASP5, CD48, CSNK1A1, FCN1, FGR, FPR2, MEFV, PNP, CFP, LILRA1, FKBP15, CLEC5A, SIGLEC9, ARL8B, RETN
Monocytes_HPCA_3___327	Aran_et_al_2017	AP1G1, ASGR2, BNIP2, TSPO, CASP5, CD1E, TNFRSF8, CD33, CDK9, CSF1R, DDX3X, DHX8, DLG4, GPR183, ETF1, ETV3, EWSR1, FCAR, FCN1, FOLR2, FOLR3, FPR2, GALNT3, HIF1A, HNRNPU, AGFG1, KCNC3, KCNMB1, MEFV, MMP17, MTF1, MTHFR, OSM, PDE6H, CFP, PGGT1B, PLD2, PLEK, POU2F2, PPM1A, PRKACA, MAPK6, MAP2K1, PTGIR, RAB5A, RELA, CLIP1, S100A10, S100A12, SH3BP2, SLC11A1, STX5, SUPT6H, PHLDA2, UBE2D1, UPK3A, PTP4A2, ELL, NDST2, DNAH17, PTCH2, RIOK3, KSR1, BCL10, SOCS3, USP8, DDX21, PLAA, CD101, QKI, WTAP, AATK, TTLL4, BCL2L11, MPHOSPH6, AKAP8, LILRB2, APBB3, CLEC10A, GNA13, MAP3K2, LILRB1, CD300C, FGL2, LILRA1, OGFR, AKAP13, TREX1, CD93, IQSEC2, STAB1, JMJD6, CBX6, CLEC5A, PGLS, GGA1, FBXL5, CLEC4E, SERP1, GNMT, GPR162, VENTX, RABGEF1, TBK1, STRN4, CLEC4A, ADIPOR1, METTL9, RLIM, DCTN4, CLEC1A, ZBTB7A, CDC40, AZIN1, P2RY13, TREM1, MIOS, TMEM104, VNN3, SAR1A, CYSLTR2, DENND1A, SAMSN1, MS4A6A, MTMR14, ZNF668, GRPEL1, REEP4, ZNF787, ZFC3H1, YTHDF3, LILRA5, ZNF710, TMEM110
Monocytes_IRIS_1___328	Aran_et_al_2017	ASGR2, TNFRSF8, FCAR, FCN1, FOLR2, HIC1, MEFV, CFP, S100A12, SLC11A1, FBXL5, VENTX, RABGEF1, TREM1, VNN3, SAMSN1, MS4A6A, LILRA5
Monocytes_IRIS_2___329	Aran_et_al_2017	ASGR2, FCAR, FCN1, S100A12, UPK3A, VENTX, TREM1, VNN3, MS4A6A, LILRA5
Monocytes_IRIS_3___330	Aran_et_al_2017	ASGR2, CASP5, TNFRSF8, FCAR, FCN1, FOLR2, HIC1, HIF1A, MEFV, MMP17, OSM, CFP, PTGIR, S100A12, SLC11A1, UPK3A, RIOK3, SOCS3, SEMA6B, GNA13, FGL2, STAB1, PADI4, CLEC5A, FBXL5, VENTX, RABGEF1, METTL9, P2RY13, TREM1, VNN3, DENND1A, SAMSN1, MS4A6A, LILRA5
Monocytes_NOVERSHTERN_1___331	Aran_et_al_2017	AIF1, ANXA1, ASGR2, BPI, BTK, TSPO, C3AR1, CAPN3, CAST, CD4, CD33, CEACAM4, MAPK14, CSF3R, CTBP2, CX3CR1, CYBB, F13A1, FCAR, FCER1A, FCN1, FGR, FPR2, HCK, HK3, HSPA6, CXCR2, KCNMB1, LTBR, LYL1, MNDA, MYO1F, NCF4, CFP, PLP2, PTEN, RNASE2, S100A12, NEK4, CLEC3B, UBE2D1, LST1, NUP214, MARCO, DOK2, PSTPIP1, CD163, CD101, LY86, PPM1F, GIT2, USP15, RGS19, LILRB2, RTN3, CLEC10A, LILRB1, CD300C, LILRA1, LILRB3, LILRA2, CD93, STAB1, ANKS1A, ACAP2, PADI4, PGLS, FBXL5, PTPN18, COQ2, COMMD9, PILRA, CLEC4A, METTL9, TLR7, TLR8, MS4A4A, P2RY13, TREM1, RHOT1, RETN, PCTP, DPEP2, MS4A6A, ATG3, AHNAK, TSEN34, CARS2, UBXN2B, LILRA5, CCR2
Monocytes_NOVERSHTERN_2___332	Aran_et_al_2017	ASGR2, FCAR, FCN1, FOLR2, MEFV, CFP, S100A12, SLC11A1, UPK3A, FBXL5, VENTX, TREM1, VNN3, MS4A6A
Monocytes_NOVERSHTERN_3___333	Aran_et_al_2017	ASGR2, FCAR, FCN1, FOLR2, MEFV, CFP, S100A12, SLC11A1, UPK3A, VENTX, TREM1, VNN3, MS4A6A
MPP_BLUEPRINT_1___334	Aran_et_al_2017	ADCY3, CRHBP, ERG, CAPRIN1, P2RX1, SERPINI2, TEC, TIE1, UMPS, MLF2, ZNF282, NSMAF, USP6, MED7, H2AFY, PPM1F, PARP2, MRPS31, CALCOCO2, TMEM147, SF3A3, CSTF2T, LSM5, ATXN10, HIBCH, ATP2C1, ZNF219, TAOK3, PTRH2, TBC1D13, TMEM70, ATP5SL, WDR60, DPPA4, AGK, MKL2, BAHCC1, ALG8, CENPO, SPAG16, UBA5, SFXN3, FAM136A, G6PC3, HNRNPA3, DPY19L4

MPP_BLUEPRINT_2___335	Aran_et_al_2017	ABO, AIF1, AMD1, ATP5J, AVP, BTF3, ERCC8, CLNS1A, CPA3, CRHBP, CRYGD, DNTT, DUT, FANCA, HINT1, IGLL1, EIF3E, ITGA9, LRCH4, MPL, MPO, NACA, NFYA, NPM1, NUP98, PIGF, PRTN3, PSMA2, PSMA6, RAG2, RBBP7, RFC2, RNASE2, RNASE3, RPS3, RPS24, SMARCC2, SNRPF, TEC, TERT, TRH, TSSC1, TTF1, ZNF32, ZNF35, ZNF134, CD164, GALR2, CDC123, MED7, PMPCB, TATDN2, KIAA0125, EIF1B, PPIH, ANP32B, ERLIN1, AKAP13, ESYT1, HAUS5, NAT6, ATP2C1, INVS, LSM1, BZW2, GLTSCR2, RSL24D1, ZNF639, REV1, TEX10, SRBD1, NHP2, PCID2, NSFL1C, TMEM9B, TFB2M, GGCT, MAP7D3, MTDH, IMP4
MPP_BLUEPRINT_3___336	Aran_et_al_2017	ABO, AMD1, ATP5J, AVP, BTF3, ERCC8, CLNS1A, CRHBP, CRYGD, DUT, FANCA, HINT1, IGBP1, IGLL1, IMPDH2, EIF3E, ITGA9, LRCH4, MPL, MPO, NACA, NUP98, PIGF, PSMA2, PSMA6, RBBP7, RFC2, RNASE2, RPS3, RPS24, SNRPF, TERT, ZNF32, ZNF134, GALR2, MED7, TATDN2, KIAA0125, NUBP2, PPIH, AKAP13, ESYT1, HAUS5, INVS, LSM1, BZW2, RSL24D1, ZNF639, TEX10, SRBD1, NHP2, PCID2, NSFL1C, TMEM9B, TFB2M, ZDHHC6, GGCT, MAP7D3, CSPP1, FAM136A, MTDH, IMP4
MPP_ENCODE_1___337	Aran_et_al_2017	AVP, AZU1, CRHBP, DNTT, FLT3, IGLL1, ITGA9, MPL, MPO, NUP98, RNASE2, SPN, CD164, KIAA0125, LSM1, VPREB3
MPP_ENCODE_2___338	Aran_et_al_2017	AVP, CRHBP, FLT3, IGLL1, MPL, MPO, KIAA0125, VPREB3
MPP_ENCODE_3___339	Aran_et_al_2017	AVP, CRHBP, FLT3, IGLL1, MPL, MPO, KIAA0125, VPREB3
MPP_FANTOM_1___340	Aran_et_al_2017	ABO, AVP, CD37, CD52, CPA3, CRHBP, CSF2RB, CSF3R, CTSW, FCER1A, FLT3, GATA1, HBB, HBD, HDC, HLA-DOA, IGLL1, ITGA2B, ITGA9, ITGAL, JAK3, CIITA, MPL, MPO, NKG7, OSM, P2RX1, PIK3CG, PLCB2, PRKCB, PRTN3, PTPN6, PTPN7, PTPRC, PTPRCAP, SELPLG, SPN, GFI1B, S1PR4, BAIAP3, ACAP1, KIAA0125, TSPAN32, IKZF1, KLF1, MLC1, CD244, DPPA4, ARHGAP15, DPEP2, DOK3, TRAF3IP3, AGAP2
MPP_FANTOM_2___341	Aran_et_al_2017	ABO, AVP, FMNL1, CD37, CD52, CPA3, CRHBP, CSF2RB, CSF3R, CTSW, FCER1A, FLT3, GATA1, HBB, HBD, HDC, HLA-DOA, IGLL1, IRF5, ITGA2B, ITGA9, ITGAL, JAK3, CIITA, MPL, MPO, NKG7, OSM, P2RX1, PIK3CG, PLCB2, PRKCB, PRTN3, PTPN6, PTPN7, PTPRC, PTPRCAP, SELPLG, SPN, LST1, GFI1B, S1PR4, BAIAP3, ACAP1, KIAA0125, TSPAN32, IKZF1, KLF1, CD300A, MLC1, CD244, SASH3, DPPA4, ARHGAP15, DPEP2, ATP8B4, DOK3, TRAF3IP3, AGAP2
MPP_FANTOM_3___342	Aran_et_al_2017	AVP, CD37, CD53, CPA3, CRHBP, CSF3R, CTSW, FCER1A, HBD, NCKAP1L, HLA-DOA, IGLL1, ITGA2B, LAIR1, MPO, MYO1F, NCF4, OSM, P2RX1, PTPN7, PTPRC, PTPRCAP, SPI1, SPN, GFI1B, S1PR4, KIAA0125, TSPAN32, IKZF1, MLC1, SASH3, AGAP2
MPP_NOVERSHTERN_1___343	Aran_et_al_2017	AVP, CRHBP, FLT3, IGLL1, MPL, MPO, NUP98, KIAA0125, ESYT1, VPREB3
MPP_NOVERSHTERN_2___344	Aran_et_al_2017	AVP, CRHBP, FLT3, IGLL1, MPL, MPO, KIAA0125, VPREB3
MPP_NOVERSHTERN_3___345	Aran_et_al_2017	AVP, CRHBP, FLT3, IGLL1, MPL, MPO, KIAA0125, VPREB3
MSC_FANTOM_1___346	Aran_et_al_2017	HTR7, MMP17, PLA2G5, CDKL5, PKD2L1, ADAMTS12, ZNRF4, CTRB2
MSC_FANTOM_2___347	Aran_et_al_2017	HTR7, MMP17, PRB3, CCL21, CDKL5, TNP2, PKD2L1, ZNF408, ADAMTS12, ZNRF4, CTRB2
MSC_FANTOM_3___348	Aran_et_al_2017	COL10A1, DVL1, HAS1, HTR7, MMP17, NPAS1, PLA2G5, PRB3, CDKL5, TNP2, WISP1, PKD2L1, TRIM3, COPS8, ADAMTS12, TSSK1B, ZNRF4, CTRB2
MSC_HPCA_1___349	Aran_et_al_2017	DCTD, DDOST, EEF1D, KIF22, LAMP1, NDUFA8, PARN, POLR2G, SLC35A2, HAND2, EIF4E2, CUL7, SCAMP3, STUB1, TMEM147, YIF1A, RER1, EMILIN1, SNF8, CABIN1, MKRN2, SPCS1, TRMT112, ZNF446, PRX, MRPS11, CUEDC2, SPAG16, MRPL24, TBC1D17, NHEJ1, PODNL1, SF3B5, THAP3, SLC38A10, SIX5
MSC_HPCA_2___350	Aran_et_al_2017	DCTD, EEF1D, KIF22, POLR2G, HAND2, EIF4E2, CUL7, SCAMP3, YIF1A, RER1, EMILIN1, CABIN1, TRMT112, PRX, MRPL24, NHEJ1, PODNL1, SF3B5, THAP3
MSC_HPCA_3___351	Aran_et_al_2017	ATP6V1C1, CYC1, DCTD, DDOST, EEF1D, ERCC1, GSTM5, HIC1, IMPDH1, KIF22, LAMP1, MDH2, NDUFA8, NDUFB4, NFATC4, PARN, POLR2G, PTPN11, RPL8, RPN1, SLC35A2, AAAS, TMEM11, HAND2, EIF4E2, BAG3, RNF7, SNX17, CUL7, SCAMP3, RNF41, STUB1, TMEM147, TIMM44, VTI1B, YIF1A, KDELR1, LMAN2, RER1, EMILIN1, SNF8, COPZ1, HMGXB3, CLUAP1, IQSEC2, PMPCA, CABIN1, MKRN2, LMOD1, TMEM184B, TCTN3, TIMM10, SPCS1, MRPS18B, GMPPA, STOML2, CYHR1, ARL6IP4, ZNF771, MRPS17, TRMT112, CUTA, TMEM161A, COMMD4, CPSF3L, OGFOD1, LRRC59, ZNF446, PRX, MRPS11, CUEDC2, C7orf26, TMEM223, SPAG16, MRPL24, TBC1D17, NHEJ1, PODNL1, INTS5, SF3B5, MFSD5, THAP3, SLC38A10, SIX5, GTF2H5

mv_Endothelial_cells_ENCODE_1___352	Aran_et_al_2017	ACVRL1, ANGPT2, SLC25A6, RHOC, TSPO, CAV2, CCNG1, CD34, CETP, COX4I1, CSNK2A2, DYNC1LI2, ECHS1, FDPS, FLT4, GJA4, GOLGA3, GOT2, HADHA, HCFC1, HCRTR1, HOXD3, ILF2, ITGA9, KDR, LGALS1, NOTCH4, NOVA2, PLXNB3, MAPK3, PRPSAP1, PSMB7, PSMC5, PSMD8, RALA, RANGAP1, MAPK12, SELE, SH3GL1, SMARCD1, SMARCE1, SUMO3, SNAPC4, SNTB2, TARBP2, TBX1, TEK, TIE1, CLDN5, TPM3, UBE2E1, UFD1L, VWF, YWHAE, ZNF205, CLPP, ZNF282, SCARF1, HYAL2, MBTPS1, PABPC4, RGS11, BANF1, EIF2B2, MTMR2, BCL10, TNFSF18, DYRK1B, COX7A2L, SLC24A1, GTF3C5, VAMP3, TAOK2, EI24, PPM1F, DHX38, KEAP1, MED16, NUBP2, ACTR1A, TXNDC9, RAMP3, PCGF3, LYPLA1, STK25, SEMA6B, DCTN2, HTATIP2, GIPC1, LYVE1, RNPS1, COPS6, RAB35, TMEM115, STRAP, ATXN2L, LYPLA2, TUSC2, RRAS2, ARHGEF15, RALY, TTLL5, N4BP3, STAB1, ZC3H7B, SUN1, ABCB9, KCTD2, DNPEP, EDC4, MTCH1, MTCH2, PRKD2, GPKOW, CECR5, MRPS28, GIT1, MRPL15, CNIH4, F11R, MRPS16, CLEC1A, BFAR, GMPR2, PCDH12, PIAS4, FAM96B, HSPB11, EMCN, SOX18, ROBO4, NDUFB11, DEF8, CPSF3L, EXD2, CHST12, LMBR1L, UBAP2, CISD1, METTL3, INPP5E, CIAPIN1, FN3K, MMS19, RNF25, WDR13, TUT1, MRPL9, SPATS2, DDA1, DCTPP1, FAM65A, CXorf36, GEMIN7, MMRN2, FAM124B, EDC3, MYCT1, GFOD2, URM1, FRMD8, MTG1, FTSJ3, SAMD14, ZDHHC24, KANK3
mv_Endothelial_cells_ENCODE_2___353	Aran_et_al_2017	ACVRL1, ANGPT2, SLC25A6, RHOC, ATP1B3, BYSL, TSPO, CAV2, CD34, CDC27, CETP, CLIC1, COPA, COX4I1, CSNK2A2, DYNC1LI2, FDPS, FLT4, GDI2, GOT2, GYG1, HCFC1, HCRTR1, HOXD3, ILF2, KDR, LAMP1, LGALS1, MTHFR, MYL6, NAP1L4, NDUFC2, NOTCH4, NOVA2, PLD2, PLXNB3, POLR2F, MAPK3, PRPSAP1, PSMB7, PSMC5, ABCD4, RALA, RANGAP1, RPL4, RPN2, MAPK12, SELE, SH3GL1, SMARCD1, SMARCE1, SNAPC4, SNTB2, SSBP1, TARBP2, TBX1, TEK, TIAL1, TIE1, CLDN5, TPM3, UFD1L, YWHAE, ZFPL1, CLPP, ZNF282, SCARF1, HYAL2, MBTPS1, PABPC4, EIF2B2, MTMR2, TNFSF18, DYRK1B, COX7A2L, SLC24A1, TAOK2, TXNL1, EIF4E2, DHX38, KEAP1, ACTR1A, TXNDC9, DCAF7, RAMP3, PCGF3, LYPLA1, STK25, KAT5, DCTN2, HTATIP2, GIPC1, FRS3, LYVE1, COPS6, ATF7, RAB35, TMEM115, STRAP, TUSC2, RRAS2, ARHGEF15, TTLL5, N4BP3, ZC3H7B, EXOC7, SIN3B, ABCB9, KCTD2, EDC4, STX12, MTCH1, MTCH2, PGLS, PRKD2, TBC1D10B, FBXO22, GPKOW, AHDC1, EIF3K, CECR5, MRPS28, GIT1, MRPL15, CNIH4, UBIAD1, F11R, MRPS16, UBXN1, THAP4, TACO1, CLEC1A, BFAR, GMPR2, PCDH12, PIAS4, FAM96B, HSPB11, EMCN, INPP5K, SOX18, ROBO4, NDUFB11, GPN2, DEF8, CPSF3L, C19orf24, ZWILCH, TMEM39B, EXD2, CHST12, LMBR1L, UBAP2, CISD1, METTL3, INPP5E, CIAPIN1, ENOPH1, CCDC90B, FN3K, MOSPD3, WDR13, ELOVL1, SPATS2, FAM65A, MUL1, CXorf36, GEMIN7, GSDMD, MMRN2, FAM124B, EDC3, MYCT1, URM1, FRMD8, FTSJ3, TOR1AIP2, SAMD14, SENP5, ZDHHC24, KANK3, TIPRL
mv_Endothelial_cells_ENCODE_3___354	Aran_et_al_2017	ACVRL1, ANGPT2, SLC25A6, RHOC, CAV2, CD34, CETP, COX4I1, CSNK2A2, DYNC1LI2, FDPS, FLT4, GJA4, GOLGA3, GOT2, HADHA, HCFC1, HCRTR1, HOXD3, ILF2, ITGA9, KDR, LGALS1, NOTCH4, NOVA2, PLXNB3, MAPK3, PRPSAP1, PSMB7, PSMC5, RALA, RANGAP1, MAPK12, SELE, SH3GL1, SMARCD1, SMARCE1, SUMO3, SNAPC4, SNTB2, TARBP2, TBX1, TEK, TIE1, CLDN5, TPM3, UFD1L, VWF, ZNF205, CLPP, ZNF282, HYAL2, RGS11, EIF2B2, BCL10, TNFSF18, DYRK1B, SLC24A1, GTF3C5, TAOK2, EI24, PPM1F, KEAP1, MED16, RAMP3, PCGF3, LYPLA1, STK25, SEMA6B, HTATIP2, GIPC1, LYVE1, COPS6, RAB35, TMEM115, STRAP, ATXN2L, LYPLA2, TUSC2, RRAS2, ARHGEF15, RALY, TTLL5, N4BP3, STAB1, ZC3H7B, ABCB9, KCTD2, EDC4, MTCH1, MTCH2, PRKD2, CECR5, MRPS28, GIT1, MRPL15, F11R, MRPS16, CLEC1A, BFAR, GMPR2, PCDH12, HSPB11, EMCN, SOX18, ROBO4, NDUFB11, CPSF3L, CHST12, LMBR1L, CISD1, METTL3, CIAPIN1, FN3K, WDR13, TUT1, MRPL9, SPATS2, DDA1, DCTPP1, FAM65A, CXorf36, GEMIN7, MMRN2, FAM124B, EDC3, MYCT1, GFOD2, URM1, FRMD8, MTG1, FTSJ3, SAMD14, KANK3
mv_Endothelial_cells_FANTOM_1___355	Aran_et_al_2017	ACVRL1, ANGPT2, ART4, ARVCF, BMX, CALM1, CASP10, CAV1, CD9, CD34, ENTPD1, CDK9, CETP, CSF2RB, CTNNA1, ACE, ELK4, ERG, ERH, FLOT2, FLT1, FLT4, GABPB1, HDAC1, HOXD3, HTR1B, IL3RA, KDR, LYL1, MAP3K3, MGAT5, NCK1, NOTCH4, NOVA2, PNP, PDCL, PIK3CG, PLCG1, PRPSAP1, RALA, RALB, SELE, TAL1, TBX1, TEK, TIE1, CLDN5, UFD1L, VWF, ZNF22, SCARF1, HYAL2, BCL10, HERC1, ATP6V0E1, TNFSF18, LRRFIP1, TAOK2, PPM1F, RAMP3, SEMA6C, SEMA6B, HTATIP2, MYL12A, LYVE1, TFEC, ARHGEF15, MMRN1, CD93, ATF6, N4BP3, STAB1, TDRD7, TSPAN13, MAT2B, CLEC1A, PCDH12, SPTBN5, EMCN, SOX18, ROBO4, RASIP1, TMEM39B, GIMAP4, LSG1, NECAP2, LMBR1L, ANO2, KIF17, TMEM109, CXorf36, MMRN2, FAM124B, MYCT1, RNF34, GFOD2, PLVAP, CEACAM21, KANK3, GIMAP6
mv_Endothelial_cells_FANTOM_2___356	Aran_et_al_2017	ACVRL1, ANGPT2, ARVCF, BMX, CASP10, CAV1, CD9, CD34, ENTPD1, CETP, CSF2RB, ACE, ERG, FLT1, FLT4, HTR1B, IL3RA, KDR, LYL1, MAP3K3, NOTCH4, PNP, NPR1, TAL1, TBX1, TEK, TIE1, CLDN5, VWF, SCARF1, HYAL2, TNFSF18, TAOK2, PPM1F, RAMP3, SEMA6B, LYVE1, TFEC, ARHGEF15, MMRN1, CD93, ATF6, N4BP3, STAB1,

		CLEC1A, PCDH12, EMCN, SOX18, ROBO4, RASIP1, GIMAP4, ANO2, KIF17, CXorf36, MMRN2, FAM124B, MYCT1, KANK3, GIMAP6
mv_Endothelial_cells_FANTOM_3___357	Aran_et_al_2017	ACTG1, ADRA1B, ANXA2, ARF1, RHOA, RHOC, ATP2B3, BMX, PTTG1IP, CAV1, CCKAR, CD34, AP2M1, CLTA, CPA1, CTNNA1, DAD1, DLST, DYNC1H1, EIF4G1, FOXC2, GJA4, GPR4, HSPA4, HSP90AB1, MAGEB1, MIF, MIP, MYL6, 44076, NEDD8, NOTCH4, OXA1L, P4HB, PLS3, POLR2J, PPP2R1A, PPP2R2A, MAPK3, PSMB7, PSMD1, PSMD10, RALA, RCN2, SLC6A7, TAF12, TJP1, CLDN5, DNAJC7, UFD1L, VWF, KCNAB1, USP5, CLPP, AKAP4, EIF2B2, TAOK2, MED20, SAE1, ABCF2, ACTR1A, B3GALT5, CLEC4M, PCGF3, SEMA6B, GLRX3, ARPC1A, YKT6, ACTL7A, COPS6, TMEM115, CAPN11, PWP1, CDC37, FAM107A, STRAP, LYPLA2, ECD, TUSC2, NCBP2, CD93, GANAB, PMPCA, NUP188, CLDN14, ARL2BP, PRND, PITPNB, MTCH1, TRPC4AP, NOC2L, FOXD3, TRAPPC3, SEC61A1, CHCHD2, PCDH12, SPTBN5, SNTG2, DDX56, TMED9, FNDC8, COMMD4, C1orf123, AURKAIP1, UNC45A, PCDHA6, C16orf62, LYZL6, MRPL17, MMRN2, FAM124B, LRRC3, DCTN5, YIF1B, POM121L2
mv_Endothelial_cells_HPCA_1___358	Aran_et_al_2017	ACVRL1, CETP, RANGAP1, SELE, TIE1, CLDN5, VWF, HYAL2, EIF2B2, TNFSF18, LYVE1, ARHGEF15, CLEC1A, ROBO4, CXorf36, MMRN2, KANK3
mv_Endothelial_cells_HPCA_2___359	Aran_et_al_2017	ACVRL1, CETP, FLT4, HCRTR1, KDR, NOVA2, RALA, RANGAP1, SELE, TEK, TIE1, CLDN5, TPM3, VWF, HYAL2, EIF2B2, TNFSF18, LYVE1, ARHGEF15, CLEC1A, PCDH12, SOX18, ROBO4, TUT1, FAM65A, CXorf36, MMRN2, MYCT1, KANK3
mv_Endothelial_cells_HPCA_3___360	Aran_et_al_2017	ACVRL1, CETP, FLT4, RANGAP1, SELE, TIE1, CLDN5, VWF, HYAL2, EIF2B2, TNFSF18, LYVE1, ARHGEF15, CLEC1A, SOX18, ROBO4, TUT1, CXorf36, MMRN2, KANK3
Myocytes_ENCODE_1___361	Aran_et_al_2017	EVC, SMAD5, MUSK, SGCA, SIM1, BAG2, SHQ1, IMPACT, EXOC1, KRTAP1-1
Myocytes_ENCODE_2___362	Aran_et_al_2017	COPB1, EVC, SMAD5, MUSK, MYF5, SGCA, SIM1, MAP3K7, DENR, BAG2, SHQ1, IMPACT, EXOC1, XPNPEP3, KRTAP1-1, PRRC1
Myocytes_ENCODE_3___363	Aran_et_al_2017	ACTG1, ALDOA, ANXA5, ARF4, CAD, CAST, CAV1, CAV3, CCNG1, CCT6A, CDH15, CHRNG, CLTC, COPB1, COX8A, CSNK1G3, DCTD, DDOST, TOR1A, EIF4EBP2, EPRS, ERCC4, EVC, GDI2, GLE1, GNAS, GRSF1, GTF2H1, HIF1A, HRC, HSPA8, IDUA, KIF2A, TNPO1, LAMP1, LGALS1, SMAD2, SMAD5, MIF, MKLN1, MMP11, MSH3, MUSK, MYBPH, MYF5, MYOD1, NDUFA10, 44076, NONO, PAFAH1B1, PARN, PCDHGC3, SLC25A3, PLXNB3, POLR2J, PSMB4, PTPN11, RAPSN, RCN2, RPN2, SGCA, FBXW4, SIM1, SPG7, SSR4, SS18, STAU1, MAP3K7, TIAL1, UBE3A, COL14A1, ZNF37A, ZNF221, ZNF214, RAB7A, DENR, WISP1, EIF2S2, USP14, BAG5, BAG2, PREPL, SNX17, UBAP2L, UBA2, RAD50, LRPPRC, MPHOSPH6, ZMPSTE24, BCKDK, YAP1, CDIPT, TMEM147, IPO7, PTGES3, FRS2, CPSF4, COPS8, SPIN1, AFG3L2, HNRNPA0, ERP29, METAP2, XPOT, HMGXB3, CLUAP1, POFUT2, DNAJC16, HARS2, POFUT1, ARL2BP, MKRN2, TXN2, IBTK, TCTN3, FBXL4, MYOF, RPS6KC1, RNF11, NPTN, SND1, HSPB7, TUBG2, VPS4A, NFU1, UTP20, MCTS1, MYLPF, EPN1, SEC61A1, MRPS16, IFT52, METTL9, MBTPS2, UCHL5, RWDD1, AMOTL2, ERGIC3, UBE2D4, CMPK1, NUDT9, NUP54, GPR173, RC3H2, CHCHD3, FAM120C, ZDHHC4, SHQ1, SLC38A7, ADI1, QRSL1, IMPACT, NOP10, HIF1AN, ZNF446, PEX26, IARS2, EXOC1, SPATA7, ACTR10, DNAH7, SPPL2B, PRX, EDA2R, CCDC90B, GUF1, PKNOX2, XPNPEP3, NUCKS1, FAM160B2, DCLRE1B, MRPS11, C2orf47, SPAG16, PALB2, TBC1D17, ZNF668, METTL8, C10orf88, MYO19, RUFY1, INTS5, KRTAP1-1, TM2D1, ACTR8, SLC38A10, PRRC1, MYL6B, YTHDF3
Myocytes_FANTOM_1___364	Aran_et_al_2017	ALPL, CASQ2, CDH15, DES, HAS1, MYBPH, MYF5, MYH7, MYL1, MYL4, MYOG, RAPSN, SGCA, TNNI1, TNNT2, TTN, MYLPF
Myocytes_FANTOM_2___365	Aran_et_al_2017	ACTA1, ACTN2, ALPL, CDH15, CKM, MSTN, HAS1, HRC, MUSK, MYBPH, MYF5, MYH1, MYH2, MYH7, MYH8, MYL1, MYL4, MYOD1, MYOG, RAPSN, ROS1, SGCA, SGCG, SLN, TNNC2, TNNI1, TNNT2, ATP1B4, HEYL, ITGB1BP2, MYLPF, GSG1
Myocytes_FANTOM_3___366	Aran_et_al_2017	CDH15, HAS1, MYF5, MYH7, MYL1, MYL4, MYOG, RAPSN, TNNI1, TNNT2
naive_Bcells_BLUEPRINT_1___367	Aran_et_al_2017	BLK, CXCR5, CD19, MS4A1, CD72, SPIB, TCL1A, FCRL2
naive_Bcells_BLUEPRINT_2___368	Aran_et_al_2017	BLK, CXCR5, CD19, MS4A1, CD22, CD72, CD79B, CCR6, GPR18, CD180, MGAT5, SPIB, TCL1A, MBD4, TSPAN13, UTP6, FCRL2, TREML2
naive_Bcells_BLUEPRINT_3___369	Aran_et_al_2017	BLK, CXCR5, CD19, MS4A1, CD72, CD180, SPIB, TCL1A, FCRL2
naive_Bcells_HPCA_1___370	Aran_et_al_2017	BLK, CD19, MS4A1, CD22, CD37, CD72, CD79A, CSNK1G3, DSP, FCER2, GMFB, PNOC, SNX2, GCM1, AP3B1, MBD4, STAG3, PRDM4, PWP1, RRAS2, GGA2, SIPA1L3, STAP1, P2RY10, DEF8, MFN1, FCRL2, EGOT
naive_Bcells_HPCA_2___371	Aran_et_al_2017	CD1A, CD19, MS4A1, CD22, CD37, CD72, CD79A, CSNK1G3, DSP, FCER2, GMFB, MGAT5, PNOC, SNX2, AP3B1, MBD4, STAG3, PRDM4, PWP1, RRAS2, GGA2,

		SIPA1L3, STAP1, P2RY10, VPREB3, DEF8, MFN1, FCRL2, EGOT
naive_Bcells_HPCA_3___372	Aran_et_al_2017	BLK, CAPN3, CD1A, CD19, MS4A1, CD22, CD37, CD72, CD79A, CD79B, CSNK1G3, DSP, FCER2, GMFB, HSPA4, MGAT5, PNOC, SNX2, GCM1, AP3B1, MBD4, STAG3, PRDM4, PWP1, SP140, RRAS2, GGA2, SIPA1L3, STAP1, P2RY10, VPREB3, DEF8, MFN1, FCRL2, C10orf76, SMC6, MCM9, EGOT
naive_Bcells_NOVERSHTERN_1___373	Aran_et_al_2017	CXCR5, BMP3, CACNA1F, CAPN3, CD19, MS4A1, CD22, CD72, COL19A1, CSNK1G3, DAZL, DSP, FCER2, GNG3, GPR18, MATN1, MAP3K9, MMP17, MYBPC2, PAX5, PHKG1, PYGM, ZNF154, PRDM2, USP7, TCL1A, SYN3, ADAM20, USP6, AKAP6, TCL1B, BCL2L10, FRS2, PRDM4, RRAS2, GGA2, SIPA1L3, TCL6, TSPAN13, P2RY10, MYO3A, SDK2, WDR74, UBE2O, RBM15, SMC6, KHDRBS2
naive_Bcells_NOVERSHTERN_2___374	Aran_et_al_2017	CXCR5, BMP3, CACNA1F, CAPN3, CD19, MS4A1, CD22, CD72, COL19A1, CSNK1G3, DAZL, FCER2, GNG3, MAP3K9, MMP17, MYBPC2, PAX5, PHKG1, PRDM2, USP7, TCL1A, SYN3, ADAM20, USP6, AKAP6, TCL1B, FRS2, PRDM4, RRAS2, GGA2, SIPA1L3, TCL6, TSPAN13, P2RY10, WDR74, UBE2O, SMC6, KHDRBS2
naive_Bcells_NOVERSHTERN_3___375	Aran_et_al_2017	RERE, CXCR5, BMP3, CACNA1F, CAPN3, CD1A, CD19, MS4A1, CD22, CD72, COL19A1, CSNK1G3, DAZL, DSP, FCER2, GH1, GNG3, HLA-DOA, LY9, MATN1, CIITA, MAP3K9, MMP17, MYBPC2, PAX5, PGAM2, PHKG1, POU2F1, PRKCB, PYGM, RB1, TRA2B, ZNF154, SLC30A4, PRDM2, USP7, CUBN, TCL1A, SYN3, CDK13, PTCH2, ADAM20, USP6, AKAP6, TCL1B, TBC1D5, BCL2L11, STAG3, FRS2, PRDM4, RRAS2, GGA2, SIPA1L3, N4BP3, TCL6, TSPAN13, P2RY10, SNTG2, SDK2, WDR74, UBE2O, NOC3L, RBM15, FCRL2, SMC6, TRAPPC9, PIKFYVE, KHDRBS2, 43898
Neurons_ENCODE_1___376	Aran_et_al_2017	EPHA3, GNG3, INSM1, KCNQ2, NEUROD2, PCDH8, ACTL6B, CAMKV, STMN4
Neurons_ENCODE_2___377	Aran_et_al_2017	ABCA3, ACVR2B, ADRA2A, CACNA1B, CACNB3, CHRNB2, CPE, CRABP1, CRMP1, NCAN, CTNNA1, EFNB3, CELSR3, ELAVL3, EPHA3, FOXG1, GAD2, GAP43, GEM, GNG3, GRIK3, GRM2, ID1, INSM1, IREB2, KCNN1, KCNQ2, KCNQ3, KLC1, MEIS2, MLF1, MLLT3, MN1, CD200, MVD, NEUROD2, NNAT, NPY, NPTX1, NTRK3, PCDH8, PLXNA2, POU3F1, POU3F2, POU3F3, PKIA, MAPK8, PTPRZ1, PTX3, RPE65, SCN3A, CXCL12, SFRP4, SH3GL2, SH3GL3, SOX4, SOX11, SPAST, ZNF354A, THRA, ZFP37, ZNF711, ZNF14, ZNF43, ZNF195, ZNF223, TUBA1A, ST8SIA4, PDHX, ST8SIA2, HIST1H3D, DCHS1, STX16, B3GALT2, CDK5R1, BSN, HAP1, CYTH2, LHX2, IPO13, NUP93, TSPAN2, CFDP1, SEMA6C, ZNF211, IFI44, DPYSL4, SCGN, PNMA2, ZBTB6, MLLT11, TMSB15A, STMN2, C14orf1, ZFP30, ZNF510, WDR47, KIF21B, ARC, KIAA1107, AGTPBP1, CUX2, SEZ6L, SULT4A1, SETBP1, LRRTM2, RANBP6, CACNG5, CACNG4, PDLIM3, PCSK1N, KCNMB4, SCG3, HUNK, PODXL2, PARD6A, ZNF117, ACTL6B, GPR173, FAM105A, LRRN3, FNBP1L, ENOX1, AGPAT5, AP1AR, ZNF821, ANKRD10, ZNF415, BEX1, PCDHB11, ZNF253, C21orf62, KCNK12, ZNF529, PTBP2, ZBED5, NEUROG2, NEUROD6, REEP1, RASL11B, GDAP1L1, CAMKV, NKAIN1, RNF219, ZMAT4, VASH2, ZNF669, PGAP1, ZNF606, ZNF614, ZNF430, ZSCAN16, ZNF34, STMN4, ZNF484, YIPF4, MUM1, C16orf45, ZNF682, C1orf216, EMID1, PAQR3, ZNF675, TET3, KCTD13, ZNF493
Neurons_ENCODE_3___378	Aran_et_al_2017	ABCA3, ADRA2A, CACNA1B, CHRNB2, CPE, CRABP1, CRMP1, NCAN, CTNNA1, EFNB3, CELSR3, ELAVL3, EPHA3, FOXG1, GAD2, GAP43, GEM, GNG3, GRIK3, GRM2, INSM1, KCNQ2, KCNQ3, KLC1, MLLT3, CD200, NEUROD2, NNAT, NPY, NPTX1, PCDH8, PLXNA2, POU3F1, POU3F3, PKIA, MAPK8, PTPRZ1, SH3GL2, SH3GL3, SOX11, ZFP37, ZNF14, TUBA1A, ST8SIA4, PDHX, B3GALT2, BSN, HAP1, CYTH2, TSPAN2, SCGN, ZBTB6, MLLT11, TMSB15A, STMN2, WDR47, KIF21B, ARC, KIAA1107, CUX2, SEZ6L, SULT4A1, PCSK1N, SCG3, HUNK, PODXL2, ACTL6B, FAM105A, ENOX1, ZNF821, ZNF415, BEX1, KCNK12, PTBP2, ZBED5, NEUROG2, NEUROD6, REEP1, RASL11B, GDAP1L1, CAMKV, NKAIN1, RNF219, ZMAT4, PGAP1, ZNF614, ZNF34, STMN4, YIPF4, EMID1, ZNF675
Neurons_FANTOM_1___379	Aran_et_al_2017	ALDOC, ATP1A3, ATP1B1, ATP2B2, ATP6V1G2, CA8, CACNB4, CALB1, CAMK2B, CDH18, COX7A1, COX7B, DGKB, DEFB1, DLG2, DPP6, FABP6, FGF9, FGF12, GABBR1, GABRA1, GABRB2, GABRG2, GAD1, GNAO1, GNG3, GPM6A, GRIA2, GRID2, GRIK1, GRM1, GRM7, ID2, ITPR1, KCNC1, KIF5C, LPL, MT3, NDUFA5, NEFM, NEFH, NELL1, NEFL, NPPC, NPTX1, OMG, PCDH9, PCP4, SERPINI1, PRKCG, PTPRR, PVALB, RGS16, RORA, RTN1, SH3GL2, SLC6A1, SNAP25, SNCG, SYP, TAC1, SEC62, TSPAN7, TRPC3, ZNF208, AP3B2, SPARCL1, CACNA1G, INA, NRXN1, ELMO1, SNAP91, C1orf61, STMN2, CNKSR2, FAIM2, ARHGAP26, FSTL4, KIAA1107, ACSL6, SEZ6L, SMPX, SLC24A2, SCG3, DDX25, SPOCK3, ST8SIA3, GPRC5B, GNG13, TM6SF1, FXYD7, IL20RA, LRRN3, SUSD4, BEX1, PRMT8, DNAJC12, ANKS1B, PLXDC1, SLC12A5, KLHL1, REEP1, GPR63, STMN4, TRIM9, TCEAL2, SHISA6

Neurons_FANTOM_2___380	Aran_et_al_2017	ACYP2, AGTR2, ALDOC, ATP1A3, ATP1B1, ATP1B2, ATP2A3, ATP2B2, ATP6V1G2, CA7, CA8, CACNA1A, CACNB2, CACNB4, CALB1, CAMK2B, CDH18, CHGB, COX7A1, COX7B, DAB1, DACH1, DGKB, DGKG, DEFB1, DLG2, DYNC1I1, DPP6, FABP3, FABP6, FGF9, FGF12, FGF14, GABBR1, GABRA1, GABRB2, GABRB3, GABRG2, GAD1, GAD2, GNAO1, GNG3, GNG4, GPM6A, GRIA2, GRIA3, GRID2, GRIK1, GRM1, GRM7, HSBP1, HTR5A, ID2, ITPR1, KCNA2, KCNC1, KCNK1, KIF5A, KIF5C, KPNA5, LPL, MT3, NAP1L2, NCAM1, NDUFA3, NDUFA4, NDUFA5, NEFM, NEFH, NELL1, NEFL, NELL2, NPPC, NPTX1, NRCAM, OMG, PCDH9, PCP4, PDE9A, PEG3, SERPINI1, PPP3CA, PRKCG, MAPK10, PTPRR, PVALB, RAB3A, RGS7, RGS16, RORA, RTN1, RYR2, SCN1A, SCN2A, SH3GL2, SLC1A6, SLC6A1, SLC8A1, SNAP25, SNCG, ABCC8, VAMP2, SYP, TAC1, SEC62, TSPAN7, TRPC3, ZNF208, KCNAB1, AP3B2, PIP5K1B, SPARCL1, PPFIA2, SYNJ1, CACNA1G, INA, CACNA2D2, CPNE6, RAB33A, NRXN3, NRXN1, AKAP7, GABBR2, GPRASP1, JAKMIP2, ELMO1, SNAP91, SAP18, CORO2B, C1orf61, ATP5L, STMN2, KIF3A, HHLA3, MGAT4A, GABARAPL2, CNKSR2, KIFAP3, SV2C, FAIM2, MYT1L, ARHGAP26, FSTL4, KIAA1107, ACSL6, SEZ6L, SMPX, SLC24A2, SULT4A1, DNM3, GALNT8, CLUL1, PCDH17, GOLIM4, KCNMB4, PCLO, NDUFAF4, SCG3, DDX25, NME7, SPOCK3, ST8SIA3, PIGP, GPRC5B, GNG13, BCL11A, TM6SF1, FXYD7, IL20RA, FAM134B, LRRN3, LRRC49, SUSD4, CISD1, BEX1, PRMT8, DNAJC12, ANKS1B, PLXDC1, TTYH1, SLC12A5, KLHL1, NDRG4, REEP1, FAM184A, ZNF385D, MAP9, CEP76, GPR63, STMN4, HOPX, TRIM9, IQCK, RALYL, TCEAL2, THSD7A, LPCAT4, FAM21A, SHISA6,
Neurons_FANTOM_3___381	Aran_et_al_2017	ACYP2, ALDOC, ANK2, ATP1A3, ATP1B1, ATP2A3, ATP2B2, ATP6V1G2, CA8, CACNA1A, CACNB2, CACNB4, CALB1, CAMK2B, CDH18, CHGB, CHN1, COX7A1, COX7B, CRMP1, DGKB, DGKG, DEFB1, DLG2, DYNC1I1, DPP6, FABP3, FABP6, FABP7, FGF9, FGF12, FGF14, GABBR1, GABRA1, GABRB2, GABRG2, GAD1, GNAO1, GNG3, GPM6A, GPM6B, GRIA2, GRID2, GRIK1, GRM1, GRM7, ID2, ITPR1, KCNC1, KIF5C, LHX1, LPL, MT3, NAP1L2, NCAM1, NDUFA3, NDUFA5, NEFM, NEFH, NELL1, NEFL, NELL2, NOVA1, NPPC, NPTX1, OMG, PCDH9, PCP4, PDE9A, PEG3, SERPINI1, PRKCG, PTPRN, PTPRR, PVALB, RAB3A, RGS16, RORA, RTN1, SCN1A, SH3GL2, SLC1A6, SLC6A1, SLC8A1, SNAP25, SNCG, SORL1, SYP, TAC1, SEC62, TSPAN7, TRPC3, ZNF208, KCNAB1, AP3B2, NME5, SPARCL1, CACNA1G, INA, RAB33A, NRXN3, NRXN1, GABBR2, SPOCK2, JAKMIP2, ELMO1, SNAP91, RCAN2, C1orf61, STMN2, KLK8, MGAT4A, GABARAPL2, CNKSR2, FAIM2, ARHGAP26, FSTL4, KIAA1107, ACSL6, SEZ6L, SMPX, SLC24A2, SOSTDC1, DNM3, PCDH17, NDUFAF4, SCG3, DDX25, SNX10, NME7, SPOCK3, ST8SIA3, PIGP, GPRC5B, GNG13, BCL11A, TM6SF1, FXYD7, IL20RA, FAM134B, LRRN3, LRRC49, SUSD4, BEX1, PRMT8, DNAJC12, ANKS1B, PLXDC1, SLC12A5, KLHL1, NDRG4, REEP1, ZNF385D, CEP76, GPR63, STMN4, HOPX, TRIM9, TCEAL2, SHISA6
Neutrophils_BLUEPRINT_1___382	Aran_et_al_2017	CA4, CEACAM3, FCGR3B, CXCR1, CXCR2, PGLYRP1, MMP25, ZDHHC18, TRPM6
Neutrophils_BLUEPRINT_2___383	Aran_et_al_2017	CA4, CEACAM3, FCGR3B, CXCR1, PGLYRP1, VNN3, MMP25, ZDHHC18
Neutrophils_BLUEPRINT_3___384	Aran_et_al_2017	CA4, CEACAM3, CXCR1, CXCR2, PGLYRP1, P2RY13, MMP25, ZDHHC18
Neutrophils_FANTOM_1___385	Aran_et_al_2017	APAF1, CBL, CEACAM3, CSF2RB, DDX3X, FCAR, FCGR3B, FPR2, CXCR2, NFYA, PAK2, PTEN, SLC19A1, UBE2B, BEST1, ELL, MTMR3, HERC3, PGLYRP1, SLC25A44, DHX34, TOX4, TECPR2, WWP2, LMTK2, WDFY3, MED13L, ACAP2, CLEC4E, UBN1, NRBF2, TREM1, HRH4, RMND5A, TMUB2, TMEM185B, GCC1, TREML2, BTNL8, NDEL1, ZDHHC18, UBXN2B
Neutrophils_FANTOM_2___386	Aran_et_al_2017	APAF1, CA4, CASP5, CBL, CEACAM3, CEACAM8, CSF2RB, DDX3X, FCAR, FCGR3B, FPR2, CXCR2, MAK, NFYA, PTEN, SDF2, SLC19A1, TGM3, TOP1, UBE2B, UBE2D1, BEST1, TRIM25, ELL, KSR1, MTMR3, HERC3, PGLYRP1, SPAG9, NMI, CIR1, TTLL4, SLC25A44, DHX34, IP6K1, TOX4, TECPR2, USP15, CAMKK2, LILRA1, WWP2, BTN2A1, LMTK2, WDFY3, MED13L, ACAP2, PADI4, CLEC4E, UBN1, NRBF2, TREM1, HRH4, TMUB2, GCC1, TREML2, BTNL8, FBXO38, NDEL1, ZDHHC18, UBXN2B
Neutrophils_FANTOM_3___387	Aran_et_al_2017	APAF1, CBL, CEACAM3, CSF2RB, DDX3X, FCGR3B, FPR2, CXCR2, NFYA, PTEN, SLC19A1, UBE2B, BEST1, ELL, MTMR3, HERC3, PGLYRP1, DHX34, TECPR2, WWP2, LMTK2, WDFY3, MED13L, ACAP2, CLEC4E, UBN1, NRBF2, TREM1, HRH4, TMUB2, GCC1, TREML2, BTNL8, NDEL1, ZDHHC18, UBXN2B
Neutrophils_HPCA_1___388	Aran_et_al_2017	CLC, CSF3R, FCGR3B, FPR2, HBB, CXCR2, S100A12, P2RY13, TREM1
Neutrophils_HPCA_2___389	Aran_et_al_2017	CLC, CSF3R, FCGR3B, FPR2, HSPA6, CXCR2, S100A12, P2RY13, TREM1

Neutrophils_HPCA_3___390	Aran_et_al_2017	CLC, CSF3R, FCGR3B, FPR2, HBB, HSPA6, CXCR2, S100A12, LILRB2, LILRA2, P2RY13, TREM1
Neutrophils_IRIS_1___391	Aran_et_al_2017	CEACAM3, FCGR3B, CXCR1, CXCR2, MEFV, VNN3, MMP25, BTNL8
Neutrophils_IRIS_2___392	Aran_et_al_2017	BMX, CA4, CEACAM3, FCGR3B, FPR2, CXCR1, CXCR2, MEFV, AATK, P2RY13, TREM1, VNN3, MMP25, BTNL8, TRPM6
Neutrophils_IRIS_3___393	Aran_et_al_2017	BMX, CA4, CEACAM3, FCGR3B, FPR2, GPR27, CXCR1, CXCR2, MEFV, IL18RAP, AATK, P2RY13, TREM1, VNN3, MMP25, BTNL8, TRPM6
NK_cells_BLUEPRINT_1___394	Aran_et_al_2017	FASLG, KLRD1, PTGDR, PTPN4, XCL1, TKTL1, IL18RAP, NCR1, ZMYND11, SACM1L, CD244, AGK, DNAJB14
NK_cells_BLUEPRINT_2___395	Aran_et_al_2017	FASLG, CX3CR1, GZMB, GZMM, IL2RB, KLRD1, KPNB1, MED1, PRF1, MAPK1, PTGDR, PTPN4, BRD2, XCL1, MAP3K7, TKTL1, TNFSF11, IL18RAP, ZNF264, NCR1, STX8, PJA2, HELZ, GNLY, STAG2, ZMYND11, ZBTB1, SACM1L, TBX21, RAB14, CD244, AGK, DNAJB14, FIP1L1, ARPC5L, HIPK1
NK_cells_BLUEPRINT_3___396	Aran_et_al_2017	FASLG, CD247, CTSW, CX3CR1, GZMB, IL2RB, KLRD1, LTA, PRF1, PTGDR, PTPN4, XCL1, TKTL1, TNFSF11, IL18RAP, NCR1, GNLY, ZMYND11, SACM1L, TBX21, CD244, AGK, ZNF426, DNAJB14, HIPK1
NK_cells_FANTOM_1___397	Aran_et_al_2017	FASLG, CD247, CX3CR1, GRIK4, GZMB, IL2RB, LIM2, PRF1, PTGDR, TKTL1, NCR1, NMUR1, GNLY, CD160, TBX21
NK_cells_FANTOM_2___398	Aran_et_al_2017	IL2RB, PTGDR, XCL1, IL18RAP, NCR1, SACM1L, DNAJB14, ARPC5L
NK_cells_FANTOM_3___399	Aran_et_al_2017	GZMB, IL2RB, LIM2, PTGDR, NCR1, NMUR1, GNLY, CD160
NK_cells_HPCA_1___400	Aran_et_al_2017	BAD, CD247, 44081, CHRNE, DR1, GIPR, GOLGA4, GZMH, GZMB, GZMM, HNRNPL, IFNG, LAG3, MGAT2, MLH1, NEK1, NFE2L2, NKG7, PPP2CA, PRF1, PRKAG1, PTGDR, XCL1, SON, SUPV3L1, TBCC, PRDM2, HIST1H3A, NCR1, PRDX6, YAF2, KLRG1, SF3B4, NMUR1, GNA13, GPATCH8, DNAJC2, ASTE1, ANKRD11, TBX21, AMZ2, THAP1, UBE2Q1, CDKN2AIP, RBM25, DNAJB14, OSBPL7, TSTD2, HIPK1, NCR3, C1orf174
NK_cells_HPCA_2___401	Aran_et_al_2017	BAD, CHRNE, CTSW, DR1, GIPR, GOLGA4, GTF3C1, GZMH, IFNG, KLRD1, LAG3, MGAT2, NKG7, PPP2CA, PTGDR, XCL1, FBXW4, SUPV3L1, TSPYL1, HIST1H3A, RGS9, IL18RAP, NCR1, RBM39, NMUR1, GNLY, ZCCHC11, LEMD3, ASTE1, TBX21, IL21R, AMZ2, WBP11, RSRC2, ALG13, COQ10B, HIPK1
NK_cells_HPCA_3___402	Aran_et_al_2017	BAD, CD247, 44081, CHRNE, DR1, GIPR, HNRNPL, IFNG, IL2RB, MLH1, NEK1, NKG7, PRF1, PRKAG1, PTGDR, SBF1, CCL4, XCL1, TBCC, HIST1H3A, NCR1, GGPS1, PRDX6, ZBTB39, YAF2, SF3B4, NMUR1, WDR45, GPATCH8, ASTE1, TBX21, IL21R, AMZ2, THAP1, UBE2Q1, CDKN2AIP, OSBPL7, TSTD2, NCR3
NK_cells_IRIS_1___403	Aran_et_al_2017	FASLG, KLRD1, PTGDR, PTPN4, XCL1, TKTL1, IL18RAP, NCR1, ZMYND11, SACM1L, CD244, AGK, DNAJB14
NK_cells_IRIS_2___404	Aran_et_al_2017	FASLG, CTSW, CX3CR1, GZMM, KLRD1, MED1, PTGDR, PTPN4, XCL1, TKTL1, TNFSF11, IL18RAP, ZNF264, NCR1, ZMYND11, SACM1L, CD244, AGK, DNAJB14
NK_cells_IRIS_3___405	Aran_et_al_2017	KLRD1, PTGDR, PTPN4, XCL1, TKTL1, IL18RAP, NCR1, DNAJB14
NK_cells_NOVERSHTERN_1___406	Aran_et_al_2017	CHRNE, GIPR, IFNG, HIST1H3A, NCR1, NMUR1, ASTE1, TBX21
NK_cells_NOVERSHTERN_2___407	Aran_et_al_2017	CHRNE, GIPR, IFNG, PRKAG1, HIST1H3A, NCR1, NMUR1, ASTE1, AMZ2
NK_cells_NOVERSHTERN_3___408	Aran_et_al_2017	GZMH, GZMB, KLRD1, PRF1, NCR1, NMUR1, GNLY, CD160, TBX21
NKT_NOVERSHTERN_1___409	Aran_et_al_2017	CASP5, PHKG1, RARA, S100B, BEST1, DOLK, TP53TG5, GMIP, GSG1
NKT_NOVERSHTERN_2___410	Aran_et_al_2017	AMBN, CASP5, PHKG1, RARA, S100B, SGCA, TCOF1, BEST1, IL17B, TP53TG5, GMIP, L1TD1
NKT_NOVERSHTERN_3___411	Aran_et_al_2017	PHKG1, RARA, BEST1, ARPC1A, TP53TG5, L1TD1, KLHL26, GEMIN7, GSG1, PMFBP1
Osteoblast_FANTOM_1___412	Aran_et_al_2017	BMPR1A, COMP, DCTD, DYNC1LI2, EPYC, GRSF1, IBSP, MFAP3, PRELP, SGCG, UBE2L3, COL14A1, ITGA8, EIF2S2, KIF3B, LAPTM4A, APPBP2, MAB21L2, ILVBL, XPOT, DOLK, ICMT, TMEM50A, CBY1, RPS6KC1, COPS7A, MYOZ2, NUDT9, RNF121, LIN7C, DHX32, IL26, STARD7, C5orf15, PAPPA2, EBF2, ATG9A, MUL1, TM2D3, UNC119B, LRRC15
Osteoblast_FANTOM_2___413	Aran_et_al_2017	ARCN1, BMPR1A, DYNC1LI2, EPYC, GRSF1, HTR2A, IBSP, MFAP3, PSMB5, SGCG, UBE2L3, COL14A1, ITGA8, KIF3B, EIF4E2, FRMPD4, APPBP2, MAB21L2, YME1L1,

		ILVBL, XPOT, ICMT, CBY1, RPS6KC1, MYOZ2, RNF121, LIN7C, NPLOC4, IL26, STARD7, PAPPA2, EBF2, FTO, TM2D3, UNC119B, FAM168B, LRRC15, YIPF6
Osteoblast_FANTOM_3___414	Aran_et_al_2017	ARCN1, BMPR1A, COMP, DCTD, DYNC1LI2, EPYC, GRSF1, HLCS, IBSP, MFAP3, PRELP, PSMB5, SGCD, SGCG, UBE2L3, COL14A1, ITGA8, EIF2S2, KIF3B, LAPTM4A, FRMPD4, YAP1, APPBP2, MAB21L2, YME1L1, ILVBL, XPOT, DOLK, TRIM32, ZNF629, ICMT, TMEM50A, MKRN2, CBY1, TRAF3IP1, RPS6KC1, COPS7A, AMOTL2, MYOZ2, NUDT9, RNF121, LIN7C, NPLOC4, DHX32, IL26, KCMF1, STARD7, C5orf15, CCDC47, PAPPA2, EBF2, ATG9A, FTO, TMEM185B, MUL1, TM2D3, UNC119B, FAM168B, LRRC15, DPY19L4
Osteoblast_HPCA_1___415	Aran_et_al_2017	ACTG1, RHOA, CCKAR, EXTL1, FOXC2, GGCX, GLUD1, HIST1H1A, NFATC4, PGK1, SLC9A5, TUB, ZNF16, GDF5, SSNA1, EIF3G, FIBP, LONP1, ABCF2, RNF41, ARPC1A, MAB21L2, TXNL4A, CBY1, TRPC4AP, CHMP2A, ARL6IP4, TMED9, GLT8D1, PCDHGA11, IFT46, CUEDC2, FIP1L1, SFXN3, TMEM222
Osteoblast_HPCA_2___416	Aran_et_al_2017	RHOA, EXTL1, GGCX, NFATC4, ZNF16, GDF5, FIBP, LONP1, ABCF2, MAB21L2, TXNL4A, CHMP2A, ARL6IP4, PCDHGA11, CUEDC2, TMEM222
Osteoblast_HPCA_3___417	Aran_et_al_2017	ACTG1, RHOA, CCKAR, AP2S1, ATF6B, DPAGT1, DRG2, EXTL1, FOXC2, GGCX, GLUD1, HIST1H1A, HARS, IARS, EIF6, KIF22, NFATC4, PGK1, SLC9A5, SSR1, TUB, ZFPL1, ZNF16, GDF5, AKR7A2, SSNA1, EIF3G, FIBP, COX7A2L, LONP1, SNX17, ABCF2, SNUPN, RNF41, ARPC1A, MAB21L2, TXNL4A, PARK7, EXOC7, ABCB9, MTCH1, CBY1, LMOD1, TRPC4AP, HSPB7, CHMP2A, EPN1, ARL6IP4, TMED9, GLT8D1, PCDHGA11, IFT46, PRDM11, C12orf43, CUEDC2, ZNF768, CPSF7, FIP1L1, SFXN3, BRIP1, TMEM222
pDC_FANTOM_1___418	Aran_et_al_2017	APOC3, CSHL1, DNASE1L3, GZMB, HIST1H2BB, HPD, KCNA5, SCT, P2RY14, LILRB4, CUX2, LRRC36
pDC_FANTOM_2___419	Aran_et_al_2017	CSHL1, DNASE1L3, GZMB, HIST1H2BB, KCNA5, RPL3L, SCT, P2RY14, LILRB4, CUX2, LRRC36
pDC_FANTOM_3___420	Aran_et_al_2017	CACNB1, DNASE1L3, FKBP2, GZMB, IL3RA, KCNA5, MYBPC1, SCT, SLC12A3, SPIB, ZNF221, MAPKAPK2, P2RY14, CXCL13, LILRB4, SLITRK3, CUX2, TSPAN13, SPCS1, TLR7, KCNK10, KCTD5, LRRC36, CELA2A, CCR2
pDC_NOVERSHTERN_1___421	Aran_et_al_2017	FLT3, FUT7, GZMB, IDH3A, SCT, SPIB, CD2AP, TLR7, PTCRA
pDC_NOVERSHTERN_2___422	Aran_et_al_2017	FLT3, FUT7, GZMB, IDH3A, SCT, SPIB, CD2AP, TLR7, KCNK10, PTCRA
pDC_NOVERSHTERN_3___423	Aran_et_al_2017	RUNX2, FLT3, FUT7, CXCR3, GZMB, IDH3A, SCT, SPIB, TACR1, CD2AP, TLR7, KCNK10, PTCRA
Pericytes_ENCODE_1___424	Aran_et_al_2017	ADCY3, CDK4, GGCX, HIC1, MLLT1, MYBPC2, P4HB, MAPK3, PTGER1, SLC6A13, TAF15, DYNLL1, ASH2L, ZBTB22, S1PR2, BCKDK, COPS6, TMED1, CBX6, TXN2, TMEM184B, ERGIC3, MIER2, ZNF444, PRDM11
Pericytes_ENCODE_2___425	Aran_et_al_2017	ADCY3, ARF5, ARNT, ATP5G1, ATP5J, CALR, CDK4, CTBP1, DMWD, ELK1, GGCX, HIC1, ISLR, KIF22, MLLT1, MMP11, MYBPC2, MYBPH, NDUFB1, NDUFB7, NNAT, P4HB, PFN1, PPP2CB, PPP2R5D, MAPK3, PSMD8, PTGER1, TRIM27, SLC6A13, TNXB, SLC35A2, ZFPL1, TAF15, LZTR1, AKR7A2, DYNLL1, AP3D1, ASH2L, KCNAB3, ZBTB22, S1PR2, MED16, CTDSP2, ARFRP1, RNF41, BCKDK, TAB1, TIMM44, TM9SF1, TRIM3, GIPC1, YIF1A, KDELR1, LMAN2, COPS6, TMED1, RAB35, TMEM115, LZTS1, WDR6, COPZ1, MLXIP, RALY, CIC, USP22, CBX6, LMOD1, TXN2, TMEM184B, ZNF500, IRF2BP1, ERAL1, VPS4A, AHDC1, C19orf53, MYLPF, GMPPA, ERGIC3, GAR1, MIER2, TMEM161A, ATP5SL, ZNF444, PCDHGA9, PRDM11, PKNOX2, C11orf95, C7orf26, TSEN34, TMEM185B, ZNF768, HPS6, ADAMTS12, IL17RC, TOM1L2, TXLNA
Pericytes_ENCODE_3___426	Aran_et_al_2017	ATP5J, BCS1L, CALR, DPAGT1, ELK1, GNAS, HIC1, DNAJC4, MMP11, MYBPC2, NDUFA8, NDUFB1, NDUFB7, NDUFS5, P4HB, PPIB, PPP2R5D, PSKH1, PSMD8, PTGER1, RAB5B, DPF2, TRIM27, RPN1, RXRB, SH3GL1, SLC6A4, SLC6A13, SSR2, SLC35A2, ZFPL1, USP5, LZTR1, CDK10, KHSRP, AKR7A2, DYNLL1, EDF1, AP3D1, ASH2L, KCNAB3, ZBTB22, SEC24C, SNX17, SEC16A, MED16, CTDSP2, ARFRP1, RNF41, TAB1, TM9SF1, 44083, EHMT2, KDELR1, LMAN2, TMEM115, WDR6, COPZ1, RALY, TCF25, GANAB, SMG5, SIRT3, CBX6, PES1, POFUT1, DNPEP, CBY1, TMEM184B, ZNF500, IRF2BP1, ERAL1, CHMP2A, SGSM3, REM1, C19orf53, MYLPF, GMPPA, ZNF771, CUTA, ERGIC3, GPR173, DDX49, TMEM161A, ATP5SL, PACS1, PCDHGB5, PRDM11, PKNOX2, TSEN34, HPS6, OPA3, UBXN6, TOM1L2
Pericytes_FANTOM_1___427	Aran_et_al_2017	FLT1, GDNF, HTR2B, P2RX1, POU2F2, MASP1, KSR1, S1PR2, HAND2, ADAMTSL2, COLEC10, BTN2A1, KIF26B, ADAMTS12

Pericytes_FANTOM_2___428	Aran_et_al_2017	COL10A1, SLC31A1, FLT1, GDNF, HTR2B, MASP1, HAND2, ADAMTSL2, DSCR4, COLEC10, IGF2BP3, ABTB2, ADAMTS12
Pericytes_FANTOM_3___429	Aran_et_al_2017	FLT1, GDNF, HTR2B, MASP1, COLEC10, BTN2A1, KIF26B, ADAMTS12
Plasma_cells_BLUEPRINT_1___430	Aran_et_al_2017	AMPD1, TNFRSF17, FKBP2, PNOC, SSR4, RNGTT, SPATS2, UBA5, ZBP1
Plasma_cells_BLUEPRINT_2___431	Aran_et_al_2017	ALPI, AMPD1, AVP, TNFRSF17, BMP8B, C21orf2, CAMP, CASP10, CCNC, CD27, ENTPD1, CD79A, CDH15, COX6A2, CRYBB1, CRYBB3, CRYGC, CYP11A1, EPO, FKBP2, GDF2, GOLGA3, GOLGA4, GRM4, GUCA1A, HIST1H2BB, HSF4, HSPA6, IDE, IRF4, KCNN3, MARS, MGAT2, NEUROG1, NOS2, NPAS1, NPPA, NPPC, PNOC, PRM1, PTPRS, RAB3A, RAD17, RGS1, RGS13, RPN2, CCL25, SLC5A2, SRP54, SSR4, SS18, SYT5, TG, SEC62, TNNT3, HSP90B1, UBE2G1, ZNF133, RNF113A, RNF103, MANF, ITGA8, B4GALT3, MBTPS1, RNGTT, MATN4, EBAG9, HAND2, TBX4, CLINT1, KNTC1, KIAA0125, DMTF1, PREB, SLC35B1, MRPS31, CNKSR1, SEMA6C, PRDX4, LBX1, AVIL, SEC24A, TMED10, ZPBP, WDR45, TREH, FNDC3A, ATF6, MAST1, MAPK8IP3, UBXN4, SCFD1, CUX2, AIPL1, GORASP2, TBL2, DKKL1, NDOR1, SERP1, ARHGEF16, MCTS1, ALG5, GMPPA, SHANK1, CELA2B, YIPF1, LAX1, CCDC40, TMEM39A, UFSP2, CCDC88A, LTB4R2, PRX, SRPRB, PRDM14, MAGEF1, CHST8, PDIA2, SPATS2, MIS12, CCDC121, UBA5, ADM2, CNTD2, CCDC33, TRABD, ZBP1, STMN4, SLCO5A1, GRWD1, USP48, MTDH, IMP4, SIX5
Plasma_cells_BLUEPRINT_3___432	Aran_et_al_2017	AMPD1, APOA1, APOC3, ARL1, PHOX2A, ARSA, AUP1, TNFRSF17, BMP8B, CA7, CACNA1S, CASP10, CAV1, CCNC, CD19, CD79A, CD79B, CHRNA4, CHRNG, CLCNKB, CRYBA4, CRYBB1, CSHL1, CYBA, CYP11A1, DAD1, DDOST, DNASE1L2, DPAGT1, DRD4, DRD5, FGF6, FKBP2, GH2, GNB3, GOLGB1, GP9, GRIN1, HDLBP, DNAJC4, IGF1, KCNN3, KRT10, CD180, MYL2, NTRK1, P2RY4, PDE6A, PNOC, POMC, POU3F3, PPIB, PTGER1, RAD17, RFX2, RGS13, RPN1, RPN2, SHBG, SLC6A13, SMPD2, SNAPC4, SSR1, SSR4, SURF1, SYT5, T, TERT, LEFTY2, SEC62, TNNT3, TP73, HSP90B1, TSHR, VPREB1, ZNF37A, ZNF133, ZNF142, MANF, ELL, BFSP2, UTF1, ITGA8, B4GALT3, MYH13, PABPC4, FBP2, SLC13A2, KCNQ4, GPR37L1, PICK1, TMEM59, CUL7, KIAA0125, PREB, PDIA6, SLC35B1, TIMM17B, CNKSR1, CNPY2, TM9SF1, PRDX4, LBX1, SEC24A, FTCD, SEC61B, LMAN2, TMED10, KDELR2, WDR45, SEC63, FNDC3A, RALY, MAST1, SIPA1L3, DDN, ABCB9, ISCU, SEC61G, TNFRSF13B, TSSK2, PPIL2, GORASP2, HEYL, TBL2, NDOR1, SERP1, ARHGEF16, SPCS1, SAP30BP, VPREB3, ALG5, GMPPA, SEC61A1, RAX, VSX1, SLC35C2, THAP4, IFT52, ERGIC3, YIPF1, TMED9, DEF8, RASIP1, ZDHHC4, C19orf73, UFSP2, GPRC5D, NGLY1, GLT8D1, PCDHA5, IRGC, LTB4R2, UGGT1, SRPRB, MYL7, MAGEF1, ACBD3, KLC2, SPATS2, YIPF2, FCRL2, OGFOD2, LMAN1L, ALG9, CSPP1, UBA5, DOK3, CNTD2, C6orf25, CEACAM21, MTDH, SLC38A10, SIX5, R3HCC1, MIA3, RNF208
Plasma_cells_HPCA_1___433	Aran_et_al_2017	TNFRSF17, CD79A, GABRR2, GPLD1, KCNN3, NPAS1, PNOC, RGS13, CCL25, SLC5A2, SSR4, TP73, TSHR, PREB, CNKSR1, KLF15, VPREB3, TMEM39A, FN3K, UBA5, ADM2, ZBP1
Plasma_cells_HPCA_2___434	Aran_et_al_2017	TNFRSF17, BMP8B, C21orf2, CD27, CD79A, CNR1, FKBP2, GABRR2, GPLD1, HSF4, KCNN3, NPAS1, PNOC, RGS13, CCL25, SLC5A2, SSR4, TCF3, TP73, HSP90B1, TSHR, WNT1, PREB, CNKSR1, SERP1, KLF15, VPREB3, HOOK2, SEC61A1, TMEM39A, FN3K, C16orf58, SPATS2, ACBD4, UBA5, ADM2, ZBP1, SIX5
Plasma_cells_HPCA_3___435	Aran_et_al_2017	TNFRSF17, CD79A, GABRR2, GPLD1, KCNN3, NPAS1, PNOC, RGS13, CCL25, SLC5A2, SSR4, TP73, CNKSR1, KLF15, VPREB3, ADM2, ZBP1
Plasma_cells_IRIS_1___436	Aran_et_al_2017	AMPD1, TNFRSF17, KCNN3, PNOC, RGS13, SSR4, RNGTT, SEC24A, ZBP1
Plasma_cells_IRIS_2___437	Aran_et_al_2017	AMPD1, TNFRSF17, CCNC, CD79A, FKBP2, GOLGA3, HSF4, KCNN3, MGAT2, PNOC, RGS13, SRP54, SSR4, HSP90B1, UBE2G1, RNF113A, MANF, RNGTT, EBAG9, PREB, MRPS31, SEC24A, FNDC3A, ATF6, MAST1, UBXN4, TBL2, SERP1, ALG5, GMPPA, YIPF1, LAX1, TMEM39A, LTB4R2, SPATS2, UBA5, ADM2, ZBP1, USP48, MTDH, IMP4
Plasma_cells_IRIS_3___438	Aran_et_al_2017	AMPD1, TNFRSF17, CCNC, CD79A, FKBP2, KCNN3, PNOC, RGS13, SSR4, TSHR, UBE2G1, RNGTT, PREB, SEC24A, TMEM39A, SPATS2, UBA5, ZBP1, MTDH
Platelets_HPCA_1___439	Aran_et_al_2017	ADCY8, ALOX12, APOA1, ARHGAP6, AVPR1A, BMP8B, BNIP2, CASQ1, CETP, COL10A1, GNAS, GNB3, GP1BA, GP5, GPR3, GPR17, LPAR4, HPCA, HSD17B3, IL5, ITGA2B, KIF2A, LCN1, LIMS1, SMAD2, MAN2A2, MAX, MEA1, RAB8A, CIITA, MPL, MUC6, NPPA, SIX6, PDE6A, PDE6H, PF4V1, PHKB, PITX3, PLXNB3, PPM1A, PPY, PRKCG, PTPRA, RNF2, RPA1, SELP, SLC6A4, NEK4, TACR2, TAL1, TG, TNNC2, CRISP2, VCL, VDAC3, XPNPEP1, ZNF37A, ZNF214, ST7, NCOA4, CDK2AP1, HIST1H2BO, SNX3, MYOM1, KSR1, ASAP2, ENDOU, RNF8, CHD1L, RGS6, SEC14L5, SNPH, TLK1, TECPR2, RNF10, TRIM10, HTATIP2, AKAP3, LEFTY1, IGF2BP3, CD160, PARK7,

		ACSBG1, ARHGEF12, TMEM50A, FBXO9, RNF11, DNAI1, MLH3, EIF2AK1, MORC1, LSM1, RABGEF1, RAX, KLK14, DERA, PHF20L1, RDH11, GP6, CLEC1B, TAOK3, BIN2, NCKIPSD, UIMC1, CMPK1, TUBA8, RNF186, L1TD1, BEST2, TXNL4B, CCDC40, ADI1, WDR11, ACTR10, NXF3, PCDHGB5, PCDHGA1, CABP5, ANO2, LRTM1, NEUROD4, PCTP, CHST8, ARMC7, SYNPO2L, PANK2, RUFY1, ULBP1, TUBB1, ASB8, PTCRA
Platelets_HPCA_2___440	Aran_et_al_2017	ADCY8, ALOX12, AMD1, ANXA7, ARHGAP6, AVPR1A, BMP8B, BNIP2, CASQ1, CETN2, COL10A1, DPYS, EGR4, F13A1, GNAS, GNB3, GP1BA, GP5, GP9, GPR3, GPR17, LPAR4, GRIK1, HPCA, HSD17B3, IL5, ITGA2B, KIF2A, LCN1, LIMS1, SMAD2, MAN2A2, MAX, MEA1, RAB8A, FOXO4, MPL, MTR, NOS2, NPPA, ODC1, SIX6, PDE6A, PDE6H, PF4V1, PHKB, PITX3, PPM1A, PPY, PRKCG, PTGDR, PTPRA, RHCE, RIT2, RNF2, RPA1, SELP, SLC6A4, SLC18A2, NEK4, TACR2, TAL1, TG, TNNC2, CRISP2, VCL, VDAC3, XPNPEP1, ZNF37A, ZNF214, ST7, NCOA4, CDK2AP1, HIST1H2BO, NCK2, TCAP, SNX3, MYOM1, PEX11B, KSR1, ASAP2, ENDOU, RNF8, ADIPOQ, CHD1L, RGS6, ZNF592, SEC14L5, SNPH, TLK1, TECPR2, NR2E3, TRIM10, HTATIP2, AKAP3, MYL12A, LEFTY1, IGF2BP3, PGRMC1, CD160, PDCD10, PARK7, SNW1, ACSBG1, ARHGEF12, SLC16A8, TMEM50A, FAM32A, IPCEF1, LSM14A, FBXO9, RNF11, DNAI1, NPTN, MLH3, EIF2AK1, MORC1, LSM1, RABGEF1, RAX, KLK14, DERA, RDH11, GP6, CLEC1B, CEND1, TAOK3, BIN2, NCKIPSD, UIMC1, CMPK1, TUBA8, RNF186, L1TD1, BEST2, TXNL4B, CCDC40, ADI1, C7orf43, WDR11, ACTR10, NXF3, PCDHGB5, PCDHGA1, CABP5, KCMF1, ANO2, LRTM1, PRX, PCTP, NPFFR1, NPVF, ADIPOR2, ARMC7, ZMYM1, SYNPO2L, PANK2, RUFY1, ULBP1, TUBB1, TRAPPC9, ASB8, PTCRA, USP12, 43898
Platelets_HPCA_3___441	Aran_et_al_2017	ALOX12, ARHGAP6, GP1BA, PDE6H, PF4V1, SELP, SEC14L5, ACSBG1, MLH3, CLEC1B, CABP5, TUBB1
Preadipocytes_ENCODE_1___442	Aran_et_al_2017	ARCN1, ARL1, CDK7, FGF7, GLG1, HAS1, HDLBP, SSR1, TESK1, TMEM11, BUB3, HAND2, BAG3, MORF4L2, TUBA1B, NCKAP1, KDELR2, SEC63, FAM98A, TCTN3, MMADHC, GLT8D1, SRPRB, ALG8, SLC25A32
Preadipocytes_ENCODE_2___443	Aran_et_al_2017	FGF7, HAS1, SSR1, TMEM11, BUB3, HAND2, BAG3, MORF4L2, NCKAP1, KDELR2, GLT8D1, ALG8, SLC25A32
Preadipocytes_ENCODE_3___444	Aran_et_al_2017	ADH5, ALCAM, SLC25A6, ANXA1, ANXA5, ARCN1, RHOA, ARL1, ARSB, ATP5C1, ATP5G1, CANX, CAST, CDK7, COPA, COPB1, ATF6B, DPAGT1, TOR1A, ECT2, ELK1, EPRS, FGF7, GLG1, GRSF1, HAS1, HDLBP, HIF1A, HSPA4, IARS, ISLR, MOCS2, NACA, NDUFB4, OCRL, P4HB, PPIB, PTGIR, PTPN11, PURA, RARS, RNF2, RPN2, RRM1, SNAPC2, SSR1, TESK1, TNXB, HSP90B1, PTP4A2, GNPAT, EIF3H, MBTPS1, TMEM11, WISP1, EIF2S2, MTMR2, WASL, MPZL1, BUB3, COPB2, KIF3B, HAND2, FXR2, BAG3, BAG2, MINPP1, MORF4L2, LAPTM4A, RANBP9, RAD50, CNPY2, TUBA1B, YAP1, TMEM147, SYNCRIP, APPBP2, MYL12A, YKT6, DCTN6, NCKAP1, PGRMC1, YIF1A, COPS8, HNRNPA0, SEC61B, ASCC3, METAP2, KDELR2, DSTN, PWP1, MRPL3, SEC63, XPOT, SCRG1, DOLK, ICMT, MKRN2, LMOD1, FAM98A, TMEM87A, DHRS7B, TCTN3, MYOF, NPTN, MMADHC, SEC61A1, COQ6, TMED7, FCF1, COPS4, DDX47, MBTPS2, UCHL5, ATP6V1D, RWDD1, UFC1, ERGIC3, C14orf166, MRPL20, IMPACT, RIOK2, GLT8D1, IFT46, SRPRB, EDA2R, CCDC90B, RIC8A, RNF25, EBF2, ACBD3, C7orf25, C7orf26, ALG8, TBC1D17, METTL8, PODNL1, ADM2, SLC25A32, ADAMTS12, MAGT1, ADO, SLC38A10, LRRC15, PRRC1, SENP5, TIPRL, GTF2H5
Preadipocytes_FANTOM_1___445	Aran_et_al_2017	ADCYAP1R1, AQP9, CYP19A1, HIST1H2BB, IRF4, IPO5, SLC1A2, TNXB, IPO7, ADAMTS8, ABCA6, FAM98A, SNED1, PIWIL2, EDA2R, EBF2, TRPM3, IL17RC, TBC1D16
Preadipocytes_FANTOM_2___446	Aran_et_al_2017	ARHGAP6, ACE, HAS1, IRF4, TNXB, ADAMTS8, ABCA6, SNED1, EBF2
Preadipocytes_FANTOM_3___447	Aran_et_al_2017	ADCYAP1R1, AQP9, ARHGAP6, CENPE, CYP19A1, ACE, GLUD1, HIST1H2BB, HAS1, IRF4, IPO5, LSP1, SLC1A2, TNXB, XPNPEP2, HIST1H2BM, S1PR2, IPO7, ADAMTS8, MRPS27, SMC5, MDN1, ABCA6, FAM98A, SNED1, TREM1, PIWIL2, EDA2R, EBF2, TRPM3, IL17RC, TBC1D16
pro_Bcells_HPCA_1___448	Aran_et_al_2017	AFM, ALPL, CCR8, CRX, DCC, DNTT, GABRA6, GCK, GPR3, GPR4, GRIK3, GYS2, TLX2, KNG1, MEN1, MUSK, OMD, PARK2, POU3F1, RAG2, RPE65, SLC2A2, SLC12A1, SRY, VPREB1, CSRP3, HIST1H2BM, SOX14, CACNA1G, GREB1, CNKSR1, ARPP21, FRS3, LILRB1, CLDN14, PLA2G2D, OR7A5, AKAP8L, STRN4, GNL2, TNFRSF12A, LARP7, BTBD7, SPATA7, HAMP, C2orf49, CXorf36, LRRTM4, FIP1L1, SLC25A31, SCRT1, IMP4, HSPB6, ZNF81, ZNF674
pro_Bcells_HPCA_2___449	Aran_et_al_2017	ADARB2, ADCY8, AGXT, ALOX15B, ANXA3, APOC3, ARG1, ART4, BMX, CA1, CCKAR, SIGLEC6, CETP, CLCN1, CNTFR, CRH, CSHL1, CTSG, CYP2A7, DCC, DLX4, DNTT, DPYS, DSP, EFNA2, FCAR, FCN2, FGF8, MSTN, GDF10, GPR3, GPR4, GRIK3, GYS2, HCRTR2, ONECUT1, PRMT1, HTR1B, HTR5A, IFNA1, IGLL1, IL12B, KCNJ9, KCNJ13,

		KRT12, KRT19, LLGL1, LTC4S, MAG, MKI67, TRPM1, MUC6, MYH4, MYH8, NEUROG1, NOTCH4, OMG, PARK2, PMP2, POU1F1, PPEF2, PRB4, PSG11, RAD23A, RAG2, RAPSN, RBP3, MRPL12, RRM2, CCL17, TACR3, TCF3, TESK1, TGM3, THPO, TNNT2, UCP3, VPREB1, ZNF155, CSRP3, HIST1H2BL, HIST1H2BM, SOX14, EDF1, ADAM21, KCNQ4, OTOF, KIF23, CNKSR1, NMUR1, PLK4, ARPP21, FRS3, LILRB1, STIP1, LILRB4, KERA, ADAMTS8, CLCA4, FSTL4, PMPCA, ATP1B4, CLDN14, PADI4, TSSK2, CA14, FBXO24, SLC13A4, OR7A5, AKAP8L, DKKL1, AHDC1, BMP10, VPREB3, ANAPC2, VSX1, CALY, HP1BP3, PDE11A, CLEC1A, GMIP, TNFRSF12A, SPTBN5, ZMYND10, LARP7, COQ3, CNGB3, ZNF407, IQCC, BTBD7, SPATA7, NXF3, PAPOLB, RPGRIP1, SLURP1, SPC25, HAMP, MYL7, KLHL12, FBRS, RNF25, TUT1, MRPS15, KRI1, NOL12, CXorf36, KRTAP1-3, PCDH11Y, IMP4, HSPB6, PPP4R2, KCNV2, PSORS1C2, R3HCC1, ASPM, NACA2
pro_Bcells_HPCA_3___450	Aran_et_al_2017	ARG1, CLCN1, CNTFR, CSHL1, DCC, DNTT, FCAR, FGF8, GPR3, HTR1B, IGLL1, LTC4S, MEP1B, TRPM1, MUC6, PRB4, PSG11, RAG2, MRPL12, SGCA, TACR3, TCOF1, TESK1, TGM3, VPREB1, ZNF155, HIST1H2BL, SOX14, ADAM21, KCNQ4, ARPP21, FRS3, LILRB1, ADAMTS8, LAMB4, CLDN14, FBXO24, AKAP8L, AHDC1, BMP10, VSX1, PCDHA5, OTUD7B, SPC25, HAMP, MYL7, DPEP3, C2orf49, CXorf36, IMP4, HSPB6
pro_Bcells_NOVERSHTERN_1___451	Aran_et_al_2017	AZU1, BLK, CD72, CD79B, CENPA, CETP, DNTT, FOXM1, FLT3, H2AFX, IGLL1, KIF11, LY6H, MKI67, MYBL2, PDE6D, POLA1, PRTN3, RAG2, RFC2, RFC5, RRM2, SMARCA4, SNRPD1, SPTA1, TCF3, TERT, TOP2B, TSSC1, VPREB1, XPNPEP2, PTTG1, TCL1B, ESPL1, KIF14, P2RY14, SMC4, TACC3, NOP56, SIVA1, ARPP21, HNRNPA0, UBE2C, KIF4A, OR7A5, VPREB3, TRA2A, SAC3D1, MRTO4, NUSAP1, AHSP, GTSE1, CEP55, QRSL1, SPC25, LSM2, CCDC81, SHCBP1, C16orf59, HPS4
pro_Bcells_NOVERSHTERN_2___452	Aran_et_al_2017	BLK, CD72, CD79B, DNTT, FLT3, IGLL1, PRTN3, RAG2, VPREB1, ARPP21, VPREB3, QRSL1, CCDC81
pro_Bcells_NOVERSHTERN_3___453	Aran_et_al_2017	BLK, CD72, CD79B, DNTT, FLT3, IGLL1, LY6H, MYBL2, PRTN3, RAG2, VPREB1, ARPP21, VPREB3, QRSL1, CCDC81
Sebocytes_FANTOM_1___454	Aran_et_al_2017	CALML3, CSF2, CSTA, CTSK, DSG3, GJB5, SFN, IL1A, IRF6, KRT6B, LAD1, MMP3, PI3, PKP1, SOX15, SULT2B1, KRT75, FGFBP1, AP1M2, PDZK1IP1, FST, IL24, LPAR3, TFCP2L1, HES2, TMEM40, LTB4R2, RAB25, S100A14, C1orf116, ZNF750, KRT6C
Sebocytes_FANTOM_2___455	Aran_et_al_2017	CALML3, CSF2, CSTA, CTSK, DSG3, GJB5, SFN, IL1A, KRT6B, MMP3, PI3, PKP1, SOX15, SULT2B1, KRT75, FGFBP1, AP1M2, PDZK1IP1, HES2, TMEM40, RAB25, S100A14, ZNF750, KRT6C
Sebocytes_FANTOM_3___456	Aran_et_al_2017	CALML3, ENTPD3, CSF2, CSTA, CTSK, DSG3, GJB3, GJB5, SFN, IL1A, IRF6, KRT6B, LAD1, MMP3, PI3, PKP1, PTK6, SOX15, SULT2B1, KRT75, FGFBP1, AP1M2, PDZK1IP1, FST, IL24, LPAR3, TFCP2L1, HES2, TMEM40, LTB4R2, RAB25, S100A14, C1orf116, ZNF750, KRT6C
Skeletal_muscle_ENCODE_1___457	Aran_et_al_2017	CAV3, CDH15, CHRNG, MYBPH, MYF5, RAPSN, MYLPF, EBF2
Skeletal_muscle_ENCODE_2___458	Aran_et_al_2017	CAV3, CDH15, CHRNG, MYBPH, MYF5, MYOD1, RAPSN, MYLPF, LSM2, EBF2
Skeletal_muscle_ENCODE_3___459	Aran_et_al_2017	CDK1, CENPE, CHRNG, MYBPH, MYOG, RAPSN, MYLPF, EBF2
Skeletal_muscle_FANTOM_1___460	Aran_et_al_2017	ACHE, ACTA1, ACTN2, AMPD1, ART1, ART3, ATP1A2, ATP2A1, CA3, CACNA1S, CACNB1, CACNG1, CASQ1, CDH15, CHRND, CHRNG, CKM, CNTFR, COX6A2, DES, RAPGEF1, HADHB, HRC, KCNN3, MUSK, MYBPC1, MYBPC2, MYBPH, MYF6, MYH1, MYH2, MYH6, MYH7, MYL1, MYL2, MYL3, MYOD1, MYOG, NRAP, PCNT, PGAM2, PGM1, PHKG1, PPP1R3A, PYGM, RAD23A, RAPSN, RPL3L, CLIP1, RYR3, MAPK12, SGCA, SGCG, SLC2A4, SLN, SPTB, TNNC2, TNNI1, TNNT3, TTN, UCP3, CSRP3, TCAP, MYOM1, FBP2, MYOT, BAG3, ABCC9, TRDN, UBAC1, SEMA6C, LBX1, APOBEC2, LDB3, SIRT2, IQSEC2, DDN, MAST2, KIAA0368, HSPB7, CTNNA3, MYLPF, UBE2D4, ASB4, FBXO40, MYOZ2, MIOS, CASZ1, MYOZ1, POPDC2, LONRF3, SYNPO2L, OBSCN, OSBPL11
Skeletal_muscle_FANTOM_2___461	Aran_et_al_2017	ACTA1, ACTN2, AMPD1, ART1, ART3, ATP1A2, ATP2A1, CA3, CACNA1S, CACNB1, CACNG1, CASQ1, CDH15, CHRND, CHRNG, CKM, COX6A2, DES, HRC, KCNN3, MYBPC1, MYBPC2, MYBPH, MYF6, MYH1, MYH2, MYH6, MYH7, MYL1, MYL2, MYL3, MYOG, NRAP, PGAM2, PHKG1, PPP1R3A, PYGM, RAPSN, RPL3L, SGCA, SGCG, SLC2A4, SLN, SPTB, TNNC2, TNNI1, TNNT3, TTN, UCP3, CSRP3, TCAP, MYOM1, FBP2, MYOT, TRDN, SEMA6C, APOBEC2, LDB3, IQSEC2, DDN, CTNNA3, MYLPF, FBXO40, MYOZ2, MYOZ1, POPDC2, SYNPO2L, OBSCN, OSBPL11
Skeletal_muscle_FANTOM_3___462	Aran_et_al_2017	ACTA1, ACTN2, AMPD1, ART1, ART3, ATP1A2, ATP2A1, CA3, CACNA1S, CACNB1, CACNG1, CASQ1, CDH15, CHRND, CHRNG, CKM, COX6A2, DES, HADHB, HRC,

		KCNN3, MUSK, MYBPC1, MYBPC2, MYBPH, MYF6, MYH1, MYH2, MYH6, MYH7, MYL1, MYL2, MYL3, MYOG, NRAP, PCNT, PGAM2, PHKG1, PPP1R3A, PYGM, RAPSN, RPL3L, CLIP1, SGCA, SGCG, SLC2A4, SLN, SPTB, TNNC2, TNNI1, TNNT3, TTN, UCP3, CSRP3, TCAP, MYOM1, FBP2, MYOT, TRDN, SEMA6C, LBX1, APOBEC2, LDB3, IQSEC2, DDN, HSPB7, CTNNA3, MYLPF, ASB4, FBXO40, MYOZ2, CASZ1, MYOZ1, POPDC2, SYNPO2L, OBSCN, OSBPL11
Smooth_muscle_ENCODE_1___463	Aran_et_al_2017	COPA, COPB1, FKTN, HDLBP, PFN2, PRKAG1, PRKG1, USO1, ASAP2, BAG2, PREPL, VTI1B, HSPB6
Smooth_muscle_ENCODE_2___464	Aran_et_al_2017	ARL1, COPA, COPB1, DCTN1, DYNC1LI2, FKTN, HDLBP, HOXA3, LGALS1, PFN2, PRKAG1, PRKG1, TCF21, GDF5, USO1, MBTPS1, ASAP2, COPS2, VTI1B, ANAPC13, MYOF, AMZ2, KLHL9, ACBD3, DYNLRB1, HSPB6
Smooth_muscle_ENCODE_3___465	Aran_et_al_2017	ADH1B, ALCAM, ARCN1, ARL1, COPA, COPB1, DCTN1, DYNC1LI2, EIF4G2, FKTN, HDLBP, HOXA3, HPD, ISLR, IPO5, LGALS1, NBR1, OCRL, OGN, PFN2, PRKAG1, PRKG1, SDF2, TCF21, GDF5, USO1, MBTPS1, ASAP2, COPS2, KIF3B, SCAMP1, BAG2, PREPL, TTC37, CRYZL1, STAM2, VTI1B, ERLIN1, FAF2, EID1, ANAPC13, MYOF, INVS, COPS7A, DCTN4, AMZ2, UFSP2, KLHL9, THAP10, IFT46, EDA2R, PKNOX2, ACBD3, MRPL40, DYNLRB1, HSPB6
Smooth_muscle_FANTOM_1___466	Aran_et_al_2017	ADD1, ADH1B, ADH5, ALCAM, ANXA5, ARCN1, ARF4, ARHGAP6, ARL1, BAD, CACNA1C, CAPNS1, CETN2, COPA, COPB1, CSNK1G3, CSNK2A2, CTNNA1, DCTN1, DDB1, DYNC1LI2, DPT, EIF4G2, ELN, FKTN, FGF7, FSHB, GDF10, GOLGA3, GRSF1, HDLBP, HLCS, HOXA3, HPD, HSP90AB1, ISLR, IPO5, KRT19, KTN1, LGALS1, LTC4S, NBR1, SMAD5, MEA1, 44076, NFATC4, OCRL, OGN, PCDHGC3, PEX12, PFN2, PHKG1, PPP2R1A, PRKAG1, PRKG1, PSMB7, PTPN11, RARS, RBMS1, RFX2, RING1, S100A6, S100A10, ATXN2, SDF2, SGCD, SNTB2, SSR1, MAP3K7, TCF21, CLEC3B, VCL, VIM, GDF5, USO1, MBTPS1, ASAP2, ZMYM4, COPS2, VAMP3, TXNL1, KIF3B, SCAMP1, BAG2, SPAG7, PREPL, TTC37, TOMM20, CUL7, CRYZL1, SNUPN, ABI2, STAM2, ZMPSTE24, SPEG, TFG, YAP1, VTI1B, ERLIN1, IGF2BP3, FAM189B, SPIN1, KDELR1, ASCC3, KDELR2, RER1, EMILIN1, SCRG1, EPN2, TRIM32, WDFY3, FBXO21, ERC1, SNX13, GANAB, SCFD1, EXOC7, DNAJC13, EID1, TMEM59L, CIZ1, LMOD1, TMEM184B, ANAPC13, FAM98A, GORASP2, MYOF, TBL2, MLH3, DKKL1, HSPB7, INVS, TUBG2, METTL5, TNPO2, COPS7A, CCDC53, DCTN4, AMZ2, NGRN, MBTPS2, ANKFY1, ERGIC3, MYOZ2, DHX29, TMED9, ASPN, FBXL12, DALRD3, UFSP2, EXOC1, SPATA7, GLT8D1, TMEM165, KLHL9, THAP10, IFT46, ZNF471, SRPRB, EDA2R, CCDC90B, MRPL17, PKNOX2, ACBD3, MRPL40, SPATS2, C11orf95, YIPF2, CUEDC2, DDA1, TSEN34, FTO, C2orf49, ZNF426, SPAG16, TBC1D17, CCDC102B, SLC35E1, SVEP1, NETO2, DYNLRB1, TM2D1, COG7, TEX261, HSPB6, PRRC1, MYL6B, ZNF358, SIX5, DPY19L4, C6orf120, GTF2H5
Smooth_muscle_FANTOM_2___467	Aran_et_al_2017	ARL1, COPA, COPB1, DCTN1, DYNC1LI2, FKTN, HDLBP, HOXA3, LGALS1, PFN2, PRKAG1, PRKG1, TCF21, GDF5, USO1, MBTPS1, ASAP2, COPS2, VTI1B, ANAPC13, MYOF, AMZ2, KLHL9, ACBD3, DYNLRB1, HSPB6
Smooth_muscle_FANTOM_3___468	Aran_et_al_2017	ADD1, ALCAM, ARF4, COPA, DCTN1, DPT, FKTN, FGF7, GDF10, HDLBP, HOXA3, HPD, ISLR, LGALS1, NBR1, SMAD5, NFATC4, OCRL, OGN, PCDHGC3, PEX12, PFN2, PRKG1, S100A6, S100A10, ATXN2, SSR1, TCF21, CLEC3B, GDF5, ZMYM4, COPS2, TXNL1, SCAMP1, BAG2, TTC37, CUL7, ABI2, SPIN1, KDELR1, EMILIN1, EXOC7, EID1, TMEM59L, CIZ1, FAM98A, HSPB7, AMZ2, TMED9, ASPN, SPATA7, KLHL9, THAP10, ZNF471, EDA2R, CCDC90B, ACBD3, C11orf95, FTO, SPAG16, CCDC102B, SVEP1, DYNLRB1, HSPB6, MYL6B, ZNF358
Smooth_muscle_HPCA_1___469	Aran_et_al_2017	CDK4, LGALS1, PLS3, SOD1, TMED7, DDX47, UFC1, CISD1
Smooth_muscle_HPCA_2___470	Aran_et_al_2017	CCNG1, CETN2, NDUFS4, PLS3, RPL10, RPS19, S1PR2, RNF7, MCTS1, DDX47, POMP, TMED9, ADAMTS12, GTF2H5
Smooth_muscle_HPCA_3___471	Aran_et_al_2017	ACTG1, ADH5, ALDOA, ANXA1, ANXA5, RHOC, ATP5G1, ATP5J, BYSL, PTTG1IP, CALR, CANX, CAPN2, CCNG1, CDK4, CDK7, CETN2, CNN2, COPA, COPB1, COX8A, ECT2, EEF1D, ERCC1, FKTN, GOLGA3, HADHA, HDLBP, HIC1, HSPA8, EIF6, LAMP1, LGALS1, MIF, MYL6, NDUFA8, NDUFB4, NDUFS4, NDUFS5, 44076, NFATC4, P4HB, PLS3, POLR2F, PSMB1, PSMB4, PSMB5, PSMC5, PSMD10, PTGIR, PTPN11, PEX2, RARS, RPL4, RPL10, RPL35A, RPN2, RPS19, CCL8, SOD1, SSBP1, ST13, TPI1, TPM3, HSP90B1, UFD1L, VCP, MANF, SHFM1, PTP4A2, EIF3I, MBTPS1, MTMR2, ATP6V0E1, MPZL1, BUB3, COPB2, S1PR2, KIF3B, TMEM59, BAG3, C14orf2, MINPP1, RNF7, MORF4L2, LAPTM4A, RWDD2B, PSMD14, CDIPT, TIMM17A, ZNHIT1, AHSA1, NCKAP1, PGRMC1, SEC61B, TMED1, EMILIN1, POFUT2, ISCU, SEC61G, STX12, LMOD1, ATXN10, FAM98A, TMEM87A, FBXO22, UQCRQ, MMADHC, MCTS1, MRPL15, CNIH4, TMED7, DDX47, GMPR2, ZNF771, POMP, ATP6V1D, TRAPPC4, UFC1,

		CUTA, ERGIC3, MRPS33, RIN2, TMED9, C19orf24, NOP10, CISD1, PCDHGB5, SRPRB, C12orf10, EDA2R, FAM160B2, MRPS11, SPATS2, CUEDC2, TBC1D17, MYCT1, ADAMTS12, DYNLRB1, TM2D1, LRRC15, PRRC1, SENP5, GTF2H5
Tgd_cells_HPCA_1___472	Aran_et_al_2017	AK2, ABCD2, FASLG, BUB1, CCNF, CD2, CD247, CD40LG, CDC25C, CENPA, CHEK1, CLIC1, CCR3, CCR5, DAXX, DR1, ECT2, GLE1, GLO1, GPI, CXCR3, GPR15, GYG1, GZMH, GZMA, GZMB, GZMK, H2AFX, HMOX2, IL2RA, IL2RB, IL4, IL5, IL12RB1, IL13, ITGAL, ITGB7, KIF2A, KIF11, KIF22, LAG3, LAIR2, LCP2, LIM2, LTA, MKI67, MSH3, NEK2, NKG7, PDE4A, PDE6D, SLC26A4, PFN1, PPID, PPP1CA, PRF1, PSMA3, PSMB2, PSMC4, PSMD13, PTPN4, PTPN7, PTPN9, PEX2, RBL1, RPA1, RRM1, RRM2, SOS1, AURKA, TMPO, ZBTB16, ZNF174, PTP4A2, GPR68, CLPP, DGCR14, RANBP3, HAT1, RGS9, IL18RAP, TOP3B, TAF1B, PSTPIP1, CD101, GRAP2, ATG5, DLGAP5, KIF14, ARPC2, RAD50, KLRG1, CD96, SF3B4, HMGN4, GNLY, SMC2, CXCR6, GMEB1, PLK4, POP4, DBF4, STIP1, KIF2C, HNRNPUL1, GLMN, CD300A, RALY, TPX2, NCDN, FAM120A, NCAPD3, RNF167, TOR1AIP1, TINF2, ZBTB32, GPKOW, CHMP4A, RACGAP1, SAC3D1, GPR171, TBX21, NUSAP1, UCHL5, FAM96B, HSPB11, WBP11, CD244, ARMC1, CEP55, ASXL2, UEVLD, HJURP, CHST12, IL26, CENPJ, CENPN, ZMAT5, KLHL7, SPC25, IKZF4, MMP25, ACD, PVRIG, ZNF668, ATP8B4, VANGL1, ARPC5L, MTDH, ACTR8, SFXN1, NCR3, CCR2
Tgd_cells_HPCA_2___473	Aran_et_al_2017	ABCD2, FASLG, BUB1, CCNA2, CCNF, CD2, CD247, CDK1, CDC5L, CDC25C, CENPA, CLIC1, CCR3, CCR5, COX8A, CSTF1, DAXX, DR1, DRG2, TOR1A, ECT2, GLE1, GLO1, GPI, CXCR3, GPR15, GYG1, GZMH, GZMA, GZMB, GZMK, H2AFX, HIC1, HMOX2, IFNG, IL2RA, IL2RB, IL4, IL5, IL12RB1, IL13, INPP4A, ITGAL, ITGB7, LAG3, LAIR2, LCP2, LIM2, LTA, MKI67, MNAT1, MSH3, NEK2, NFKBIB, NMT1, PDE4A, PDE6D, SLC26A4, PPID, PPP1CA, PRF1, PSMA1, PSMA3, PSMB2, PSMC4, PSMD13, PTPN4, PTPN7, PTPN9, PEX2, RBL1, RNF6, RRM1, RRM2, SOS1, SRF, AURKA, TMPO, TTK, ZBTB16, ZNF174, PTP4A2, GPR68, CLPP, DGCR14, RANBP3, RGS9, CDC123, TOP3B, TAF1B, PSTPIP1, CIAO1, CD101, GRAP2, ATG5, STX8, DLGAP5, ARPC2, PSMD14, KLRG1, CD96, DCAF7, SF3B4, GNLY, SMC2, CXCR6, GMEB1, PLK4, POP4, ARPP19, DBF4, STIP1, KIF2C, FAF1, GLMN, PUF60, TPX2, NCDN, FAM120A, RNF167, TOR1AIP1, ZBTB32, GPKOW, ZCCHC4, CHMP4A, GPR171, TBX21, ASCC1, DBR1, GMIP, UCHL5, PIAS4, FAM96B, HSPB11, WBP11, SASH3, COMMD8, ARMC1, CEP55, ASXL2, UEVLD, HJURP, TDP1, IL26, CENPN, KLHL7, SPC25, IKZF4, MMP25, MRPS15, ACD, PVRIG, ZNF668, ATP8B4, SLC25A32, VANGL1, ARPC5L, MTDH, ACTR8, SFXN1, NCR3, TIPRL, TMEM110, CCR2
Tgd_cells_HPCA_3___474	Aran_et_al_2017	ABCD2, FASLG, BARD1, BUB1, CCNA2, CCNF, CD2, CD247, CD40LG, CDK1, CDC25C, CENPA, CLIC1, CCR3, CCR5, COX8A, CTLA4, DAXX, DR1, DRG2, ECT2, GLE1, GLO1, GPI, CXCR3, GPR15, GYG1, GZMH, GZMA, GZMB, GZMK, H2AFX, HIC1, HMOX2, HNRNPF, IFNG, IL2RA, IL2RB, IL4, IL5, IL12RB1, IL13, INPP4A, ITGAL, ITGB7, KIF2A, KIF22, LAG3, LAIR2, LCP2, LIM2, LTA, SH2D1A, MKI67, MNAT1, MSH3, NEK2, NFKBIB, NKG7, NMT1, PDE4A, PDE6D, SLC26A4, PPID, PPP1CA, PRF1, PSMA3, PSMB2, PSMC4, PSMD4, PSMD7, PSMD13, PTPN4, PTPN7, PTPN9, PEX2, RAD21, RB1, RBL1, RRM1, CCL1, SHMT2, SLAMF1, SOS1, AURKA, TMPO, USP1, ZBTB16, ZNF174, PTP4A2, GPR68, CLPP, DGCR14, COLQ, RANBP3, RGS9, CDC123, TOP3B, TAF1B, PSTPIP1, CIAO1, CD101, GRAP2, ATG5, STX8, DLGAP5, MELK, G3BP2, KIF14, DCLRE1A, SCAMP2, ARPC2, RAD50, PSMD14, KLRG1, CD96, SF3B4, TACC3, GNLY, SMC2, CXCR6, GMEB1, PLK4, POP4, ARPP19, DBF4, STIP1, KIF2C, GLMN, CD300A, TPX2, TAB2, NCDN, FAM120A, RNF167, TOR1AIP1, ZBTB32, GPKOW, ZCCHC4, CHMP4A, GPR171, TBX21, ASCC1, DBR1, GMIP, AMZ2, FZR1, UCHL5, FAM96B, HSPB11, WBP11, CD244, CSNK1G1, SASH3, RC3H2, COMMD8, AGGF1, CEP55, ASXL2, UEVLD, HJURP, IL26, CENPN, PBK, KLHL7, SPC25, HIVEP3, IKZF4, MMP25, ACD, PVRIG, ZNF668, ATP8B4, SLC25A32, VANGL1, MFSD5, MTDH, ACTR8, SFXN1, NCR3, TIPRL, TMEM110, CCR2
Th1_cells_IRIS_1___475	Aran_et_al_2017	CHD4, CSTF1, IFNG, LAG3, MNAT1, POLD2, PPM1G, SLAMF1, SNRPC, THOP1, CDC123, EIF2B2, FIBP, CHD1L, MDC1, TRIM28, GNLY, RUVBL2, NCAPD3, R3HDM1, TACO1, TMEM39B, UBAP2, CUEDC2
Th1_cells_IRIS_2___476	Aran_et_al_2017	IFNG, LAG3, SNRPC, GNLY, RUVBL2, NCAPD3, TACO1, TMEM39B, UBAP2, CUEDC2
Th1_cells_IRIS_3___477	Aran_et_al_2017	COX10, IFNG, LAG3, PSMD3, SNRPC, EIF2B2, PKMYT1, PTTG1, KIF20A, RNPS1, TTLL5, NUP205, ZBTB32, HTRA2, WRAP53, WDR18, CUEDC2
Th2_cells_IRIS_1___478	Aran_et_al_2017	GZMK, IL5, IL13, MAD2L1, RRM2, BAG2, CXCR6, CEP55
Th2_cells_IRIS_2___479	Aran_et_al_2017	IL5, IL13, MAD2L1, BAG2, CXCR6, RRAS2, CEP55, NUP37, NPHP4

Th2_cells_IRIS_3___480	Aran_et_al_2017	GPR15, GZMA, IL5, IL13, SMAD2, CDK2AP1, RGS9, BAG2, SLC25A44, RAD50, CXCR6, TMEM39B, UBAP2, THADA, RNF34, NPHP4
Tregs_BLUEPRINT_1___481	Aran_et_al_2017	CCR3, CTLA4, IL2RA, PLCL1, PPM1B, TTN, ZNF236, STAM, UBE4A, CXCR6, IPCEF1, ICOS, VPS54, LAX1, BANP, ATG2B, ZCCHC8, TULP4, IKZF4, ZMYM1, ZFC3H1, MCM9
Tregs_BLUEPRINT_2___482	Aran_et_al_2017	CTLA4, IL2RA, PLCL1, ZNF236, STAM, IPCEF1, ICOS, BANP, IKZF4
Tregs_BLUEPRINT_3___483	Aran_et_al_2017	CTLA4, IL2RA, PLCL1, PPM1B, ZNF236, STAM, IPCEF1, ICOS, VPS54, BANP, ATG2B, ZCCHC8, TULP4, IKZF4, ZFC3H1
Tregs_FANTOM_1___484	Aran_et_al_2017	CTLA4, IL2RA, PLCL1, ZNF236, STAM, IPCEF1, ICOS, BANP, IKZF4
Tregs_FANTOM_2___485	Aran_et_al_2017	CTLA4, IL2RA, PLCL1, PPM1B, ZNF236, STAM, IPCEF1, ICOS, VPS54, BANP, ATG2B, ZCCHC8, TULP4, IKZF4, ZFC3H1
Tregs_FANTOM_3___486	Aran_et_al_2017	CD5, CD28, CCR4, CCR8, CTLA4, GPR25, IL2RA, IL10RA, ITGB7, KCNA2, PLCL1, RGS1, SPTAN1, HS3ST3B1, MCF2L2, GALNT8, SIT1, ICOS, FOXP3, LRP2BP, TULP4
Tregs_HPCA_1___487	Aran_et_al_2017	CCR4, CCR8, CTLA4, GPR25, IL2RA, KCNA2, LAIR2, RGS1, HS3ST3B1, MCF2L2, ICOS, FOXP3
Tregs_HPCA_2___488	Aran_et_al_2017	CCR4, CCR8, CTLA4, GPR25, IL2RA, KCNA2, LAIR2, HS3ST3B1, MCF2L2, FOXP3
Tregs_HPCA_3___489	Aran_et_al_2017	CCR4, CCR8, CTLA4, GPR25, IL2RA, KCNA2, LAIR2, RGS1, HS3ST3B1, MCF2L2, FOXP3
Bcells___490	Bindea_et_al_2013	ABCB4, BACH2, BCL11A, BLK, BLNK, CCR9, CD19, CD72, COCH, CR2, DTNB, FCRL2, GLDC, GNG7, HLA-DOB, HLA-DQA1, IGHA1, IGHG1, IGHM, IGKC, IGL, KIAA0125, MEF2C, MICAL3, MS4A1, OSBPL10, PNOC, QRSL1, SCN3A, SLC15A2, SPIB, TCL1A, TNFRSF17
CD4_Tcm___491	Bindea_et_al_2013	AQP3, ATF7IP, ATM, CASP8, CDC14A, CEP68, CLUAP1, CREBZF, CYLD, DOCK9, FAM153B, FOXP1, FYB, HNRPH1, INPP4B, KLF12, LOC441155, MAP3K1, MLL, N4BP2L2-IT2, NEFL, NFATC3, PCM1, PCNX, PDXDC2, PHC3, POLR2J2, PSPC1, REPS1, RPP38, SLC7A6, SNRPN, ST3GAL1, STX16, TIMM8A, TRAF3IP3, TXK, TXLNGY, USP9Y
CD4_Tem___492	Bindea_et_al_2013	AKT3, C7orf54, CCR2, DDX17, EWSR1, FLI1, GDPD5, LTK, MEFV, NFATC4, PRKY, TBC1D5, TBCD, TRA, VIL2
CD8_Tcells___493	Bindea_et_al_2013	ABT1, AES, APBA2, ARHGAP8, C12orf47, C19orf6, C4orf15, CAMLG, CD8A, CD8B, CDKN2AIP, DNAJB1, FLT3LG, GADD45A, GZMM, KLF9, LEPROTL1, LIME1, MYST3, PF4, PPP1R2, PRF1, PRR5, RBM3, SF1, SFRS7, SLC16A7, TBCC, THUMPD1, TMC6, TSC22D3, VAMP2, ZEB1, ZFP36L2, ZNF22, ZNF609, ZNF91
DC___494	Bindea_et_al_2013	CCL13, CCL17, CCL22, CD209, HSD11B1, NPR1, PPFIBP2
Eosinophils___495	Bindea_et_al_2013	ABHD2, ACACB, C9orf156, CAT, CCR3, CLC, CYSLTR2, EMR1, EPN2, GALC, GPR44, HES1, HIST1H1C, HRH4, IGSF2, IL5RA, KBTBD11, KCNH2, LRP5L, MYO15B, RCOR3, RNASE2, RRP12, SIAH1, SMPD3, SYNJ1, TGIF1, THBS1, THBS4, TIPARP, TKTL1
Macrophages___496	Bindea_et_al_2013	APOE, ATG7, BCAT1, CCL7, CD163, CD68, CD84, CHI3L1, CHIT1, CLEC5A, COL8A2, COLEC12, CTSK, CXCL5, CYBB, DNASE2B, EMP1, FDX1, FN1, GM2A, GPC4, KAL1, MARCO, ME1, MS4A4A, MSR1, PCOLCE2, PTGDS, RAI14, SCARB2, SCG5, SGMS1, SULT1C2
Mast_cells___497	Bindea_et_al_2013	ABCC4, ADCYAP1, CALB2, CEACAM8, CMA1, CPA3, CTSG, ELA2, GATA2, HDC, HPGD, HPGDS, KIT, LINC01140, MAOB, MLPH, MPO, MS4A2, NR0B1, PPM1H, PRG2, PTGS1, SCG2, SIGLEC6, SLC18A2, SLC24A3, TAL1, TPSAB1, TPSB2, VWA5A
Neutrophils___498	Bindea_et_al_2013	ADARB1, AF107846, ALDH1B1, APBB2, ATL2, BCL2, CDC5L, FGF18, FUT5, FZR1, GAGE2A, IGFBP5, KANK2, LDB3, MAPRE3, MCM3AP, MRC2, NCR1, PDLIM4, PRX, PSMD4, RP5-886K2.1, SGMS1, SLC30A5, SMEK1, SPN, TBXA2R, TCTN2, TINAGL1, TRPV6, XCL1, XCL2, ZNF205, ZNF528, ZNF747
NK_cells___499	Bindea_et_al_2013	ALPL, BST1, CD93, CEACAM3, CREB5, CRISPLD2, CSF3R, CYP4F3, DYSF, FCAR, FCGR3B, FLJ11151, FPR1, FPRL1, G0S2, HIST1H2BC, HPSE, IL8RA, IL8RB, KCNJ15, LILRB2, MGAM, MME, PDE4B, S100A12, SIGLEC5, SLC22A4, SLC25A37, TECPR2, TNFRSF10C, VNN3
Tgd_cells___500	Bindea_et_al_2013	C1orf61, CD160, FEZ1, TARP, TRD, TRGV9
Th1_cells___501	Bindea_et_al_2013	APBB2, APOD, ATP9A, BST2, BTG3, CCL4, CD38, CD70, CMAH, CSF2, CTLA4, DGKI, DOK5, DPP4, DUSP5, EGFL6, GGT1, HBEGF, IFNG, IL12RB2, IL22, LRP8, LRRN3,

		LTA, SGCB, SYNGR3, ZBTB32
Th2_cells___502	Bindea_et_al_2013	ADCY1, AHI1, ANK1, BIRC5, CDC25C, CDC7, CENPF, CXCR6, DHFR, EVI5, GATA3, GSTA4, HELLS, IL26, LAIR2, LIMA1, MB, MICAL2, NEIL3, PHEX, PMCH, PTGIS, SLC39A14, SMAD2, SNRPD1, WDHD1
Tregs___503	Bindea_et_al_2013	FOXP3
aDC___504	Bindea_et_al_2013	CCL1, EBI3, INDO, LAMP3, OAS3
iDC___505	Bindea_et_al_2013	ABCG2, BLVRB, CARD9, CD1A, CD1B, CD1C, CD1E, CH25H, CLEC10A, CSF1R, CTNS, F13A1, FABP4, FZD2, GSTT1, GUCA1A, HS3ST2, LMAN2L, MMP12, MS4A6A, NUDT9, PDXK, PPARG, PREP, RAP1GAP, SLC26A6, SLC7A8, SYT17, TACSTD2, TM7SF4, VASH1
pDC___506	Bindea_et_al_2013	IL3RA
Bcells___507	Charoentong_et_al_2017	CD180, CD79B, BLK, CD19, MS4A1, TNFRSF17, IGHM, GNG7, MICAL3, SPIB, HLA-DOB, IGKC, PNOC, FCRL2, BACH2, CR2, TCL1A, AKNA, ARHGAP25, CCL21, CD27, CD38, CLEC17A, CLEC9A, CLECL1
CD4_Tcells___508	Charoentong_et_al_2017	AIM2, BIRC3, BRIP1, CCL20, CCL4, CCL5, CCNB1, CCR7, DUSP2, ESCO2, ETS1, EXO1, EXOC6, IARS, ITK, KIF11, KNTC1, NUF2, PRC1, PSAT1, RGS1, RTKN2, SAMSN1, SELL, TRAT1
CD4_Tcm___509	Charoentong_et_al_2017	ABHD3, AHNAK, ANXA2P2, AQP3, ATHL1, BMI1, BZW2, CD63, COL4A1, CYLD, ELMO2, FYN, GLIPR1, GSS, IFITM2, ITGB1, ITGB2, KLF5, LSP1, NDUFB9, PKM2, SFXN3, SIRPG, SMAD4, STX4, TRADD, VIM, XRCC6
CD4_Tem___510	Charoentong_et_al_2017	ATM, CASP3, CASQ1, CD300E, DARS, DOCK9, EXOSC9, EZH2, GDE1, IL34, NCOA4, NEFL, PDGFRL, PTGS1, REPS1, SCG2, SDPR, SIGLEC14, SIGLEC6, TAL1, TFEC, TIPIN, TPK1, UQCRB, USP9Y, WIPF1, ZCRB1
CD8_Tcells___511	Charoentong_et_al_2017	ADRM1, AHSA1, C1GALT1C1, CCT6B, CD37, CD3D, CD3E, CD3G, CD69, CD8A, CETN3, CSE1L, GEMIN6, GNLY, GPT2, GZMA, GZMH, GZMK, IL2RB, LCK, MPZL1, NKG7, PIK3IP1, PTRH2, TIMM13, ZAP70
CD8_Tcm___512	Charoentong_et_al_2017	ACTN4, ADAM12, ADCY9, F13A1, FCER1G, FCGR3B, FGF7, FKBP4, GLUD1, GM2A, GUSB, IL1RN, NOL11, NTRK1, RARA, RNF128, SIGLEC1, TNFRSF11A, TOX4, UBA52, ULBP1
CD8_Tem___513	Charoentong_et_al_2017	ACAP1, APOL3, ARHGAP10, ATP10D, C3AR1, CCR5, CD160, CD55, CFLAR, CMKLR1, DAPP1, FCRL6, FLT3LG, GZMM, HAPLN3, HLA-DMB, HLA-DPA1, HLA-DPB1, IFI16, LIME1, LTK, NFKBIA, SETD7, SIK1, TRIB2
Eosinophils___514	Charoentong_et_al_2017	GIPR, KRT18P50, LRMP, FOSB, RRP12, GPR183, NR4A3, ST3GAL6, DEPDC5, PDE6C, PKD2L2, GPR65, IL5RA, P2RY14, DACH1, DAPK2, EMR3
Macrophages___515	Charoentong_et_al_2017	AIF1, CCL1, CCL14, CCL23, CCL26, CD300LB, CNR1, CNR2, EIF1, EIF4A1, FPR1, FPR2, FRAT2, GPR27, GPR77, RNASE2, MS4A2, BASP1, IGSF6, HK3, VNN1, FES, NPL, FZD2, FAM198B, HNMT, SLC15A3, CD4, TXNDC3, FRMD4A, CRYBB1, HRH1, WNT5B
Mast_cells___516	Charoentong_et_al_2017	ADAMTS3, CPA3, CMA1, CTSG, ARHGAP15, CPM, FCN1, FTL, HSPA6, ITGA9, RNASE3, S100A4, SIGLEC8, SLC6A4, PTGS2, EGR3, PILRA
Memory_Bcells___517	Charoentong_et_al_2017	AICDA, CCNA2, CDKN3, CLCN5, ENPP1, FCER1A, FCRL4, MYC, RUNX2, SORL1, SOX5, STAT5A, STAT5B, TLR9
Monocytes___518	Charoentong_et_al_2017	ASGR2, CFP, ASGR1, CD1D, UPK3A, ACTG1, ANXA5, ATP6V1B2, CFL1, DAZAP2, CTBS, EMR4P, HIVEP2, MARCKSL1, MBP, MMP15, PNPLA6, TMBIM6, PQBP1, TEX264, IKZF1
NK_cells___519	Charoentong_et_al_2017	AKT3, AXL, BST2, CDH2, CRTAM, CSF2RA, CTSZ, CXCL1, CYTH1, DAXX, DGKH, DLL4, DPYD, ERBB3, F11R, FAM27A, FAM49A, FASLG, FCGR1A, FN1, FSTL1, FUCA1, GBP3, GLS2, GRB2, LST1, BCL2, CDC5L, FGF18, FUT5, FZR1, GAGE2, IGFBP5, KANK2, LDB3
NKT___520	Charoentong_et_al_2017	BTN2A2, CD101, CD109, CNPY3, CNPY4, CREB1, CRTC2, CRTC3, CSF2, KLRC1, FUT4, ICAM2, IL32, LAMP2, LILRB5, KLRG1, HSPA4, HSPB6, ISM2, ITIH2, KDM4C,

		KIR2DS4, KIRREL3, SDCBP, NFATC2IP, MICB, KIR2DL1, KIR2DL3, KIR3DL1, KIR3DL2, NCR1, FOSL1, TSLP, SLC7A7, SPP1, TREM2, UBASH3A, YBX2, CCDC88A, CLEC1A, THBD, PDPN, VCAM1, EMR1
Neutrophils___521	Charoentong_et_al_2017	CREB5, CDA, CHST15, S100A12, APOBEC3A, CASP5, MMP25, HAL, C1orf183, FFAR2, MAK, CXCR1, STEAP4, MGAM, BTNL8, CXCR2, TNFRSF10C, VNN3
Tgd_cells___522	Charoentong_et_al_2017	ACP5, AQP9, BTN3A2, C1orf54, CARD8, CCL18, CD209, CD33, CD36, CDK5, IL10RB, KLRF1, LGALS1, MAPK7, KLHL7, KRT80, LAMC1, LCORL, LMNB1, MEIS3P1, MPL, FABP1, FABP5, FADD, MFAP3L, MINPP1, RPS24, RPS7, RPS9, ABP1, CCL13
Th1_cells___523	Charoentong_et_al_2017	CD70, TBX21, ADAM8, AHCYL2, ALCAM, B3GALNT1, BBS12, BST1, CD151, CD47, CD48, CD52, CD53, CD59, CD6, CD68, CD7, CD96, CFHR3, CHRM3, CLEC7A, COL23A1, COL4A4, COL5A3, DAB1, DLEU7, DOC2B, EMP1, F12, FURIN, GAB3, GATM, GFPT2, GPR25, GREM2, HAVCR1, HSD11B1, HUNK, IGF2, RCSD1, RYR1, SAV1, SELE, SELP, SH3KBP1, SIT1, SLC35B3, SIGLEC10, SKAP1, THUMPD2, TIGIT, ZEB2, ENC1, FAM134B, FBXO30, FCGR2C, STAC, LTC4S, MAN1B1, MDH1, MMD, RGS16, IL12A, P2RX5, CD97, ITGB4, ICAM3, METRNL, TNFRSF1A, IRF1, HTR2B, CALD1, MOCOS, TRAF3IP2, TLR8, TRAF1, DUSP14
Th2_cells___524	Charoentong_et_al_2017	ASB2, CSRP2, DAPK1, DLC1, DNAJC12, DUSP6, GNAI1, LAMP3, NRP2, OSBPL1A, PDE4B, PHLDA1, PLA2G4A, RAB27B, RBMS3, RNF125, TMPRSS3, GATA3
Tregs___525	Charoentong_et_al_2017	CCL3L1, CD72, CLEC5A, FOXP3, ITGA4, L1CAM, LIPA, LRP1, LRRC42, MARCO, MMP12, MNDA, MRC1, MS4A6A, PELO, PLEK, PRSS23, PTGIR, ST8SIA4, STAB1
aDC___526	Charoentong_et_al_2017	ABCD1, C1QC, CAPG, CCL3L3, CD207, CD302, ATP5B, ATP5L, ATP6V1A, BCL2L1, C1QB, SNURF, SPCS3, CCNA1, CEACAM8, NOS2, SRA1, TNFRSF6B, TREM1, TREML1, RHOA, SLC25A37, TNFSF14, TREML4, VNN2, XPO6, CLEC4C, TNFAIP2, UBD, ACTR3, RAB1A, SLA, HLA-DQA2, SIGLEC5, SLAMF9
iDC___527	Charoentong_et_al_2017	ACADM, AHCYL1, ALDH1A2, ALDH3A2, ALDH9A1, ALOX15, AMT, ARL1, ATIC, ATP5A1, CAPZA1, LILRA5, RDX, RRAGD, TACSTD2, INPP5F, RAB38, PLAU, CSF3R, SLC18A2, AMPD2, CLTB, C1orf162
pDC___528	Charoentong_et_al_2017	CBX6, DAB2, DDX17, HIGD1A, IDH3A, IL3RA, MAGED1, NUCB2, OFD1, OGT, PDIA4, SERTAD2, SIRPA, TMED2, ENG, FCAR, IGF1, ITGA2B, GABARAP, GPX1, KRT23, PROK2, RALB, RETNLB, RNF141, SEC14L1, SEPX1, EMP3, CD300LF, ABTB1, KLHL21, PHRF1
Bcells___529	Rooney_et_al_2015	CD79B, BTLA, FCRL3, BANK1, CD79A, BLK, RALGPS2, FCRL1, HVCN1, BACH2
CD4_Tcells___530	Rooney_et_al_2015	FOXP3, C15orf53, IL5, CTLA4, IL32, GPR15, IL4
CD8_Tcells___531	Rooney_et_al_2015	CD8A
DC___532	Rooney_et_al_2015	LILRA4, CLEC4C, PLD4, PHEX, IL3RA, PTCRA, IRF8, IRF7, GZMB, CXCR3
Macrophages___533	Rooney_et_al_2015	FUCA1, MMP9, LGMN, HS3ST2, TM4SF19, CLEC5A, GPNMB, C11orf45, CD68, CYBB
NK_cells___534	Rooney_et_al_2015	KLRF1, KLRC1
Neutrophils___535	Rooney_et_al_2015	KDM6B, HSD17B11, EVI2B, MNDA, MEGF9, SELL, NLRP12, PADI4, TRANK1, VNN3
Bcells___536	Tirosh_et_al_2016b	CD19, CD79A, CD79B, BLK, MS4A1, BANK1, IGLL3P, FCRL1, PAX5, CLEC17A, CD22, BCL11A, VPREB3, HLA-DOB, STAP1, FAM129C, TLR10, RALGPS2, AFF3, POU2AF1, CXCR5, PLCG2, HVCN1, CCR6, P2RX5, BLNK, KIAA0226L, POU2F2, IRF8, FCRLA, CD37
Endothelial_cells___537	Tirosh_et_al_2016b	PECAM1, VWF, CDH5, CLDN5, PLVAP, ECSCR, SLCO2A1, CCL14, MMRN1, MYCT1, KDR, TM4SF18, TIE1, ERG, FABP4, SDPR, HYAL2, FLT4, EGFL7, ESAM, CXorf36, TEK, TSPAN18, EMCN, MMRN2, ELTD1, PDE2A, NOS3, ROBO4, APOLD1, PTPRB, RHOJ, RAMP2, GPR116, F2RL3, JUP, CCBP2, GPR146, RGS16, TSPAN7, RAMP3, PLA2G4C, TGM2, LDB2, PRCP, ID1, SMAD1, AFAP1L1, ELK3, ANGPT2, LYVE1, ARHGAP29, IL3RA, ADCY4, TFPI, TNFAIP1, SYT15, DYSF, PODXL, SEMA3A, DOCK9, F8, NPDC1, TSPAN15, CD34, THBD, ITGB4, RASA4, COL4A1, ECE1, GFOD2, EFNA1, PVRL2, GNG11, HERC2P2, MALL, HERC2P9, PPM1F, PKP4, LIMS3, CD9, RAI14, ZNF521, RGL2, HSPG2, TGFBR2, RBP1, FXYD6, MATN2, S1PR1, PIEZO1, PDGFA, ADAM15, HAPLN3, APP
Fibroblasts___538	Tirosh_et_al_2016b	FAP, THY1, DCN, COL1A1, COL1A2, COL6A1, COL6A2, COL6A3, CXCL14, LUM, COL3A1, DPT, ISLR, PODN, CD248, FGF7, MXRA8, PDGFRL, COL14A1, MFAP5, MEG3,

		SULF1, AOX1, SVEP1, LPAR1, PDGFRB, TAGLN, IGFBP6, FBLN1, CA12, SPOCK1, TPM2, THBS2, FBLN5, TMEM119, ADAM33, PRRX1, PCOLCE, IGF2, GFPT2, PDGFRA, CRISPLD2, CPE, F3, MFAP4, C1S, PTGIS, LOX, CYP1B1, CLDN11, SERPINF1, OLFML3, COL5A2, ACTA2, MSC, VASN, ABI3BP, C1R, ANTXR1, MGST1, C3, PALLD, FBN1, CPXM1, CYBRD1, IGFBP5, PRELP, PAPSS2, MMP2, CKAP4, CCDC80, ADAMTS2, TPM1, PCSK5, ELN, CXCL12, OLFML2B, PLAC9, RCN3, LTBP2, NID2, SCARA3, AMOTL2, TPST1, MIR100HG, CTGF, RARRES2, FHL2
Macrophages___539	Tirosh_et_al_2016b	CD163, CD14, CSF1R, C1QC, VSIG4, C1QA, FCER1G, F13A1, TYROBP, MSR1, C1QB, MS4A4A, FPR1, S100A9, IGSF6, LILRB4, FPR3, SIGLEC1, LILRA1, LYZ, HK3, SLC11A1, CSF3R, CD300E, PILRA, FCGR3A, AIF1, SIGLEC9, FCGR1C, OLR1, TLR2, LILRB2, C5AR1, FCGR1A, MS4A6A, C3AR1, HCK, IL4I1, LST1, LILRA5, CSTA, IFI30, CD68, TBXAS1, FCGR1B, LILRA6, CXCL16, NCF2, RAB20, MS4A7, NLRP3, LRRC25, ADAP2, SPP1, CCR1, TNFSF13, RASSF4, SERPINA1, MAFB, IL18, FGL2, SIRPB1, CLEC4A, MNDA, FCGR2A, CLEC7A, SLAMF8, SLC7A7, ITGAX, BCL2A1, PLAUR, SLCO2B1, PLBD1, APOC1, RNF144B, SLC31A2, PTAFR, NINJ1, ITGAM, CPVL, PLIN2, C1orf162, FTL, LIPA, CD86, GLUL, FGR, GK, TYMP, GPX1, NPL, ACSL1
Melanocytes___540	Tirosh_et_al_2016b	MIA, TYR, SLC45A2, CDH19, PMEL, SLC24A5, MAGEA6, GJB1, PLP1, PRAME, CAPN3, ERBB3, GPM6B, S100B, FXYD3, PAX3, S100A1, MLANA, SLC26A2, GPR143, CSPG4, SOX10, MLPH, LOXL4, PLEKHB1, RAB38, QPCT, BIRC7, MFI2, LINC00473, SEMA3B, SERPINA3, PIR, MITF, ST6GALNAC2, ROPN1B, CDH1, ABCB5, QDPR, SERPINE2, ATP1A1, ST3GAL4, CDK2, ACSL3, NT5DC3, IGSF8, MBP

Supplementary Data 2A. The grouping of GO terms in Cohort 1.

GO_term	Enrichment in	Type 1	Type 2	Type 3	Type 4
GO_REGULATION_OF_VASCULAR_ENDOTHELIAL_GROWTH_FACTOR_PRODUCTION	NIR	angiogenesis			
GO_REGULATION_OF_VASCULATURE_DEVELOPMENT	NIR	angiogenesis			
GO_POSITIVE_REGULATION_OF_VASCULATURE_DEVELOPMENT	NIR	angiogenesis			
GO_POSITIVE_REGULATION_OF_VASODILATION	NIR	angiogenesis			
GO_CELL_CELL_ADHESION_VIA_PLASMA_MEMBRANE_ADHESION_MOLECULES	PIR	Cell migration	cell adhesion		
GO_HOMOPHILIC_CELL_ADHESION_VIA_PLASMA_MEMBRANE_ADHESION_MOLECULES	PIR	Cell migration	cell adhesion		
GO_NEURON_CELL_CELL_ADHESION	PIR	immune system	cell adhesion		
GO_SYNAPSE_ASSEMBLY	PIR	immune system	cell adhesion		
GO_REGULATION_OF_CELL_CELL_ADHESION	NIR	immune system	cell adhesion		
GO_PROTEIN_BINDING_INVOLVED_IN_CELL_ADHESION	NIR	immune system	cell adhesion		
GO_DESMOSOME	NIR	immune system	cell adhesion		
GO_REGULATION_OF_CELL_ADHESION_MEDIATED_BY_INTEGRIN	NIR	immune system	cell adhesion		
GO_REGULATION_OF_HOMOTYPIC_CELL_CELL_ADHESION	NIR	immune system	cell adhesion		
GO_POSITIVE_REGULATION_OF_CELL_ADHESION_MEDIATED_BY_INTEGRIN	NIR	immune system	cell adhesion		
GO_HETEROTYPIC_CELL_CELL_ADHESION	NIR	immune system	cell adhesion		
GO_NEGATIVE_REGULATION_OF_CELL_CELL_ADHESION	NIR	immune system	cell adhesion		
GO_NEGATIVE_REGULATION_OF_HOMOTYPIC_CELL_CELL_ADHESION	NIR	immune system	cell adhesion		
GO_POSITIVE_REGULATION_OF_CELL_ADHESION	NIR	immune system	cell adhesion		
GO_SINGLE_ORGANISM_CELL_ADHESION	NIR	immune system	cell adhesion		
GO_POSITIVE_REGULATION_OF_CELL_CELL_ADHESION	NIR	immune system	cell adhesion		
GO_LEUKOCYTE_CELL_CELL_ADHESION	NIR	immune system	cell adhesion		
GO_REGULATION_OF_LYMPHOCYTE_APOPTOTIC_PROCESS	NIR	immune system	cell death	apoptosis	
GO_REGULATION_OF_T_CELL_APOPTOTIC_PROCESS	NIR	immune system	cell death	apoptosis	
GO_NEGATIVE_REGULATION_OF_T_CELL_APOPTOTIC_PROCESS	NIR	immune system	cell death	apoptosis	
GO_NEGATIVE_REGULATION_OF_LYMPHOCYTE_APOPTOTIC_PROCESS	NIR	immune system	cell death	apoptosis	
GO_NEGATIVE_REGULATION_OF_LEUKOCYTE_APOPTOTIC_PROCESS	NIR	immune system	cell death	apoptosis	
GO_REGULATION_OF_LEUKOCYTE_APOPTOTIC_PROCESS	NIR	immune system	cell death	apoptosis	
GO_NEGATIVE_REGULATION_OF_CELL_KILLING	NIR	immune system	cell death	cell kill	
GO_REGULATION_OF_CELL_KILLING	NIR	immune system	cell death	cell kill	
GO_CELL_KILLING	NIR	immune system	cell death	cell kill	
GO_NECROTIC_CELL_DEATH	NIR	immune system	cell death	necrosis	
GO_NECROPTOTIC_PROCESS	NIR	immune system	cell death	necrosis	
GO_REGULATION_OF_LYMPHOCYTE_CHEMOTAXIS	NIR	immune system	cell migration		
GO_LEUKOCYTE_CHEMOTAXIS	NIR	immune system	cell migration		
GO_DENDRITIC_CELL_CHEMOTAXIS	NIR	immune system	cell migration		
GO_POSITIVE_REGULATION_OF_CHEMOTAXIS	NIR	immune system	cell migration		
GO_REGULATION_OF_CELLULAR_EXTRAVASATION	NIR	immune system	cell migration		
GO_REGULATION_OF_LEUKOCYTE_CHEMOTAXIS	NIR	immune system	cell migration		
GO_GRANULOCYTE_MIGRATION	NIR	immune system	cell migration		
GO_REGULATION_OF_NEUTROPHIL_MIGRATION	NIR	immune system	cell migration		
GO_REGULATION_OF_CHEMOTAXIS	NIR	immune system	cell migration		
GO_REGULATION_OF_MACROPHAGE_CHEMOTAXIS	NIR	immune system	cell migration		
GO_REGULATION_OF_LEUKOCYTE_MIGRATION	NIR	immune system	cell migration		
GO_CELL_CHEMOTAXIS	NIR	immune system	cell migration		
GO_MYELOID_LEUKOCYTE_MIGRATION	NIR	immune system	cell migration		
GO_POSITIVE_REGULATION_OF_LEUKOCYTE_CHEMOTAXIS	NIR	immune system	cell migration		
GO_REGULATION_OF_GRANULOCYTE_CHEMOTAXIS	NIR	immune system	cell migration		
GO_POSITIVE_REGULATION_OF_LYMPHOCYTE_MIGRATION	NIR	immune system	cell migration		
GO_DENDRITIC_CELL_MIGRATION	NIR	immune system	cell migration		
GO_POSITIVE_REGULATION_OF_NEUTROPHIL_MIGRATION	NIR	immune system	cell migration		
GO_REGULATION_OF_T_CELL_MIGRATION	NIR	immune system	cell migration		
GO_REGULATION_OF_PEPTIDE_TRANSPORT	NIR	immune system	cytokine		
GO_REGULATION_OF_TUMOR_NECROSIS_FACTOR_SUPERFAMILY_CYTOKINE_PRODUCTION	NIR	immune system	cytokine		
GO_REGULATION_OF_INTERLEUKIN_4_PRODUCTION	NIR	immune system	cytokine		
GO_POSITIVE_REGULATION_OF_INTERLEUKIN_1_SECRETION	NIR	immune system	cytokine		
GO_REGULATION_OF_INTERLEUKIN_5_PRODUCTION	NIR	immune system	cytokine		
GO_REGULATION_OF_INTERLEUKIN_2_PRODUCTION	NIR	immune system	cytokine		
GO_POSITIVE_REGULATION_OF_INTERLEUKIN_6_PRODUCTION	NIR	immune system	cytokine		
GO_REGULATION_OF_INTERLEUKIN_6_PRODUCTION	NIR	immune system	cytokine		
GO_POSITIVE_REGULATION_OF_INTERLEUKIN_1_BETA_PRODUCTION	NIR	immune system	cytokine		
GO_REGULATION_OF_IMMUNOGLOBULIN_PRODUCTION	NIR	immune system	cytokine		
GO_NEGATIVE_REGULATION_OF_CYTOKINE_SECRETION	NIR	immune system	cytokine		
GO_POSITIVE_REGULATION_OF_TRANSCRIPTION_FACTOR_IMPORT_INTO_NUCLEUS	NIR	immune system	cytokine		
GO_REGULATION_OF_INTERLEUKIN_12_PRODUCTION	NIR	immune system	cytokine		
GO_POSITIVE_REGULATION_OF_NF_KAPPAB_IMPORT_INTO_NUCLEUS	NIR	immune system	cytokine		
GO_POSITIVE_REGULATION_OF_INTERLEUKIN_1_PRODUCTION	NIR	immune system	cytokine		
GO_NEGATIVE_REGULATION_OF_INTERFERON_GAMMA_PRODUCTION	NIR	immune system	cytokine		
GO_PROTEIN_ACTIVATION_CASCADE	NIR	immune system	cytokine		
GO_POSITIVE_REGULATION_OF_CHEMOKINE_PRODUCTION	NIR	immune system	cytokine		
GO_NEGATIVE_REGULATION_OF_PROTEIN_SECRETION	NIR	immune system	cytokine		
GO_POSITIVE_REGULATION_OF_INTERLEUKIN_8_PRODUCTION	NIR	immune system	cytokine		
GO_REGULATION_OF_INTERLEUKIN_2_BIOSYNTHETIC_PROCESS	NIR	immune system	cytokine		
O_NEGATIVE_REGULATION_OF_TUMOR_NECROSIS_FACTOR_SUPERFAMILY_CYTOKINE_PRODUCTIC	NIR	immune system	cytokine		
GO_POSITIVE_REGULATION_OF_HORMONE_SECRETION	NIR	immune system	cytokine		
GO_POSITIVE_REGULATION_OF_CYTOKINE_PRODUCTION	NIR	immune system	cytokine		
GO_POSITIVE_REGULATION_OF_CYTOKINE_PRODUCTION_INVOLVED_IN_IMMUNE_RESPONSE	NIR	immune system	cytokine		
GO_REGULATION_OF_INTERLEUKIN_1_BETA_PRODUCTION	NIR	immune system	cytokine		
GO_CYTOKINE_PRODUCTION	NIR	immune system	cytokine		
iO_POSITIVE_REGULATION_OF_TUMOR_NECROSIS_FACTOR_SUPERFAMILY_CYTOKINE_PRODUCTIO	NIR	immune system	cytokine		
GO_POSITIVE_REGULATION_OF_INSULIN_SECRETION	NIR	immune system	cytokine		
GO_NEGATIVE_REGULATION_OF_CYTOKINE_BIOSYNTHETIC_PROCESS	NIR	immune system	cytokine		
GO_REGULATION_OF_PROTEIN_SECRETION	NIR	immune system	cytokine		
GO_CYTOKINE_SECRETION	NIR	immune system	cytokine		
GO_REGULATION_OF_INTERLEUKIN_8_PRODUCTION	NIR	immune system	cytokine		
GO_POSITIVE_REGULATION_OF_PROTEIN_MATURATION	NIR	immune system	cytokine		

GO_POSITIVE_REGULATION_OF_INTERLEUKIN_12_PRODUCTION	NIR	immune system	cytokine
GO_REGULATION_OF_PRODUCTION_OF_MOLECULAR_MEDIATOR_OF_IMMUNE_RESPONSE	NIR	immune system	cytokine
GO_REGULATION_OF_INTERLEUKIN_1_SECRETION	NIR	immune system	cytokine
GO_POSITIVE_REGULATION_OF_INTERFERON_GAMMA_PRODUCTION	NIR	immune system	cytokine
GO_REGULATION_OF_INTERLEUKIN_10_PRODUCTION	NIR	immune system	cytokine
GO_POSITIVE_REGULATION_OF_PROTEIN_SECRETION	NIR	immune system	cytokine
GO_NEGATIVE_REGULATION_OF_INTERLEUKIN_2_PRODUCTION	NIR	immune system	cytokine
ITIVE_REGULATION_OF_INSULIN_SECRETION_INVOLVED_IN_CELLULAR_RESPONSE_TO_GLUCOSE_S	NIR	immune system	cytokine
O_POSITIVE_REGULATION_OF_PRODUCTION_OF_MOLECULAR_MEDIATOR_OF_IMMUNE_RESPONS	NIR	immune system	cytokine
GO_POSITIVE_REGULATION_OF_CYTOKINE_SECRETION	NIR	immune system	cytokine
GO_POSITIVE_REGULATION_OF_CYTOKINE_BIOSYNTHETIC_PROCESS	NIR	immune system	cytokine
GO_REGULATION_OF_CYTOKINE_SECRETION	NIR	immune system	cytokine
GO_NEGATIVE_REGULATION_OF_CYTOKINE_PRODUCTION	NIR	immune system	cytokine
GO_POSITIVE_REGULATION_OF_INTERLEUKIN_4_PRODUCTION	NIR	immune system	cytokine
GO_POSITIVE_REGULATION_OF_SECRETION	NIR	immune system	cytokine
GO_POSITIVE_REGULATION_OF_INTERLEUKIN_10_PRODUCTION	NIR	immune system	cytokine
GO_INTERLEUKIN_1_PRODUCTION	NIR	immune system	cytokine
GO_REGULATION_OF_INTERFERON_GAMMA_PRODUCTION	NIR	immune system	cytokine
GO_REGULATION_OF_INTERLEUKIN_1_PRODUCTION	NIR	immune system	cytokine
GO_REGULATION_OF_CYTOKINE_BIOSYNTHETIC_PROCESS	NIR	immune system	cytokine
GO_NEGATIVE_REGULATION_OF_INTERLEUKIN_6_PRODUCTION	NIR	immune system	cytokine
GO_REGULATION_OF_CYTOKINE_PRODUCTION_INVOLVED_IN_IMMUNE_RESPONSE	NIR	immune system	cytokine
GO_NEGATIVE_REGULATION_OF_CYTOKINE_PRODUCTION_INVOLVED_IN_IMMUNE_RESPONSE	NIR	immune system	cytokine
GO_REGULATION_OF_PROTEIN_MATURATION	NIR	immune system	cytokine
GO_NEGATIVE_REGULATION_OF_CHEMOKINE_PRODUCTION	NIR	immune system	cytokine
GO_REGULATION_OF_CHEMOKINE_PRODUCTION	NIR	immune system	cytokine
GO_CYTOKINE_ACTIVITY	NIR	immune system	cytokine
GO_POSITIVE_REGULATION_OF_IMMUNOGLOBULIN_PRODUCTION	NIR	immune system	cytokine
GO_REGULATION_OF_NF_KAPPAB_IMPORT_INTO_NUCLEUS	NIR	immune system	cytokine
GO_COMPLEMENT_ACTIVATION	NIR	immune system	cytokine
GO_REGULATION_OF_TUMOR_NECROSIS_FACTOR_BIOSYNTHETIC_PROCESS	NIR	immune system	cytokine
GO_NEGATIVE_REGULATION_OF_TYPE_I_INTERFERON_PRODUCTION	NIR	immune system	cytokine
GO_POSITIVE_REGULATION_OF_PEPTIDE_SECRETION	NIR	immune system	cytokine
GO_REGULATION_OF_INTERLEUKIN_8_SECRETION	NIR	immune system	cytokine
O_NEGATIVE_REGULATION_OF_PRODUCTION_OF_MOLECULAR_MEDIATOR_OF_IMMUNE_RESPON	NIR	immune system	cytokine
GO_PRODUCTION_OF_MOLECULAR_MEDIATOR_OF_IMMUNE_RESPONSE	NIR	immune system	cytokine
GO_CHEMOKINE_ACTIVITY	NIR	immune system	cytokine
GO_MHC_CLASS_I_PROTEIN_BINDING	NIR	immune system	cytokine
GO_MHC_PROTEIN_BINDING	NIR	immune system	cytokine
GO_NEGATIVE_REGULATION_OF_MULTI_ORGANISM_PROCESS	NIR	immune system	defense response
GO_RESPONSE_TO_PROTOZOAN	NIR	immune system	defense response
GO_DEFENSE_RESPONSE_TO_GRAM_NEGATIVE_BACTERIUM	NIR	immune system	defense response
GO_DETECTION_OF_OTHER_ORGANISM	NIR	immune system	defense response
GO_MODULATION_OF_GROWTH_OF_SYMBIONT_INVOLVED_IN_INTERACTION_WITH_HOST	NIR	immune system	defense response
GO_RESPONSE_TO_VIRUS	NIR	immune system	defense response
GO_DETECTION_OF_BIOTIC_STIMULUS	NIR	immune system	defense response
GO_NEGATIVE_REGULATION_OF_VIRAL_ENTRY_INTO_HOST_CELL	NIR	immune system	defense response
GO_POSITIVE_REGULATION_OF_RESPONSE_TO_EXTERNAL_STIMULUS	NIR	immune system	defense response
GO_DEFENSE_RESPONSE_TO_BACTERIUM	NIR	immune system	defense response
GO_RESPONSE_TO_BACTERIUM	NIR	immune system	defense response
GO_KILLING_OF_CELLS_OF_OTHER_ORGANISM	NIR	immune system	defense response
GO_DISRUPTION_OF_CELLS_OF_OTHER_ORGANISM	NIR	immune system	defense response
GO_RESPONSE_TO_FUNGUS	NIR	immune system	defense response
GO_INFLAMMATORY_RESPONSE_TO_ANTIGENIC_STIMULUS	NIR	immune system	defense response
GO_REGULATION_OF_VIRAL_ENTRY_INTO_HOST_CELL	NIR	immune system	defense response
GO_DEFENSE_RESPONSE_TO_OTHER_ORGANISM	NIR	immune system	defense response
GO_CELLULAR_RESPONSE_TO_INTERFERON_GAMMA	NIR	immune system	immune response
ISE_BASED_ON_SOMATIC_RECOMBINATION_OF_IMMUNE_RECEPTORS_BUILT_FROM_IMMUNOGL	NIR	immune system	immune response
GO_ACTIVATION_OF_INNATE_IMMUNE_RESPONSE	NIR	immune system	immune response
GO_REGULATION_OF_CELL_ACTIVATION	NIR	immune system	immune response
GO_ACUTE_INFLAMMATORY_RESPONSE	NIR	immune system	immune response
GO_POSITIVE_REGULATION_OF_INFLAMMATORY_RESPONSE	NIR	immune system	immune response
GO_POSITIVE_REGULATION_OF_RESPONSE_TO_WOUNDING	NIR	immune system	immune response
GO_REGULATION_OF_PROTEIN_ACTIVATION_CASCADE	NIR	immune system	immune response
GO_REGULATION_OF_ACUTE_INFLAMMATORY_RESPONSE	NIR	immune system	immune response
GO_NEGATIVE_REGULATION_OF_IMMUNE_RESPONSE	NIR	immune system	immune response
GO_POSITIVE_REGULATION_OF_ADAPTIVE_IMMUNE_RESPONSE	NIR	immune system	immune response
GO_INFLAMMATORY_RESPONSE	NIR	immune system	immune response
GO_POSITIVE_REGULATION_OF_INNATE_IMMUNE_RESPONSE	NIR	immune system	immune response
GO_T_HELPER_1_TYPE_IMMUNE_RESPONSE	NIR	immune system	immune response
GO_REGULATION_OF_RESPONSE_TO_WOUNDING	NIR	immune system	immune response
GO_REGULATION_OF_HUMORAL_IMMUNE_RESPONSE	NIR	immune system	immune response
GO_ACUTE_PHASE_RESPONSE	NIR	immune system	immune response
GO_NEGATIVE_REGULATION_OF_IMMUNE_EFFECTOR_PROCESS	NIR	immune system	immune response
GO_POSITIVE_REGULATION_OF_HUMORAL_IMMUNE_RESPONSE	NIR	immune system	immune response
GO_RESPONSE_TO_INTERFERON_GAMMA	NIR	immune system	immune response
GO_POSITIVE_REGULATION_OF_IMMUNE_EFFECTOR_PROCESS	NIR	immune system	immune response
GO_RESPONSE_TO_TUMOR_NECROSIS_FACTOR	NIR	immune system	immune response
GO_CELL_ACTIVATION_INVOLVED_IN_IMMUNE_RESPONSE	NIR	immune system	immune response
GO_NEGATIVE_REGULATION_OF_CELL_ACTIVATION	NIR	immune system	immune response
GO_REGULATION_OF_TYPE_2_IMMUNE_RESPONSE	NIR	immune system	immune response
GO_REGULATION_OF_IMMUNE_EFFECTOR_PROCESS	NIR	immune system	immune response
GO_POSITIVE_REGULATION_OF_DEFENSE_RESPONSE	NIR	immune system	immune response
GO_HUMORAL_IMMUNE_RESPONSE	NIR	immune system	immune response
GO_NEGATIVE_REGULATION_OF_DEFENSE_RESPONSE	NIR	immune system	immune response
GO_ACTIVATION_OF_IMMUNE_RESPONSE	NIR	immune system	immune response
GO_REGULATION_OF_ADAPTIVE_IMMUNE_RESPONSE	NIR	immune system	immune response

GO_IMMUNE_EFFECTOR_PROCESS	NIR	immune system	immune response		
GO_REGULATION_OF_INFLAMMATORY_RESPONSE	NIR	immune system	immune response		
GO_RESPIRATORY_BURST	NIR	immune system	immune response		
GO_POSITIVE_REGULATION_OF_CELL_ACTIVATION	NIR	immune system	immune response		
GO_NEGATIVE_REGULATION_OF_ADAPTIVE_IMMUNE_RESPONSE	NIR	immune system	immune response		
GO_NEGATIVE_REGULATION_OF_INNATE_IMMUNE_RESPONSE	NIR	immune system	immune response		
GO_ADAPTIVE_IMMUNE_RESPONSE	NIR	immune system	immune response		
GO_NEGATIVE_REGULATION_OF_IMMUNE_SYSTEM_PROCESS	NIR	immune system	immune response		
GO_REGULATION_OF_INNATE_IMMUNE_RESPONSE	NIR	immune system	immune response		
NSE_BASED_ON_SOMATIC_RECOMBINATION_OF_IMMUNE_RECEPTORS_BUILT_FROM_IMMUNOGL	NIR	immune system	immune response		
GO_HUMORAL_IMMUNE_RESPONSE	NIR	immune system	lymphoid lineage	B cell	immune response
GO_REGULATION_OF_HUMORAL_IMMUNE_RESPONSE	NIR	immune system	lymphoid lineage	B cell	immune response
GO_POSITIVE_REGULATION_OF_HUMORAL_IMMUNE_RESPONSE	NIR	immune system	lymphoid lineage	B cell	immune response
GO_REGULATION_OF_IMMUNOGLOBULIN_SECRETION	NIR	immune system	lymphoid lineage	B cell	immunoglobulin
GO_HUMORAL_IMMUNE_RESPONSE_MEDIATED_BY_CIRCULATING_IMMUNOGLOBULIN	NIR	immune system	lymphoid lineage	B cell	immunoglobulin
GO_REGULATION_OF_B_CELL_MEDIATED_IMMUNITY	NIR	immune system	lymphoid lineage	B cell	
GO_B_CELL_PROLIFERATION	NIR	immune system	lymphoid lineage	B cell	
GO_POSITIVE_REGULATION_OF_B_CELL_ACTIVATION	NIR	immune system	lymphoid lineage	B cell	
GO_POSITIVE_REGULATION_OF_B_CELL_MEDIATED_IMMUNITY	NIR	immune system	lymphoid lineage	B cell	
GO_REGULATION_OF_B_CELL_DIFFERENTIATION	NIR	immune system	lymphoid lineage	B cell	
GO_REGULATION_OF_B_CELL_ACTIVATION	NIR	immune system	lymphoid lineage	B cell	
GO_B_CELL_ACTIVATION	NIR	immune system	lymphoid lineage	B cell	
GO_B_CELL_MEDIATED_IMMUNITY	NIR	immune system	lymphoid lineage	B cell	
GO_NEGATIVE_REGULATION_OF_B_CELL_ACTIVATION	NIR	immune system	lymphoid lineage	B cell	
GO_MATURE_B_CELL_DIFFERENTIATION	NIR	immune system	lymphoid lineage	B cell	
GO_CD4_POSITIVE_ALPHA_BETA_T_CELL_ACTIVATION	NIR	immune system	lymphoid lineage	T cell	
GO_REGULATION_OF_ALPHA_BETA_T_CELL_DIFFERENTIATION	NIR	immune system	lymphoid lineage	T cell	
GO_POSITIVE_T_CELL_SELECTION	NIR	immune system	lymphoid lineage	T cell	
GO_REGULATION_OF_T_CELL_DIFFERENTIATION	NIR	immune system	lymphoid lineage	T cell	
GO_ALPHA_BETA_T_CELL_DIFFERENTIATION	NIR	immune system	lymphoid lineage	T cell	
GO_THYMIC_T_CELL_SELECTION	NIR	immune system	lymphoid lineage	T cell	
GO_POSITIVE_REGULATION_OF_ALPHA_BETA_T_CELL_ACTIVATION	NIR	immune system	lymphoid lineage	T cell	
GO_T_CELL_RECEPTOR_COMPLEX	NIR	immune system	lymphoid lineage	T cell	
GO_ALPHA_BETA_T_CELL_ACTIVATION	NIR	immune system	lymphoid lineage	T cell	
GO_T_CELL_MEDIATED_IMMUNITY	NIR	immune system	lymphoid lineage	T cell	
GO_NEGATIVE_REGULATION_OF_T_CELL_PROLIFERATION	NIR	immune system	lymphoid lineage	T cell	
GO_NEGATIVE_REGULATION_OF_ALPHA_BETA_T_CELL_ACTIVATION	NIR	immune system	lymphoid lineage	T cell	
GO_NEGATIVE_REGULATION_OF_T_CELL_MEDIATED_IMMUNITY	NIR	immune system	lymphoid lineage	T cell	
GO_REGULATION_OF_T_CELL_PROLIFERATION	NIR	immune system	lymphoid lineage	T cell	
GO_T_CELL_DIFFERENTIATION_IN_THYMUS	NIR	immune system	lymphoid lineage	T cell	
GO_T_CELL_ACTIVATION_INVOLVED_IN_IMMUNE_RESPONSE	NIR	immune system	lymphoid lineage	T cell	
GO_REGULATION_OF_T_HELPER_CELL_DIFFERENTIATION	NIR	immune system	lymphoid lineage	T cell	
GO_REGULATION_OF_ALPHA_BETA_T_CELL_PROLIFERATION	NIR	immune system	lymphoid lineage	T cell	
GO_POSITIVE_REGULATION_OF_CD4_POSITIVE_ALPHA_BETA_T_CELL_ACTIVATION	NIR	immune system	lymphoid lineage	T cell	
GO_T_CELL_SELECTION	NIR	immune system	lymphoid lineage	T cell	
GO_REGULATION_OF_CD4_POSITIVE_ALPHA_BETA_T_CELL_ACTIVATION	NIR	immune system	lymphoid lineage	T cell	
GO_T_CELL_DIFFERENTIATION	NIR	immune system	lymphoid lineage	T cell	
GO_POSITIVE_REGULATION_OF_T_CELL_PROLIFERATION	NIR	immune system	lymphoid lineage	T cell	
GO_T_CELL_LINEAGE_COMMITMENT	NIR	immune system	lymphoid lineage	T cell	
GO_THYMOCYTE_AGGREGATION	NIR	immune system	lymphoid lineage	T cell	
GO_NEGATIVE_REGULATION_OF_T_CELL_DIFFERENTIATION	NIR	immune system	lymphoid lineage	T cell	
GO_POSITIVE_REGULATION_OF_ALPHA_BETA_T_CELL_DIFFERENTIATION	NIR	immune system	lymphoid lineage	T cell	
GO_REGULATION_OF_ALPHA_BETA_T_CELL_ACTIVATION	NIR	immune system	lymphoid lineage	T cell	
GO_REGULATION_OF_T_CELL_MEDIATED_IMMUNITY	NIR	immune system	lymphoid lineage	T cell	
GO_POSITIVE_REGULATION_OF_ALPHA_BETA_T_CELL_PROLIFERATION	NIR	immune system	lymphoid lineage	T cell	
GO_T_CELL_DIFFERENTIATION_INVOLVED_IN_IMMUNE_RESPONSE	NIR	immune system	lymphoid lineage	T cell	
GO_NEGATIVE_REGULATION_OF_CD4_POSITIVE_ALPHA_BETA_T_CELL_ACTIVATION	NIR	immune system	lymphoid lineage	T cell	
GO_NEGATIVE_REGULATION_OF_T_CELL_RECEPTOR_SIGNALING_PATHWAY	NIR	immune system	lymphoid lineage	T cell	
NSE_BASED_ON_SOMATIC_RECOMBINATION_OF_IMMUNE_RECEPTORS_BUILT_FROM_IMMUNOGL	NIR	immune system	lymphoid lineage		immune response
GO_REGULATION_OF_ADAPTIVE_IMMUNE_RESPONSE	NIR	immune system	lymphoid lineage		immune response
GO_NEGATIVE_REGULATION_OF_ADAPTIVE_IMMUNE_RESPONSE	NIR	immune system	lymphoid lineage		immune response
GO_LYMPHOCYTE_ACTIVATION	NIR	immune system	lymphoid lineage		
GO_NATURAL_KILLER_CELL_MEDIATED_IMMUNITY	NIR	immune system	lymphoid lineage		
GO_NEGATIVE_REGULATION_OF_LYMPHOCYTE_DIFFERENTIATION	NIR	immune system	lymphoid lineage		
GO_LYMPHOCYTE_DIFFERENTIATION	NIR	immune system	lymphoid lineage		
GO_REGULATION_OF_LYMPHOCYTE_DIFFERENTIATION	NIR	immune system	lymphoid lineage		
GO_REGULATION_OF_NATURAL_KILLER_CELL_MEDIATED_IMMUNITY	NIR	immune system	lymphoid lineage		
GO_LYMPHOCYTE_MEDIATED_IMMUNITY	NIR	immune system	lymphoid lineage		
GO_POSITIVE_REGULATION_OF_LYMPHOCYTE_MEDIATED_IMMUNITY	NIR	immune system	lymphoid lineage		
GO_NATURAL_KILLER_CELL_ACTIVATION	NIR	immune system	lymphoid lineage		
GO_POSITIVE_REGULATION_OF_LYMPHOCYTE_DIFFERENTIATION	NIR	immune system	lymphoid lineage		
GO_REGULATION_OF_COAGULATION	NIR	immune system	Myeloid lineage		
GO_REGULATION_OF_PLATELET_ACTIVATION	NIR	immune system	Myeloid lineage		
GO_POSITIVE_REGULATION_OF_HEMOPOIESIS	NIR	immune system	Myeloid lineage		
GO_REGULATION_OF_MAST_CELL_ACTIVATION_INVOLVED_IN_IMMUNE_RESPONSE	NIR	immune system	Myeloid lineage		
GO_MYELOID_LEUKOCYTE_ACTIVATION	NIR	immune system	Myeloid lineage		
GO_POSITIVE_REGULATION_OF_LEUKOCYTE_MEDIATED_IMMUNITY	NIR	immune system	Myeloid lineage		
GO_NEGATIVE_REGULATION_OF_LEUKOCYTE_PROLIFERATION	NIR	immune system	Myeloid lineage		
GO_LEUKOCYTE_PROLIFERATION	NIR	immune system	Myeloid lineage		
GO_REGULATION_OF_LEUKOCYTE_DIFFERENTIATION	NIR	immune system	Myeloid lineage		
GO_LEUKOCYTE_DEGRANULATION	NIR	immune system	Myeloid lineage		
GO_REGULATION_OF_LEUKOCYTE_DEGRANULATION	NIR	immune system	Myeloid lineage		
GO_LEUKOCYTE_DIFFERENTIATION	NIR	immune system	Myeloid lineage		
GO_MYELOID_CELL_ACTIVATION_INVOLVED_IN_IMMUNE_RESPONSE	NIR	immune system	Myeloid lineage		
GO_LEUKOCYTE_MEDIATED_CYTOTOXICITY	NIR	immune system	Myeloid lineage		
GO_POSITIVE_REGULATION_OF_MYELOID_LEUKOCYTE_MEDIATED_IMMUNITY	NIR	immune system	Myeloid lineage		
GO_MAST_CELL_MEDIATED_IMMUNITY	NIR	immune system	Myeloid lineage		

GO_MYELOID_LEUKOCYTE_MEDIATED_IMMUNITY	NIR	immune system	Myeloid lineage
GO_POSITIVE_REGULATION_OF_MAST_CELL_ACTIVATION	NIR	immune system	Myeloid lineage
GO_MAST_CELL_ACTIVATION	NIR	immune system	Myeloid lineage
GO_REGULATION_OF_LEUKOCYTE_MEDIATED_CYTOTOXICITY	NIR	immune system	Myeloid lineage
GO_MYELOID_CELL_DIFFERENTIATION	NIR	immune system	Myeloid lineage
GO_MACROPHAGE_ACTIVATION	NIR	immune system	Myeloid lineage
GO_REGULATION_OF_LEUKOCYTE_MEDIATED_IMMUNITY	NIR	immune system	Myeloid lineage
GO_NEGATIVE_REGULATION_OF_LYMPHOCYTE_MEDIATED_IMMUNITY	NIR	immune system	Myeloid lineage
GO_MAST_CELL_GRANULE	NIR	immune system	Myeloid lineage
GO_DENDRITIC_CELL_DIFFERENTIATION	NIR	immune system	Myeloid lineage
GO_MYELOID_LEUKOCYTE_DIFFERENTIATION	NIR	immune system	Myeloid lineage
GO_MACROPHAGE_DIFFERENTIATION	NIR	immune system	Myeloid lineage
GO_LEUKOCYTE_ACTIVATION	NIR	immune system	Myeloid lineage
GO_POSITIVE_REGULATION_OF_LEUKOCYTE_PROLIFERATION	NIR	immune system	Myeloid lineage
GO_LYSOSOME_LOCALIZATION	NIR	immune system	Myeloid lineage
GO_NEGATIVE_REGULATION_OF_LEUKOCYTE_MEDIATED_IMMUNITY	NIR	immune system	Myeloid lineage
GO_LYMPHOCYTE_ACTIVATION_INVOLVED_IN_IMMUNE_RESPONSE	NIR	immune system	Myeloid lineage
GO_POSITIVE_REGULATION_OF_LEUKOCYTE_DEGRANULATION	NIR	immune system	Myeloid lineage
GO_REGULATION_OF_MAST_CELL_DEGRANULATION	NIR	immune system	Myeloid lineage
GO_POSITIVE_REGULATION_OF_LEUKOCYTE_DIFFERENTIATION	NIR	immune system	Myeloid lineage
GO_REGULATION_OF_LYMPHOCYTE_MEDIATED_IMMUNITY	NIR	immune system	Myeloid lineage
GO_GRANULOCYTE_ACTIVATION	NIR	immune system	Myeloid lineage
GO_GRANULOCYTE_DIFFERENTIATION	NIR	immune system	Myeloid lineage
GO_MYELOID_DENDRITIC_CELL_ACTIVATION	NIR	immune system	Myeloid lineage
GO_LEUKOCYTE_MEDIATED_IMMUNITY	NIR	immune system	Myeloid lineage
GO_REGULATION_OF_LEUKOCYTE_PROLIFERATION	NIR	immune system	Myeloid lineage
GO_TOLL_LIKE_RECEPTOR_SIGNALING_PATHWAY	NIR	immune system	signal transduction
GO_CHEMOKINE_RECEPTOR_BINDING	NIR	immune system	signal transduction
GO_POSITIVE_REGULATION_OF_TOLL_LIKE_RECEPTOR_SIGNALING_PATHWAY	NIR	immune system	signal transduction
GO_REGULATION_OF_T_CELL_RECEPTOR_SIGNALING_PATHWAY	NIR	immune system	signal transduction
GO_IMMUNE_RESPONSE_REGULATING_CELL_SURFACE_RECEPTOR_SIGNALING_PATHWAY	NIR	immune system	signal transduction
GO_POSITIVE_REGULATION_OF_ANTIGEN_PROCESSING_AND_PRESENTATION	NIR	immune system	signal transduction
GO_PATTERN_RECOGNITION_RECEPTOR_SIGNALING_PATHWAY	NIR	immune system	signal transduction
GO_CYTOKINE_BINDING	NIR	immune system	signal transduction
GO_DIVALENT_INORGANIC_CATION_HOMEOSTASIS	NIR	immune system	signal transduction
GO_MHC_PROTEIN_COMPLEX	NIR	immune system	signal transduction
GO_SIDE_OF_MEMBRANE	NIR	immune system	signal transduction
GO_CELL_CELL_RECOGNITION	NIR	immune system	signal transduction
GO_REGULATION_OF_ANTIGEN_RECEPTOR_MEDIATED_SIGNALING_PATHWAY	NIR	immune system	signal transduction
GO_REGULATION_OF_ANTIGEN_PROCESSING_AND_PRESENTATION	NIR	immune system	signal transduction
GO_IMMUNOLOGICAL_SYNAPSE	NIR	immune system	signal transduction
GO_TUMOR_NECROSIS_FACTOR_MEDIATED_SIGNALING_PATHWAY	NIR	immune system	signal transduction
GO_REGULATION_OF_ERK1_AND_ERK2_CASCADE	NIR	immune system	signal transduction
GO_REGULATION_OF_CYTOSOLIC_CALCIUM_ION_CONCENTRATION	NIR	immune system	signal transduction
GO_CYTOKINE_MEDIATED_SIGNALING_PATHWAY	NIR	immune system	signal transduction
GO_CLATHRIN_COATED_ENDOCYTIC_VESICLE	NIR	immune system	signal transduction
GO_LYMPHOCYTE_COSTIMULATION	NIR	immune system	signal transduction
GO_B_CELL_RECEPTOR_SIGNALING_PATHWAY	NIR	immune system	signal transduction
GO_MHC_CLASS_II_PROTEIN_COMPLEX_BINDING	NIR	immune system	signal transduction
GO_CYTOKINE_RECEPTOR_ACTIVITY	NIR	immune system	signal transduction
GO_CLATHRIN_COATED_ENDOCYTIC_VESICLE_MEMBRANE	NIR	immune system	signal transduction
GO_CHEMOKINE_MEDIATED_SIGNALING_PATHWAY	NIR	immune system	signal transduction
GO_CCR_CHEMOKINE_RECEPTOR_BINDING	NIR	immune system	signal transduction
GO_PHAGOCYTOSIS_RECOGNITION	NIR	immune system	signal transduction
GO_NEGATIVE_REGULATION_OF_ANTIGEN_RECEPTOR_MEDIATED_SIGNALING_PATHWAY	NIR	immune system	signal transduction
GO_CYTOKINE_RECEPTOR_BINDING	NIR	immune system	signal transduction
GO_PEPTIDYL_TYROSINE_AUTOPHOSPHORYLATION	NIR	immune system	signal transduction
GO_T_CELL_RECEPTOR_SIGNALING_PATHWAY	NIR	immune system	signal transduction
GO_MHC_PROTEIN_COMPLEX_BINDING	NIR	immune system	signal transduction
GO_INTERFERON_GAMMA_MEDIATED_SIGNALING_PATHWAY	NIR	immune system	signal transduction
GO_ANTIGEN_RECEPTOR_MEDIATED_SIGNALING_PATHWAY	NIR	immune system	signal transduction
GO_EXTERNAL_SIDE_OF_PLASMA_MEMBRANE	NIR	immune system	signal transduction
GO_POSITIVE_REGULATION_OF_ERK1_AND_ERK2_CASCADE	NIR	immune system	signal transduction
GO_ANTIGEN_BINDING	NIR	immune system	signal transduction
GO_MHC_CLASS_II_PROTEIN_COMPLEX	NIR	immune system	signal transduction
GO_PLASMA_MEMBRANE_RECEPTOR_COMPLEX	NIR	immune system	signal transduction
GO_CELL_RECOGNITION	NIR	immune system	signal transduction
GO_TUMOR_NECROSIS_FACTOR_RECEPTOR_BINDING	NIR	immune system	signal transduction
GO_CHEMOKINE_BINDING	NIR	immune system	signal transduction
GO_AROMATIC_AMINO_ACID_FAMILY_METABOLIC_PROCESS	NIR	metabolism	amino-acid
GO_AMINE_CATABOLIC_PROCESS	NIR	metabolism	amino-acid
GO_INDOLALKYLAMINE_METABOLIC_PROCESS	NIR	metabolism	amino-acid
GO_CELLULAR_MODIFIED_AMINO_ACID_METABOLIC_PROCESS	NIR	metabolism	amino-acid
GO_GLUTATHIONE_DERIVATIVE_METABOLIC_PROCESS	NIR	metabolism	amino-acid
GO_AROMATIC_AMINO_ACID_FAMILY_CATABOLIC_PROCESS	NIR	metabolism	amino-acid
GO_GLUTATHIONE_TRANSFERASE_ACTIVITY	NIR	metabolism	amino-acid
GO_CELLULAR_BIOGENIC_AMINE_CATABOLIC_PROCESS	NIR	metabolism	amino-acid
GO_AMINE_METABOLIC_PROCESS	NIR	metabolism	amino-acid
GO_GLUTATHIONE_DERIVATIVE_BIOSYNTHETIC_PROCESS	NIR	metabolism	amino-acid
GO_BENZENE_CONTAINING_COMPOUND_METABOLIC_PROCESS	NIR	metabolism	amino-acid
GO_SMALL_MOLECULE_BIOSYNTHETIC_PROCESS	NIR	metabolism	amino-acid
GO_ARACHIDONIC_ACID_METABOLIC_PROCESS	NIR	metabolism	amino-acid
GO_CELLULAR_AMINO_ACID_BIOSYNTHETIC_PROCESS	NIR	metabolism	amino-acid
)_REGULATION_OF_INTRINSIC_APOPTOTIC_SIGNALING_PATHWAY_IN_RESPONSE_TO_DNA_DAMA	NIR	metabolism	apoptosis
GO_POSITIVE_REGULATION_OF_INTRINSIC_APOPTOTIC_SIGNALING_PATHWAY	NIR	metabolism	apoptosis
GATIVE_REGULATION_OF_INTRINSIC_APOPTOTIC_SIGNALING_PATHWAY_IN_RESPONSE_TO_DNA_[	NIR	metabolism	apoptosis
GO_REGULATION_OF_INTRINSIC_APOPTOTIC_SIGNALING_PATHWAY	NIR	metabolism	apoptosis

GO_REGULATION_OF_PHAGOCYTOSIS	NIR	metabolism	cytosis
GO_POSITIVE_REGULATION_OF_ENDOCYTOSIS	NIR	metabolism	cytosis
GO_POSITIVE_REGULATION_OF_PHAGOCYTOSIS	NIR	metabolism	cytosis
GO_MEMBRANE_INVAGINATION	NIR	metabolism	cytosis
GO_PHAGOCYTOSIS_ENGULFMENT	NIR	metabolism	cytosis
GO_ACTIVATION_OF_CYSTEINE_TYPE_ENDOPEPTIDASE_ACTIVITY	NIR	metabolism	decomposition
GO_METALLOEXOPEPTIDASE_ACTIVITY	NIR	metabolism	decomposition
GO_SERINE_TYPE_EXOPEPTIDASE_ACTIVITY	NIR	metabolism	decomposition
GO_POSITIVE_REGULATION_OF_PEPTIDASE_ACTIVITY	NIR	metabolism	decomposition
_REGULATION_OF_CYSTEINE_TYPE_ENDOPEPTIDASE_ACTIVITY_INVOLVED_IN_APOPTOTIC_SIGNALI	NIR	metabolism	decomposition
GO_CARBOXYPEPTIDASE_ACTIVITY	NIR	metabolism	decomposition
GO_NEGATIVE_REGULATION_OF_PROTEOLYSIS	NIR	metabolism	decomposition
;ATIVE_REGULATION_OF_EXTRINSIC_APOPTOTIC_SIGNALING_PATHWAY_VIA_DEATH_DOMAIN_RE(	NIR	metabolism	decomposition
GO_PEPTIDASE_INHIBITOR_ACTIVITY	NIR	metabolism	decomposition
GO_PEPTIDASE_ACTIVATOR_ACTIVITY_INVOLVED_IN_APOPTOTIC_PROCESS	NIR	metabolism	decomposition
GO_CALCIUM_DEPENDENT_CYSTEINE_TYPE_ENDOPEPTIDASE_ACTIVITY	NIR	metabolism	decomposition
ULATION_OF_CYSTEINE_TYPE_ENDOPEPTIDASE_ACTIVITY_INVOLVED_IN_APOPTOTIC_SIGNALING_P	NIR	metabolism	decomposition
GO_CYSTEINE_TYPE_ENDOPEPTIDASE_INHIBITOR_ACTIVITY	NIR	metabolism	decomposition
GO_EXOPEPTIDASE_ACTIVITY	NIR	metabolism	decomposition
GO_CYSTEINE_TYPE_ENDOPEPTIDASE_ACTIVITY	NIR	metabolism	decomposition
GO_REGULATION_OF_PEPTIDASE_ACTIVITY	NIR	metabolism	decomposition
GO_NEGATIVE_REGULATION_OF_HYDROLASE_ACTIVITY	NIR	metabolism	decomposition
GO_PEPTIDASE_REGULATOR_ACTIVITY	NIR	metabolism	decomposition
GO_METALLOCARBOXYPEPTIDASE_ACTIVITY	NIR	metabolism	decomposition
GO_CYSTEINE_TYPE_ENDOPEPTIDASE_INHIBITOR_ACTIVITY_INVOLVED_IN_APOPTOTIC_PROCESS	NIR	metabolism	decomposition
GO_SERINE_TYPE_ENDOPEPTIDASE_INHIBITOR_ACTIVITY	NIR	metabolism	decomposition
GO_CYSTEINE_TYPE_ENDOPEPTIDASE_REGULATOR_ACTIVITY_INVOLVED_IN_APOPTOTIC_PROCESS	NIR	metabolism	decomposition
GO_ENDOPEPTIDASE_ACTIVITY	NIR	metabolism	decomposition
GO_PROTEIN_MATURATION	NIR	metabolism	decomposition
GO_PEPTIDASE_ACTIVATOR_ACTIVITY	NIR	metabolism	decomposition
GO_ZYMOGEN_ACTIVATION	NIR	metabolism	decomposition
GO_SERINE_HYDROLASE_ACTIVITY	NIR	metabolism	decomposition
GO_NEGATIVE_REGULATION_OF_PEPTIDASE_ACTIVITY	NIR	metabolism	decomposition
GO_CARBON_CARBON_LYASE_ACTIVITY	NIR	metabolism	decomposition
GO_HYDRO_LYASE_ACTIVITY	NIR	metabolism	decomposition
GO_NUCLEOSIDE_BISPHOSPHATE_METABOLIC_PROCESS	NIR	metabolism	decomposition
GO_HYDROLASE_ACTIVITY_ACTING_ON_CARBON_NITROGEN_BUT_NOT_PEPTIDE_BONDS	NIR	metabolism	decomposition
GO_RIBONUCLEOSIDE_BISPHOSPHATE_METABOLIC_PROCESS	NIR	metabolism	decomposition
GO_PURINE_RIBONUCLEOSIDE_BISPHOSPHATE_METABOLIC_PROCESS	NIR	metabolism	decomposition
GO_PURINE_NUCLEOSIDE_BISPHOSPHATE_METABOLIC_PROCESS	NIR	metabolism	decomposition
GO_LYASE_ACTIVITY	NIR	metabolism	decomposition
IYDROLASE_ACTIVITY_ACTING_ON_CARBON_NITROGEN_BUT_NOT_PEPTIDE_BONDS_IN_LINEAR_AI	NIR	metabolism	decomposition
GO_DEAMINASE_ACTIVITY	NIR	metabolism	decomposition
GO_PYRIDOXAL_PHOSPHATE_BINDING	NIR	metabolism	decomposition
'DROLASE_ACTIVITY_ACTING_ON_CARBON_NITROGEN_BUT_NOT_PEPTIDE_BONDS_IN_CYCLIC_AM	NIR	metabolism	decomposition
GO_PIR_CHAIN_FATTY_ACID_METABOLIC_PROCESS	NIR	metabolism	fatty-acid
GO_LIGASE_ACTIVITY_FORMING_CARBON_SULFUR_BONDS	NIR	metabolism	fatty-acid
GO_FATTY_ACID_BIOSYNTHETIC_PROCESS	NIR	metabolism	fatty-acid
GO_PROSTAGLANDIN_METABOLIC_PROCESS	NIR	metabolism	fatty-acid
GO_PROSTAGLANDIN_BIOSYNTHETIC_PROCESS	NIR	metabolism	fatty-acid
GO_ICOSANOID_BIOSYNTHETIC_PROCESS	NIR	metabolism	fatty-acid
GO_FATTY_ACID_DERIVATIVE_BIOSYNTHETIC_PROCESS	NIR	metabolism	fatty-acid
GO_LEUKOTRIENE_METABOLIC_PROCESS	NIR	metabolism	fatty-acid
GO_FATTY_ACID_LIGASE_ACTIVITY	NIR	metabolism	fatty-acid
GO_UNSATURATED_FATTY_ACID_METABOLIC_PROCESS	NIR	metabolism	fatty-acid
GO_ICOSANOID_METABOLIC_PROCESS	NIR	metabolism	fatty-acid
GO_ACID_THIOL_LIGASE_ACTIVITY	NIR	metabolism	fatty-acid
GO_PROSTANOID_METABOLIC_PROCESS	NIR	metabolism	fatty-acid
GO_CARBOXYLIC_ACID_BIOSYNTHETIC_PROCESS	NIR	metabolism	fatty-acid
GO_ORGANIC_ACID_BIOSYNTHETIC_PROCESS	NIR	metabolism	fatty-acid
GO_FATTY_ACID_DERIVATIVE_METABOLIC_PROCESS	NIR	metabolism	fatty-acid
GO_LEUKOTRIENE_BIOSYNTHETIC_PROCESS	NIR	metabolism	fatty-acid
GO_PROSTANOID_BIOSYNTHETIC_PROCESS	NIR	metabolism	fatty-acid
GO_UNSATURATED_FATTY_ACID_BIOSYNTHETIC_PROCESS	NIR	metabolism	fatty-acid
GO_DNA_DEALKYLATION	PIR	metabolism	organonitrogen
GO_DNA_DEMETHYLATION	PIR	metabolism	organonitrogen
GO_ORGANONITROGEN_COMPOUND_CATABOLIC_PROCESS	NIR	metabolism	organonitrogen
GO_CELLULAR_METABOLIC_COMPOUND_SALVAGE	NIR	metabolism	organonitrogen
GO_GLYCOSYL_COMPOUND_CATABOLIC_PROCESS	NIR	metabolism	organonitrogen
GO_COFACTOR_CATABOLIC_PROCESS	NIR	metabolism	organonitrogen
GO_NUCLEOSIDE_PHOSPHATE_CATABOLIC_PROCESS	NIR	metabolism	organonitrogen
GO_CARBOHYDRATE_DERIVATIVE_CATABOLIC_PROCESS	NIR	metabolism	organonitrogen
GO_PURINE_CONTAINING_COMPOUND_SALVAGE	NIR	metabolism	organonitrogen
GO_PURINE_CONTAINING_COMPOUND_CATABOLIC_PROCESS	NIR	metabolism	organonitrogen
GO_RIBONUCLEOSIDE_CATABOLIC_PROCESS	NIR	metabolism	organonitrogen
GO_POSITIVE_REGULATION_OF_REACTIVE_OXYGEN_SPECIES_METABOLIC_PROCESS	NIR	metabolism	oxidoreduction
GO_MONOOXYGENASE_ACTIVITY	NIR	metabolism	oxidoreduction
GO_IRON_ION_BINDING	NIR	metabolism	oxidoreduction
GO_REGULATION_OF_RESPONSE_TO_OXIDATIVE_STRESS	NIR	metabolism	oxidoreduction
GO_REGULATION_OF_REACTIVE_OXYGEN_SPECIES_METABOLIC_PROCESS	NIR	metabolism	oxidoreduction
NORS_WITH_INCORPORATION_OR_REDUCTION_OF_MOLECULAR_OXYGEN_NAD_P_H_AS_ONE_DC	NIR	metabolism	oxidoreduction
GO_HYDROGEN_PEROXIDE_METABOLIC_PROCESS	NIR	metabolism	oxidoreduction
GO_TETRAPYRROLE_BINDING	NIR	metabolism	oxidoreduction
GO_OXIDOREDUCTASE_ACTIVITY_ACTING_ON_PEROXIDE_AS_ACCEPTOR	NIR	metabolism	oxidoreduction
GO_REGULATION_OF_NITRIC_OXIDE_BIOSYNTHETIC_PROCESS	NIR	metabolism	oxidoreduction
GO_RESPONSE_TO_HYDROPEROXIDE	NIR	metabolism	oxidoreduction
)OREDUCTASE_ACTIVITY_ACTING_ON_SINGLE_DONORS_WITH_INCORPORATION_OF_MOLECULAR_	NIR	metabolism	oxidoreduction

GO_ANTIOXIDANT_ACTIVITY	NIR	metabolism	oxidoreduction
GO_TERPENOID_METABOLIC_PROCESS	NIR	metabolism	sterols/alcohols
GO_ALCOHOL_DEHYDROGENASE_NADP_ACTIVITY	NIR	metabolism	sterols/alcohols
GO_ORGANIC_HYDROXY_COMPOUND_TRANSPORT	NIR	metabolism	sterols/alcohols
GO_PHOSPHATIDYLCHOLINE_METABOLIC_PROCESS	NIR	metabolism	sterols/alcohols
GO_REGULATION_OF_STEROID_METABOLIC_PROCESS	NIR	metabolism	sterols/alcohols
GO_REGULATION_OF_HORMONE_LEVELS	NIR	metabolism	sterols/alcohols
GO_ALDO_KETO_REDUCTASE_NADP_ACTIVITY	NIR	metabolism	sterols/alcohols
GO_REGULATION_OF_ALCOHOL_BIOSYNTHETIC_PROCESS	NIR	metabolism	sterols/alcohols
GO_PRIMARY_ALCOHOL_METABOLIC_PROCESS	NIR	metabolism	sterols/alcohols
GO_HORMONE_METABOLIC_PROCESS	NIR	metabolism	sterols/alcohols
GO_ALCOHOL_BINDING	NIR	metabolism	sterols/alcohols
GO_POSITIVE_REGULATION_OF_FATTY_ACID_BIOSYNTHETIC_PROCESS	NIR	metabolism	sterols/alcohols
GO_DIGESTIVE_SYSTEM_PROCESS	NIR	metabolism	sterols/alcohols
GO_REGULATION_OF_STEROID_BIOSYNTHETIC_PROCESS	NIR	metabolism	sterols/alcohols
GO_PHOSPHOLIPASE_C_ACTIVITY	NIR	metabolism	sterols/alcohols
GO_REGULATION_OF_LIPID_BIOSYNTHETIC_PROCESS	NIR	metabolism	sterols/alcohols
GO_LIPID_DIGESTION	NIR	metabolism	sterols/alcohols
GO_VITAMIN_METABOLIC_PROCESS	NIR	metabolism	sterols/alcohols
GO_CELLULAR_HORMONE_METABOLIC_PROCESS	NIR	metabolism	sterols/alcohols
GO_ALCOHOL_BIOSYNTHETIC_PROCESS	NIR	metabolism	sterols/alcohols
GO_ORGANIC_HYDROXY_COMPOUND_TRANSMEMBRANE_TRANSPORTER_ACTIVITY	NIR	metabolism	sterols/alcohols
GO_RETINOL_DEHYDROGENASE_ACTIVITY	NIR	metabolism	sterols/alcohols
GO_ETHANOLAMINE_CONTAINING_COMPOUND_METABOLIC_PROCESS	NIR	metabolism	sterols/alcohols
GO_ISOPRENOID_METABOLIC_PROCESS	NIR	metabolism	sterols/alcohols
GO_FAT_SOLUBLE_VITAMIN_METABOLIC_PROCESS	NIR	metabolism	sterols/alcohols
GO_SECONDARY_METABOLIC_PROCESS	NIR	metabolism	sterols/alcohols
GO_REGULATION_OF_PLASMA_LIPOPROTEIN_PARTICLE_LEVELS	NIR	metabolism	sterols/alcohols
GO_AZOLE_TRANSPORT	NIR	metabolism	sterols/alcohols
GO_PHOSPHOLIPASE_A2_ACTIVITY	NIR	metabolism	sterols/alcohols
GO_PHOSPHORIC_DIESTER_HYDROLASE_ACTIVITY	NIR	metabolism	sterols/alcohols
GO_STEROID_BIOSYNTHETIC_PROCESS	NIR	metabolism	sterols/alcohols
GO_C21_STEROID_HORMONE_METABOLIC_PROCESS	NIR	metabolism	sterols/alcohols
GO_STEROID_DEHYDROGENASE_ACTIVITY	NIR	metabolism	sterols/alcohols
GO_ALCOHOL_METABOLIC_PROCESS	NIR	metabolism	sterols/alcohols
GO_POSITIVE_REGULATION_OF_LIPID_BIOSYNTHETIC_PROCESS	NIR	metabolism	sterols/alcohols
GO_OXIDOREDUCTASE_ACTIVITY_ACTING_ON_CH_OH_GROUP_OF_DONORS	NIR	metabolism	sterols/alcohols
GO_ORGANIC_HYDROXY_COMPOUND_BIOSYNTHETIC_PROCESS	NIR	metabolism	sterols/alcohols
GO_CARBOXYLIC_ESTER_HYDROLASE_ACTIVITY	NIR	metabolism	sterols/alcohols
GO_MONOAMINE_TRANSPORT	NIR	metabolism	sterols/alcohols
GO_STEROL_TRANSPORT	NIR	metabolism	sterols/alcohols
GO_HIGH_DENSITY_LIPOPROTEIN_PARTICLE_REMODELING	NIR	metabolism	sterols/alcohols
GO_DIGESTION	NIR	metabolism	sterols/alcohols
GO_GLYCOSIDE_METABOLIC_PROCESS	NIR	metabolism	sterols/alcohols
GO_PHOSPHOLIPASE_ACTIVITY	NIR	metabolism	sterols/alcohols
GO_STEROID_BINDING	NIR	metabolism	sterols/alcohols
GO_REGULATION_OF_CHOLESTEROL_METABOLIC_PROCESS	NIR	metabolism	sterols/alcohols
GO_AMMONIUM_TRANSPORT	NIR	metabolism	sterols/alcohols
GO_LIPID_LOCALIZATION	NIR	metabolism	sterols/alcohols
GO_ORGANIC_HYDROXY_COMPOUND_METABOLIC_PROCESS	NIR	metabolism	sterols/alcohols
GO_POSITIVE_REGULATION_OF_STEROID_METABOLIC_PROCESS	NIR	metabolism	sterols/alcohols
GO_LIPASE_ACTIVITY	NIR	metabolism	sterols/alcohols
GO_STEROID_METABOLIC_PROCESS	NIR	metabolism	sterols/alcohols
GO_NEGATIVE_REGULATION_OF_LIPID_METABOLIC_PROCESS	NIR	metabolism	sterols/alcohols
GO_PROTEIN_LIPID_COMPLEX_SUBUNIT_ORGANIZATION	NIR	metabolism	sterols/alcohols
GO_RETINOL_METABOLIC_PROCESS	NIR	metabolism	sterols/alcohols
GO_RESPONSE_TO_TOXIC_SUBSTANCE	NIR	metabolism	toxicity
GO_PROTEIN_DNA_COMPLEX	PIR	Nucleus activity	
GO_NON_RECOMBINATIONAL_REPAIR	PIR	Nucleus activity	
GO_NEGATIVE_REGULATION_OF_HEMATOPOIETIC_PROGENITOR_CELL_DIFFERENTIATION	PIR	Nucleus activity	
GO_BETA_CATENIN_TCF_COMPLEX_ASSEMBLY	PIR	Nucleus activity	
GO_GENE_SILENCING_BY_RNA	PIR	Nucleus activity	
GO_DNA_PACKAGING	PIR	Nucleus activity	
GO_CHROMATIN_SILENCING_AT_RDNA	PIR	Nucleus activity	
GO_CHROMATIN_ASSEMBLY_OR_DISASSEMBLY	PIR	Nucleus activity	
GO_NUCLEAR_CHROMOSOME_TELOMERIC_REGION	PIR	Nucleus activity	
GO_TELOMERE_CAPPING	PIR	Nucleus activity	
GO_PROTEIN_HETEROTETRAMERIZATION	PIR	Nucleus activity	
GO_REGULATION_OF_HEMATOPOIETIC_PROGENITOR_CELL_DIFFERENTIATION	PIR	Nucleus activity	
GO_REGULATION_OF_GENE_EXPRESSION_EPIGENETIC	PIR	Nucleus activity	
GO_POSITIVE_REGULATION_OF_GENE_EXPRESSION_EPIGENETIC	PIR	Nucleus activity	
GO_NEGATIVE_REGULATION_OF_GENE_EXPRESSION_EPIGENETIC	PIR	Nucleus activity	
GO_DNA_PACKAGING_COMPLEX	PIR	Nucleus activity	
GO_DNA_REPLICATION_DEPENDENT_NUCLEOSOME_ASSEMBLY	PIR	Nucleus activity	
GO_TELOMERE_ORGANIZATION	PIR	Nucleus activity	
GO_REGULATION_OF_GENE_SILENCING	PIR	Nucleus activity	
GO_GENE_SILENCING	PIR	Nucleus activity	
GO_DNA_REPLICATION_DEPENDENT_NUCLEOSOME_ORGANIZATION	PIR	Nucleus activity	
GO_PYRIDINE_CONTAINING_COMPOUND_BIOSYNTHETIC_PROCESS	NIR	Nucleus activity	
GO_NICOTINAMIDE_NUCLEOTIDE_BIOSYNTHETIC_PROCESS	NIR	Nucleus activity	
GO_PYRIDINE_NUCLEOTIDE_BIOSYNTHETIC_PROCESS	NIR	Nucleus activity	
GO_POSITIVE_REGULATION_OF_EPIDERMIS_DEVELOPMENT	NIR	organogenesis	epidermis development
GO_REGULATION_OF_HAIR_FOLLICLE_DEVELOPMENT	NIR	organogenesis	epidermis development
GO_MUSCULOSKELETAL_MOVEMENT	NIR	physiological function	muslce movement
GO_SKELETAL_MUSCLE_CONTRACTION	NIR	physiological function	muslce movement
GO_MULTICELLULAR_ORGANISMAL_MOVEMENT	NIR	physiological function	muslce movement
GO_MUSCLE_FILAMENT_SLIDING	NIR	physiological function	muslce movement

GO_ACTIN_MYOSIN_FILAMENT_SLIDING	NIR	physiological function	muslce movement
GO_STRIATED_MUSCLE_CONTRACTION	NIR	physiological function	muslce movement
GO_ACTIN_FILAMENT_POLYMERIZATION	NIR	organogenesis	muscle development
GO_ACTIN_POLYMERIZATION_OR_DEPOLYMERIZATION	NIR	organogenesis	muscle development
GO_CEREBRAL_CORTEX_RADIAL_GLIA_GUIDED_MIGRATION	PIR	organogenesis	neural development
GO_CEREBRAL_CORTEX_RADIALLY_ORIENTED_CELL_MIGRATION	PIR	organogenesis	neural development
GO_TELENCEPHALON_GLIAL_CELL_MIGRATION	PIR	organogenesis	neural development
GO_ORGAN_REGENERATION	NIR	organogenesis	organ regeneration
GO_REGENERATION	NIR	organogenesis	organ regeneration
GO_LIVER_REGENERATION	NIR	organogenesis	organ regeneration
GO_TISSUE_REGENERATION	NIR	organogenesis	organ regeneration
GO_PLATELET_ALPHA_GRANULE_LUMEN	NIR	organogenesis	Secretory granules
GO_PLATELET_DEGRANULATION	NIR	organogenesis	Secretory granules
GO_PRIMARY_LYSOSOME	NIR	organogenesis	Secretory granules
GO_SECRETORY_GRANULE	NIR	organogenesis	Secretory granules
GO_VESICLE_LUMEN	NIR	organogenesis	Secretory granules
GO_SECRETORY_GRANULE_LUMEN	NIR	organogenesis	Secretory granules
GO_PLATELET_ALPHA_GRANULE	NIR	organogenesis	Secretory granules
GO_SECRETORY_GRANULE_MEMBRANE	NIR	organogenesis	Secretory granules
GO_MAINTENANCE_OF_GASTROINTESTINAL_EPITHELIUM	NIR	organogenesis	Tissue homeostasis
GO_EPITHELIAL_STRUCTURE_MAINTENANCE	NIR	organogenesis	Tissue homeostasis
GO_TISSUE_HOMEOSTASIS	NIR	organogenesis	Tissue homeostasis
GO_RETINA_HOMEOSTASIS	NIR	organogenesis	Tissue homeostasis
GO_MULTICELLULAR_ORGANISMAL_HOMEOSTASIS	NIR	organogenesis	Tissue homeostasis
GO_CELL_MATURATION	NIR	physiological function	fertility
GO_SPERM_CAPACITATION	NIR	physiological function	fertility
GO_SPERM_PART	NIR	physiological function	fertility
GO_NEGATIVE_REGULATION_OF_STAT_CASCADE	PIR	signal transduction	
GO_NEGATIVE_REGULATION_OF_JAK_STAT_CASCADE	PIR	signal transduction	
GO_POSITIVE_REGULATION_OF_TYROSINE_PHOSPHORYLATION_OF_STAT3_PROTEIN	NIR	signal transduction	
GO_POSITIVE_REGULATION_OF_STAT_CASCADE	NIR	signal transduction	
GO_POSITIVE_REGULATION_OF_JAK_STAT_CASCADE	NIR	signal transduction	
GO_REGULATION_OF_PEPTIDYL_TYROSINE_PHOSPHORYLATION	NIR	signal transduction	
GO_REGULATION_OF_TYROSINE_PHOSPHORYLATION_OF_STAT_PROTEIN	NIR	signal transduction	
GO_POSITIVE_REGULATION_OF_PEPTIDYL_TYROSINE_PHOSPHORYLATION	NIR	signal transduction	
GO_POSITIVE_REGULATION_OF_SODIUM_ION_TRANSMEMBRANE_TRANSPORT	NIR	transport	
GO_POSITIVE_REGULATION_OF_TRANSMEMBRANE_TRANSPORT	NIR	transport	
GO_POSITIVE_REGULATION_OF_CALCIUM_MEDIATED_SIGNALING	NIR	transport	
GO_POSITIVE_REGULATION_OF_CALCIUM_ION_TRANSPORT	NIR	transport	
GO_POSITIVE_REGULATION_OF_CATION_TRANSMEMBRANE_TRANSPORT	NIR	transport	
GO_REGULATION_OF_SYNAPTIC_TRANSMISSION_DOPAMINERGIC	NIR	transport	
GO_POSITIVE_REGULATION_OF_ION_TRANSPORT	NIR	transport	
GO_POSITIVE_REGULATION_OF_CALCIUM_ION_TRANSMEMBRANE_TRANSPORT	NIR	transport	
GO_POSITIVE_REGULATION_OF_CALCIUM_ION_TRANSPORT_INTO_CYTOSOL	NIR	transport	
GO_BASOLATERAL_PLASMA_MEMBRANE	NIR	transport	
GO_POSITIVE_REGULATION_OF_SODIUM_ION_TRANSPORT	NIR	transport	
GO_POSITIVE_REGULATION_OF_TRANSPORTER_ACTIVITY	NIR	transport	
GO_REGULATION_OF_CALCIUM_MEDIATED_SIGNALING	NIR	transport	
GO_CHLORIDE_CHANNEL_REGULATOR_ACTIVITY	NIR	transport	
GO_BICARBONATE_TRANSMEMBRANE_TRANSPORTER_ACTIVITY	NIR	transport	
GO_BASAL_PLASMA_MEMBRANE	NIR	transport	
GO_REGULATION_OF_CALCIUM_ION_TRANSPORT	NIR	transport	
GO_POSITIVE_REGULATION_OF_LIPID_TRANSPORT	NIR	transport	
GO_CALCIUM_MEDIATED_SIGNALING	NIR	transport	
GO_CALCIUM_MEDIATED_SIGNALING_USING_INTRACELLULAR_CALCIUM_SOURCE	NIR	transport	

Supplementary Data 2B. The grouping of GO terms in Cohort 2.

GO_term	Enrichment in	Type 1	Type 2	Type 3	Type 4
GO_CELLULAR_RESPONSE_TO_GLUCAGON_STIMULUS	PIR	Cellular response	hormone	glucagon	
GO_RESPONSE_TO_GLUCAGON	PIR	Cellular response	hormone	glucagon	
GO_CELLULAR_RESPONSE_TO_PROSTAGLANDIN_E_STIMULUS	NIR	Cellular response	hormone		
GO_RESPONSE_TO_PROSTAGLANDIN	NIR	Cellular response	hormone		
GO_RESPONSE_TO_PROSTAGLANDIN_E	NIR	Cellular response	hormone		
GO_CELLULAR_RESPONSE_TO_CALCIUM_ION	PIR	Cellular response	metal ion		
GO_CELLULAR_RESPONSE_TO_INORGANIC_SUBSTANCE	PIR	Cellular response	metal ion		
GO_RESPONSE_TO_CALCIUM_ION	PIR	Cellular response	metal ion		
GO_RESPONSE_TO_MANGANESE_ION	PIR	Cellular response	metal ion		
GO_RESPONSE_TO_METAL_ION	PIR	Cellular response	metal ion		
GO_CELLULAR_RESPONSE_TO_NUTRIENT	NIR	Cellular response	nutrient		
GO_CELLULAR_RESPONSE_TO_VITAMIN	NIR	Cellular response	nutrient		
GO_CELLULAR_RESPONSE_TO_RADIATION	NIR	Cellular response	radiation		
GO_CELLULAR_RESPONSE_TO_IONIZING_RADIATION	NIR	Cellular response	radiation		
GO_RESPONSE_TO_GAMMA_RADIATION	NIR	Cellular response	radiation		
GO_RESPONSE_TO_IONIZING_RADIATION	NIR	Cellular response	radiation		
GO_RESPONSE_TO_UV	NIR	Cellular response	radiation		
GO_RESPONSE_TO_X_RAY	NIR	Cellular response	radiation		
GO_FEAR_RESPONSE	PIR	Cellular response	stress		
GO_MULTICELLULAR_ORGANISMAL_RESPONSE_TO_STRESS	PIR	Cellular response	stress		
GO_LEUKOCYTE_CELL_CELL_ADHESION	NIR	immune system	cell adhesion		
GO_NEGATIVE_REGULATION_OF_CELL_ADHESION	NIR	immune system	cell adhesion		
GO_NEGATIVE_REGULATION_OF_CELL_CELL_ADHESION	NIR	immune system	cell adhesion		
GO_NEGATIVE_REGULATION_OF_HOMOTYPIC_CELL_CELL_ADHESION	NIR	immune system	cell adhesion		
GO_POSITIVE_REGULATION_OF_CELL_ADHESION	NIR	immune system	cell adhesion		
GO_POSITIVE_REGULATION_OF_CELL_CELL_ADHESION	NIR	immune system	cell adhesion		
GO_REGULATION_OF_CELL_CELL_ADHESION	NIR	immune system	cell adhesion		
GO_REGULATION_OF_HOMOTYPIC_CELL_CELL_ADHESION	NIR	immune system	cell adhesion		
GO_LYMPHOCYTE_APOPTOTIC_PROCESS	NIR	immune system	cell death	apoptosis	
GO_NEGATIVE_REGULATION_OF_LEUKOCYTE_APOPTOTIC_PROCESS	NIR	immune system	cell death	apoptosis	
GO_NEGATIVE_REGULATION_OF_LYMPHOCYTE_APOPTOTIC_PROCESS	NIR	immune system	cell death	apoptosis	
GO_NEGATIVE_REGULATION_OF_T_CELL_APOPTOTIC_PROCESS	NIR	immune system	cell death	apoptosis	
GO_POSITIVE_REGULATION_OF_LYMPHOCYTE_APOPTOTIC_PROCESS	NIR	immune system	cell death	apoptosis	
GO_REGULATION_OF_B_CELL_APOPTOTIC_PROCESS	NIR	immune system	cell death	apoptosis	
GO_REGULATION_OF_LEUKOCYTE_APOPTOTIC_PROCESS	NIR	immune system	cell death	apoptosis	
GO_REGULATION_OF_LYMPHOCYTE_APOPTOTIC_PROCESS	NIR	immune system	cell death	apoptosis	
GO_REGULATION_OF_T_CELL_APOPTOTIC_PROCESS	NIR	immune system	cell death	apoptosis	
GO_NEGATIVE_REGULATION_OF_CELL_KILLING	NIR	immune system	cell death	cell kill	
GO_REGULATION_OF_CELL_KILLING	NIR	immune system	cell death	cell kill	
GO_LEUKOCYTE_HOMEOSTASIS	NIR	immune system	cell death	HEMOPOIESIS	
GO_LYMPHOCYTE_HOMEOSTASIS	NIR	immune system	cell death	HEMOPOIESIS	
GO_NEGATIVE_REGULATION_OF_HEMOPOIESIS	NIR	immune system	cell death	HEMOPOIESIS	
GO_REGULATION_OF_HEMOPOIESIS	NIR	immune system	cell death	HEMOPOIESIS	
GO_DENDRITIC_CELL_CHEMOTAXIS	NIR	immune system	cell migration		
GO_DENDRITIC_CELL_MIGRATION	NIR	immune system	cell migration		
GO_POSITIVE_REGULATION_OF_CELL_ADHESION_MEDIATED_BY_INTEGRIN	NIR	immune system	cell migration		
GO_REGULATION_OF_CELLULAR_EXTRAVASATION	NIR	immune system	cell migration		
GO_CYTOKINE_PRODUCTION	NIR	immune system	cytokine		
GO_CYTOKINE_SECRETION	NIR	immune system	cytokine		
GO_LYMPHOCYTE_COSTIMULATION	NIR	immune system	cytokine		
GO_NEGATIVE_REGULATION_OF_CYTOKINE_PRODUCTION	NIR	immune system	cytokine		
GO_NEGATIVE_REGULATION_OF_INTERFERON_GAMMA_PRODUCTION	NIR	immune system	cytokine		
GO_NEGATIVE_REGULATION_OF_INTERLEUKIN_12_PRODUCTION	NIR	immune system	cytokine		
GATIVE_REGULATION_OF_TUMOR_NECROSIS_FACTOR_SUPERFAMILY_CYTOKINE_PRODU	NIR	immune system	cytokine		
GO_NEGATIVE_REGULATION_OF_TYPE_I_INTERFERON_PRODUCTION	NIR	immune system	cytokine		
GO_POSITIVE_REGULATION_OF_CYTOKINE_BIOSYNTHETIC_PROCESS	NIR	immune system	cytokine		
GO_POSITIVE_REGULATION_OF_INTERFERON_ALPHA_PRODUCTION	NIR	immune system	cytokine		
GO_POSITIVE_REGULATION_OF_INTERFERON_BETA_PRODUCTION	NIR	immune system	cytokine		
GO_POSITIVE_REGULATION_OF_INTERFERON_GAMMA_PRODUCTION	NIR	immune system	cytokine		
GO_POSITIVE_REGULATION_OF_INTERLEUKIN_1_BETA_PRODUCTION	NIR	immune system	cytokine		
GO_POSITIVE_REGULATION_OF_INTERLEUKIN_1_PRODUCTION	NIR	immune system	cytokine		
GO_POSITIVE_REGULATION_OF_INTERLEUKIN_12_PRODUCTION	NIR	immune system	cytokine		
OSITIVE_REGULATION_OF_TUMOR_NECROSIS_FACTOR_SUPERFAMILY_CYTOKINE_PRODU	NIR	immune system	cytokine		
GO_POSITIVE_REGULATION_OF_TYPE_I_INTERFERON_PRODUCTION	NIR	immune system	cytokine		
GO_REGULATION_OF_CYTOKINE_BIOSYNTHETIC_PROCESS	NIR	immune system	cytokine		
GO_REGULATION_OF_INTERFERON_ALPHA_PRODUCTION	NIR	immune system	cytokine		
GO_REGULATION_OF_INTERFERON_BETA_PRODUCTION	NIR	immune system	cytokine		
GO_REGULATION_OF_INTERFERON_GAMMA_PRODUCTION	NIR	immune system	cytokine		
GO_REGULATION_OF_INTERLEUKIN_1_BETA_PRODUCTION	NIR	immune system	cytokine		
GO_REGULATION_OF_INTERLEUKIN_1_PRODUCTION	NIR	immune system	cytokine		
GO_REGULATION_OF_INTERLEUKIN_1_SECRETION	NIR	immune system	cytokine		
GO_REGULATION_OF_INTERLEUKIN_10_PRODUCTION	NIR	immune system	cytokine		
GO_REGULATION_OF_INTERLEUKIN_12_PRODUCTION	NIR	immune system	cytokine		
GO_REGULATION_OF_INTERLEUKIN_2_BIOSYNTHETIC_PROCESS	NIR	immune system	cytokine		
GO_REGULATION_OF_INTERLEUKIN_8_SECRETION	NIR	immune system	cytokine		
O_REGULATION_OF_TUMOR_NECROSIS_FACTOR_SUPERFAMILY_CYTOKINE_PRODUCTIO	NIR	immune system	cytokine		
GO_REGULATION_OF_TYPE_I_INTERFERON_PRODUCTION	NIR	immune system	cytokine		
GO_MHC_PROTEIN_BINDING	NIR	immune system	cytokine		
GO_MHC_CLASS_I_PROTEIN_BINDING	NIR	immune system	cytokine		
ATIVE_REGULATION_OF_SEQUENCE_SPECIFIC_DNA_BINDING_TRANSCRIPTION_FACTOR_	NIR	immune system	cytokine		
GO_NEGATIVE_REGULATION_OF_NF_KAPPAB_TRANSCRIPTION_FACTOR_ACTIVITY	NIR	immune system	cytokine		
GO_DEFENSE_RESPONSE_TO_OTHER_ORGANISM	NIR	immune system	defense response		
GO_DEFENSE_RESPONSE_TO_VIRUS	NIR	immune system	defense response		
GO_DETECTION_OF_BIOTIC_STIMULUS	NIR	immune system	defense response		
GO_NEGATIVE_REGULATION_OF_DEFENSE_RESPONSE	NIR	immune system	defense response		
GO_NEGATIVE_REGULATION_OF_DEFENSE_RESPONSE_TO_VIRUS	NIR	immune system	defense response		
GO_NEGATIVE_REGULATION_OF_MULTI_ORGANISM_PROCESS	NIR	immune system	defense response		
GO_NEGATIVE_REGULATION_OF_RESPONSE_TO_BIOTIC_STIMULUS	NIR	immune system	defense response		
GO_NEGATIVE_REGULATION_OF_VIRAL_PROCESS	NIR	immune system	defense response		
GO_POSITIVE_REGULATION_OF_MULTI_ORGANISM_PROCESS	NIR	immune system	defense response		
GO_POSITIVE_REGULATION_OF_VIRAL_GENOME_REPLICATION	NIR	immune system	defense response		
GO_POSITIVE_REGULATION_OF_VIRAL_PROCESS	NIR	immune system	defense response		
GO_REGULATION_OF_DEFENSE_RESPONSE_TO_VIRUS	NIR	immune system	defense response		
GO_REGULATION_OF_DEFENSE_RESPONSE_TO_VIRUS_BY_HOST	NIR	immune system	defense response		
GO_REGULATION_OF_DEFENSE_RESPONSE_TO_VIRUS_BY_VIRUS	NIR	immune system	defense response		
GO_REGULATION_OF_MULTI_ORGANISM_PROCESS	NIR	immune system	defense response		
GO_REGULATION_OF_RESPONSE_TO_BIOTIC_STIMULUS	NIR	immune system	defense response		
GO_REGULATION_OF_SYMBIOSIS_ENCOMPASSING_MUTUALISM_THROUGH_PARASITISM	NIR	immune system	defense response		
GO_REGULATION_OF_VIRAL_GENOME_REPLICATION	NIR	immune system	defense response		
GO_REGULATION_OF_VIRAL_TRANSCRIPTION	NIR	immune system	defense response		
GO_RESPONSE_TO_VIRUS	NIR	immune system	defense response		

GO_TRANSPORT_VESICLE	NIR	immune system	defense response		
GO_ACTIVATION_OF_IMMUNE_RESPONSE	NIR	immune system	immune response		
GO_ACTIVATION_OF_INNATE_IMMUNE_RESPONSE	NIR	immune system	immune response		
GO_ADAPTIVE_IMMUNE_RESPONSE	NIR	immune system	immune response		
GO_INNATE_IMMUNE_RESPONSE	NIR	immune system	immune response		
GO_NEGATIVE_REGULATION_OF_ADAPTIVE_IMMUNE_RESPONSE	NIR	immune system	immune response		
GO_NEGATIVE_REGULATION_OF_CELL_ACTIVATION	NIR	immune system	immune response		
GO_NEGATIVE_REGULATION_OF_IMMUNE_EFFECTOR_PROCESS	NIR	immune system	immune response		
GO_NEGATIVE_REGULATION_OF_IMMUNE_RESPONSE	NIR	immune system	immune response		
GO_NEGATIVE_REGULATION_OF_IMMUNE_SYSTEM_PROCESS	NIR	immune system	immune response		
GO_NEGATIVE_REGULATION_OF_INNATE_IMMUNE_RESPONSE	NIR	immune system	immune response		
GO_NEGATIVE_REGULATION_OF_TOLL_LIKE_RECEPTOR_SIGNALING_PATHWAY	NIR	immune system	immune response		
GO_POSITIVE_REGULATION_OF_ADAPTIVE_IMMUNE_RESPONSE	NIR	immune system	immune response		
GO_POSITIVE_REGULATION_OF_CELL_ACTIVATION	NIR	immune system	immune response		
GO_POSITIVE_REGULATION_OF_IMMUNE_RESPONSE	NIR	immune system	immune response		
GO_POSITIVE_REGULATION_OF_INNATE_IMMUNE_RESPONSE	NIR	immune system	immune response		
GO_REGULATION_OF_ADAPTIVE_IMMUNE_RESPONSE	NIR	immune system	immune response		
GO_REGULATION_OF_CELL_ACTIVATION	NIR	immune system	immune response		
GO_REGULATION_OF_IMMUNE_EFFECTOR_PROCESS	NIR	immune system	immune response		
GO_REGULATION_OF_INNATE_IMMUNE_RESPONSE	NIR	immune system	immune response		
GO_REGULATION_OF_TOLERANCE_INDUCTION	NIR	immune system	immune response		
GO_RESPONSE_TO_INTERFERON_ALPHA	NIR	immune system	immune response		
GO_RESPONSE_TO_INTERFERON_BETA	NIR	immune system	immune response		
GO_RESPONSE_TO_TYPE_I_INTERFERON	NIR	immune system	immune response		
ASED_ON_SOMATIC_RECOMBINATION_OF_IMMUNE_RECEPTORS_BUILT_FROM_IMMUI	NIR	immune system	lymphoid lineage	Bcell	immunoglobulin
GO_B_CELL_ACTIVATION	NIR	immune system	lymphoid lineage	Bcell	
GO_B_CELL_ACTIVATION_INVOLVED_IN_IMMUNE_RESPONSE	NIR	immune system	lymphoid lineage	Bcell	
GO_B_CELL_DIFFERENTIATION	NIR	immune system	lymphoid lineage	Bcell	
GO_B_CELL_MEDIATED_IMMUNITY	NIR	immune system	lymphoid lineage	Bcell	
GO_B_CELL_PROLIFERATION	NIR	immune system	lymphoid lineage	Bcell	
GO_IMMUNOGLOBULIN_PRODUCTION	NIR	immune system	lymphoid lineage	Bcell	immunoglobulin
JNOGLOBULIN_PRODUCTION_INVOLVED_IN_IMMUNOGLOBULIN_MEDIATED_IMMUNE_	NIR	immune system	lymphoid lineage	Bcell	immunoglobulin
GO_ISOTYPE_SWITCHING	NIR	immune system	lymphoid lineage	Bcell	immunoglobulin
GO_NEGATIVE_REGULATION_OF_B_CELL_ACTIVATION	NIR	immune system	lymphoid lineage	Bcell	
GO_POSITIVE_REGULATION_OF_B_CELL_ACTIVATION	NIR	immune system	lymphoid lineage	Bcell	
GO_PRODUCTION_OF_MOLECULAR_MEDIATOR_OF_IMMUNE_RESPONSE	NIR	immune system	lymphoid lineage	Bcell	
GO_REGULATION_OF_B_CELL_ACTIVATION	NIR	immune system	lymphoid lineage	Bcell	
GO_REGULATION_OF_B_CELL_DIFFERENTIATION	NIR	immune system	lymphoid lineage	Bcell	
GO_REGULATION_OF_ISOTYPE_SWITCHING	NIR	immune system	lymphoid lineage	Bcell	
GO_SOMATIC_DIVERSIFICATION_OF_IMMUNOGLOBULINS	NIR	immune system	lymphoid lineage	Bcell	immunoglobulin
_SOMATIC_DIVERSIFICATION_OF_IMMUNOGLOBULINS_INVOLVED_IN_IMMUNE_RESPOI	NIR	immune system	lymphoid lineage	Bcell	immunoglobulin
GO_SOMATIC_RECOMBINATION_OF_IMMUNOGLOBULIN_GENE_SEGMENTS	NIR	immune system	lymphoid lineage	Bcell	immunoglobulin
MATIC_RECOMBINATION_OF_IMMUNOGLOBULIN_GENES_INVOLVED_IN_IMMUNE_RES	NIR	immune system	lymphoid lineage	Bcell	immunoglobulin
GO_LYMPHOCYTE_ACTIVATION	NIR	immune system	lymphoid lineage	lymphocyte	
GO_LYMPHOCYTE_ACTIVATION_INVOLVED_IN_IMMUNE_RESPONSE	NIR	immune system	lymphoid lineage	lymphocyte	
GO_LYMPHOCYTE_DIFFERENTIATION	NIR	immune system	lymphoid lineage	lymphocyte	
GO_LYMPHOCYTE_MEDIATED_IMMUNITY	NIR	immune system	lymphoid lineage	lymphocyte	
GO_NEGATIVE_REGULATION_OF_LYMPHOCYTE_DIFFERENTIATION	NIR	immune system	lymphoid lineage	lymphocyte	
GO_NEGATIVE_REGULATION_OF_LYMPHOCYTE_MEDIATED_IMMUNITY	NIR	immune system	lymphoid lineage	lymphocyte	
GO_REGULATION_OF_LYMPHOCYTE_MEDIATED_IMMUNITY	NIR	immune system	lymphoid lineage	lymphocyte	
GO_REGULATION_OF_NATURAL_KILLER_CELL_MEDIATED_IMMUNITY	NIR	immune system	lymphoid lineage	NK cell	
GO_SOMATIC_CELL_DNA_RECOMBINATION	NIR	immune system	lymphoid lineage	RECOMBINATION	
GO_SOMATIC_DIVERSIFICATION_OF_IMMUNE_RECEPTORS	NIR	immune system	lymphoid lineage	RECOMBINATION	
IVERSIFICATION_OF_IMMUNE_RECEPTORS_VIA_GERMLINE_RECOMBINATION_WITHIN_/	NIR	immune system	lymphoid lineage	RECOMBINATION	
GO_V_D_J_RECOMBINATION	NIR	immune system	lymphoid lineage	RECOMBINATION	
GO_ALPHA_BETA_T_CELL_ACTIVATION	NIR	immune system	lymphoid lineage	Tcell	
GO_ALPHA_BETA_T_CELL_DIFFERENTIATION	NIR	immune system	lymphoid lineage	Tcell	
GO_CD4_POSITIVE_ALPHA_BETA_T_CELL_ACTIVATION	NIR	immune system	lymphoid lineage	Tcell	
GO_NEGATIVE_REGULATION_OF_ALPHA_BETA_T_CELL_ACTIVATION	NIR	immune system	lymphoid lineage	Tcell	
GO_NEGATIVE_REGULATION_OF_T_CELL_DIFFERENTIATION	NIR	immune system	lymphoid lineage	Tcell	
GO_NEGATIVE_REGULATION_OF_T_CELL_PROLIFERATION	NIR	immune system	lymphoid lineage	Tcell	
GO_POSITIVE_REGULATION_OF_ALPHA_BETA_T_CELL_ACTIVATION	NIR	immune system	lymphoid lineage	Tcell	
GO_POSITIVE_REGULATION_OF_ALPHA_BETA_T_CELL_DIFFERENTIATION	NIR	immune system	lymphoid lineage	Tcell	
GO_POSITIVE_T_CELL_SELECTION	NIR	immune system	lymphoid lineage	Tcell	
GO_REGULATION_OF_ACTIVATED_T_CELL_PROLIFERATION	NIR	immune system	lymphoid lineage	Tcell	
GO_REGULATION_OF_ALPHA_BETA_T_CELL_ACTIVATION	NIR	immune system	lymphoid lineage	Tcell	
GO_REGULATION_OF_ALPHA_BETA_T_CELL_DIFFERENTIATION	NIR	immune system	lymphoid lineage	Tcell	
GO_REGULATION_OF_ALPHA_BETA_T_CELL_PROLIFERATION	NIR	immune system	lymphoid lineage	Tcell	
GO_REGULATION_OF_T_CELL_DIFFERENTIATION	NIR	immune system	lymphoid lineage	Tcell	
GO_REGULATION_OF_T_CELL_PROLIFERATION	NIR	immune system	lymphoid lineage	Tcell	
GO_T_CELL_ACTIVATION_INVOLVED_IN_IMMUNE_RESPONSE	NIR	immune system	lymphoid lineage	Tcell	
GO_T_CELL_DIFFERENTIATION	NIR	immune system	lymphoid lineage	Tcell	
GO_T_CELL_DIFFERENTIATION_INVOLVED_IN_IMMUNE_RESPONSE	NIR	immune system	lymphoid lineage	Tcell	
GO_T_CELL_MEDIATED_IMMUNITY	NIR	immune system	lymphoid lineage	Tcell	
GO_T_CELL_SELECTION	NIR	immune system	lymphoid lineage	Tcell	
GO_T_HELPER_1_TYPE_IMMUNE_RESPONSE	NIR	immune system	lymphoid lineage	Tcell	
GO_LYMPH_NODE_DEVELOPMENT	NIR	immune system	lymphoid lineage		
GO_POSITIVE_REGULATION_OF_ERYTHROCYTE_DIFFERENTIATION	NIR	immune system	Myeloid lineage	MEP	
GO_REGULATION_OF_ERYTHROCYTE_DIFFERENTIATION	NIR	immune system	Myeloid lineage	MEP	
GO_DENDRITIC_CELL_DIFFERENTIATION	NIR	immune system	Myeloid lineage		
GO_LEUKOCYTE_ACTIVATION	NIR	immune system	Myeloid lineage		
GO_LEUKOCYTE_DIFFERENTIATION	NIR	immune system	Myeloid lineage		
GO_LEUKOCYTE_PROLIFERATION	NIR	immune system	Myeloid lineage		
GO_MYELOID_CELL_DIFFERENTIATION	NIR	immune system	Myeloid lineage		
GO_MYELOID_DENDRITIC_CELL_DIFFERENTIATION	NIR	immune system	Myeloid lineage		
GO_MYELOID_LEUKOCYTE_DIFFERENTIATION	NIR	immune system	Myeloid lineage		
GO_NEGATIVE_REGULATION_OF_LEUKOCYTE_DIFFERENTIATION	NIR	immune system	Myeloid lineage		
GO_NEGATIVE_REGULATION_OF_LEUKOCYTE_MEDIATED_IMMUNITY	NIR	immune system	Myeloid lineage		
GO_NEGATIVE_REGULATION_OF_LEUKOCYTE_PROLIFERATION	NIR	immune system	Myeloid lineage		
GO_NEGATIVE_REGULATION_OF_MYELOID_CELL_DIFFERENTIATION	NIR	immune system	Myeloid lineage		
GO_NEGATIVE_REGULATION_OF_MYELOID_LEUKOCYTE_DIFFERENTIATION	NIR	immune system	Myeloid lineage		
GO_POSITIVE_REGULATION_OF_LEUKOCYTE_DEGRANULATION	NIR	immune system	Myeloid lineage		
GO_POSITIVE_REGULATION_OF_LEUKOCYTE_MEDIATED_IMMUNITY	NIR	immune system	Myeloid lineage		
GO_POSITIVE_REGULATION_OF_MYELOID_LEUKOCYTE_MEDIATED_IMMUNITY	NIR	immune system	Myeloid lineage		
GO_REGULATION_OF_LEUKOCYTE_DEGRANULATION	NIR	immune system	Myeloid lineage		
GO_REGULATION_OF_LEUKOCYTE_DIFFERENTIATION	NIR	immune system	Myeloid lineage		
GO_REGULATION_OF_LEUKOCYTE_MEDIATED_CYTOTOXICITY	NIR	immune system	Myeloid lineage		
GO_REGULATION_OF_LEUKOCYTE_MEDIATED_IMMUNITY	NIR	immune system	Myeloid lineage		
GO_REGULATION_OF_LEUKOCYTE_PROLIFERATION	NIR	immune system	Myeloid lineage		
GO_REGULATION_OF_LYMPHOCYTE_DIFFERENTIATION	NIR	immune system	Myeloid lineage		
GO_REGULATION_OF_MAST_CELL_ACTIVATION	NIR	immune system	Myeloid lineage		
GO_REGULATION_OF_MAST_CELL_ACTIVATION_INVOLVED_IN_IMMUNE_RESPONSE	NIR	immune system	Myeloid lineage		
GO_REGULATION_OF_MAST_CELL_DEGRANULATION	NIR	immune system	Myeloid lineage		

GO_REGULATION_OF_MYELOID_CELL_DIFFERENTIATION	NIR	immune system	Myeloid lineage	
GO_ANTIGEN_PROCESSING_AND_PRESENTATION	NIR	immune system	signal transduction	
GEN_PROCESSING_AND_PRESENTATION_OF_EXOGENOUS_PEPTIDE_ANTIGEN_VIA_MHC	NIR	immune system	signal transduction	
GO_ANTIGEN_PROCESSING_AND_PRESENTATION_OF_PEPTIDE_ANTIGEN	NIR	immune system	signal transduction	
O_ANTIGEN_PROCESSING_AND_PRESENTATION_OF_PEPTIDE_ANTIGEN_VIA_MHC_CLASS	NIR	immune system	signal transduction	
_PROCESSING_AND_PRESENTATION_OF_PEPTIDE_OR_POLYSACCHARIDE_ANTIGEN_VIA_	NIR	immune system	signal transduction	
GO_ANTIGEN_RECEPTOR_MEDIATED_SIGNALING_PATHWAY	NIR	immune system	signal transduction	
GO_B_CELL_RECEPTOR_SIGNALING_PATHWAY	NIR	immune system	signal transduction	
GO_CELLULAR_RESPONSE_TO_GROWTH_HORMONE_STIMULUS	NIR	immune system	signal transduction	
GO_CHEMOKINE_BINDING	NIR	immune system	signal transduction	
GO_CYTOKINE_MEDIATED_SIGNALING_PATHWAY	NIR	immune system	signal transduction	
GO_CYTOKINE_RECEPTOR_ACTIVITY	NIR	immune system	signal transduction	
GO_ENDOLYSOSOME	NIR	immune system	signal transduction	
GO_ER_TO_GOLGI_TRANSPORT_VESICLE	NIR	immune system	signal transduction	
GO_FC_EPSILON_RECEPTOR_SIGNALING_PATHWAY	NIR	immune system	signal transduction	
GO_FC_GAMMA_RECEPTOR_SIGNALING_PATHWAY	NIR	immune system	signal transduction	
GO_FC_RECEPTOR_SIGNALING_PATHWAY	NIR	immune system	signal transduction	
GO_G_PROTEIN_COUPLED_CHEMOATTRACTANT_RECEPTOR_ACTIVITY	NIR	immune system	signal transduction	
O_IMMUNE_RESPONSE_REGULATING_CELL_SURFACE_RECEPTOR_SIGNALING_PATHWA	NIR	immune system	signal transduction	
NNATE_IMMUNE_RESPONSE_ACTIVATING_CELL_SURFACE_RECEPTOR_SIGNALING_PATH	NIR	immune system	signal transduction	
GO_LIPOPOLYSACCHARIDE_BINDING	NIR	immune system	signal transduction	
GO_LUMENAL_SIDE_OF_MEMBRANE	NIR	immune system	signal transduction	
GO_MHC_PROTEIN_COMPLEX	NIR	immune system	signal transduction	
GO_MYD88_DEPENDENT_TOLL_LIKE_RECEPTOR_SIGNALING_PATHWAY	NIR	immune system	signal transduction	
GO_MYD88_INDEPENDENT_TOLL_LIKE_RECEPTOR_SIGNALING_PATHWAY	NIR	immune system	signal transduction	
GO_NON_MEMBRANE_SPANNING_PROTEIN_TYROSINE_KINASE_ACTIVITY	NIR	immune system	signal transduction	
GO_PATTERN_RECOGNITION_RECEPTOR_SIGNALING_PATHWAY	NIR	immune system	signal transduction	
GO_PEPTIDYL_TYROSINE_AUTOPHOSPHORYLATION	NIR	immune system	signal transduction	
GO_PEPTIDYL_TYROSINE_MODIFICATION	NIR	immune system	signal transduction	
GO_POSITIVE_REGULATION_OF_TOLL_LIKE_RECEPTOR_SIGNALING_PATHWAY	NIR	immune system	signal transduction	
GO_PROTEIN_AUTOPHOSPHORYLATION	NIR	immune system	signal transduction	
GO_PROTEIN_POLYUBIQUITINATION	NIR	immune system	signal transduction	
GO_REGULATION_OF_ANTIGEN_PROCESSING_AND_PRESENTATION	NIR	immune system	signal transduction	
GO_REGULATION_OF_TOLL_LIKE_RECEPTOR_4_SIGNALING_PATHWAY	NIR	immune system	signal transduction	
GO_REGULATION_OF_TOLL_LIKE_RECEPTOR_SIGNALING_PATHWAY	NIR	immune system	signal transduction	
GO_T_CELL_RECEPTOR_COMPLEX	NIR	immune system	signal transduction	
GO_T_CELL_RECEPTOR_SIGNALING_PATHWAY	NIR	immune system	signal transduction	
GO_TOLL_LIKE_RECEPTOR_4_SIGNALING_PATHWAY	NIR	immune system	signal transduction	
GO_TOLL_LIKE_RECEPTOR_SIGNALING_PATHWAY	NIR	immune system	signal transduction	
GO_TUMOR_NECROSIS_FACTOR_MEDIATED_SIGNALING_PATHWAY	NIR	immune system	signal transduction	
GO_TUMOR_NECROSIS_FACTOR_RECEPTOR_BINDING	NIR	immune system	signal transduction	
GO_TUMOR_NECROSIS_FACTOR_RECEPTOR_SUPERFAMILY_BINDING	NIR	immune system	signal transduction	
GO_IMMUNE_EFFECTOR_PROCESS	NIR	immune system		
GO_POLYUBIQUITIN_BINDING	NIR	metabolism	Apoptosis	ubiqutin
GO_UBIQUITIN_LIKE_PROTEIN_BINDING	NIR	metabolism	Apoptosis	ubiqutin
GO_DNA_DAMAGE_RESPONSE_SIGNAL_TRANSDUCTION_RESULTING_IN_TRANSCRIPTION	NIR	metabolism	Apoptosis	
IC_APOPTOTIC_SIGNALING_PATHWAY_IN_RESPONSE_TO_DNA_DAMAGE_BY_P53_CLASS	NIR	metabolism	Apoptosis	
_OF_MITOCHONDRIAL_OUTER_MEMBRANE_PERMEABILIZATION_INVOLVED_IN_APOPT	NIR	metabolism	Apoptosis	
N_OF_PROTEIN_INSERTION_INTO_MITOCHONDRIAL_MEMBRANE_INVOLVED_IN_APOPT	NIR	metabolism	Apoptosis	
MITOCHONDRIAL_OUTER_MEMBRANE_PERMEABILIZATION_INVOLVED_IN_APOPTOTIC_	NIR	metabolism	Apoptosis	
_PROTEIN_INSERTION_INTO_MITOCHONDRIAL_MEMBRANE_INVOLVED_IN_APOPTOTIC_	NIR	metabolism	Apoptosis	
GO_SIGNAL_TRANSDUCTION_BY_P53_CLASS_MEDIATOR	NIR	metabolism	Apoptosis	
ON_OF_CYSTEINE_TYPE_ENDOPEPTIDASE_ACTIVITY_INVOLVED_IN_APOPTOTIC_SIGNALI	NIR	metabolism	Apoptosis	
ULATION_OF_CYSTEINE_TYPE_ENDOPEPTIDASE_ACTIVITY_INVOLVED_IN_APOPTOTIC_SIG	NIR	metabolism	Apoptosis	
GO_REGULATION_OF_MICROTUBULE_POLYMERIZATION	PIR	metabolism	Cell proliferation	
GO_ACTIN_NUCLEATION	NIR	metabolism	Cell proliferation	
GO_MICROTUBULE_CYTOSKELETON_ORGANIZATION_INVOLVED_IN_MITOSIS	NIR	metabolism	Cell proliferation	
GO_MITOTIC_SPINDLE_ASSEMBLY	NIR	metabolism	Cell proliferation	
GO_POSITIVE_REGULATION_OF_PROTEIN_COMPLEX_ASSEMBLY	NIR	metabolism	Cell proliferation	
GO_SPINDLE_ASSEMBLY	NIR	metabolism	Cell proliferation	
GO_ATPASE_ACTIVATOR_ACTIVITY	PIR	metabolism	cellular respiration	
GO_ATPASE_REGULATOR_ACTIVITY	PIR	metabolism	cellular respiration	
GO_POSITIVE_REGULATION_OF_ATPASE_ACTIVITY	PIR	metabolism	cellular respiration	
GO_REGULATION_OF_ATPASE_ACTIVITY	PIR	metabolism	cellular respiration	
GO_BETA_CATENIN_TCF_COMPLEX_ASSEMBLY	NIR	Nucleus activity	function	
GO_CHROMATIN	NIR	Nucleus activity	function	
GO_CHROMATIN_SILENCING	NIR	Nucleus activity	function	
GO_CHROMATIN_SILENCING_AT_RDNA	NIR	Nucleus activity	function	
GO_CHROMOSOME_TELOMERIC_REGION	NIR	Nucleus activity	function	
GO_DNA_DEPENDENT_DNA_REPLICATION_MAINTENANCE_OF_FIDELITY	NIR	Nucleus activity	function	
GO_DNA_REPLICATION_DEPENDENT_NUCLEOSOME_ASSEMBLY	NIR	Nucleus activity	function	
GO_DNA_REPLICATION_DEPENDENT_NUCLEOSOME_ORGANIZATION	NIR	Nucleus activity	function	
GO_GENE_SILENCING	NIR	Nucleus activity	function	
GO_GENE_SILENCING_BY_RNA	NIR	Nucleus activity	function	
GO_NEGATIVE_REGULATION_OF_DNA_METABOLIC_PROCESS	NIR	Nucleus activity	function	
GO_NEGATIVE_REGULATION_OF_DNA_RECOMBINATION	NIR	Nucleus activity	function	
GO_NEGATIVE_REGULATION_OF_DNA_REPLICATION	NIR	Nucleus activity	function	
GO_NEGATIVE_REGULATION_OF_GENE_EXPRESSION_EPIGENETIC	NIR	Nucleus activity	function	
GO_NEGATIVE_REGULATION_OF_GENE_SILENCING	NIR	Nucleus activity	function	
GO_NUCLEAR_CHROMATIN	NIR	Nucleus activity	function	
GO_NUCLEAR_CHROMOSOME	NIR	Nucleus activity	function	
GO_NUCLEAR_CHROMOSOME_TELOMERIC_REGION	NIR	Nucleus activity	function	
GO_POSITIVE_REGULATION_OF_GENE_EXPRESSION_EPIGENETIC	NIR	Nucleus activity	function	
GO_PROTEIN_HETEROTETRAMERIZATION	NIR	Nucleus activity	function	
GO_PROTEIN_HOMOTETRAMERIZATION	NIR	Nucleus activity	function	
GO_PROTEIN_TETRAMERIZATION	NIR	Nucleus activity	function	
GO_REGULATION_OF_DNA_METABOLIC_PROCESS	NIR	Nucleus activity	function	
GO_REGULATION_OF_DNA_RECOMBINATION	NIR	Nucleus activity	function	
GO_REGULATION_OF_DOUBLE_STRAND_BREAK_REPAIR	NIR	Nucleus activity	function	
GO_REGULATION_OF_GENE_EXPRESSION_EPIGENETIC	NIR	Nucleus activity	function	
GO_REGULATION_OF_GENE_SILENCING	NIR	Nucleus activity	function	
GO_REGULATION_OF_RESPONSE_TO_DNA_DAMAGE_STIMULUS	NIR	Nucleus activity	function	
GO_TELOMERE_ORGANIZATION	NIR	Nucleus activity	function	
GO_DNA_DEALKYLATION	NIR	Nucleus activity	function	
GO_DNA_MODIFICATION	NIR	Nucleus activity	function	
GO_ENDONUCLEASE_ACTIVITY	NIR	Nucleus activity	nucleotide decomposition	
GO_ENDORIBONUCLEASE_ACTIVITY	NIR	Nucleus activity	nucleotide decomposition	
GO_NUCLEIC_ACID_PHOSPHODIESTER_BOND_HYDROLYSIS	NIR	Nucleus activity	nucleotide decomposition	
GO_RNA_PHOSPHODIESTER_BOND_HYDROLYSIS_ENDONUCLEOLYTIC	NIR	Nucleus activity	nucleotide decomposition	
GO_GLYCOSYL_COMPOUND_CATABOLIC_PROCESS	NIR	Nucleus activity	nucleotide synthesis	
GO_PYRIMIDINE_CONTAINING_COMPOUND_CATABOLIC_PROCESS	NIR	Nucleus activity	nucleotide synthesis	
GO_PYRIMIDINE_NUCLEOSIDE_CATABOLIC_PROCESS	NIR	Nucleus activity	nucleotide synthesis	
GO_PYRIMIDINE_NUCLEOSIDE_METABOLIC_PROCESS	NIR	Nucleus activity	nucleotide synthesis	

GO_PYRIMIDINE_RIBONUCLEOSIDE_METABOLIC_PROCESS	NIR	Nucleus activity	nucleotide synthesis	
GO_RIBONUCLEOSIDE_CATABOLIC_PROCESS	NIR	Nucleus activity	nucleotide synthesis	
GO_NEGATIVE_REGULATION_OF_RNA_SPLICING	PIR	Nucleus activity	RNA regulation	
GO_REGULATION_OF_ALTERNATIVE_MRNA_SPLICING_VIA_SPLICEOSOME	PIR	Nucleus activity	RNA regulation	
GO_REGULATION_OF_MRNA_SPLICING_VIA_SPLICEOSOME	PIR	Nucleus activity	RNA regulation	
GO_MAIN_AXON	PIR	organogenesis	Neural development	neural cells
GO_NEGATIVE_REGULATION_OF_DENDRITE_DEVELOPMENT	PIR	organogenesis	Neural development	neural cells
GO_NEGATIVE_REGULATION_OF_DENDRITE_MORPHOGENESIS	PIR	organogenesis	Neural development	neural cells
GO_POSITIVE_REGULATION_OF_CELL_PROJECTION_ORGANIZATION	PIR	organogenesis	Neural development	neural cells
GO_POSITIVE_REGULATION_OF_DENDRITE_EXTENSION	PIR	organogenesis	Neural development	neural cells
GO_POSITIVE_REGULATION_OF_LAMELLIPODIUM_ASSEMBLY	PIR	organogenesis	Neural development	neural cells
GO_POSITIVE_REGULATION_OF_NEURON_PROJECTION_DEVELOPMENT	PIR	organogenesis	Neural development	neural cells
GO_REGULATION_OF_DENDRITE_DEVELOPMENT	PIR	organogenesis	Neural development	neural cells
GO_REGULATION_OF_DENDRITE_EXTENSION	PIR	organogenesis	Neural development	neural cells
GO_REGULATION_OF_DENDRITE_MORPHOGENESIS	PIR	organogenesis	Neural development	neural cells
GO_REGULATION_OF_NEURON_PROJECTION_DEVELOPMENT	PIR	organogenesis	Neural development	neural cells
GO_SITE_OF_POLARIZED_GROWTH	PIR	organogenesis	Neural development	neural cells
GO_NEURON_PROJECTION_REGENERATION	PIR	organogenesis	Neural development	neural regeneration
GO_AXON_REGENERATION	PIR	organogenesis	Neural development	neural regeneration
GO_NEGATIVE_REGULATION_OF_MACROAUTOPHAGY	PIR	organogenesis	Neural development	nutrient
GO_NEGATIVE_REGULATION_OF_RESPONSE_TO_EXTRACELLULAR_STIMULUS	PIR	organogenesis	Neural development	nutrient
GO_NEGATIVE_REGULATION_OF_RESPONSE_TO_NUTRIENT_LEVELS	PIR	organogenesis	Neural development	nutrient
GO_REGULATION_OF_APPETITE	PIR	organogenesis	Neural development	nutrient
GO_REGULATION_OF_RESPONSE_TO_EXTRACELLULAR_STIMULUS	PIR	organogenesis	Neural development	nutrient
GO_REGULATION_OF_RESPONSE_TO_FOOD	PIR	organogenesis	Neural development	nutrient
GO_REGULATION_OF_RESPONSE_TO_NUTRIENT_LEVELS	PIR	organogenesis	Neural development	nutrient
GO_NEURAL_RETINA_DEVELOPMENT	PIR	organogenesis	Neural development	retine
GO_RETINA_DEVELOPMENT_IN_CAMERA_TYPE_EYE	PIR	organogenesis	Neural development	retine
GO_RETINA_LAYER_FORMATION	PIR	organogenesis	Neural development	retine
GO_RETINA_MORPHOGENESIS_IN_CAMERA_TYPE_EYE	PIR	organogenesis	Neural development	retine
GO_AUDITORY_RECEPTOR_CELL_DEVELOPMENT	PIR	organogenesis	Neural development	sensory of mechanical stimulus
GO_AUDITORY_RECEPTOR_CELL_DIFFERENTIATION	PIR	organogenesis	Neural development	sensory of mechanical stimulus
GO_DETECTION_OF_MECHANICAL_STIMULUS_INVOLVED_IN_SENSORY_PERCEPTION	PIR	organogenesis	Neural development	sensory of mechanical stimulus
GO_SENSORY_PERCEPTION	PIR	organogenesis	Neural development	sensory of mechanical stimulus
GO_SENSORY_PERCEPTION_OF_MECHANICAL_STIMULUS	PIR	organogenesis	Neural development	sensory of mechanical stimulus
GO_PROSTATE_GLAND_DEVELOPMENT	NIR	organogenesis	secretory gland	prostate gland
GO_PROSTATE_GLAND_MORPHOGENESIS	NIR	organogenesis	secretory gland	prostate gland
GO_MAMMARY_GLAND_EPITHELIAL_CELL_DIFFERENTIATION	NIR	organogenesis	secretory gland	
GO_MAMMARY_GLAND_EPITHELIUM_DEVELOPMENT	NIR	organogenesis	secretory gland	
GO_ANATOMICAL_STRUCTURE_HOMEOSTASIS	NIR	organogenesis	Tissue homeostasis	
GO_BONE_RESORPTION	NIR	organogenesis	Tissue homeostasis	
GO_HOMEOSTASIS_OF_NUMBER_OF_CELLS	NIR	organogenesis	Tissue homeostasis	
GO_HOMEOSTASIS_OF_NUMBER_OF_CELLS_WITHIN_A_TISSUE	NIR	organogenesis	Tissue homeostasis	
GO_TISSUE_HOMEOSTASIS	NIR	organogenesis	Tissue homeostasis	
GO_POSITIVE_REGULATION_OF_MYOTUBE_DIFFERENTIATION	NIR	organogenesis	muscle development	
GO_REGULATION_OF_SYNCYTIUM_FORMATION_BY_PLASMA_MEMBRANE_FUSION	NIR	organogenesis	muscle development	
GO_ACTIN_FILAMENT_BASED_MOVEMENT	PIR	physiological function	Cardiovascular function	
GO_ACTIN_MEDIATED_CELL_CONTRACTION	PIR	physiological function	Cardiovascular function	
GO_ACTIN_MYOSIN_FILAMENT_SLIDING	PIR	physiological function	Cardiovascular function	
GO_ACTION_POTENTIAL	PIR	physiological function	Cardiovascular function	
GO_CARDIAC_MUSCLE_CELL_CONTRACTION	PIR	physiological function	Cardiovascular function	
GO_CELL_CELL_SIGNALING_INVOLVED_IN_CARDIAC_CONDUCTION	PIR	physiological function	Cardiovascular function	
GO_CELL_COMMUNICATION_BY_ELECTRICAL_COUPLING	PIR	physiological function	Cardiovascular function	
GO_HEART_PROCESS	PIR	physiological function	Cardiovascular function	
GO_MEMBRANE_DEPOLARIZATION_DURING_ACTION_POTENTIAL	PIR	physiological function	Cardiovascular function	
GO_MUSCLE_CONTRACTION	PIR	physiological function	Cardiovascular function	
GO_MUSCLE_FILAMENT_SLIDING	PIR	physiological function	Cardiovascular function	
GO_MUSCLE_SYSTEM_PROCESS	PIR	physiological function	Cardiovascular function	
GO_REGULATION_OF_BLOOD_CIRCULATION	PIR	physiological function	Cardiovascular function	
GO_REGULATION_OF_HEART_CONTRACTION	PIR	physiological function	Cardiovascular function	
GO_REGULATION_OF_HEART_RATE	PIR	physiological function	Cardiovascular function	
GO_REGULATION_OF_STRIATED_MUSCLE_CONTRACTION	PIR	physiological function	Cardiovascular function	
GO_REGULATION_OF_THE_FORCE_OF_HEART_CONTRACTION	PIR	physiological function	Cardiovascular function	
GO_STRIATED_MUSCLE_CONTRACTION	PIR	physiological function	Cardiovascular function	
GO_VOLTAGE_GATED_ION_CHANNEL_ACTIVITY	PIR	physiological function	Cardiovascular function	
GO_ACETYLCHOLINE_RECEPTOR_ACTIVITY	PIR	physiological function	neural function	synapse
GO_ADULT_BEHAVIOR	PIR	physiological function	neural function	synapse
GO_AMMONIUM_ION_BINDING	PIR	physiological function	neural function	synapse
GO_ANTEROGRADE_AXONAL_TRANSPORT	PIR	physiological function	neural function	synapse
GO_AXON	PIR	physiological function	neural function	synapse
GO_AXON_CYTOPLASM	PIR	physiological function	neural function	synapse
GO_AXON_PART	PIR	physiological function	neural function	synapse
GO_BLOC_1_COMPLEX	PIR	physiological function	neural function	synapse
GO_BLOC_COMPLEX	PIR	physiological function	neural function	synapse
GO_CALCIUM_ION_REGULATED_EXOCYTOSIS	PIR	physiological function	neural function	synapse
GO_CALCIUM_ION_REGULATED_EXOCYTOSIS_OF_NEUROTRANSMITTER	PIR	physiological function	neural function	synapse
GO_CELL_BODY	PIR	physiological function	neural function	synapse
GO_CELL_PROJECTION_CYTOPLASM	PIR	physiological function	neural function	synapse
)_CELL_SURFACE_RECEPTOR_SIGNALING_PATHWAY_INVOLVED_IN_CELL_CELL_SIGNALI	PIR	physiological function	neural function	synapse
GO_CLATHRIN_BINDING	PIR	physiological function	neural function	synapse
GO_CLATHRIN_MEDIATED_ENDOCYTOSIS	PIR	physiological function	neural function	synapse
GO_COPI_COATED_VESICLE	PIR	physiological function	neural function	synapse
GO_CYTOSOLIC_TRANSPORT	PIR	physiological function	neural function	synapse
GO_DENDRITE	PIR	physiological function	neural function	synapse
GO_DENDRITE_MEMBRANE	PIR	physiological function	neural function	synapse
GO_DENDRITIC_SHAFT	PIR	physiological function	neural function	synapse
GO_DNA_DAMAGE_RESPONSE_SIGNAL_TRANSDUCTION_RESULTING_IN_TRANSCRIPTIO	PIR	physiological function	neural function	synapse
GO_DOPAMINE_METABOLIC_PROCESS	PIR	physiological function	neural function	synapse
GO_ESTABLISHMENT_OF_MITOCHONDRION_LOCALIZATION	PIR	physiological function	neural function	synapse
GO_EXCITATORY_EXTRACELLULAR_LIGAND_GATED_ION_CHANNEL_ACTIVITY	PIR	physiological function	neural function	synapse
GO_EXCITATORY_POSTSYNAPTIC_POTENTIAL	PIR	physiological function	neural function	synapse
GO_EXCITATORY_SYNAPSE	PIR	physiological function	neural function	synapse
GO_EXOCYTIC_VESICLE	PIR	physiological function	neural function	synapse
GO_EXOCYTIC_VESICLE_MEMBRANE	PIR	physiological function	neural function	synapse
GO_EXTRACELLULAR_LIGAND_GATED_ION_CHANNEL_ACTIVITY	PIR	physiological function	neural function	synapse
GO_GLUTAMATE_SECRETION	PIR	physiological function	neural function	synapse
GO_INTRASPECIES_INTERACTION_BETWEEN_ORGANISMS	PIR	physiological function	neural function	synapse
GO_LIGAND_GATED_CHANNEL_ACTIVITY	PIR	physiological function	neural function	synapse
GO_PIR_TERM_MEMORY	PIR	physiological function	neural function	synapse
GO_PIR_TERM_SYNAPTIC_DEPRESSION	PIR	physiological function	neural function	synapse
GO_MITOCHONDRION_LOCALIZATION	PIR	physiological function	neural function	synapse
GO_MODULATION_OF_EXCITATORY_POSTSYNAPTIC_POTENTIAL	PIR	physiological function	neural function	synapse
GO_MODULATION_OF_SYNAPTIC_TRANSMISSION	PIR	physiological function	neural function	synapse

GO_NEGATIVE_REGULATION_OF_AMINE_TRANSPORT	PIR	physiological function	neural function	synapse
GO_NEGATIVE_REGULATION_OF_CATECHOLAMINE_SECRETION	PIR	physiological function	neural function	synapse
GO_NEUROMUSCULAR_SYNAPTIC_TRANSMISSION	PIR	physiological function	neural function	synapse
GO_NEURON_NEURON_SYNAPTIC_TRANSMISSION	PIR	physiological function	neural function	synapse
GO_NEURON_PROJECTION_MEMBRANE	PIR	physiological function	neural function	synapse
GO_NEURON_PROJECTION_TERMINUS	PIR	physiological function	neural function	synapse
GO_NEURONAL_POSTSYNAPTIC_DENSITY	PIR	physiological function	neural function	synapse
GO_NEUROTRANSMITTER_BINDING	PIR	physiological function	neural function	synapse
GO_NEUROTRANSMITTER_METABOLIC_PROCESS	PIR	physiological function	neural function	synapse
GO_NEUROTRANSMITTER_RECEPTOR_ACTIVITY	PIR	physiological function	neural function	synapse
GO_NEUROTRANSMITTER_TRANSPORT	PIR	physiological function	neural function	synapse
GO_ORGANELLE_FUSION	PIR	physiological function	neural function	synapse
GO_ORGANELLE_LOCALIZATION	PIR	physiological function	neural function	synapse
GO_ORGANELLE_MEMBRANE_FUSION	PIR	physiological function	neural function	synapse
GO_ORGANELLE_TRANSPORT_APIR_MICROTUBULE	PIR	physiological function	neural function	synapse
GO_PERIKARYON	PIR	physiological function	neural function	synapse
GO_POSITIVE_REGULATION_OF_EXCITATORY_POSTSYNAPTIC_POTENTIAL	PIR	physiological function	neural function	synapse
GO_POSITIVE_REGULATION_OF_SYNAPSE_ASSEMBLY	PIR	physiological function	neural function	synapse
GO_POSITIVE_REGULATION_OF_SYNAPTIC_TRANSMISSION	PIR	physiological function	neural function	synapse
GO_POSTSYNAPSE	PIR	physiological function	neural function	synapse
GO_POSTSYNAPTIC_MEMBRANE	PIR	physiological function	neural function	synapse
GO_PRESYNAPSE	PIR	physiological function	neural function	synapse
GO_PRESYNAPTIC_ACTIVE_ZONE	PIR	physiological function	neural function	synapse
GO_PRESYNAPTIC_MEMBRANE	PIR	physiological function	neural function	synapse
GO_PRESYNAPTIC_PROCESS_INVOLVED_IN_SYNAPTIC_TRANSMISSION	PIR	physiological function	neural function	synapse
GO_REGULATION_OF_CALCIUM_ION_DEPENDENT_EXOCYTOSIS	PIR	physiological function	neural function	synapse
GO_REGULATION_OF_CATECHOLAMINE_METABOLIC_PROCESS	PIR	physiological function	neural function	synapse
GO_REGULATION_OF_DOPAMINE_METABOLIC_PROCESS	PIR	physiological function	neural function	synapse
GO_REGULATION_OF_PIR_TERM_NEURONAL_SYNAPTIC_PLASTICITY	PIR	physiological function	neural function	synapse
GO_REGULATION_OF_MEMBRANE_POTENTIAL	PIR	physiological function	neural function	synapse
GO_REGULATION_OF_NEURONAL_SYNAPTIC_PLASTICITY	PIR	physiological function	neural function	synapse
GO_REGULATION_OF_NEUROTRANSMITTER_LEVELS	PIR	physiological function	neural function	synapse
GO_REGULATION_OF_NEUROTRANSMITTER_SECRETION	PIR	physiological function	neural function	synapse
GO_REGULATION_OF_NOREPINEPHRINE_SECRETION	PIR	physiological function	neural function	synapse
GO_REGULATION_OF_POSTSYNAPTIC_MEMBRANE_POTENTIAL	PIR	physiological function	neural function	synapse
GO_REGULATION_OF_SYNAPSE_ASSEMBLY	PIR	physiological function	neural function	synapse
GO_REGULATION_OF_SYNAPSE_ORGANIZATION	PIR	physiological function	neural function	synapse
GO_REGULATION_OF_SYNAPSE_STRUCTURE_OR_ACTIVITY	PIR	physiological function	neural function	synapse
GO_REGULATION_OF_SYNAPTIC_PLASTICITY	PIR	physiological function	neural function	synapse
GO_REGULATION_OF_SYNAPTIC_VESICLE_EXOCYTOSIS	PIR	physiological function	neural function	synapse
GO_REGULATION_OF_SYNAPTIC_VESICLE_TRANSPORT	PIR	physiological function	neural function	synapse
GO_RESPONSE_TO_ALKALOID	PIR	physiological function	neural function	synapse
GO_RESPONSE_TO_ISOQUINOLINE_ALKALOID	PIR	physiological function	neural function	synapse
GO_RESPONSE_TO_MORPHINE	PIR	physiological function	neural function	synapse
GO_SIGNAL_RELEASE	PIR	physiological function	neural function	synapse
GO_SINGLE_ORGANISM_MEMBRANE_FUSION	PIR	physiological function	neural function	synapse
GO_SNAP_RECEPTOR_ACTIVITY	PIR	physiological function	neural function	synapse
GO_SNARE_BINDING	PIR	physiological function	neural function	synapse
GO_SNARE_COMPLEX	PIR	physiological function	neural function	synapse
GO_SOCIAL_BEHAVIOR	PIR	physiological function	neural function	synapse
GO_STARTLE_RESPONSE	PIR	physiological function	neural function	synapse
GO_SYNAPTIC_MEMBRANE	PIR	physiological function	neural function	synapse
GO_SYNAPTIC_SIGNALING	PIR	physiological function	neural function	synapse
GO_SYNAPTIC_TRANSMISSION_CHOLINERGIC	PIR	physiological function	neural function	synapse
GO_SYNAPTIC_TRANSMISSION_DOPAMINERGIC	PIR	physiological function	neural function	synapse
GO_SYNAPTIC_TRANSMISSION_GLUTAMATERGIC	PIR	physiological function	neural function	synapse
GO_SYNAPTIC_VESICLE_CYCLE	PIR	physiological function	neural function	synapse
GO_SYNAPTIC_VESICLE_ENDOCYTOSIS	PIR	physiological function	neural function	synapse
GO_SYNAPTIC_VESICLE_LOCALIZATION	PIR	physiological function	neural function	synapse
GO_SYNAPTIC_VESICLE_RECYCLING	PIR	physiological function	neural function	synapse
GO_SYNTAXIN_1_BINDING	PIR	physiological function	neural function	synapse
GO_SYNTAXIN_BINDING	PIR	physiological function	neural function	synapse
GO_TERMINAL_BOUTON	PIR	physiological function	neural function	synapse
GO_TRANSPORT_VESICLE	PIR	physiological function	neural function	synapse
GO_VESICLE_DOCKING	PIR	physiological function	neural function	synapse
GO_VESICLE_LOCALIZATION	PIR	physiological function	neural function	synapse
GO_VESICLE_ORGANIZATION	PIR	physiological function	neural function	synapse
GO_INOSITOL_LIPID_MEDIATED_SIGNALING	NIR	Signal transduction	lipid	
GO_LIPID_PHOSPHORYLATION	NIR	Signal transduction	lipid	
GO_PHOSPHATIDYLINOSITOL_3_KINASE_ACTIVITY	NIR	Signal transduction	lipid	
GO_PHOSPHATIDYLINOSITOL_3_KINASE_COMPLEX	NIR	Signal transduction	lipid	
GO_REGULATION_OF_PHOSPHATIDYLINOSITOL_3_KINASE_SIGNALING	NIR	Signal transduction	lipid	
GO_JNK_CASCADE	NIR	Signal transduction	protein	
GO_POSITIVE_REGULATION_OF_JUN_KINASE_ACTIVITY	NIR	Signal transduction	protein	
O_POSITIVE_REGULATION_OF_STRESS_ACTIVATED_PROTEIN_KINASE_SIGNALING_CASCA	NIR	Signal transduction	protein	
GO_REGULATION_OF_JNK_CASCADE	NIR	Signal transduction	protein	
GO_REGULATION_OF_JUN_KINASE_ACTIVITY	NIR	Signal transduction	protein	
GO_REGULATION_OF_STRESS_ACTIVATED_PROTEIN_KINASE_SIGNALING_CASCADE	NIR	Signal transduction	protein	
GO_STRESS_ACTIVATED_PROTEIN_KINASE_SIGNALING_CASCADE	NIR	Signal transduction	protein	
GO_SIGNALING_ADAPTOR_ACTIVITY	NIR	Signal transduction	protein	
GO_SH3_SH2_ADAPTOR_ACTIVITY	NIR	Signal transduction	protein	
GO_ANDROGEN_RECEPTOR_SIGNALING_PATHWAY	NIR	Signal transduction	sterols	
GO_BHLH_TRANSCRIPTION_FACTOR_BINDING	NIR	Signal transduction	sterols	
GO_DNA_BINDING_BENDING	NIR	Signal transduction	sterols	
GO_DNA_TEMPLATED_TRANSCRIPTION_INITIATION	NIR	Signal transduction	sterols	
GO_ESTROGEN_RECEPTOR_BINDING	NIR	Signal transduction	sterols	
GO_INTRACELLULAR_RECEPTOR_SIGNALING_PATHWAY	NIR	Signal transduction	sterols	
GO_REPRESSING_TRANSCRIPTION_FACTOR_BINDING	NIR	Signal transduction	sterols	
GO_RETINOIC_ACID_RECEPTOR_BINDING	NIR	Signal transduction	sterols	
GO_STEROID_HORMONE_MEDIATED_SIGNALING_PATHWAY	NIR	Signal transduction	sterols	
GO_STEROID_HORMONE_RECEPTOR_ACTIVITY	NIR	Signal transduction	sterols	
GO_STEROID_HORMONE_RECEPTOR_BINDING	NIR	Signal transduction	sterols	
RIPTION_FACTOR_ACTIVITY_RNA_POLYMERASE_II_DISTAL_ENHANCER_SEQUENCE_SPECI	NIR	Signal transduction	sterols	
GO_TRANSCRIPTION_FACTOR_BINDING	NIR	Signal transduction	sterols	
_REGULATION_OF_INTRACELLULAR_STEROID_HORMONE_RECEPTOR_SIGNALING_PATHV	NIR	Signal transduction	sterols	
ATIVE_REGULATION_OF_INTRACELLULAR_STEROID_HORMONE_RECEPTOR_SIGNALING_I	NIR	Signal transduction	sterols	
GO_POSITIVE_REGULATION_OF_POTASSIUM_ION_TRANSMEMBRANE_TRANSPORT	PIR	transport	synaptic	
_POSITIVE_REGULATION_OF_POTASSIUM_ION_TRANSMEMBRANE_TRANSPORTER_ACTIV	PIR	transport	synaptic	
GO_POSITIVE_REGULATION_OF_POTASSIUM_ION_TRANSPORT	PIR	transport	synaptic	
GO_POSITIVE_REGULATION_OF_TRANSPORTER_ACTIVITY	PIR	transport	synaptic	
GO_REGULATION_OF_CALCIUM_ION_TRANSMEMBRANE_TRANSPORTER_ACTIVITY	PIR	transport	synaptic	
GO_REGULATION_OF_CATION_CHANNEL_ACTIVITY	PIR	transport	synaptic	
GO_REGULATION_OF_POTASSIUM_ION_TRANSMEMBRANE_TRANSPORTER_ACTIVITY	PIR	transport	synaptic	

GO_REGULATION_OF_VOLTAGE_GATED_CALCIUM_CHANNEL_ACTIVITY	PIR	transport	synaptic
GO_NEGATIVE_REGULATION_OF_CALCIUM_MEDIATED_SIGNALING	NIR	transport	synaptic
GO_ACTIVE_ION_TRANSMEMBRANE_TRANSPORTER_ACTIVITY	PIR	transport	
GO_ACTIVE_TRANSMEMBRANE_TRANSPORTER_ACTIVITY	PIR	transport	
GO_AMINO_ACID_TRANSMEMBRANE_TRANSPORT	PIR	transport	
GO_AMINO_ACID_TRANSMEMBRANE_TRANSPORTER_ACTIVITY	PIR	transport	
GO_AMINO_ACID_TRANSPORT	PIR	transport	
GO_AMMONIUM_TRANSMEMBRANE_TRANSPORT	PIR	transport	
GO_AMMONIUM_TRANSPORT	PIR	transport	
GO_ANION_CATION_SYMPORTER_ACTIVITY	PIR	transport	
GO_ANION_TRANSMEMBRANE_TRANSPORT	PIR	transport	
GO_ANION_TRANSMEMBRANE_TRANSPORTER_ACTIVITY	PIR	transport	
GO_ANION_TRANSPORT	PIR	transport	
GO_CATION_CHANNEL_ACTIVITY	PIR	transport	
GO_CATION_CHANNEL_COMPLEX	PIR	transport	
GO_CHLORIDE_CHANNEL_COMPLEX	PIR	transport	
GO_CHLORIDE_TRANSPORT	PIR	transport	
GO_DELAYED_RECTIFIER_POTASSIUM_CHANNEL_ACTIVITY	PIR	transport	
GO_GATED_CHANNEL_ACTIVITY	PIR	transport	
GO_INORGANIC_ANION_TRANSMEMBRANE_TRANSPORTER_ACTIVITY	PIR	transport	
GO_INORGANIC_ANION_TRANSPORT	PIR	transport	
GO_INORGANIC_CATION_TRANSMEMBRANE_TRANSPORTER_ACTIVITY	PIR	transport	
GO_L_AMINO_ACID_TRANSMEMBRANE_TRANSPORTER_ACTIVITY	PIR	transport	
GO_L_AMINO_ACID_TRANSPORT	PIR	transport	
GO_METAL_ION_TRANSMEMBRANE_TRANSPORTER_ACTIVITY	PIR	transport	
GO_MONOVALENT_INORGANIC_CATION_TRANSMEMBRANE_TRANSPORTER_ACTIVITY	PIR	transport	
GO_MONOVALENT_INORGANIC_CATION_TRANSPORT	PIR	transport	
GO_NEUROTRANSMITTER_SODIUM_SYMPORTER_ACTIVITY	PIR	transport	
GO_NEUROTRANSMITTER_TRANSPORTER_ACTIVITY	PIR	transport	
GO_NEUTRAL_AMINO_ACID_TRANSMEMBRANE_TRANSPORTER_ACTIVITY	PIR	transport	
GO_NEUTRAL_AMINO_ACID_TRANSPORT	PIR	transport	
GO_NITROGEN_COMPOUND_TRANSPORT	PIR	transport	
GO_NUCLEOTIDE_TRANSPORT	PIR	transport	
GO_ORGANIC_ACID_SODIUM_SYMPORTER_ACTIVITY	PIR	transport	
GO_ORGANIC_ACID_TRANSMEMBRANE_TRANSPORT	PIR	transport	
GO_ORGANIC_ACID_TRANSMEMBRANE_TRANSPORTER_ACTIVITY	PIR	transport	
GO_ORGANIC_ACID_TRANSPORT	PIR	transport	
GO_PASSIVE_TRANSMEMBRANE_TRANSPORTER_ACTIVITY	PIR	transport	
GO_POTASSIUM_CHANNEL_COMPLEX	PIR	transport	
GO_SODIUM_CHANNEL_ACTIVITY	PIR	transport	
GO_SODIUM_CHANNEL_COMPLEX	PIR	transport	
GO_SODIUM_ION_TRANSMEMBRANE_TRANSPORT	PIR	transport	
GO_SODIUM_ION_TRANSMEMBRANE_TRANSPORTER_ACTIVITY	PIR	transport	
GO_SODIUM_ION_TRANSPORT	PIR	transport	
GO_SOLUTE_CATION_SYMPORTER_ACTIVITY	PIR	transport	
GO_SOLUTE_SODIUM_SYMPORTER_ACTIVITY	PIR	transport	
GO_SYMPORTER_ACTIVITY	PIR	transport	
GO_TRANSPORTER_COMPLEX	PIR	transport	
GO_VOLTAGE_GATED_CATION_CHANNEL_ACTIVITY	PIR	transport	
GO_VOLTAGE_GATED_SODIUM_CHANNEL_ACTIVITY	PIR	transport	

Supplementary Data 2C. The grouping of GO terms in Cohort 3.

GO_term	Enrichment in	Type 1	Type 2
GO_POSITIVE_REGULATION_OF_ENDOTHELIAL_CELL_APOPTOTIC_PROCESS	NIR	angiogenesis	apototic
GO_POSITIVE_REGULATION_OF_EPITHELIAL_CELL_APOPTOTIC_PROCESS	NIR	angiogenesis	apototic
GO_REGULATION_OF_ENDOTHELIAL_CELL_APOPTOTIC_PROCESS	NIR	angiogenesis	apototic
GO_REGULATION_OF_EPITHELIAL_CELL_APOPTOTIC_PROCESS	NIR	angiogenesis	apototic
GO_ENDOTHELIUM_DEVELOPMENT	NIR	angiogenesis	
GO_MORPHOGENESIS_OF_AN_ENDOTHELIUM	NIR	angiogenesis	
GO_NEGATIVE_REGULATION_OF_BLOOD_VESSEL_ENDOTHELIAL_CELL_MIGRATION	NIR	angiogenesis	
GO_NEGATIVE_REGULATION_OF_ENDOTHELIAL_CELL_MIGRATION	NIR	angiogenesis	
GO_NEGATIVE_REGULATION_OF_ENDOTHELIAL_CELL_PROLIFERATION	NIR	angiogenesis	
GO_NEGATIVE_REGULATION_OF_EPITHELIAL_CELL_MIGRATION	NIR	angiogenesis	
GO_POSITIVE_REGULATION_OF_ENDOTHELIAL_CELL_MIGRATION	NIR	angiogenesis	
GO_POSITIVE_REGULATION_OF_EPITHELIAL_CELL_MIGRATION	NIR	angiogenesis	
GO_REGULATION_OF_BLOOD_VESSEL_ENDOTHELIAL_CELL_MIGRATION	NIR	angiogenesis	
GO_REGULATION_OF_ENDOTHELIAL_CELL_CHEMOTAXIS	NIR	angiogenesis	
GO_REGULATION_OF_ENDOTHELIAL_CELL_MIGRATION	NIR	angiogenesis	
GO_REGULATION_OF_ENDOTHELIAL_CELL_PROLIFERATION	NIR	angiogenesis	
GO_REGULATION_OF_EPITHELIAL_CELL_MIGRATION	NIR	angiogenesis	
GO_REGULATION_OF_VASCULAR_ENDOTHELIAL_GROWTH_FACTOR_RECEPTOR_SIGNALING_PATHWAY	NIR	angiogenesis	
GO_NEGATIVE_REGULATION_OF_SMOOTH_MUSCLE_CELL_PROLIFERATION	NIR	angiogenesis	
GO_ARTERY_MORPHOGENESIS	NIR	angiogenesis	
GO_INTRACILIARY_TRANSPORT	PIR	cell structure	intracellular transport
GO_INTRACILIARY_TRANSPORT_PARTICLE	PIR	cell structure	intracellular transport
GO_INTRACILIARY_TRANSPORT_PARTICLE_B	PIR	cell structure	intracellular transport
GO_ANCHORED_COMPONENT_OF_EXTERNAL_SIDE_OF_PLASMA_MEMBRANE	NIR	cell structure	plasma membrane
GO_ANCHORED_COMPONENT_OF_PLASMA_MEMBRANE	NIR	cell structure	plasma membrane
GO_INTRINSIC_COMPONENT_OF_EXTERNAL_SIDE_OF_PLASMA_MEMBRANE	NIR	cell structure	plasma membrane
GO_AXONEMAL_DYNEIN_COMPLEX_ASSEMBLY	PIR	cell structure	
GO_AXONEME_ASSEMBLY	PIR	cell structure	
GO_AXONEME_PART	PIR	cell structure	
GO_CELLULAR_COMPONENT_ASSEMBLY_INVOLVED_IN_MORPHOGENESIS	PIR	cell structure	
GO_CELL_PROJECTION_ASSEMBLY	PIR	cell structure	
GO_CENTRIOLAR_SATELLITE	PIR	cell structure	
GO_CILIARY_BASAL_BODY	PIR	cell structure	
GO_CILIARY_PART	PIR	cell structure	
GO_CILIARY_PLASM	PIR	cell structure	
GO_CILIARY_TIP	PIR	cell structure	
GO_CILIARY_TRANSITION_ZONE	PIR	cell structure	
GO_CILIUM	PIR	cell structure	
GO_CILIUM_MORPHOGENESIS	PIR	cell structure	
GO_CILIUM_MOVEMENT	PIR	cell structure	
GO_CILIUM_ORGANIZATION	PIR	cell structure	
GO_DYNEIN_COMPLEX	PIR	cell structure	
GO_EPITHELIAL_CILIUM_MOVEMENT	PIR	cell structure	
GO_MICROTUBULE_BASED_MOVEMENT	PIR	cell structure	
GO_MICROTUBULE_BUNDLE_FORMATION	PIR	cell structure	
GO_MOTILE_CILIUM	PIR	cell structure	
GO_NONMOTILE_PRIMARY_CILIUM	PIR	cell structure	
GO_NONMOTILE_PRIMARY_CILIUM_ASSEMBLY	PIR	cell structure	
GO_PHOTORECEPTOR_CONNECTING_CILIUM	PIR	cell structure	
GO_PRIMARY_CILIUM	PIR	cell structure	
GO_PROTEIN_COMPLEX_LOCALIZATION	PIR	cell structure	
GO_PROTEIN_TRANSPORT_APIR_MICROTUBULE	PIR	cell structure	
GO_NEGATIVE_REGULATION_OF_NF_KAPPAB_IMPORT_INTO_NUCLEUS	NIR	immune system	cytokine
GO_NEGATIVE_REGULATION_OF_INTERLEUKIN_12_PRODUCTION	NIR	immune system	cytokine
GO_CYTOKINE_BINDING	NIR	immune system	cytokine
GO_MODULATION_BY_HOST_OF_VIRAL_PROCESS	NIR	immune system	defense response
GO_INFLAMMATORY_RESPONSE_TO_ANTIGENIC_STIMULUS	NIR	immune system	defense response
GO_POSITIVE_REGULATION_OF_NATURAL_KILLER_CELL_MEDIATED_IMMUNITY	NIR	immune system	lymphoid lineage
GO_T_CELL_DIFFERENTIATION_INVOLVED_IN_IMMUNE_RESPONSE	NIR	immune system	lymphoid lineage
GO_ANTIGEN_PROCESSING_AND_PRESENTATION_VIA_MHC_CLASS_IB	NIR	immune system	lymphoid lineage
GO_MAST_CELL_GRANULE	NIR	immune system	myeloid lineage
GO_REGULATION_OF_MACROPHAGE_ACTIVATION	NIR	immune system	myeloid lineage
GO_NEGATIVE_REGULATION_OF_LYMPHOCYTE_APOPTOTIC_PROCESS	NIR	immune system	lymphoid lineage
GO_NEGATIVE_REGULATION_OF_T_CELL_APOPTOTIC_PROCESS	NIR	immune system	lymphoid lineage
GO_POSITIVE_REGULATION_OF_LYMPHOCYTE_APOPTOTIC_PROCESS	NIR	immune system	lymphoid lineage
GO_REGULATION_OF_B_CELL_APOPTOTIC_PROCESS	NIR	immune system	lymphoid lineage
GO_REGULATION_OF_LYMPHOCYTE_APOPTOTIC_PROCESS	NIR	immune system	lymphoid lineage
GO_REGULATION_OF_T_CELL_APOPTOTIC_PROCESS	NIR	immune system	lymphoid lineage
GO_AMMONIUM_ION_BINDING	NIR	metabolism	ammonium
GO_PHOSPHATIDYLCHOLINE_BINDING	NIR	metabolism	ammonium
GO_QUATERNARY_AMMONIUM_GROUP_BINDING	NIR	metabolism	ammonium
GO_POSITIVE_REGULATION_OF_APOPTOTIC_SIGNALING_PATHWAY	NIR	metabolism	apotosis
GO_POSITIVE_REGULATION_OF_EXTRINSIC_APOPTOTIC_SIGNALING_PATHWAY_VIA_DEATH_DOMAIN_RECEPTORS	NIR	metabolism	apotosis
GO_POSITIVE_REGULATION_OF_INTRINSIC_APOPTOTIC_SIGNALING_PATHWAY	NIR	metabolism	apotosis
GO_ATP_HYDROLYSIS_COUPLED_TRANSMEMBRANE_TRANSPORT	NIR	metabolism	cell respiration
GO_HYDROGEN_EXPORTING_ATPASE_ACTIVITY	NIR	metabolism	cell respiration
GO_PH_REDUCTION	NIR	metabolism	cell respiration
GO_PROTON_TRANSPORTING_TWO_SECTOR_ATPASE_COMPLEX_CATALYTIC_DOMAIN	NIR	metabolism	cell respiration
GO_PROTON_TRANSPORTING_V_TYPE_ATPASE_COMPLEX	NIR	metabolism	cell respiration
GO_REGULATION_OF_CELLULAR_PH	NIR	metabolism	cell respiration
GO_REGULATION_OF_PH	NIR	metabolism	cell respiration
GO_COATED_PIT	NIR	metabolism	cytosis
GO_PROTEIN_LIPID_COMPLEX_BINDING	NIR	metabolism	cytosis
GO_RECEPTOR_MEDIATED_ENDOCYTOSIS	NIR	metabolism	cytosis
GO_EMBRYONIC_HEMOPOIESIS	NIR	metabolism	homeostasis
GO_MACROMOLECULE_METHYLATION	PIR	metabolism	methylation
GO_S_ADENOSYLMETHIONINE_DEPENDENT_METHYLTRANSFERASE_ACTIVITY	PIR	metabolism	methylation
GO_TRNA_METHYLATION	PIR	metabolism	methylation
GO_NEGATIVE_REGULATION_OF_ERBB_SIGNALING_PATHWAY	NIR	metabolism	signal transport

GO_NEGATIVE_REGULATION_OF_PROTEIN_TYROSINE_KINASE_ACTIVITY	NIR	metabolism	signal transport
GO_REGULATION_OF_PROTEIN_TYROSINE_KINASE_ACTIVITY	NIR	metabolism	signal transport
GO_CHOLESTEROL_HOMEOSTASIS	NIR	metabolism	sterol
GO_HIGH_DENSITY_LIPOPROTEIN_PARTICLE	NIR	metabolism	sterol
GO_PLASMA_LIPOPROTEIN_PARTICLE_CLEARANCE	NIR	metabolism	sterol
GO_STEROL_HOMEOSTASIS	NIR	metabolism	sterol
GO_TRIGLYCERIDE_RICH_LIPOPROTEIN_PARTICLE	NIR	metabolism	sterol
GO_VERY_LOW_DENSITY_LIPOPROTEIN_PARTICLE	NIR	metabolism	sterol
GO_FILAMENTOUS_ACTIN	NIR	organogenesis	muscle
GO_ACTIN_CYTOSKELETON_REORGANIZATION	NIR	organogenesis	muscle
GO_ACTOMYOSIN	NIR	organogenesis	muscle
GO_POSITIVE_REGULATION_OF_HEART_CONTRACTION	NIR	physiological function	Cardiovascular function
GO_POSITIVE_REGULATION_OF_HEART_RATE	NIR	physiological function	Cardiovascular function
GO_POSITIVE_REGULATION_OF_STRIATED_MUSCLE_CONTRACTION	NIR	physiological function	Cardiovascular function
GO_REGULATION_OF_MUSCLE_CONTRACTION	NIR	physiological function	Cardiovascular function
GO_SPECIFICATION_OF_SYMMETRY	PIR	physiological function	fertility
GO_SPERM_FLAGELLUM	PIR	physiological function	fertility
GO_SPERM_MOTILITY	PIR	physiological function	fertility
GO_FERTILIZATION	PIR	physiological function	fertility
GO_SINGLE_FERTILIZATION	PIR	physiological function	fertility
GO_SPERM_EGG_RECOGNITION	PIR	physiological function	fertility
GO_REGULATION_OF_RESPIRATORY_SYSTEM_PROCESS	PIR	physiological function	respiratoration
GO_REGULATION_OF_RESPIRATORY_GASEOUS_EXCHANGE	PIR	physiological function	respiratoration

Supplementary Data 2D. The grouping of GO terms in Cohort 4.

GO_term	Enrichment in	Type 1	Type 2	Type 3
GO_REGULATION_OF_CELL_SUBSTRATE_ADHESION	NIR	cell migration		
GO_POSITIVE_REGULATION_OF_CELL_JUNCTION_ASSEMBLY	NIR	cell migration		
GO_EXTRACELLULAR_MATRIX	NIR	cell structure	Extracellular components	
GO_EXTRACELLULAR_MATRIX_COMPONENT	NIR	cell structure	Extracellular components	
GO_COLLAGEN_TRIMER	NIR	cell structure	Extracellular components	
GO_FRIZZLED_BINDING	NIR	cell structure	Extracellular components	
GO_PROTEINACEOUS_EXTRACELLULAR_MATRIX	NIR	cell structure	Extracellular components	
GO_RECEPTOR_AGONIST_ACTIVITY	NIR	cell structure	Extracellular components	
GO_COLLAGEN_FIBRIL_ORGANIZATION	NIR	cell structure	Extracellular components	
GO_RECEPTOR_ACTIVATOR_ACTIVITY	NIR	cell structure	Extracellular components	
GO_RECEPTOR_REGULATOR_ACTIVITY	NIR	cell structure	Extracellular components	
GO_ER_TO_GOLGI_TRANSPORT_VESICLE	NIR	cell structure	Intracellular components	
GO_ER_TO_GOLGI_TRANSPORT_VESICLE_MEMBRANE	NIR	cell structure	Intracellular components	
GO_REGULATION_OF_MEMBRANE_LIPID_DISTRIBUTION	NIR	cell structure	trans-membrane	
GO_LIPID_TRANSLOCATION	NIR	cell structure	trans-membrane	
GO_TUMOR_NECROSIS_FACTOR_RECEPTOR_SUPERFAMILY_BINDING	NIR	immune system	cytokine	
GO_TUMOR_NECROSIS_FACTOR_RECEPTOR_BINDING	NIR	immune system	cytokine	
GO_REGULATION_OF_I_KAPPAB_KINASE_NF_KAPPAB_SIGNALING	NIR	immune system	cytokine	
GO_DEATH_RECEPTOR_BINDING	NIR	immune system	cytokine	
GO_NEGATIVE_REGULATION_OF_I_KAPPAB_KINASE_NF_KAPPAB_SIGNALING	NIR	immune system	cytokine	
GO_NEGATIVE_REGULATION_OF_CYTOKINE_BIOSYNTHETIC_PROCESS	NIR	immune system	cytokine	
GO_NEGATIVE_REGULATION_OF_CYTOKINE_PRODUCTION	NIR	immune system	cytokine	
GO_INTERACTION_WITH_SYMBIONT	NIR	immune system	defense response	
GO_NEGATIVE_REGULATION_OF_RESPONSE_TO_BIOTIC_STIMULUS	NIR	immune system	defense response	
GO_NEGATIVE_REGULATION_OF_MULTI_ORGANISM_PROCESS	NIR	immune system	defense response	
GO_REGULATION_OF_MULTI_ORGANISM_PROCESS	NIR	immune system	defense response	
GO_REGULATION_OF_SYMBIOSIS_ENCOMPASSING_MUTUALISM_THROUGH_PARASITISM	NIR	immune system	defense response	
GO_REGULATION_OF_DEFENSE_RESPONSE_TO_VIRUS	NIR	immune system	defense response	
GO_NEGATIVE_REGULATION_OF_VIRAL_TRANSCRIPTION	NIR	immune system	defense response	
GO_REGULATION_OF_LIPOPOLYSACCHARIDE_MEDIATED_SIGNALING_PATHWAY	NIR	immune system	defense response	
GO_NEGATIVE_REGULATION_OF_VIRAL_PROCESS	NIR	immune system	defense response	
GO_NEGATIVE_REGULATION_OF_DEFENSE_RESPONSE_TO_VIRUS	NIR	immune system	defense response	
GO_MODIFICATION_OF_MORPHOLOGY_OR_PHYSIOLOGY_OF_OTHER_ORGANISM	NIR	immune system	defense response	
GO_REGULATION_OF_MACROPHAGE_CHEMOTAXIS	NIR	immune system	myeloid lineage	
GO_REGULATION_OF_MONONUCLEAR_CELL_MIGRATION	NIR	immune system	myeloid lineage	
GO_VITAMIN_BINDING	NIR	metablolism	oxidoreduction	
GO_L_ASCORBIC_ACID_BINDING	NIR	metablolism	oxidoreduction	
GO_DIOXYGENASE_ACTIVITY	NIR	metablolism	oxidoreduction	
GO_PEPTIDYL_PROLINE_MODIFICATION	NIR	metablolism	oxidoreduction	
CORPORATION_OR_REDUCTION_OF_MOLECULAR_OXYGEN_2_OXOGLUTARATE_AS_ONE_DONOR_AND_IN	NIR	metablolism	oxidoreduction	
GO_POSITIVE_REGULATION_OF_CARBOHYDRATE_METABOLIC_PROCESS	NIR	metabolism	carbonhydrate	
GO_REGULATION_OF_CARBOHYDRATE_METABOLIC_PROCESS	NIR	metabolism	carbonhydrate	
GO_OXIDOREDUCTASE_ACTIVITY_ACTING_ON_A_HEME_GROUP_OF_DONORS	PIR	metabolism	cellular respiration	
GO_MITOCHONDRIAL_ELECTRON_TRANSPORT_CYTOCHROME_C_TO_OXYGEN	PIR	metabolism	cellular respiration	
GO_HYDROGEN_ION_TRANSMEMBRANE_TRANSPORT	PIR	metabolism	cellular respiration	
GO_RESPIRATORY_CHAIN	PIR	metabolism	cellular respiration	
GO_CYTOCHROME_COMPLEX	PIR	metabolism	cellular respiration	
GO_HYDROGEN_ION_TRANSMEMBRANE_TRANSPORTER_ACTIVITY	PIR	metabolism	cellular respiration	
GO_REGULATION_OF_CALCIUM_ION_DEPENDENT_EXOCYTOSIS	PIR	metabolism	cytosis	exo-
GO_REGULATION_OF_SYNAPTIC_VESICLE_EXOCYTOSIS	PIR	metabolism	cytosis	exo-
GO_MEMBRANE_INVAGINATION	NIR	metabolism	cytosis	phago-
GO_PHAGOCYTOSIS_ENGULFMENT	NIR	metabolism	cytosis	phago-
GO_GLUCURONATE_METABOLIC_PROCESS	PIR	metabolism	others	
GO_URONIC_ACID_METABOLIC_PROCESS	PIR	metabolism	others	
GO_SULFURIC_ESTER_HYDROLASE_ACTIVITY	NIR	metabolism	others	
GO_ENDOPLASMIC_RETICULUM_LUMEN	NIR	metabolism	others	
GO_RESPONSE_TO_TOPOLOGICALLY_INCORRECT_PROTEIN	NIR	metabolism	protein	
GO_RETROGRADE_PROTEIN_TRANSPORT_ER_TO_CYTOSOL	NIR	metabolism	protein	
GO_RESPONSE_TO_ENDOPLASMIC_RETICULUM_STRESS	NIR	metabolism	protein	
GO_ERAD_PATHWAY	NIR	metabolism	protein	
GO_REGULATION_OF_ENDOPLASMIC_RETICULUM_UNFOLDED_PROTEIN_RESPONSE	NIR	metabolism	protein	
GO_ENDOPLASMIC_RETICULUM_TO_CYTOSOL_TRANSPORT	NIR	metabolism	protein	
GO_PROTEIN_EXIT_FROM_ENDOPLASMIC_RETICULUM	NIR	metabolism	protein	
GO_CELLULAR_RESPONSE_TO_TOPOLOGICALLY_INCORRECT_PROTEIN	NIR	metabolism	protein	
GO_ER_ASSOCIATED_UBIQUITIN_DEPENDENT_PROTEIN_CATABOLIC_PROCESS	NIR	metabolism	protein	
GO_AMINOGLYCAN_METABOLIC_PROCESS	NIR	metabolism	proteoglycan	
GO_HEPARAN_SULFATE_PROTEOGLYCAN_BIOSYNTHETIC_PROCESS	NIR	metabolism	proteoglycan	
GO_PROTEOGLYCAN_METABOLIC_PROCESS	NIR	metabolism	proteoglycan	
GO_HEPARAN_SULFATE_PROTEOGLYCAN_METABOLIC_PROCESS	NIR	metabolism	proteoglycan	
GO_AMINOGLYCAN_BIOSYNTHETIC_PROCESS	NIR	metabolism	proteoglycan	
GO_REGULATION_OF_ALCOHOL_BIOSYNTHETIC_PROCESS	NIR	metabolism	sterols	
GO_NEGATIVE_REGULATION_OF_STEROID_METABOLIC_PROCESS	NIR	metabolism	sterols	
GO_NEGATIVE_REGULATION_OF_LIPID_BIOSYNTHETIC_PROCESS	NIR	metabolism	sterols	
GO_REGULATION_OF_STEROID_METABOLIC_PROCESS	NIR	metabolism	sterols	
GO_NEGATIVE_REGULATION_OF_ALCOHOL_BIOSYNTHETIC_PROCESS	NIR	metabolism	sterols	
GO_POSITIVE_REGULATION_OF_CARTILAGE_DEVELOPMENT	NIR	organogenesis	bone development	cartilage development
GO_CHONDROCYTE_DEVELOPMENT	NIR	organogenesis	bone development	cartilage development
GO_CONNECTIVE_TISSUE_DEVELOPMENT	NIR	organogenesis	bone development	cartilage development
GO_POSITIVE_REGULATION_OF_CHONDROCYTE_DIFFERENTIATION	NIR	organogenesis	bone development	cartilage development
GO_BONE_MORPHOGENESIS	NIR	organogenesis	bone development	cartilage development
GO_REGULATION_OF_CHONDROCYTE_DIFFERENTIATION	NIR	organogenesis	bone development	cartilage development
GO_ENDOCHONDRAL_BONE_MORPHOGENESIS	NIR	organogenesis	bone development	cartilage development
GO_CHONDROCYTE_DIFFERENTIATION	NIR	organogenesis	bone development	cartilage development
GO_CARTILAGE_DEVELOPMENT_INVOLVED_IN_ENDOCHONDRAL_BONE_MORPHOGENESIS	NIR	organogenesis	bone development	cartilage development
GO_CARTILAGE_DEVELOPMENT	NIR	organogenesis	bone development	cartilage development
GO_REGULATION_OF_CARTILAGE_DEVELOPMENT	NIR	organogenesis	bone development	cartilage development
GO_REGULATION_OF_OSTEOBLAST_DIFFERENTIATION	NIR	organogenesis	bone development	ossfication
GO_POSITIVE_REGULATION_OF_OSSIFICATION	NIR	organogenesis	bone development	ossfication
GO_POSITIVE_REGULATION_OF_OSTEOBLAST_DIFFERENTIATION	NIR	organogenesis	bone development	ossfication
GO_SYNCYTIUM_FORMATION	NIR	organogenesis	muscle development	
GO_MYOBLAST_FUSION	NIR	organogenesis	muscle development	
GO_STRIATED_MUSCLE_CELL_DIFFERENTIATION	NIR	organogenesis	muscle development	
GO_CARDIAC_MUSCLE_CELL_DIFFERENTIATION	NIR	organogenesis	muscle development	
GO_SUBSTANTIA_NIGRA_DEVELOPMENT	PIR	organogenesis	neural development	
GO_NEURAL_NUCLEUS_DEVELOPMENT	PIR	organogenesis	neural development	
GO_NEURON_PROJECTION_REGENERATION	NIR	organogenesis	regeneration	
GO_NEURON_PROJECTION_REGENERATION	NIR	organogenesis	regeneration	
GO_ORGAN_REGENERATION	NIR	organogenesis	regeneration	
GO_REGENERATION	NIR	organogenesis	regeneration	

GO_ORGAN_REGENERATION	NIR	organogenesis	regeneration
GO_SERTOLI_CELL_DEVELOPMENT	NIR	physiological function	fertility
GO_SERTOLI_CELL_DIFFERENTIATION	NIR	physiological function	fertility

Supplementary Data 3. Classification of all eight samples with scRNA-seq data available into NIR or PIR clusters.

				Cohort 1			Cohort 2			Cohort 3			Cohort 4		
	**total foldchange**	**Cluster**	**macrophage proportion**	**PIR**	**NIR**	**foldchange**	**PIR**	**NIR**	**foldchange**	**PIR**	**NIR**	**foldchange**	**PIR**	**NIR**	**foldchange**
**PJ016**	0.957350139	NIR	0.06%	0.8858871	0.8665323	1.02233597	0.8816182	0.9370079	0.9408867	0.9133958	0.917739	0.995267526	1	1	1
**PJ017**	0.622907489	NIR	46.63%	0.0846774	0.0302419	2.8	0.0045707	0.0205453	0.22246696	1	1	1	1	1	1
**PJ018**	3.506670298	PIR	2.28%	0.3677419	0.2423387	1.517470882	1	1	1	0.1408291	0.1198263	1.175277252	0.1920917	0.0976955	1.9662296
**PJ025**	4.050609406	PIR	1.70%	0.0697581	0.0177419	3.931818182	1	1	1	0.4597608	0.4462775	1.030212797	1	1	1
**PJ030**	1.629268176	PIR	8.33%	0.3112903	0.275	1.131964809	0.1394696	0.1247172	1.118287373	0.2588727	0.250705	1.032579186	0.1355575	0.1087528	1.246473
**PJ032**	0.900833333	NIR	55.12%	1	1	1	0.1956738	0.2172142	0.900833333	1	1	1	1	1	1
**PJ035**	1.161568474	PIR	8.12%	0.2616935	0.2516129	1.040064103	0.1916916	0.1477057	1.297794118	1	0.0161735	1	0.2324331	0.2700966	0.8605555
**PJ048**	0.27266869	NIR	0%	1	1	1	1	1	1	0.0043756	0.0160438	0.272727273	0.2125374	0.2125831	0.9997852

Negative coefficient value is not taken into consideration. To simplify the caculation, those values were replaced with 1.

Supplementary Data 4. Clinical information and IHC staning results for 12 patients.

ID	OS	OS_Censor	PFS	PFS_Censor	MS4A4A	Grade
CGGA_1481	4.37	1	0.933333333	1	27.579	4
CGGA_P25	4.90	1	4.9	1	35.905	4
CGGA_1422	6.80	1	6.7	1	29.481	4
CGGA_1521	6.83	1	5.833333333	1	21.728	4
CGGA_P143	8.70	1	8.7	1	41.402	4
CGGA_1494	8.97	1	8.966666667	1	21.35	4
CGGA_P178	25.87	0	25.86666667	0	0.853	4
CGGA_1735	27.10	1	22	1	1.516	4
CGGA_1467	28.87	1	20.76666667	1	1.596	4
CGGA_1282	37.20	1	20	1	1.241	4
CGGA_1780	37.43	0	35.56666667	1	0.818	4
CGGA_1086	65.9	1	42.23333333	1	2.013	4

References:
Aran D, Hu Z, Butte AJ. xCell: digitally portraying the tissue cellular heterogeneity landscape. Genome Biol. 2017;18(1):220. doi:10.1186/s13059-017-1349-1
Bindea G, Mlecnik B, Tosolini M, et al. Spatiotemporal Dynamics of Intratumoral Immune Cells Reveal the Immune Landscape in Human Cancer. Immunity. 2013;39(4):782-795. doi:10.1016/j.immuni.2013.10.003
Charoentong P, Finotello F, Angelova M, et al. Pan-cancer Immunogenomic Analyses Reveal Genotype-Immunophenotype Relationships and Predictors of Response to Checkpoint Blockade. Cell Rep. 2017;18(1):248-262. doi:10.1016/j.celrep.2016.12.019
Rooney MS, Shukla SA, Wu CJ, Getz G, Hacohen N. Molecular and Genetic Properties of Tumors Associated with Local Immune Cytolytic Activity. Cell. 2015;160(1-2):48-61. doi:10.1016/j.cell.2014.12.033
Tirosh I, Izar B, Prakadan SM, et al. Dissecting the multicellular ecosystem of metastatic melanoma by single-cell RNA-seq. Science. 2016;352(6282):189-196. doi:10.1126/science.aad0501
